# Macroevolutionary diversity of traits and genomes in the model yeast genus *Saccharomyces*

**DOI:** 10.1038/s41467-023-36139-2

**Published:** 2023-02-08

**Authors:** David Peris, Emily J. Ubbelohde, Meihua Christina Kuang, Jacek Kominek, Quinn K. Langdon, Marie Adams, Justin A. Koshalek, Amanda Beth Hulfachor, Dana A. Opulente, David J. Hall, Katie Hyma, Justin C. Fay, Jean-Baptiste Leducq, Guillaume Charron, Christian R. Landry, Diego Libkind, Carla Gonçalves, Paula Gonçalves, José Paulo Sampaio, Qi-Ming Wang, Feng-Yan Bai, Russel L. Wrobel, Chris Todd Hittinger

**Affiliations:** 1 Laboratory of Genetics, J. F. Crow Institute for the Study of Evolution, Wisconsin Energy Institute, Genome Center of Wisconsin, University of Wisconsin-Madison, Madison, WI USA; 2 DOE Great Lakes Bioenergy Research Center, University of Wisconsin-Madison, Madison, WI USA; 3 Biotechnology Center, University of Wisconsin-Madison, Madison, WI USA; 4 University of Utrecht, Utrecht, The Netherlands; 5 Department of Biology, University of Rochester, Rochester, NY USA; 6 Departement des Sciences Biologiques, Université de Montréal, Montreal, QC Canada; 7 Département de Biologie, PROTEO, Pavillon Charles‑Eugène‑Marchand, Institut de Biologie Intégrative et des Systèmes (IBIS), Université Laval, Quebec City, QC Canada; 8 Canada Natural Resources, Laurentian Forestry Centre, Quebec City, QC Canada; 9 Centro de Referencia en Levaduras y Tecnología Cervecera (CRELTEC), Instituto Andino Patagónico de Tecnologías Biológicas y Geoambientales (IPATEC), Consejo Nacional de Investigaciones, Científicas y Técnicas (CONICET)-Universidad Nacional del Comahue, Bariloche, Argentina; 10 Associate Laboratory i4HB - Institute for Health and Bioeconomy, NOVA School of Science and Technology, Universidade NOVA de Lisboa, Caparica, Portugal; 11 UCIBIO-i4HB, Departamento de Ciências da Vida, Faculdade de Ciências e Tecnologia, Universidade Nova de Lisboa, Caparica, Portugal; 12 Vanderbilt University, Department of Biological Sciences, Nashville, TN USA; 13 Evolutionary Studies Initiative, Vanderbilt University, Nashville, TN USA; 14 School of Life Sciences, Institute of Life Sciences and Green Development, Hebei University, Baoding, China; 15 State Key Laboratory of Mycology, Institute of Microbiology, Chinese Academy of Sciences, Beijing, China; 16 Present Address: Section for Genetics and Evolutionary Biology, Department of Biosciences, University of Oslo, Oslo, Norway; 17 Present Address: Department of Food Biotechnology, Institute of Agrochemistry and Food Technology (IATA), CSIC, Valencia, Spain

**Keywords:** Evolutionary genetics, Phylogenetics, Population genetics, Food microbiology, Saccharomyces cerevisiae

## Abstract

Species is the fundamental unit to quantify biodiversity. In recent years, the model yeast *Saccharomyces cerevisiae* has seen an increased number of studies related to its geographical distribution, population structure, and phenotypic diversity. However, seven additional species from the same genus have been less thoroughly studied, which has limited our understanding of the macroevolutionary events leading to the diversification of this genus over the last 20 million years. Here, we show the geographies, hosts, substrates, and phylogenetic relationships for approximately 1,800 *Saccharomyces* strains, covering the complete genus with unprecedented breadth and depth. We generated and analyzed complete genome sequences of 163 strains and phenotyped 128 phylogenetically diverse strains. This dataset provides insights about genetic and phenotypic diversity within and between species and populations, quantifies reticulation and incomplete lineage sorting, and demonstrates how gene flow and selection have affected traits, such as galactose metabolism. These findings elevate the genus *Saccharomyces* as a model to understand biodiversity and evolution in microbial eukaryotes.

## Abstract (web summary)

Here, the authors describe the geographies, hosts, substrates, and phylogenetic relationships for 1,794 *Saccharomyces* strains. They provide insight into the genetic and phenotypic diversity in the genus, not seen through prior work focused on the model species *Saccharomyces cerevisiae*.

## Introduction

Global climate change is expected to significantly impact biodiversity and human health^1^. Thus, it is increasingly important to catalog and understand the origins of biological diversity. While the species is the fundamental unit to quantify biodiversity from a biological perspective^2^, the study of only one or a few representatives of each species biases our understanding of the true diversity of a species^3^. This limitation is especially problematic when current species delineations are not in full agreement with the boundaries of gene flow or when traits vary widely within a species^4^. Phenotypes can vary within a species or genus due to gene flow, selection, or other evolutionary processes^5^. Thus, it is vital that the scientific community quantifies biodiversity and strives to understand both its ecological and evolutionary contexts.

Quantifying and understanding the origins of biodiversity will advance fundamental science while also identifying and prioritizing bioresources that contribute to food, medicine, fuels, and other value-added compounds^2^. Whole genome sequencing has empowered researchers in this endeavor, and ongoing initiatives, such as the Earth BioGenome Project and the European Reference Genome Atlas (ERGA), envision cataloging most of the individual species on Earth^6,7^. Unfortunately, these studies are particularly biased toward multicellular organisms, such as insects, vertebrates, and plants, for which multiple species have been identified, geographic patterns have been described, and phenotypic traits are often visible^6^. In other species, such as microbial eukaryotes, macroevolutionary processes have been less thoroughly studied and received less attention for species- or genus-wide genome sequencing efforts. Nonetheless, microbial eukaryotes, such as yeasts, are great model organisms due to their small genomes, ease of genetic manipulation, and a large number of genes that are orthologous with multicellular eukaryotes^8^.

A major factor in the lack of quantification of eukaryotic microbes has been the influence of the hypothesis proposed by Baas Becking in 1934 and promulgated by Beijerinck that “everything is everywhere, but, the environment selects”^9^. Nevertheless, expanded strain isolation from the wild and genome sequencing have shown that eukaryotic microbes, like multicellular organisms, also have geographical structure^10,11^. While large-scale whole genome sequencing studies have investigated the evolutionary history of the model yeast *Saccharomyces cerevisiae* and its closest relative, *Saccharomyces paradoxus*^12–14^, the six other non-hybrid *Saccharomyces* species (*Saccharomyces mikatae*, *Saccharomyces jurei, Saccharomyces kudriavzevii*, *Saccharomyces arboricola*, *Saccharomyces uvarum*, and *Saccharomyces eubayanus*) have been less thoroughly studied^15–18^. In particular, several new and diverse lineages of *Saccharomyces* have recently been delineated^13,14,19–28^, but the genetic and phenotypic diversities of each species have not been studied in a comparative context^29^, which has limited our understanding of the macroevolutionary processes driving diversification in this important genus.

In this work, we cover the genetic and phenotypic diversity of the model eukaryotic genus *Saccharomyces* with unprecedented breadth and depth—reporting geographies, hosts, substrates, and phylogenetic relationships for ~1800 *Saccharomyces* strains. We generate and analyze high-quality genome sequences for representative strains of all available phylogenetic lineages, and we sequence and phenotype more than a hundred *Saccharomyces* strains to quantify the genetic and phenotypic variation across this macroevolutionary timescale (13.3–19.3 million years^30^). With this global dataset, we quantify diversity and divergence within and between species and populations, several types of natural reticulation events, and the influences of ecology and incomplete lineage sorting. This work elevates the genus *Saccharomyces* as a model for understanding biodiversity, population structure, and macroevolutionary processes in microbial eukaryotes. This fundamental understanding also provides a much-needed framework for identifying and prioritizing key bioresources.

## Results

### The Palearctic and Fagales preponderance of *Saccharomyces*

**Fig. 1:**
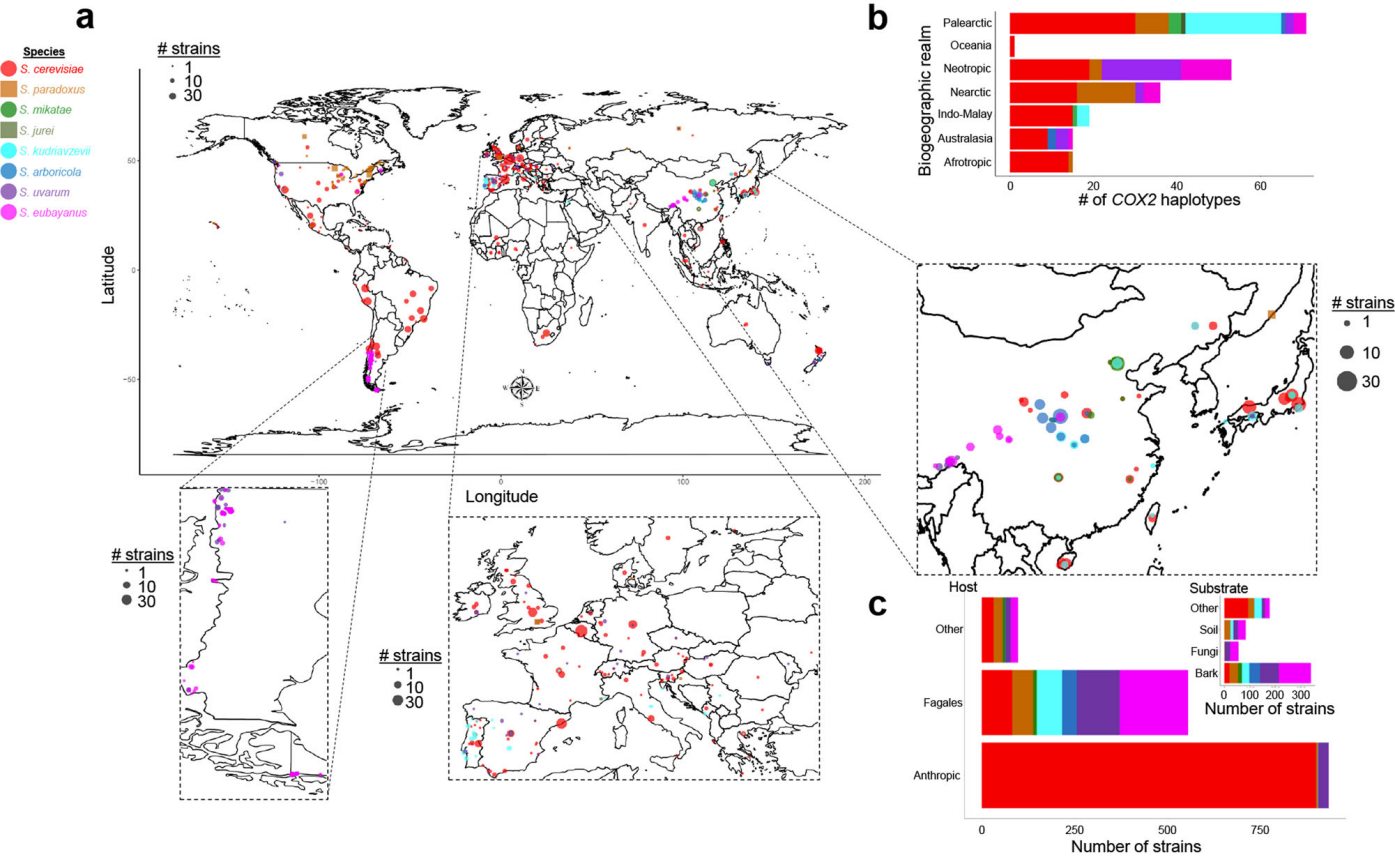
Geographic distribution of *Saccharomyces* strains. **a** Map showing the locations where wild non-interspecies hybrid *Saccharomyces* strains (*n* = 681 strains, Supplementary Data 1) have been isolated, scaled by size to the number of strains studied here. Symbols and colors designate the species. Ecological and geographic information about the strains can be found in Supplementary Data 1. The map was generated using the map_data function implemented in the R package ggplot2 ^125^. **b** Stacked bar plot showing the number of *COX2* haplotypes (*n* = 138 haplotypes including 1776 *COX2* sequences, Supplementary Data 1) isolated in each biogeographic realm (Fig. 2a). The data shows many *COX2* haplotypes from the Palearctic region, pointing to Asia as a hotspot of diversity. Bars are colored by species. **c** Bar plots represent the total number of non-interspecies hybrid strains, with both host and substrate information annotated (*n* = 1643 strains), from each *Saccharomyces* species grouped by the host (external plot) or substrates (inner plot) (full details in Supplementary Data 1 and Supplementary Fig. 2). Human-related environments, such as vineyards, were grouped in the “Anthropic” host category and they were not included in the substrate plot (*n* = 652 strains with completed information). Bar plots are colored according to species.

To place newly isolated *Saccharomyces* strains in the context of existing datasets^12,13,18,23–25,31–33^, we partially sequenced 275 *COX2* and 129 *COX3* mitochondrial genes from key strains. In total, we analyzed the mitochondrial sequences of ~1800 *Saccharomyces* strains isolated mostly from bark substrates (52% of wild isolates) from multiple continents (Fig. 1a, c, Supplementary Fig. 1, 2, and Supplementary Data 1). Across the genus, 85% of wild isolates were associated with the order Fagales, which includes oak and beech trees. In contrast, 89% of *S. cerevisiae* strains were isolated from anthropic environments (Fig. 1c and Supplementary Fig. 2a).

**Fig. 2:**
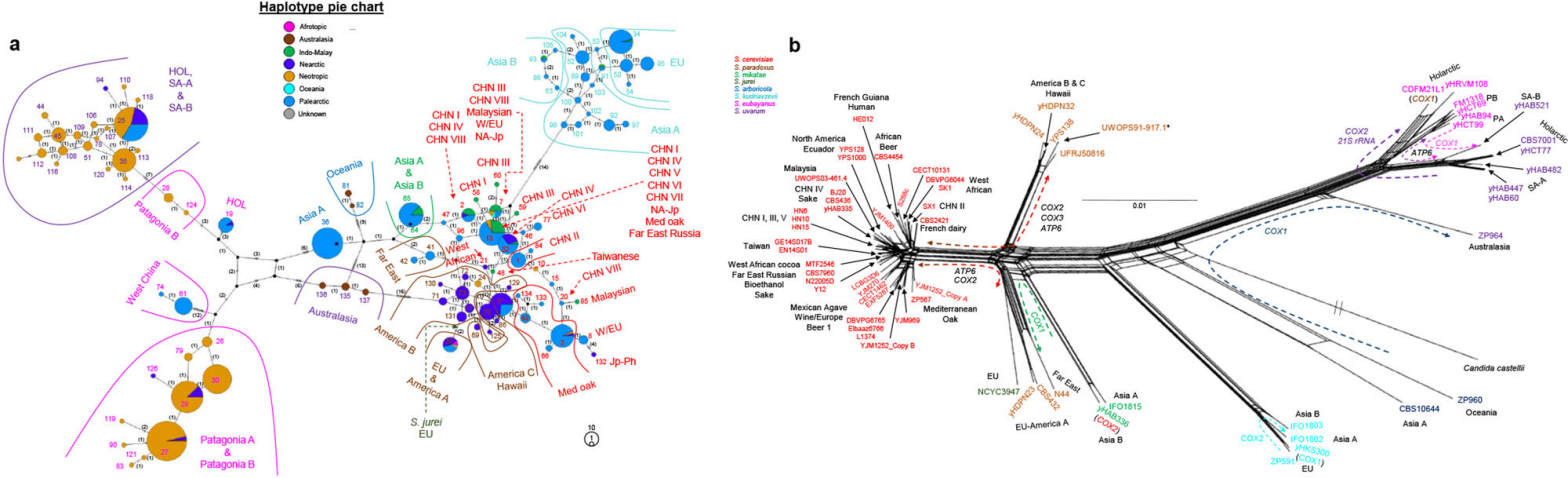
Extensive mitochondrial gene flow and introgression between *Saccharomyces* lineages. **a** Templeton, Crandall, and Sing (TCS) phylogenetic network of 739 partial *COX2* sequences from wild *Saccharomyces* strains. *COX2* haplotype classification, for the wild and anthropic *Saccharomyces* strains (*n* = 1774 *COX2* sequences), is shown in Supplementary Data 1. Haplotypes are represented by circles. The circle size is scaled according to the haplotype frequency. Pie charts show the frequency of haplotypes based on the biogeographic realm. The number of mutations separating each haplotype are indicated by lines on the edges connecting different haplotype circles and by numbers between parentheses. Haplotype numbers and populations are highlighted in the panel and colored according to species designations. CHN China, EU Europe, HOL Holarctic, Jp-Ph Japan-Philippines (=Sake-Philippines), Med oak Mediterranean oak, NA-Jp North America-Japan (=North America), SA-A South America A, SA-B South America B, W/EU Wine/European. **b** Neighbor-Net phylogenetic network reconstructed using a concatenated alignment of the coding sequences of ten mitochondrial genes (*ATP6*, *ATP8*, *ATP9*, *COB*, *COX1*, *COX2*, *COX3*, *VAR1*, and the genes encoding 15 S rRNA and 21 S rRNA*)* for 64 sequenced *Saccharomyces* strains representing all known *Saccharomyces* lineages that were available (Supplementary Data 1, 2). Strain names are colored according to species designations. Population names are highlighted in black. The scale is given in nucleotide substitution per site. Dashed lines with arrows highlight mitochondrial gene flow (intraspecies) and introgressions (interspecies) detected from individual gene trees (Supplementary Fig. 4); affected genes are shown close to the arrows with the color indicated by the species donor. Gene flow and introgressions unique to a *Saccharomyces* strain are indicated between parentheses. A similar phylogenetic network for the *COX3* mitochondrial gene is shown in Supplementary Fig. 3, which is more congruent with the concatenated data shown in panel **b** than the data for *COX2* shown in panel **a**. The asterisk indicates that UWOPS91-917.1 did not contain the introgression of *COX3* from *S. cerevisiae* found in other *Saccharomyces paradoxus* America B and C strains. Most of the *Saccharomyces jurei* (NCYC3947) protein-coding sequences were more closely related to the *S. paradoxus* Far East-EU clade, rather than to *Saccharomyces mikatae* (Supplementary Fig. 4).

A large number of haplotypes were inferred for *COX2* (Figs. 1b, 2a) and *COX3* (Supplementary Fig. 3 and Supplementary Data 1). Our results indicated that the Palearctic biogeographic realm, which includes China and Europe, contained haplotypes from all species and more haplotypes than any other biogeographic realm (Fig. 1b). The centrality of Palearctic *COX2* haplotypes in the phylogenetic network (Fig. 2a) corroborates the hypothesis that many *Saccharomyces* lineages originated in this region, particularly East Asia^25,28,34,35^.

### Genomic structural variation is common between *Saccharomyces* lineages

From our global *Saccharomyces* collection, we sequenced and assembled 22 high-quality genomes, including representatives for each major phylogenetic lineage (Supplementary Data 2). Note, we consider yeast lineages to be clades of strains with shared ancestries that have frequently interbred, even though they are not strictly panmictic populations. These assemblies had nearly complete chromosomes with additional unplaced scaffolds ranging from 0 to 39 (Supplementary Data 2). We also included 16 previously published assemblies, one of which we substantially improved, bringing the total here to 38 high-quality genome assemblies (Supplementary Data 1, 2). In addition, we generated sixteen complete mitochondrial genome assemblies, corrected the size of the previously published *S. jurei* mitochondrial genome^18^, and assembled two new 2-µm plasmids (Supplementary Data 2). Structurally, species varied by GC contents, chromosome lengths, mitochondrial genome sizes, and the synteny of nuclear and mitochondrial genomes, usually due to a modest number of translocations (Supplementary Figs. 5–8 and Supplementary Note 1).

### Analyses revealed new *Saccharomyces* lineages and populations

To better illuminate population-level diversity, especially for previously under-sampled species, 163 sequenced *Saccharomyces* strains were analyzed using several population and phylogenomic approaches (Supplementary Data 2, see Methods). Our analyses revealed new populations and cryptic lineages of *S. kudriavzevii* and of *S. mikatae* (Supplementary Fig. 9c, d). Note that populations are supported by STRUCTURE and fineSTRUCTURE analyses. Two *S. kudriavzevii* strains, originally isolated in China, belonged to a newly identified lineage (Supplementary Fig. 9d), but they had fewer fixed differences compared to European (EU) strains (5.5 thousand SNPs) than to strains from the Asia A lineage (10.2 thousand SNPs). In haplotype and phylogenetic networks, mitochondrial gene sequences for these two strains were located between Asia A and EU haplotypes or unexpectedly close to Asia A (Fig. 2a and Supplementary Figs. 3, 4b, e). Interestingly, despite the geographic proximity of this lineage to Asia A, only ~12% of the nuclear genome of these strains was more divergent from EU than from the Asia A *S. kudriavzevii* population (Supplementary Data 3 and Supplementary Fig. 9d, 10hi-ii), suggesting that these strains are descendants of an ancestral admixture event. Two distinct populations were revealed for *S. mikatae*, one of which (Asia A) may have up to three cryptic lineages and a large number of segregating polymorphisms (Supplementary Fig. 9c), possibly from lineages yet to be discovered.

### Differentiation and divergence of *Saccharomyces* lineages and species

**Fig. 3:**
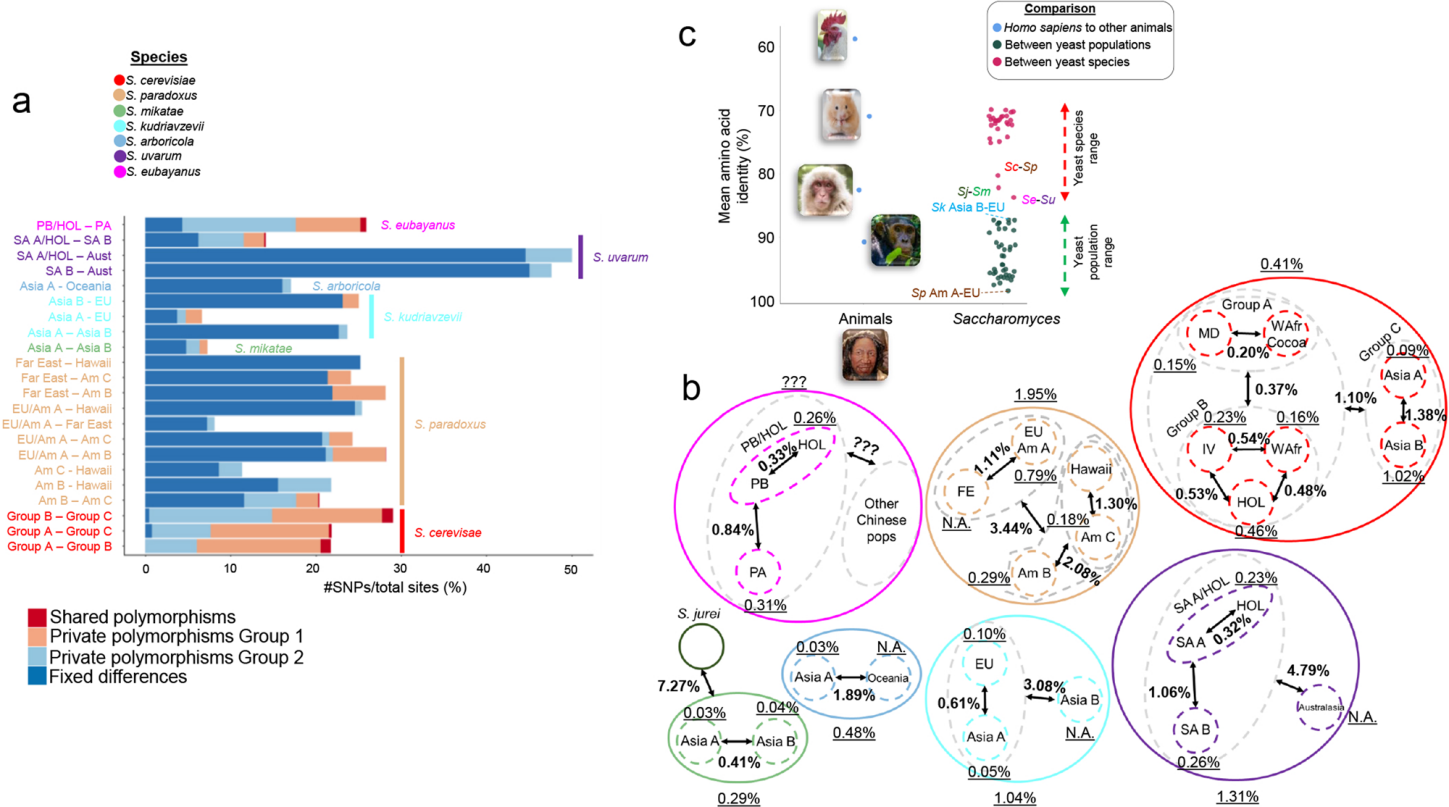
Species and population-level diversity in *Saccharomyces*. **a** Percentage of private segregating polymorphisms, fixed differences, and shared polymorphisms among SNPs found in pairwise comparisons between supported populations, except for *S. cerevisiae* where populations were grouped according to PCA and co-ancestry for better resolution (Supplementary Fig. 9a iv and v). **b** Global picture of the percentage of the Tamura-Nei-corrected pairwise genetic distance between populations and within *Saccharomyces* species and populations. Arrow width and length do not have a direct connection with genetic distance.???: values cannot be inferred because West China and Sichuan strains were unavailable for whole genome sequencing. N.A. not applicable because only one strain was available from this population. In panels **a**, **b** for *S. cerevisiae*, to simplify (Supplementary Fig. 9a), we grouped the previously described populations in three main groups: A (MD group [see below] and West African cocoa population), B (HOL group [see below], China IV, and West African populations), and C (Asia A [Taiwan and China IX populations] and B groups [China I and II populations]). **c** Right: dot plot of mean amino acid identities (AAI) calculated from pairwise comparisons between populations and between species. Left: dot plot for comparisons of *Homo sapiens* (free use picture under Wikimedia commons license; we did not modify this or any image) with *Pan troglodytes* (Francesco Ungaro’s picture, free use under Pexels license), *Macaca mulatta* (Howling Red’s picture, free use under Unsplash license), *Mus musculus* (Ricky Kharawala’s picture, free use under Unsplash license), and *Gallus gallus* (Wolfgang Hasselmann’s picture, free use under Unsplash license). Am America, EU Europe, FE Far East, HOL Holarctic (contains Asian Islands, China III/V, Ecuador, Far East Russian, French Guiana Human, Malaysian, Mexican Agave, and North American populations), MD Mediterranean domesticated (contains African beer, alpechin, beer1, beer 2, biofuel, French dairy, Laboratory, mantou, Mediterranean oak, sake, and Wine/European populations), SA-A South America A, SA-B South America B, *Sc* *S. cerevisiae,* *Se* *S. eubayanus,* *Sj* *S. jurei*, *Sm* *S. mikatae*, *Sp* *S. paradoxus*, WAfr West African, IV China IV. *Saccharomyces* strains used for each panel are indicated in Supplementary Data 1.

Studying all *Saccharomyces* species together, we inferred two or more populations, with an average of about 3 populations per species (Fig. 3 and Supplementary Fig. 9), except for *S. cerevisiae*, due partly to its multiple domestication events. *S. cerevisiae*, with 16 or more populations and extensive admixture^13,19,25,36,37^, had relatively low genetic diversity compared to other species, with an average genetic distance only slightly higher than *S. mikatae* (Fig. 3b and Supplementary Fig. 11j). Despite the low sequence diversity, phenotypic and ecological factors better differentiated *S. cerevisiae* into distinct lineages or populations than in the other *Saccharomyces* species (Fig. 3b and Supplementary Fig. 9a^13,23^). In contrast, *Saccharomyces paradoxus* was the most diverse species (1.95% average pairwise divergence), followed by *S. kudriavzevii* and *S. uvarum* (Fig. 3b and Supplementary Fig. 11j, i). *S. eubayanus* likely has diversity levels similar to *S. uvarum*^24^, but the Sichuan and West China lineages^22^ were not available for genome sequencing. At the nuclear level, each species was separated from its closest relative by genetic divergence of ~7–11% (Supplementary Fig. 11a–d, g, h, k), except for *S. arboricola* and *S. kudriavzevii*, where both showed genetic divergences higher than 18% with the rest of *Saccharomyces* species (Supplementary Fig. 11e, f, k,). At the mitochondrial level, the median genetic distances of coding sequences were in general lower than the nuclear coding sequences (Supplementary Fig. 11i), as expected for fungal organisms^38^, except for *S. arboricola*, where we were only able to explore two strains (one from Asia A and one from Oceania). Note, that intergenic regions were not analyzed here due to incomplete genome assemblies, but these regions are more variable than coding sequences. Nuclear and mitochondrial genetic distances between the closest species were lower than comparisons with other species; genetic divergence was close to ~2% for the mitochondrial genome, whereas it was higher than 7% for the nuclear genome. There was one exception: the *S. jurei* mitochondrial genome was more closely related to *S. paradoxus*, rather than *S. mikatae* (Supplementary Fig. 11k). The differentiation among *S. kudriavzevii*, *S. arboricola*, and *S. paradoxus* with other members of the genus, as measured by F_ST_, was considerably lower than other *Saccharomyces* species comparisons (Supplementary Fig. 12), an indication that these three species harbor more genetic variation that is not fixed.

In *Saccharomyces*, levels of <85% of amino acid identity (AAI) in a set of core single-copy eukaryotic genes differentiated species, while population-level AAI values were higher (Fig. 3c). The lowest AAI value within a species was the comparison between the Asia B and EU populations of *S. kudriavzevii*, whose value was between the AAI values of the *Homo sapiens/Pan troglodytes* and *Homo sapiens*/*Macaca mulatta* comparisons. *Saccharomyces paradoxus* America A versus EU produced the highest AAI value (Fig. 3c), which is consistent with the hypothesis that these populations were very recently derived due to migration from Europe to North America^39^. The maximum AAI between *Saccharomyces* species were those comparisons between *S. cerevisiae*-*S. paradoxus*, *S. jurei*-*S. mikatae*, and *S. eubayanus*-*S.uvarum*, with the latter showing the highest value. The minimum interspecies AAI value was comparable to the comparison between *Homo sapiens* and *Mus musculus* (<70% AAI).

### The non-nuclear genome is more permeable to introgression and gene flow than the nuclear genome

**Fig. 4:**
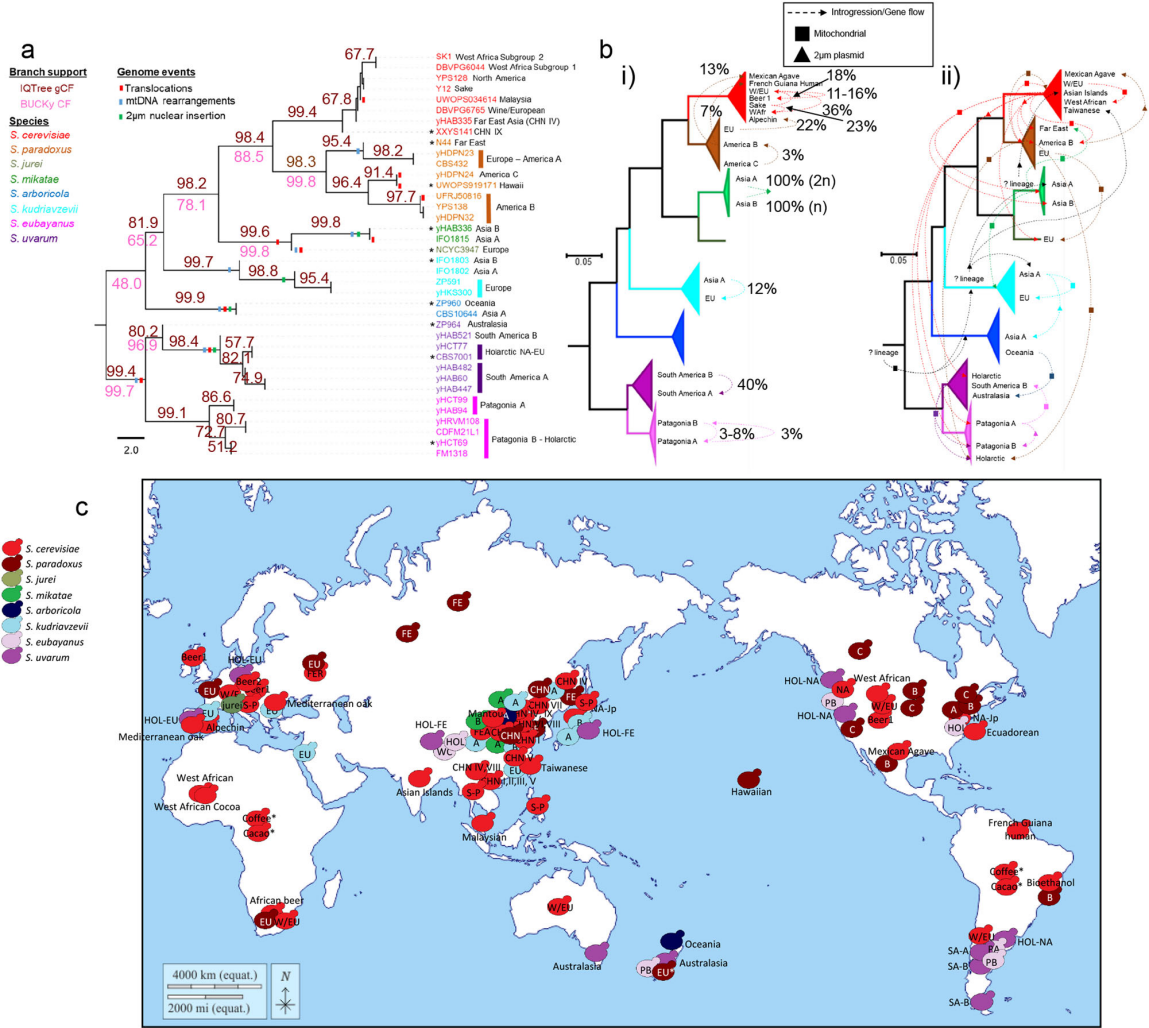
Vertical inheritance and ILS dominated in the nuclear genome, while gene flow was widespread among cytoplasmically inherited genetic elements. **a** Coalescent tree (species tree) for *Saccharomyces* lineages. Faint dotted lines connect the tips with the strain names. Two values of concordance factors (CFs) are shown. Brown gene CFs (gCFs) were generated by IQTree using a collection of Maximum-Likelihood phylogenetic trees (3850 genes) and the ASTRAL species tree. The normalized score was 0.97. Purple CFs were generated by BUCKy using representative strains. Other gene tree topologies are shown in Supplementary Fig. 13. Chromosomal translocations (Supplementary Fig. 5) and mitochondrial rearrangements (Supplementary Figs. 7, 8) are reported by red and blue bars on branches, respectively. The insertion of a 2-µm plasmid gene into the nuclear genome (Supplementary Data 4) is represented by green bars on branches. The scale is coalescent units. **b** Maximum-likelihood phylogenetic tree of all studied *Saccharomyces* strains reconstructed using the common BUSCO genes and collapsed to the species level (full tree in Fig. 5b). Scale bars show the number of substitutions per site. Population names are only shown for those involved in gene flow or introgression based on the genome-wide analysis. **b** **i** Summary of detected nuclear gene flow (between populations) and introgression (between species). The quantified percent of genome contribution by the donor is indicated near to the dashed arrow. *Saccharomyces cerevisiae* introgressions were congruent with previous reports ^13,19,66^. **b** **ii** summary of detected gene flow and introgression for the mitochondrial genome (squared symbol) and 2-µm plasmid (triangle symbol). The direction of the arrow points to the recipient lineage. Unknown donor lineages are colored in black in **b** **ii**). Branches and arrows are colored according to the species designations of their donors. Quantification of gene flow/introgression in cytoplasmic genomes is not provided due to the low number of completed genome assemblies for these genomes. **c** Geographic locations of the different wild *Saccharomyces* populations. The locations of populations, for which strains were not studied here, are indicated with an asterisk symbol. Yeast symbols are colored according to the species designation according to the left legend, and the population is written. Map from https://d-maps.com/carte.php?lib=world_pacific_ocean_centered_map&num_car=3226&lang=en under d-maps.com license.

To explore the stability of the relationships among *Saccharomyces* populations and species, we analyzed 38 high-quality nuclear genomes of representative strains using a phylogenomic framework to investigate 3850 conserved genes (Supplementary Data 1). The ASTRAL coalescent species tree and BUCKy concordance primary tree agreed with previous studies (Fig. 4a and Supplementary Fig. 13)^15,18,28,40^. Species-level branches were highly supported, while some branches close to the tips were not. Internal branch support values decreased outside of the *S. cerevisiae*-*S. paradoxus* clade and the *S. uvarum*-*S. eubayanus* clade, a phenomenon previously observed^30,41^ and proposed to be due to hybridization involving ancestors of *S. kudriavzevii* ^42^. Alternatively, the short coalescent units near the divergence of *S. arboricola* and *S. kudriavzevii* (Fig. 4a) and the low relative differentiation of *S. arboricola* and *S. kudriavzevii* with the rest of the species (Supplementary Fig. 12e, f) suggest a more nuanced model. Specifically, we propose that the conflicting data between genes are the result of diversification over a relatively narrow window of time, which allowed for the retention of considerable ancestral polymorphisms through incomplete lineage sorting (ILS), ancient gene flow between lineages in the early stages of speciation, or both. These patterns have been seen frequently across the tree of life^43^.

To further explore the phylogenetic stability of species boundaries, we applied reciprocal monophyly tests for each species using 3850 ML gene trees (Supplementary Data 5). *S. cerevisiae* and *S. paradoxus* only failed to be monophyletic in 17 and 57 genes, respectively. Gene flow from *S. cerevisiae* to *S. paradoxus* EU and America A were detected, as previously documented^44^, but the most frequent source of conflict was the location of the *S. cerevisiae* CHNIX lineage. This lineage sometimes grouped as an early-diverging member of the *S. paradoxus* clade or as an outgroup to both *S. cerevisiae* and *S. paradoxus*, topologies and branch lengths that are consistent with ILS. The *S. uvarum* Australasian lineage produced an even more striking pattern, again consistent with ILS, where more than 700 genes placed it as an early-diverging lineage of the *S. eubayanus* clade. At the species level, the Bayesian pipeline revealed many genes that supported alternative topologies, especially where the phylogenetic locations of *S. arboricola, S. kudriavzevii*, and the *S. mikatae/S. jurei* clade varied, and the consensus species tree was only supported by ~1824 genes (48% of a total of 3801 genes for this pipeline) (Supplementary Fig. 13). The presence of *Kluyveromyces lactis* in the dataset for the Bayesian pipeline, which was necessary to root the tree during phylogenetic reconstruction, might have decreased the support for internal branches in comparison with the ML pipeline (Fig. 4a).

This conflict can be recapitulated using phylogenetic networks reconstructed using genes in 38 high-quality genomes (Supplementary Data 1 and Supplementary Fig. 14a) annotated with the Yeast Genome Annotation Pipeline (YGAP) and using 14 BUSCO genes common to all (160 strains) phenotyped and previously sequenced strains (Supplementary Data 1 and Supplementary Fig. 14b). Collectively, these results support a model of a rapid radiation of some lineages with the retention of ancestral polymorphisms.

Within species, we observed much lower IQTree concordance factors at branches (Fig. 4a), which highlights ongoing gene flow within and between lineages. We next examined our sequenced and phenotyped strains (Supplementary Data 1) for genome-wide signals of gene flow between recognized lineages (Supplementary Figs. 9, 10). These analyses suggested that 27.16% of the *Saccharomyces* strains, from six of the eight species, showed evidence of admixture (Supplementary Fig. 9). Of these, 13.58% of the admixed strains were strongly supported (Supplementary Fig. 10 and Supplementary Data 3). The admixture was mostly observed in domesticated/anthropic *S. cerevisiae* strains where it was accompanied by higher levels of heterozygosity, which was generally low across the rest of the species (Supplementary Fig. 15). The genomic contributions of the minor parental donor averaged 18.30% in wild non-*S. cerevisiae* admixed strains (Fig. 4bi, Supplementary Fig. 10, and Supplementary Data 3). The smallest values belonged to two strains of *S. paradoxus* America C with contributions from America B, which were previously named the America C* lineage^14^, as well as two *S. eubayanus* strains. In the latter cases, one strain was from each Patagonian population, but it had genomic contributions from the other Patagonian population. The highest value of the genomic contribution by a minor donor in our dataset was found in a *S. uvarum* strain from South America B lineage, which had 39.53% of its genome from South America A origin (Fig. 4bi and Supplementary Fig. 10i) and a *S. mikatae* strain with full contributions of each parent (Supplementary Fig. 16d). These strains also showed high levels of heterozygosity for wild non-*S. cerevisiae* strains (Supplementary Fig. 15), further supporting recent admixture events. The low levels of heterozygosity for the rest of non-*S. cerevisiae* admixed strains might point to the rapid fixation of lineage-specific alleles following haploselfing, intratetrad mating, or a return-to-growth event. Although we found some evidence of gene flow between populations, rarer introgressions between species (Supplementary Fig. 16 and Supplementary Data 3, 13), and considerable evidence of incomplete lineage sorting, we conclude that the phylogenies of nuclear genes were generally consistent with the accepted species relationships.

We next tested how the species tree compared with phylogenies generated using the mitochondrial genome. A preliminary view of mitochondrial synteny among *Saccharomyces* immediately suggested the possibility of considerable incongruence. For example, mitochondrial genome synteny is conserved in *S. cerevisiae* and *S. paradoxus*, except in the EU–America A and the Far East populations of *S. paradoxus* (Supplementary Figs. 7, 8a^12,45^). The *S. jurei* nuclear genome was mostly syntenic with *S. mikatae* strains (Supplementary Fig. 5), but its mitochondrial genome was syntenic with the *S. paradoxus* EU and America A populations (Supplementary Fig. 8b) and differed from the *S. mikatae* Asia A population (Supplementary Fig. 7). The *S. uvarum* Australasian population and *S. eubayanus* were syntenic in both their nuclear and mitochondrial genomes (Supplementary Figs. 5, 8e), while the other *S. uvarum* populations inherited derived mitochondrial and nuclear rearrangements (Supplementary Figs. 5, 8d). At the nucleotide level, both *COX2* and *COX3* phylogenetic networks disagreed with the nuclear genome in some cases. In both mitochondrial phylogenetic networks, population haplotypes from some species were more closely related to other species’ haplotypes than to their same-species haplotypes (Fig. 2a and Supplementary Fig. 3) due to lineage-specific introgressions. For example, *S. paradoxus* America B and C strains were connected to *S. cerevisiae* haplotypes. Similarly, *S. eubayanus* West China and *S. uvarum* Australasian strains likely experienced introgressions. A phylogenetic network for mitochondrial genes of the 64 high-quality mitochondrial genomes (Supplementary Data 2) supported the broader *COX2* and *COX3* results (Fig. 2b, Supplementary Fig. 4). In addition to previously detected mitochondrial introgressions between species and gene flow between populations^45–49^, we also detected new cases of mitochondrial introgression and gene flow for *S. kudriavzevii*, *S. jurei*, and *S. mikatae* (Fig. 4bii and Supplementary Fig. 4). The *S. arboricola* and *S. kudriavzevii* mitochondrial genomes also had some affinity for the *Candida (Nakaseomyces) castellii* outgroup, as suggested by their exacerbated subtended edges in the network (Supplementary Fig. 4), so ancestral polymorphisms or introgression from unknown *Saccharomyces* lineages might have contributed to the mitochondrial genomes of these species. We conclude that increasing the number of strains from non-*S. cerevisiae* species of the *Saccharomyces* genus have provided more power to detect events of introgressions and gene flow between mitochondrial genomes, suggesting that these events have been more frequent than previously described (Fig. 4b).

Similarly, 22 interspecies transfers were detected for the 2-µm plasmid (Fig. 4bii and Supplementary Fig. 17), which is also cytoplasmically inherited. The *S. cerevisiae* 2-µm plasmid seems to be highly mobile, and we detected it in four other species. Sixteen strains had both cytoplasmic 2-µm plasmid genes and plasmid genes that had been transferred to the nuclear genome, a phenomenon previously noted for a handful of strains^50^ (Supplementary Data 4). We also detected a transfer from a hypothesized unknown source into the *S. cerevisiae* Taiwanese lineage^13^, as well as to a *S. mikatae* Asia A strain and a *S. kudriavzevii* Asia A strain (Supplementary Fig. 17a). Given its sister relationship with the previously detected *S. kudriavzevii* 2-µm plasmid, this unknown lineage may also be a close relative of *S. kudriavzevii* (Supplementary Fig. 17a).

Taken together, our results suggest that introgressions and gene flow involving the nuclear genome are limited in wild environments, while introgression and gene flow involving the cytoplasmically inherited mitochondrial genome and the 2-µm plasmid are much more frequent (Fig. 4), likely because they can occur without involving karyogamy^51^ or be aided by the activity of free-standing homing endonucleases^48,52^.

### Complex ancestries promote phenotypic diversity

**Fig. 5:**
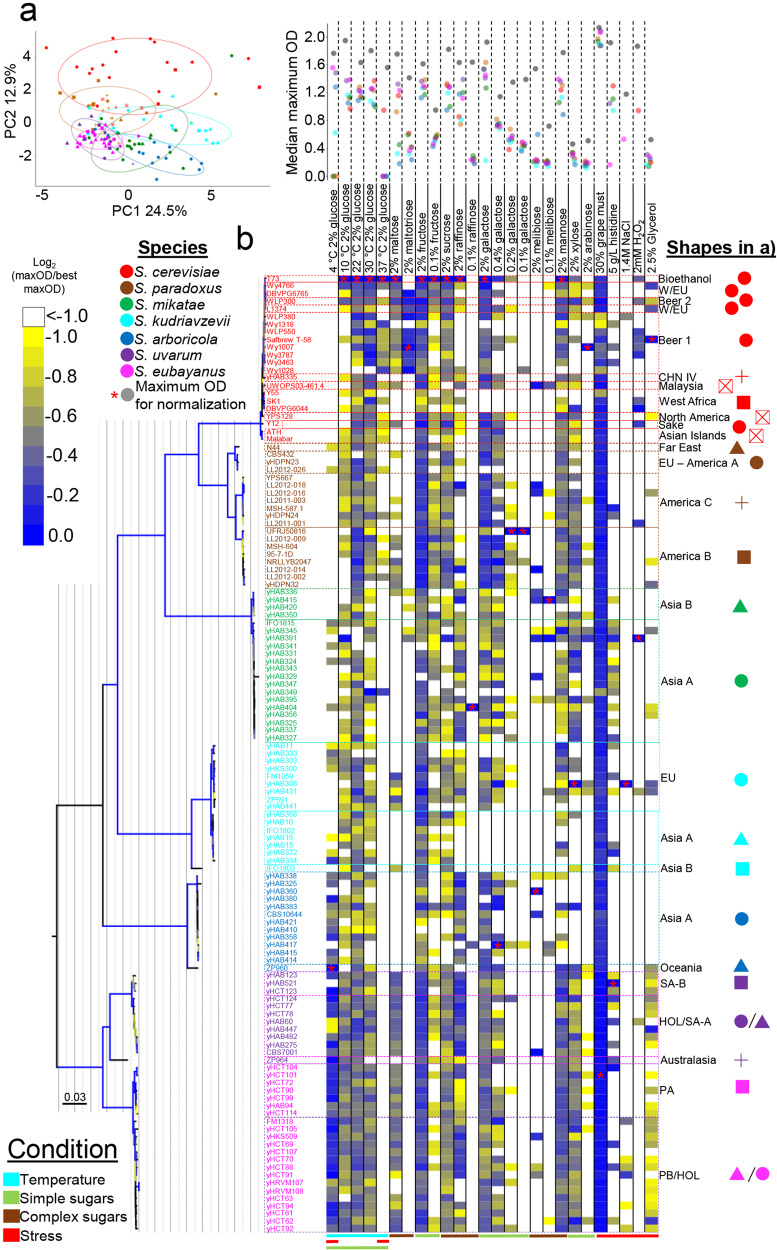
The genus *Saccharomyces* is phenotypically diverse. **a** Principal component analysis (PCA) of PC1 and PC2 of the maximum OD_600_ (maxOD) calculated from growth curves (*n* = 3) from an array of twenty-six media conditions (Supplementary Data 6). PC1 and PC2 accounted for 37.4% of the total variation. A higher image resolution PCA with growth condition weights can be found in Supplementary Fig. 24a. The variation explained by each component is shown in Supplementary Fig. 24b, and a plot of PC1 and PC3 is shown in Supplementary Fig. 24c. Points are colored according to their species designations. Shapes correspond to groups (described in Fig. 6 legend) according to the legend on the right of panel **b**. **b** Heatmap showing the maximum OD_600_, normalized by the highest value for each growth condition as indicated by a red asterisk. Heat colors from yellow (low growth) to blue (high growth) are scaled according to the bar at the left. White colors indicate log_2_ values lower than -1 or no detected growth. Growth conditions are columns, and strains (*n* = 126 *Saccharomyces* strains, two strains were removed because they are rho^0^) are rows. The dot plot above the growth conditions shows the maximum OD_600_ value used for normalizing the data for each growth condition (gray dot), and the colored dots are the median maximum OD_600_ value for each *Saccharomyces* species. A maximum-likelihood (ML) phylogenetic tree of 14 orthologs (~8.7 Kbp) for the phenotyped strains is shown to the left of the heatmap (see Methods for details about the selection of these 14 ortholog genes). Branches are colored according to their bootstrap support (minimum, yellow; maximum, dark blue; no bootstrap value, black). Strain names are colored according to species designations. Population designations are written to the right of the heatmap, and the shapes legend used on panel **a** is shown there. The colored bars below highlight the conditions tested: temperature, simple or complex sugars, and stress. CHN China, EU Europe, HOL/SA-A Holarctic/South America A, PA Patagonia A, PB/HOL Patagonia B/Holarctic, SA-B South America B. iTOL tree at http://bit.ly/2VthpGT.

To explore phenotypic variation across the genus *Saccharomyces*, we phenotyped 128 of the sequenced *Saccharomyces* strains, focusing on phylogenetically distinct lineages from different species (Supplementary Data 2, 6 and Supplementary Fig. 9). We tested the ability of these strains to grow in different carbon sources, temperatures, and stresses (Supplementary Note 2). Growth characteristics varied among *Saccharomyces* species depending on the conditions tested (Supplementary Figs. 18–22). Interestingly, *S. mikatae* had some of the lowest genetic diversity values but had some of the highest phenotypic diversity (Figs. 3b, 5a and Supplementary Fig. 23). Despite some positive correlation between the presence of admixed strains and phenotypic variance, this association was not significant (Supplementary Fig. 23c). *S. eubayanus* and *S. uvarum* strains were mostly overlapping in a principal component analysis (PCA) and were less phenotypically diverse than the other species (Fig. 5a and Supplementary Fig. 23), indicating strains from these sister species have similar traits in the conditions tested (Fig. 5a and Supplementary Fig. 24a, c). These results highlight how phenotypically diverse the *Saccharomyces* genus is and offer new bioresources for industrial applications.

Temperature tolerance was an important condition (Supplementary Figs. 25, 26) for species differentiation (Fig. 5a). *Saccharomyces eubayanus* and *S. uvarum* grew the best at lower temperatures (Fig. 5b and Supplementary Figs. 18, 26c–e), while *S. cerevisiae* and *S. paradoxus* grew the worst at lower temperatures and instead grew best at higher temperatures (Fig. 5b and Supplementary Fig. 26c–e). *Saccharomyces mikatae*, *S. arboricola*, and *S. kudriavzevii* also grew well at lower temperatures, which supports the hypothesis that lower temperature growth is an ancestral trait of the genus *Saccharomyces*^53,54^ and might influence the ecological and geographic distribution of *Saccharomyces* lineages.

**Fig. 6:**
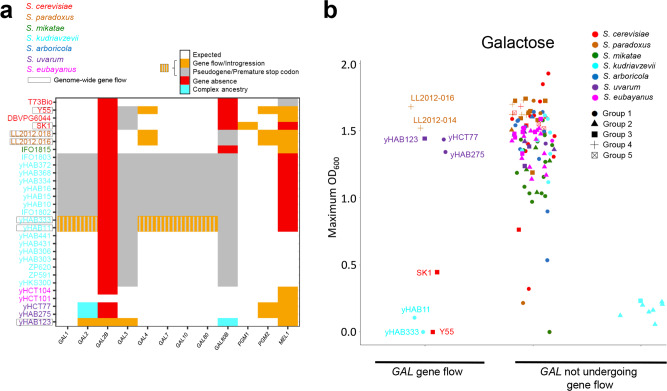
Phenotypic diversity and complex ancestries. **a** *Saccharomyces* strains affected by gene flow for the *GAL* regulon genes. Names of strains with genome-wide admixture (Supplementary Data 3) are boxed. Strain names are colored according to species designations. Complete genes with a phylogenetic position (Supplementary Fig. 28) as expected based on population genomic analysis (Supplementary Fig. 9) are labeled as white. Genes acquired from another lineage by gene flow are labeled orange. Genes with premature stop codons or in a more advanced state of pseudogenization are labeled gray. Genes with complex ancestries, such as unexpectedly ancient alleles, are labeled cyan. Genes not detected by any of the methods employed in this study (see Methods) were considered absent and are labeled red. **b)** Maximum biomass production (OD_600_) on 2 % galactose for *Saccharomyces* strains (*n* = 125, Supplementary Data 6). Each point is a strain colored by species designation. Data were split based on whether (left) or not (right) gene flow had occurred. Asia A and B *S. kudriavzevii* (on the right) were separated from the rest of *Saccharomyces* data points for clarity. The groups are defined as follows: (i) *S. cerevisiae*: Group 1 (Domesticated strains: Bioethanol, Beer 1 & 2, Wine/European, and Sake populations), Group 3 (West African population), Group 4 (CHN IV population), Group 5 (Asian Islands, Malaysian, and North American populations). (ii) *S. paradoxus*: Group 1 (European-America A population), Group 2 (Far East population), Group 3 (America B population), Group 4 (America C population). (iii) *S*. *mikatae*: Group 1 (Asia A population), Group 2 (Asia B population). (iv) *S*. *kudriavzevii*: Group 1 (EU population), Group 2 (Asia A population), Group 3 (Asia B population). (v) *S*. *arboricola*: Group 1 (Asia A population), Group 2 (Oceania population). (vi) *S*. *uvarum*: Group 1 (Holarctic population), Group 2 (South America A population), Group 3 (South America B population), Group 4 (Australasia population). (vii) *S*. *eubayanus*: Group 1 (Holarctic population), Group 2 (Patagonia B population), Group 3 (Patagonia A population).

The utilization pathway for the sugars *GAL*actose and *MEL*ibiose is well studied and highly variable in the genus *Saccharomyces* (Supplementary Fig. 27a)^55–58^. Making use of our diverse genomic and phenotypic dataset, we explored the ancestries of the individual genes involved in the *GAL*/*MEL* pathway (Supplementary Fig. 28) to determine potential genetic bases of variabilities in growth on galactose and melibiose (Fig. 6a, b and Supplementary Fig. 27b, c). Previous studies have observed loss-of-function mutations in some genes of the pathway in *S. cerevisiae*^58,59^, ancient pseudogenization of the entire *GAL* pathway in the *S. kudriavzevii* Asia A and B populations and retention of a functional pathway in the EU population^60,61^, and ancient alleles in some *S. cerevisiae* strains whose origin predates the diversification of the genus^62–65^. Our analyses here found an additional variation that suggests that some of the variations in galactose or melibiose growth were the consequence of gene flow between populations of the same species or introgression between species (Fig. 6a and Supplementary Figs. 27b, 28). For example, two strains of *S. paradoxus* from America C with evidence of gene flow from America B population (Supplementary Fig. 9g) were capable of growing on melibiose, likely because they acquired an active *MEL1* gene from the America B population (Supplementary Figs. 27c, 28h). Introgressions for genes conferring melibiose utilization were also detected between *S. cerevisiae* and *S. paradoxus*^57,59^.

The two new admixed strains of *S. kudriavzevii* provided an even more striking example of gene flow and selection. We previously inferred long-term balancing selection based on local selection regimes for the functional genes and inactivated pseudogenes of *S. kudriavzevii*^60^, but the populations with inactive (Asia A and B) or active (EU) *GAL* networks were strongly differentiated by geography and population structure. Here we discovered two strains isolated from Southern China (Supplementary Fig. 1d and Supplementary Table 2) that shared more than 87% genome ancestry with EU strains (Supplementary Fig. 10h) and yet were unable to grow on galactose (Fig. 6b). Phylogenetic analyses demonstrated that the loss of this trait was due to the acquisition of six *GAL* pseudogenes (at four loci: *GAL1/GAL10/GAL7*, *GAL4*, *GAL2*, and *GAL80*) from the *S. kudriavzevii* Asia A population after the diversification of EU and Asia A populations (Supplementary Fig. 28k). Since these two strains shared less than 12% genome ancestry with the Asia A lineage, in the absence of selection against hybrid networks or against *GAL* activity in Asia, the odds are quite low (*p* = 0.12^4^ = 0.0002) that these closely related strains would have acquired pseudogenes by chance at all 4 *GAL* loci that are functional in the EU population. Notably, the only two *GAL* loci not transferred from Asia A lineage by gene flow into the ancestors of these two strains were *GAL3* and *GAL80B* (Supplementary Figs. 28k, 10h), two pseudogenes that were inactivated in the ancestor of all known strains of *S. kudriavzevii*^60^.

The data also suggested that intricate selection dynamics may be occurring at the *GAL2* locus that are not simply qualitative. Most *S. eubayanus* and *S. uvarum* strains have a tandem duplication at the *GAL2* locus whose function is unknown^17,60–62^. Some *S. cerevisiae* strains from the CHNIII lineage that were isolated from milk fermentations also possess additional copies of *GAL2* whose origin predates the diversification of the genus; these strains lack functional copies of *HXT6* and *HXT7*, which encode hexose transporters, and seem to use *GAL2* to encode the transport of both galactose and glucose in dairy environments that are rich in lactose^65^. Some *S. eubayanus* and *S. uvarum* strains have lost the *GAL2B* gene. Despite testing several growth conditions, including various galactose concentrations, the *S. uvarum* and *S. eubayanus* strains lacking *GAL2B* only displayed maximum growth rate differences at 30 °C on 2% glucose, which was lower (Wilcoxon rank-sum test, *p* value = 5.97 × 10^−4^, Supplementary Fig. 26f). This result suggests a similar model for the evolution of the *S. uvarum*/*S. eubayanus GAL2B* gene and the additional copies of *GAL2* in *S. cerevisiae*, wherein these additional copies of *GAL2* evolved to support glucose transport in specific ecological conditions. Notably, the single copies of *GAL2* from *S. eubayanus* Holarctic strains are an outgroup to the entire *S. uvarum/S. eubayanus* clade, including all known *GAL2* and *GAL2B* alleles (Supplementary Fig. 28b), suggesting that multiple ancient alleles are segregating at this locus due to balancing selection^62^. Collectively, these results highlight how local selection regimes can maintain ancient polymorphisms, even in multi-locus gene networks.

## Discussion

### *Saccharomyces* diversification within and outside of Asia in association with plants

Several authors have postulated Asia as the geographical origin of *S. cerevisiae* and other species of *Saccharomyces*^13,22,25,28,37,66,67^. Our present results provide evidence to support several rounds of speciation in Asia, as well as potentially the origin of the genus itself: (i) the high genomic diversity in the Palearctic biogeographic realm, which includes Asia; (ii) the centrality of Palearctic mitochondrial haplotypes to the mitochondrial network; (iii) and ancestral polymorphisms in Asian strains that generate phylogenetic conflict and, in some cases, such as the *GAL* loci, phenotypic differences that are likely under strong selection. The presence of ancestral polymorphisms in several populations and species suggests that *Saccharomyces* diversification was rapid^68^, that considerable gene flow continued prior to the generation of strong species barriers^69–73^, or both. The presence of all species in association with trees of the order Fagales points to the adaptation of the last common ancestor of *Saccharomyces* to these hosts. However, there is still much to learn about the ecological distribution of yeasts in general, and *Saccharomyces* in particular^74^, where sampling has often been biased toward bark and soil samples from Fagales. Even though most new lineages and species likely originated in Asia, our comprehensive global sampling and analyses strongly support the hypothesis that several lineages originated in South America, North America, Europe, and Oceania, including lineages of *S. eubayanus*, *S. paradoxus*, *S. uvarum*, *S. jurei*, and *S. arboricola*^14,21,24,26,27,31,75–77^ (Fig. 4c). These diversifications could be accompanied by the adaptation to new hosts. For example, *S. uvarum* and *S. eubayanus* lineages are frequently isolated from fungi associated with trees of the genus *Nothafagus* in South America. This influence of related *Nothafagus* hosts during diversification might help explain the similar phenotypic traits observed among *S. uvarum* and *S. eubayanus* strains.

The ecological and genetic factors driving this diversification of the genus could also be linked to temperature fluctuations during the Miocene epoch, which is coincident with *Saccharomyces* divergence times^30^. Temperature fluctuations have played an important role in the diversification of plants^78^ and animals^79^, and temperature tolerance differentiates several *Saccharomyces* species and clades. In particular, the high-temperature tolerance of *S. cerevisiae* and *S. paradoxus*^53,54,80^ seems to be a derived trait. The influence of temperature during diversification might be one of the reasons why we observe frequent introgressions in the mitochondrial genome^45–48^, where species-specific mitotypes have been shown to strongly affect temperature tolerance^52,81^. Clear patterns of differentiation by geographic distribution and climatic conditions have also been detected for *Saccharomyces* mitotypes^26,33,67,82,83^.

The role of introgressions during lineage diversification is still under debate, but nuclear introgressions between species have been mainly observed in human-associated environments, including the horizontal gene transfer of few genes^84–86^, frequent admixture of domesticated *S. cerevisiae* strains^13,36,64^, and interspecies hybridization of strains used to produce fermented beverages^87–89^. In contrast, cytoplasmic genetic elements have undergone extensive introgression and gene flow, even in wild strains of *Saccharomyces*, as previously seen in animals^90–92^. Increasing the number of strains with complete genomes sequences, improving their assembly qualities to telomere-telomere levels, together with the complete genome annotation of all genetic elements, would facilitate a more systematic analysis of regions undergoing introgression and gene flow, including their potential roles in adaptation and diversification.

### *Saccharomyces* populations are often more genetically differentiated than multicellular eukaryotic species

Multicellular eukaryotes might be more permeable to interspecies introgression^93,94^ because animal and plant species are more closely related than species are in the genus *Saccharomyces*. The distinction is not entirely due to differences in taxonomic practice because, even when we considered phylogenetically distinct *Saccharomyces* lineages, only 27.16% of *Saccharomyces* nuclear genomes were admixed, and most of them were from anthropic environments. Spore viabilities lower than 1%^71,95^ in crosses between strains have been considered sufficient to define yeast species using the biological species concept alone. When combined with phylogenetic and ecological species concepts, taxonomic authorities have accepted spore viabilities lower than 10%, as seen for *S. eubayanus* and *S. uvarum*, which have the highest AAI values among currently recognized species^96,97^.

Our comparison of AAI values with multicellular eukaryotes suggests that species designations based on spore viability and other currently used criteria do not differentiate *Saccharomyces* species as finely as the criteria deployed by plant and animal taxonomists. If they did, what we currently consider *Saccharomyces* populations or lineages might be more analogous to the species designations of multicellular eukaryotes. Even so, current yeast taxonomic practice has the advantage of recognizing the ease with which genes of phenotypic importance flow between populations of the same species.

### Phenotypic diversity through complex ancestries

Phenotypic traits are gained and lost frequently in animals, plants, and fungi^30,98–100^. Alternatively, traits can be retained in a species by balancing selection when different lineages or populations maintain genes or even multi-locus gene networks encoding traits due to local adaptation or fluctuating conditions. For example, here we showed that some admixed *S. paradoxus* America C strains regained the ability to grow in melibiose by acquiring a functional *MEL1* gene from the *S. paradoxus* America B population. Even more strikingly, two admixed *S. kudriavzevii* strains, which were isolated in Asia but were more closely related to the EU population, lost the ability to grow in the presence of galactose by acquiring *GAL* pseudogenes from the Asia A population, directly demonstrating gene flow between Gal^+^ and Gal^-^ populations of *S. kudriavzevii*^60^. Recent studies concluded that *S. cerevisiae* maintained alternative higher-activity versions of the *GAL* network due to segregating variation at multiple loci^62^. Our results definitively show that qualitative variation can also segregate within a species for a multi-locus gene network, and indeed, suggest that pseudogenized genes may be preferred in some environments. We conclude that the maintenance of compatible alternative versions of gene networks, even at unlinked loci, may be more frequent than previously thought.

In conclusion. the model genus *Saccharomyces* and the current dataset provide an important quantitative benchmark of the boundaries of lineages, populations, and species in terms of genetic variation, phenotypic variation, and the relationship between genotype and phenotype. Setting these boundaries helps characterize eukaryotic microbial biodiversity, improves understanding of ecological dynamics, and offers bioresources of industrial interest.

## Methods

### Yeast strains and maintenance

Strains with codes FM[Number] (i.e., FM1198) or yHXX[Number] (i.e., yHAB33) are physically present and may be requested from cthittinger@wisc.edu (Supplementary Data 1). Strains that are also available from the Portuguese Yeast Culture Collection (PYCC) are indicated with PYCC accession numbers in Supplementary Data 1; most were deposited as part of a previous study by ref. ^32^. For the rest of the strains, references are provided in Supplementary Data 1 to request them from the corresponding lab. Yeast strains are stored in cryotubes with YPD (1% yeast extract, 2% peptone, and 2% glucose) and 15% glycerol at −80 °C. Routine cultures were maintained in YPD plus 2% agar plates at 24 °C. The taxonomic order of hosts was retrieved for each species based on the “Host” column of Supplementary Data 1 using the R package taxize v0.9.99^101^.

### *COX2* and *COX3* PCR amplification, sequencing, and analyses

Partial gene sequences were obtained for *COX2* (471 bp) and *COX3* (491 bp) mitochondrial genes using primers and conditions described by ref. ^32^. *COX2* is a highly polymorphic marker, which is useful to trace ancient hybridization events^32^, due to genetic footprints left by a free-standing homing endonuclease inserted into its coding sequence^48,52^. In contrast, *COX3* is less affected by homing activity than *COX2*, providing a better picture of mitochondrial inheritance^32^. Genomic DNA (gDNA) was isolated following the phenol:chloroform procedure^102^. Gene sequences were determined by PCR and Sanger sequencing. Sequences were edited and assembled with STADEN Package version 1.7^103^. *COX2* and *COX3* sequences were deposited in GenBank under accession nos. MH813536-MH813939.

*COX2* and *COX3* sequences of *Saccharomyces* strains whose whole genomes were sequenced (Supplementary Data 2) were retrieved from genome assemblies using a local BLAST v2.6^104^ or from raw Illumina reads by using HybPiper v1.2^105^. New sequences were manually added to previously aligned gene sequences^48^. 1772 *Saccharomyces COX2* sequences, and 996 *COX3* sequences in FASTA format were classified by haplotype using DnaSP v5^106^ and converted to NEXUS format. The NEXUS file, with haplotype and biogeographic realm frequency information (Supplementary Data 1), was used as input for PopART v1.7 (http://popart.otago.ac.nz) to reconstruct a phylogenetic network. The relationship among haplotypes was inferred by using the Templeton, Crandall, and Sing (TCS) method^107^ (Fig. 2a and Supplementary Fig. 3).

### Paired-end and mate-pair Illumina library preparation

Representative strains of diverse *Saccharomyces* lineages were selected to prepare 2 × 300 bp or 2 × 250 bp paired-end and 2 × 100 bp mate-pair Illumina libraries (Supplementary Data 2). To explore intrapopulation diversity across the genus (Fig. 3a, b), additional Illumina paired-end libraries for 65 *Saccharomyces* strains from different species were made, and sequencing lengths were between 100 to 300 bp.

#### Paired-end Illumina libraries

Paired-end Illumina libraries were prepared as previously described in ref. ^102^. Libraries were sequenced using Illumina HiSeq 2000, HiSeq 2500 Rapid, or MiSeq. The quality and quantity of the finished libraries were assessed using an Agilent DNA1000 series chip assay (Agilent Technologies) and Invitrogen Qubit HS Kit (Invitrogen, Carlsbad, CA), respectively, and the libraries were standardized to 2 nM. Images were analyzed using CASAVA version 1.8.2.

#### Mate-pair Illumina libraries

Genomic DNA was isolated following the phenol:chloroform procedure^102^. gDNA was quantified with a Qubit HS Kit (Invitrogen, Carlsbad, CA), and 4 µg were used to perform the Gel-plus protocol of the Nextera Mate Pair Library Prep Kit (Illumina, San Diego, CA). The target size selection was 8 kbp (Ty elements are around 6 kbp^108^), and fragments were isolated from the gel with a QIAEX II Gel Extraction Kit (QUIAGEN, Germantown, MD). Fragments were sonicated in a Covaris instrument using Covaris tubes (Covaris, Woburn, Massachusetts), and 300–400 bp fragments were targeted. gDNA cleanups were performed using Axygen Mag PCR Cleanup beads (Axygen, Union City, CA). The quality and quantity of the sonicated fragments and finished libraries were assessed using an Agilent DNA1000 series chip assay (Agilent Technologies, Santa Clara, CA) and Invitrogen Qubit HS Kit (Invitrogen, Carlsbad, CA), respectively. The libraries were standardized to 2 nM. Sequencing images were analyzed using CASAVA version 1.8.2.

### Quality filtering, genome assembly, and annotations

Reads were demultiplexed, and Illumina adapters were removed using Trimmomatic v0.33^109^ with parameters 2:30:10 TRAILING:3 MINLEN:[20 for reads shorter than 101 bp or 25 for larger reads] and NextClip v1.3.1^110^, respectively. A quick phylogenetic assessment was performed with an Alignment and Assembly Free (AAF) method v20150930^111^ to check that the correct strain was sequenced. Briefly, AAF reconstructed a phylogenetic tree using Illumina reads as an input, and the phylogenetic position of the sequenced strains was assessed.

Trimmed reads were assembled using the meta-assembler pipeline iWGS v1.1^112^. Briefly, the wrapper performs quality-based read trimming, follow by *k*-mer length optimization, and uses multiple state-of-the-art assemblers to generate genome sequence assemblies. The quality of the assemblies was assessed using QUAST v3.2^113^ as implemented in the wrapper, and the best assembly was chosen based on the number of contigs/scaffolds and N50 statistic (Supplementary Data 2). For the collection of 22 representative *Saccharomyces* strains and the *Saccharomyces eubayanus* CDFM21L.1 strain, additional steps were taken to decrease the number of scaffolds and generate a nearly complete genome assembly. Scaffolds longer than 10 kbp were retained. Ultrascaffolding was manually done in Geneious vR6^114^ by using synteny information generated by MUMmer v3.23 with parameters --maxgap=500 --mincluster=150. This process was used to order scaffolds by comparing them to previously assembled high-quality genomes and to the best newly assembled genomes. Concatenated scaffolds were separated by manual addition of 10,000 Ns. Error correction of the ultra scaffolded assembly was performed with Pilon v1.22^115^. These corrected assemblies were the final versions used for downstream analyses. Qualimap v2.2.1 ^116^ generated quality statistics for Illumina reads by using the final assemblies as a reference for where reads were mapped (Supplementary Data 2).

Complete mitochondrial genomes were filtered out from our assemblies by screening the assembly scaffolds. Mitochondrial genome scaffolds were of length between 40–90 kbp and GC content lower than 30%. The extrachromosomal 2-µm plasmid scaffolds were filtered out by detecting those scaffolds matching 2-µm plasmid genes and having a length lower than 7 kbp. We calculated the nuclear (Supplementary Fig. 6b) and mitochondrial GC content and length (Supplementary Fig. 6a, c) using the infoseq with flags --auto –only –name –length -pgc from the EMBOSS package v6.5.7^117^. The previously sequenced and assembled *S. jurei* mitochondrial genome was corrected to remove an artifactual duplicated region produced by the original PacBio assembly pipeline^18^; this correction reduced the previously described length from ~111 kbp to 79 kbp. The completeness of the nuclear genomes was quickly assessed by exploring the number of single-copy orthologous genes annotated by BUSCO v2.0.1^118^ using the saccharomycetales_odb9 database (Supplementary Fig. 30).

Genome annotations of our nuclear assemblies were performed with YGAP v7^119^. To minimize downstream analysis errors, we also re-annotated all previously published genome assemblies using YGAP. YGAP output was in GenBank format, which was converted to gff3 format in Geneious. Paralogous genes are not fully resolved by YGAP, so synteny and manual inspection was used to resolve the genomic location of each paralog. Mitochondrial genomes were annotated with MFannot v1^120^. 2-µm plasmids were manually annotated in Geneious. Genome assemblies and annotations are available in GitHub (http://bit.ly/2orfKyT) and ENA accession no. PRJEB48264.

### Phenotyping strains

We phenotyped the 87 strains whose genomes we sequenced, as well as 41 *Saccharomyces* strains whose genomes had been previously sequenced, in 26 media conditions (Supplementary Data 1, 6). We tested carbon sources (2.5% glycerol; 2% glucose; 2% maltose; 2% maltotriose; 2% and 0.1% fructose; 2% sucrose; 2% and 0.1% raffinose; 2%, 0.4%, 0.2%, and 0.1% galactose; 2% and 0.1% melibiose; 2% mannose; 2% xylose; and 2% arabinose) and stresses, including osmotic (30% must juice, 1.4 M NaCl), pH 7.5 (5 g/L histidine), oxidative (2 mM H_2_O_2_), low temperatures (4 and 10 °C), and high temperatures (30 and 37 °C). All conditions, except low temperatures and high temperatures, were performed at 22 °C. The medium composition was minimal medium (6.7 g/L Yeast Nitrogen Base without amino acids, carbohydrates, and with Ammonium Sulfate, pH 5.0) supplemented with 2% glucose when no other carbon sources or different concentrations are specified. Must or grape juice was prepared according to Clowers^121^, except 30% was our final concentration. Glucose and fructose concentrations of the must were measured by high-performance liquid chromatography (HPLC) following the procedure described in ref. ^32^. 14% fructose and 12% glucose were detected in the 30% must.

The 128 *Saccharomyces* strains were pre-cultured in deep 96-well plates with 500 µl of minimal medium supplemented with 0.2% glucose until saturation at room temperature. After pre-culturing, we used a pinner to inoculate 96-well plates (Nunc, Roskilde, Denmark) containing 240 µl of all of the media being tested. Initial optical densities at 600 nm (OD_600_) were below 0.2 (mean 0.037 ± 0.027). These 96-well plates were designed with corner containers, which we filled with 3 mL of dH_2_O to maintain humidity during culturing. As a control, each plate contained five wells with YPD medium (1% yeast extract, 2% peptone, and 2% glucose) where the *S. cerevisiae* S288C strain was inoculated. To monitor the growth of strains in the different conditions, the inoculated 96-well plates were placed in a stacker of a BMG FLUOstar Omega plate reader (BMG Labtech, Ortenberg, Germany) located inside an incubator with an interior temperature set to 22 °C. Absorbance at 600 nm was monitored every 2 h for 6–8 days with no shaking. The absorbance of low-temperature and high-temperature experiments were manually monitored (3–4 data points per day) in a BMG FLUOstar Omega, and the experiments were stopped when saturation of growing strains was detected. Strain location in the 96-well plates was randomized in each replicate. Two ρ^-^ strains, *S. eubayanus* yHCT96^122^ and Bond Lab 1063 (this study), were removed from the phenotyping analysis. Background absorbance was subtracted from the average of five negative controls (uninoculated media). Kinetic parameters for each condition were calculated using GCAT v6.3^123^. The average, median, and standard deviations of kinetic parameters from the three independent biological replicates were calculated in R v4.0.2 ^124^ (Supplementary Data 6).

Flocculation can generate artificially low or high OD values, depending on which part of the well is measured by the spectrophotometer. To correct for flocculation, we took pictures of the first replicate of each 96-well plate and manually inspected the kinetic parameters to correct for false positive and negative values. In cases where the pictures displayed no growth but there were high OD values (e.g., due to condensation on the lid), we set the growth of that particular strain to 0. In cases where growth was not detected but exaggerated OD values were observed and the pictures showed evidence for flocculation, we removed the exaggerated values and kept the parameter values from other runs close to those observed on closely related strains.

Boxplots for kinetic parameters by species (Supplementary Fig. 26) and by groups (Supplementary Fig. 29) were drawn using ggplot2 v3.3.3^125^ and gridExtra v2.3 packages in R. The stacked bar plots showing the percentages of strains that grew above an OD_600_ of 0.5 normalized by species (Supplementary Fig. 18) were drawn with ggplot2. Dot plots of the median maximum OD_600_ by species (Fig. 5b top) were drawn with ggplot2. The variance of the median maximum OD by growth condition for each species (Supplementary Fig. 23a) was calculated in R and plotted with ggplot2. The correlations of mean Tamura-Nei corrected genetic distance (see *Species tree, genetic boundaries among species, and concordance factors* section) within species and the average phenotypic variance or the percentage of admixed strains for each species were tested using the Spearman correlation test implemented in ggscatter function of the R package ggpubr v0.4^126^, and the scatterplots (Supplementary Fig. 23b, c, respectively) were drawn with ggplot2. The heatmap with median maximum OD_600_ values normalized to the most extreme OD_600_ value for each condition (Fig. 5b) was drawn, together with the phylogenetic tree (see *Phylogenomics of nuclear, mitochondrial, and 2-µm plasmid genomes of phenotyped strains* section), using iTOL v4.2.3^127^. A principal component analysis (PCA) was performed by prcomp function in R using the median maximum OD_600_ calculated from replicated growth curves. Selected PCs were plotted (Fig. 5a and Supplementary Fig. 24a, c) with ggbiplot v0.55^128^ package in R. The percentage of variance explained by each component (Supplementary Fig. 24b) was plotted with factoextra v1.0.7^129^ package in R. The percentage of variance explained by each growth condition for each component was calculated in R, and the histogram plot (Supplementary Fig. 25) was drawn with ggplot2.

### Read mapping and variant calling

Illumina reads from 163 *Saccharomyces* strains were generated in this study or downloaded (Supplementary Data 2). We mapped Illumina reads to a reference genome belonging to one of the *Saccharomyces* species, following our previously developed pipeline^24^. The *Saccharomyces cerevisiae* reference genome was DBVPG6044^12^; for *Saccharomyces paradoxus*, it was CBS432^12^; for *Saccharomyces mikatae*, IFO1815 (this study); for *Saccharomyces kudriavzevii*, IFO1802 (this study); for *Saccharomyces arboricola*, CBS10644^16^; for *S. uvarum*, CBS7001 (this study); and for *S. eubayanus*, FM1318^17^. Reference genomes consisted of the assigned chromosomes, and extra scaffolds were discarded for mapping.

Illumina reads were first mapped with bwa v0.7.12 using the bwa-mem algorithm^130^. The resulting SAM files were viewed and sorted using samtools v1.4^131^, filtering for high-quality reads with samtools view –q 30 (except for *S. kudriavzevii*, *S. arboricola*, and *S. eubayanus* where we set the quality to 20). PCR duplicates were removed with picard v1.98 (http://picard.sourceforge.net/) using MarkDuplicates.jar REMOVE_DUPLICATES=true AS=true VALIDATION_STRINGENCY=SILENT. Read groups were set using picard v1.98 AddOrReplaceReadGroups.jar with settings “VALIDATION_STRINGENCY=SILENT SORT_ORDER=coordinate CREATE_INDEX=true”. Single nucleotide polymorphisms (SNPs) were called using the GATK v3.1^132^ haplotype caller using the setting --genotyping_mode DISCOVERY -mbq 20 -stand_emit_conf 31 -stand_call_conf 31. Genome coverage was measured using BEDTOOLS v2.27.0 genomeCoverageBed -d –ibam^133^. The VCF output of GATK was converted into FASTA format using a custom python script. A specific FASTA file for each strain, now called its Whole Genome Sequence (WGS), was obtained by using the reference genome as a template and replacing the called variant with the SNP reported by GATK. The presence of heterozygous sites in the sample were coded according to their IUPAC ambiguity codes. In downstream analyses, we considered only the homozygous SNPs, which represented the vast majority of the genome (>99.4%) due to the low levels of heterozygosity (Supplementary Fig. 15). Insertions and deletions were masked by replacing the genomic sequence with a number of Ns corresponding to the length of the indel called. FASTA files for each sample were generated by masking regions with extremely high coverage (i.e., values greater than the 99.9th percentile of genome-wide coverage) and by masking regions with low coverage (i.e. either regions below 10X coverage or, for genomes with low coverage, below the 10th percentile of genome-wide coverage). Masked regions were replaced by Ns prior to downstream analyses.

### Population genomics and quantification of reticulate evolution

WGSs were aligned by species. Frequent gaps were removed using trimal v1.4.1 ^134^ with parameters –gt 0.9. We calculated the average distance within species and populations with MEGA v7^135^ using complete gap removal and Tamura-Nei correction (Fig. 3b). Polymorphism statistics from the WGS dataset were calculated in DnaSP v5 ^106^, and a stacked bar plot (Fig. 3a) with those results was drawn with ggplot2.

To delimit the number of populations in each *Saccharomyces* species, we used the program STRUCTURE v2.3.4^136–138^ (Supplementary Fig. 9i) and fineSTRUCTURE v2.0.7^139^ (Supplementary Fig. 9iv-v). VCF files were merged with GATK using the CombineVariants parameter and -genotypeMergeOptions UNIQUIFY. 10,000 random SNPs from the VCF data were picked for STRUCTURE analysis. We tested K clusters from 1 to 8 (except for *S. cerevisiae* where more clusters were tested), assuming an admixture model, with a 10,000-iteration burn-in and 100,000 iterations of sampling. Five independent runs were performed for each K cluster. The STRUCTURE output was used as input for STRUCTURE HARVESTER web v0.6.94^140^ to select the most likely number of populations. STRUCTURE HARVESTER output files were aligned in CLUMPP v1.1.2^141^ and visualized in STRUCTURE PLOT v2^142^. In addition to delimiting the likely number of populations, fineSTRUCTURE gave a deeper picture of co-ancestry among *Saccharomyces* strains (Supplementary Fig. 9iv). We converted the FASTA dataset with the complete set of SNPs to a PHASED format, the input format of fineSTRUCTURE. To reconstruct the co-ancestry heatmap with the linkage model and to perform a principal component analysis (Supplementary Fig. 9v) of the SNP dataset, fineSTRUCTURE was run with default parameters, except ¨-ploidy 1” due to the low heterozygosity in the dataset; the genetic distance map was inferred by applying the specific genetic distance for each chromosome described on the SGD database.

We reconstructed the phylogenetic tree and network using the SNP dataset for each species (Supplementary Fig. 9ii and iii, respectively). ML phylogenetic tree reconstruction was done in RAxML as above, after correcting branch lengths for the presence of invariant sites. The SNP dataset was also the input of SplitsTree, which we used to detect incongruent data. For *S. arboricola, S. eubayanus*, and *S. kudriavzevii* species datasets, the outgroup was CBS7001. For *S. cerevisiae*, the outgroup was CBS432; for *S. paradoxus*, the outgroup was S288C; for *S. mikatae*, the outgroup was NCYC3947; and for *S. uvarum*, the outgroup was FM1318.

Supported admixture strains were further analyzed to quantify the genome contributions of parental strain relatives. The WGS alignment dataset was split into alignments consisting of 50,000 bp to calculate genetic contributions (Supplementary Fig. 10) using PopGenome v2.2.4 package in R^143^. For detecting the ancestry of *GAL* genes in two Chinese *S. kudriavzevii* strains and the two closest relatives (Supplementary Fig. 10h i), the WGS alignment dataset was split consisting of 5,000 bp to calculate genetic contributions using PopGenome, and a log_2_ divergence ratio was plotted (Supplementary Fig. 10h ii). *GAL* gene coordinates were used to locate the coding sequences in the plot. Quantification of genome introgression between species was performed by analyzing sppIDer v1^144^ plots (Supplementary Data 3 and Supplementary Fig. 16).

### Phylogenomics of nuclear, mitochondrial, and 2-µm plasmid genomes of phenotyped strains

To build a phylogenetic tree of the nuclear genomes (Fig. 5b) for the phenotyped 128 *Saccharomyces* strains, we searched for a common set of complete orthologous gene sequences. First, we annotated single-copy orthologous genes of our phenotyped collection of *Saccharomyces* strains with BUSCO. We detected 18 orthologs common to all genome assemblies of phenotyped strains, but we selected 14 well-resolved gene trees: *ASF1* (*YJL115W*), *MAF1* (*YDR005C*), *NIF3* (*YGL221C*), *NSL1* (*YPL233W*), *PET117* (*YER058W*), *PLP2* (*YOR281C*), *QCR7* (*YDR529C*), *RPA34* (*YJL148W*), *SGF73* (*YGL066W*), *SHB17* (*YKR043C*), *SMT3* (*YDR510W*), *SUB1* (*YMR039C*), *TUB2* (*YFL037W*), and *YAR1* (*YPL239W*). We blasted those genes to pull out the orthologs from the strains that were not phenotyped, as well as *Kluyveromyces lactis* as an outgroup (Supplementary Data 1). Orthologous genes were concatenated with FASconCAT v1.0^145^. A maximum-likelihood (ML) phylogenetic tree for a concatenated alignment (~8.7 Kbp), with frequent gaps trimmed with trimal, was reconstructed in RAxML v8.1^146^, performing 100 iterations to search for the best tree, using the model GTRGAMMA. Bootstrap branch support was assessed by performing 1,000 pseudoreplicates using the same model parameters as above. The ML phylogenetic tree can be accessed together with the phenotypic data of phenotyped strains at iTOL (http://bit.ly/2PYRuUc). The same concatenated alignment was used in SplitsTree 4^147^ to reconstruct the phylonetwork using the NeighborNet (NN) method (Supplementary Fig. 14b). We followed a similar pipeline to reconstruct the mitochondrial and 2-µm plasmid phylogenetic networks, instead using mitochondrial and 2-µm plasmid genes (Fig. 2b and Supplementary Fig. 17, respectively). For the mitochondrial genome, we were focused on the coding sequences (CDS) and genes encoding rRNAs of 73 sequenced *Saccharomyces* strains representing the diverse *Saccharomyces* lineages (Supplementary Data 1): *ATP6*, *ATP8*, *ATP9*, *COB*, *COX1*, *COX2*, *COX3*, 15S rRNA, 21S rRNA, and *VAR1* (~9.7 kbp). Mitochondrial phylogenetic networks for individual gene alignments were also reconstructed (Supplementary Fig. 4). *COX2*, *COX3*, *ATP6, ATP8*, and *ATP*9 included the sequences of all non-ρ^-^ phenotyped *Saccharomyces* strains. For the 2-µm plasmid, we analyzed the frequently observed genes *REP1* (*R0020C*) and *REP2* (*R0040C*) (extrachromosomal tag in Supplementary Fig. 17), which were used to classify the plasmid sequences by class^13,50^ (Supplementary Data 4). It is noteworthy that some plasmid genes were inserted in the nuclear genome (nuclear tag in Supplementary Fig. 17).

### Species tree, genetic boundaries among species, and concordance factors

To infer the phylogenetic relationships of our species and lineages and the degree of genome-wide support, we performed several phylogenomic analyses. We first selected 23 strains to represent key *Saccharomyces* lineages, most of them high-quality genomes generated here, and an additional 15 previously assembled genomes (Supplementary Data 1). Then, the coding sequences and amino acid sequences annotated with YGAP were extracted using Daniel Jeffares’ perl script process_gff_cds_proteins.pl^148^. For each gene, two types of files were generated: one file containing all species’/lineages’ CDS and another file containing all species’/lineages’ protein sequences. Amino acid sequences were aligned using MAFFT v7.21^149^, using the setting “--preservecase --maxiterate 1000 --genafpair”. Amino acid alignments were back-translated to nucleotides using pal2nal v14^150^. Codon columns with gaps were removed from the alignments using trimal “-gt 1 –block 3”^134^. Gene sequences present in all specimens that retained at least 50 % of positions and with equal or more than 300 nucleotides (100 amino acids) were selected for additional analyses. A total of 3850 genes passed our filters. ML phylogenetic trees from each CDS alignment were calculated in IQTree v1.6.12^151^ following recommendations by Shen et al^152^. The next settings were used in IQTree “-bb 1000 -wbt -nt AUTO -seed 225494 -st DNA -m TEST”. The coalescent species tree was generated using the collection of ML phylogenetic trees in ASTRAL v5.7.7^153^. The gene concordance factor (gCF) (Fig. 4a) for each branch in the coalescent species tree (reference tree) was assessed using all individual gene trees as input for IQTree v2.0.3.

To assess the reciprocal monophyly of each gene, we followed the bioinformatic pipeline developed by ref. ^154^. Briefly, ML phylogenetic trees were read in R using treeio v1.12^155^ and converted to ape v5.4 format^156^. Once species designations were associated with phylogenetic tip labels, the trees were rooted in the branch generating the *S. eubayanus* and *S. uvarum* clades. Monophyly tests were performed using spider v1.5^157^, and when the test for one species was FALSE (Supplementary Data 5), the tree was printed to a TIFF file for visual exploration. Trees were drawn using R package ggtree v2.2.4^158^. The monophyly test suggested that 0.77% of genes were incorrectly annotated (e.g., due to cryptic paralogy) in at least one of the 38 genomes.

We reconstructed the concordance tree topology, which was congruent with the ASTRAL coalescent tree, and we inferred the branch support (CF, Fig. 4a) using the BUCKy v1.4.4^159^ pipeline as done previously^122^. Due to memory issues with large datasets in BUCKy, we reduced the number of *Saccharomyces* strains to 10 (highlighted with asterisks in Fig. 4a). Strains were selected based on their Asian origin when possible. We included a *Kluyveromyces lactis* strain as an outgroup (Supplementary Data 1). Before running BUCKy, the gene alignments were the input for MrBayes v3.2.3^160^ for a Bayesian phylogenetic reconstruction. 49 genes failed to be parsed through the pipeline, so 3801 genes were analyzed. Settings in MrBayes were “lset nst=6 rates=gamma; prset brlenspr=Unconstrained:Exp(50.0); mcmc nruns=2 temp=0.2 ngen=110000 burninfrac=0.0909; Nchains=4 samplefreq=10 swapfreq=10 printfreq=50000; mcmcdiagn=yes diagnfreq=50000”. Sample trees from each MCMC run were summarized for each gene with mbsum after a burn-in of 1000 trees. BUCKy was run with all collected mbsum files with settings “-a 1 -k 3 -n 100000 -c 2 --calculate-pairs --create-joint-file --create-single-file”. BUCKy generated the posterior probability that pairs of loci share the same tree, which we represented in a histogram (Supplementary Fig. 13).

Boundaries among *Saccharomyces* species were calculated using the nuclear, mitochondrial, and 2-µm plasmid CDS alignments as input. Distributions, boxplots, and heatmaps of genetic distance (Supplementary Fig. 11) and distributions of relative divergence (Fst) (Supplementary Fig. 12) were calculated in R. Tamura-Nei corrected genetic distance was calculated with dist.dna as implemented in the R package ape v5.4. Fst was calculated by the R package PopGenome v2.7.5 ^143^. Heatmaps were drawn with pheatmap v4.0.5.

### *GAL*/*MEL* pathway characterization

To characterize the *GAL/MEL* pathway from the phenotyped strains, genes were retrieved from (i) the genome assemblies using the YGAP annotation files; (ii) genome assemblies using a local BLAST v2.6 ^104^; (iii) raw Illumina reads by using HybPiper v1.2 ^105^; or (iv) PCR and Sanger sequencing of strains of interest (Supplementary Data 7). ML phylogenetic trees for individual genes from the *GAL*/*MEL* pathway were reconstructed in IQTree, with similar settings as above. Phylogenetic trees were read and manipulated with R packages treeio, ape, and phytools v0.7 ^161^, and drawn using ggtree. Conclusions about gene presence/absence and phylogenetics were displayed in heatmaps (Fig. 6a and Supplementary Fig. 27b) using the R package ggplot2.

To confirm the unexpected absence of the second copy of *GAL2* in some *S. eubayanus* and *S. uvarum* strains, we performed PCR and Sanger sequencing using primers and conditions described in Supplementary Data 7. To optimize PCR conditions, we first performed gradient PCR, and the optimal annealing temperature was selected for amplifying the target region (Supplementary Data 7). For amplifying long regions (expected length >6 kbp, Supplementary Data 7), LongAmp polymerase (New England Biolabs, Ipswich, MA, USA) was used, instead of Taq polymerase (New England Biolabs, Ipswich, MA, USA). Sanger-sequenced *GAL* sequences were deposited in GenBank under accession nos. OL660614-OL660618.

### Amino acid identity comparisons between animals and *Saccharomyces*

To compare the divergence among animals and among *Saccharomyces* species and lineages, we annotated a common set of single-copy orthologous genes with BUSCO v5.1.3 ^162^ using the eukaryota_odb10 database. We first downloaded the genome assemblies for *Homo sapiens* GRCh38p13 (GCA_000001405.28), *Pan troglodytes* ClintPTRv2 (GCA_002880755.3), *Macaca mulata* AG07107 (GCA_003339765.3), *Mus musculus* C57BL6J (GCA_000001635.9), *Takifugu rubripes* fTakRub1 (GCA_901000725.2), and *Gallus gallus domesticus* bGalGal1 (GCF_016699485.2). Then, we ran BUSCO on those genomes and high-quality genomes for *Saccharomyces* strains with no detected admixture or introgressions (Supplementary Data 1). Each organism’s amino acid sequences for each protein were pulled together. Amino acid alignments were performed using MAFFT with similar settings as above. Individual protein alignments were read in R with seqinr v4.2 package ^163^. Amino acid identity (AAI) values (Fig. 3c) for each protein between lineages of the same *Saccharomyces* species, between *Saccharomyces* species, and between the chosen animals were calculated using dist.alignment function implemented in seqinr with the “matrix=identity” setting. Mean AAI values for each comparison was plotted with ggplot2.

### Reporting summary

Further information on research design is available in the Nature Portfolio Reporting Summary linked to this article.

## Supplementary information

Supplementary Information

Description of Additional Supplementary Files

Supplementary Data 1

Supplementary Data 2

Supplementary Data 3

Supplementary Data 4

Supplementary Data 5

Supplementary Data 6

Supplementary Data 7

Reporting Summary

## Data Availability

Strains with codes FM[Number] (i.e., FM1198) or yHXX[Number] (i.e., yHAB33) are physically present and may be requested from cthittinger@wisc.edu (Supplementary Data 1). Strains that are also available from the Portuguese Yeast Culture Collection (PYCC) are indicated with PYCC accession numbers in Supplementary Data 1; most were deposited as part of a previous study by ref. ^32^. For the rest of the strains, references are provided in Supplementary Data 1 to request them from the corresponding lab. The *COX2* and *COX3* sequences generated in this study were deposited in GenBank under accession nos. MH813536-MH813939. The *GAL* genes that were Sanger-sequenced in this study were deposited in GenBank under accession nos. OL660614-OL660618. Illumina sequencing data generated in this study have been deposited in NCBI’s SRA database under accession Bioproject code PRJNA475869. Genome assemblies and annotations generated in this study are available on the European Nucleotide Archive (ENA) under project accession code PRJEB48264. Accession numbers of downloaded Illumina sequences or genome assemblies are provided in Supplementary Data 2. Details regarding the location of source data for Figs. 2–6, as well as Supplementary Figs. 3–15, and 17–29 can be found under the “Source Data” heading of the Github repository, https://perisd.github.io/Sac2.0/. Raw data generated in this study is deposited in FigShare (10.6084/m9.figshare.17185874).

## Code availability

The https://perisd.github.io/Sac2.0/ website provides access to custom scripts.
