# Supplementary material for: Macroevolutionary diversity of traits and genomes in the model yeast genus Saccharomyces
Source: Nat Commun. 2023 Feb 8;14:690. doi: 10.1038/s41467-023-36139-2 (PMC9908912; doi:10.1038/s41467-023-36139-2)
Supplement: Supplementary file 1 — Supplementary Information [file 41467_2023_36139_MOESM1_ESM.pdf]

Supplementary Figures and Notes

Macroevolutionary diversity of traits and genomes in the model yeast genus *Saccharomyces*

David Peris*, Emily J. Ubbelohde, Meihua Christina Kuang, Jacek Kominek, Quinn K. Langdon, Marie Adams, Justin A. Koshalek, Amanda Beth Hulfachor, Dana A. Opulente, David J. Hall, Katie Hyma, Justin C. Fay, Jean-Baptiste Leducq, Guillaume Charron, Christian R. Landry, Diego Libkind, Carla Gonçalves, Paula Gonçalves, José Paulo Sampaio, Qi-Ming Wang, Feng-Yan Bai, Russel L. Wrobel, Chris Todd Hittinger*

***Corresponding authors:** E-mails: david.perisnavarro@iata.csic.es, cthittinger@wisc.edu

Table of Contents

Supplementary Notes 4

Supplementary Note 1 – Nuclear and mitochondrial genome diversity 4

Supplementary Note 2 – *Saccharomyces* phenotypic diversity........ 5

Supplementary Figures 9

Supplementary Figure 1. Geographic locations of *Saccharomyces* populations. 10

Supplementary Figure 2. Association biases for hosts and substrates of *Saccharomyces* strains. 11

Supplementary Figure 3. *COX3* phylogenetic network of *Saccharomyces* strains. 12

Supplementary Figure 4. Phylogenetic networks of mitochondrial genes. 13

Supplementary Figure 5. Genome dot plots of *Saccharomyces* strains compared to the *S. cerevisiae* S288C laboratory strain. 14

Supplementary Figure 6. Highly diverse genomic architectures among *Saccharomyces* species. 15

Supplementary Figure 7. Mitochondrial genome dot plots of *Saccharomyces* strains compared to the *S. cerevisiae* S288C laboratory strain. 16

Supplementary Figure 8. Mitochondrial genome dot plots of *Saccharomyces* populations compared to other populations. 17

Supplementary Figure 9. Population genomics of seven *Saccharomyces* species. 18

Supplementary Figure 10. Genome-wide pairwise nucleotide sequence divergence plots for admixture *Saccharomyces* strains. 20

Supplementary Figure 11. Genetic distance distributions. 21

Supplementary Figure 12. Fst distributions. 22

Supplementary Figure 13. BUCKy concordance primary tree and alternative topologies. 23

Supplementary Figure 14. Phylogenomic network of *Saccharomyces s*ingle-copy orthologous genes. 24

Supplementary Figure 15. Levels of heterozygosity among *Saccharomyces* strains. 25

Supplementary Figure 16. Introgressions between *S. cerevisiae* and *S. paradoxus* and recent admixture between *S. mikatae* populations. 26

Supplementary Figure 17. *Saccharomyces* 2-µm plasmid inheritance. 27

Supplementary Figure 18. Percentage of *Saccharomyces* that grew above OD_{600}=0.5 in various growth conditions 28

Supplementary Figure 19. Growth conditions promoting flocculation among *Saccharomyces* strains. 29

Supplementary Figure 20. Growth variation in simple sugars across concentrations and the impact of Gal4-binding sites 30

Supplementary Figure 21. Lag time and maximum growth rate correlations between low and high sugar concentrations. 32

Supplementary Figure 22. Lag time correlations between monosaccharides and their disaccharides or trisaccharides. 33

Supplementary Figure 23. Phenotypic variance across *Saccharomyces* species. 34

Supplementary Figure 24. Principal component analysis of maximum OD_{600}. 35

Supplementary Figure 25. Variance contributed to each component by growth condition. 37

Supplementary Figure 26. Kinetic parameters of *Saccharomyces* strains in different growth conditions. 38

Supplementary Figure 27. Melibiose phenotypic diversity generated through complex genomic ancestries. 40

Supplementary Figure 28. Individual phylogenetics trees of the *GAL*/*MEL* pathway. 42

Supplementary Figure 29. Maximum OD_{600} violin boxplots of *Saccharomyces* populations/groups. 43

Supplementary Figure 30. Summary statistics of *Saccharomyces* genome assemblies. 44

Supplementary Notes

Supplementary Note 1 – Nuclear and mitochondrial genome diversity

The lengths of chromosomes II, IV, VI, VIII, X, XIII, and XV differed among *Saccharomyces* strains. The differences in length were driven mainly by the presence of chromosomal translocations (Supplementary Figure 5, Supplementary Data 2). Most *S. cerevisiae*, most *S. paradoxus*, and all *S. kudriavzevii* strains were syntenic with *S. cerevisiae* S288C. The other *Saccharomyces* species had particular translocations relative to *S. cerevisiae* S288C. All *S. mikatae* and *S. jurei* strains share a common translocation between chromosomes VI and VII, as noted in a previous study [1]. *S. uvarum* and *S. eubayanus* share two translocations: between chromosomes II and IV and between VIII and XV. All *S. uvarum* populations fixed three translocations (IItIV, VItX and VIIItXV), except *S. uvarum* Australasia. *S. uvarum* Australasia maintains synteny with *S. eubayanus* genomes, where VItX is absent (Supplementary Data 2, Supplementary Figure 5). Both *S. arboricola* populations share a translocation between chromosomes IV and XIII. Particular strains from *S. paradoxus*, such as the America B strain UFRJ50816, America C strain yHDPN24, and the Hawaii strain UWOPS91-917.1, contain one or multiple new translocations. *S. mikatae* Asia A and *S. cerevisiae* Malaysia populations are also differentiated from other populations of the same species by unique translocations.

The *S. cerevisiae*, *S. paradoxus*, S. *jurei*, and *S. mikatae* genomes have lower GC-contents; *S. arboricola* and *S. kudriavzevii* genomes have intermediate GC-contents; and *S. eubayanus* and *S. uvarum* genomes have higher GC-contents (Supplementary Figure

6B). *Saccharomyces eubayanus* and *S. uvarum* grew better at lower temperatures (Supplementary Figure 26c-e), whereas in bacteria, a positive correlation between optimal temperature and GC-content has been observed [2]. Some species that grew better at lower temperatures (Figures 5, 26c-e), such as *S. arboricola*, *S. eubayanus*, and *S. uvarum*, also have the smallest mitochondrial genomes (Supplementary Figure 6c).

Supplementary Note 2 – *Saccharomyces* phenotypic diversity

S. cerevisiae was the most phenotypically diverse species (Figure 5a, Supplementary Figure 23, 24), but some species also showed high phenotypic variance in particular conditions, often driven by lineage-specific phenotypic traits. For example, *S. kudriavzevii* had strains able to grow in 2 % galactose, 5 g/L histidine, and 4 °C, while other *S. kudriavzevii* strains could not. Some strains of *S. cerevisiae* grew in 2 mM H_2O_2, most of which were from domesticated lineages (Supplementary Figure 29a). Wild populations of *S. cerevisiae* (Asia Islands, CHN IV, Malaysia, and North America) were more phenotypically similar than the domesticated lineages (Supplementary Figure 24a). In general, conditions with more diversity within species were 2 % galactose, 2 % maltose, 2 % melibiose, 5 g/L histidine, and 4 °C (Supplementary Figure 23a). Strains with negative values of PC1 (Figure 5a) were characterized by better growth in 2 % galactose and their ability to produce biomass from 2 % xylose (Figure 5b, Supplementary Figure 24a, 25). We note that all *Saccharomyces* strains produce little to no biomass from 2 % xylose, but the normalization accentuates the differences amongst the strains producing some biomass (Supplementary Figure 18). PC2 classified strains by their ability to grow at 37 °C (positive values) or at 4 °C (negative values) (Supplementary Figure 25). PC3 also

split strains by their ability to grow at lower temperatures (4 °C and 10 °C) (Supplementary Figure 24c, 25). The best performers at 4°C were *S. arboricola, S. eubayanus*, and *S. uvarum*, while the best performers at 37 °C were *S. cerevisiae* and some strains from *S. paradoxus* (Supplementary Figure 26c-e). This suggests that low temperature growth is an ancestral trait (Supplementary Figure 18, 26d-e).

Saccharomyces strains grew well in the presence of glucose, fructose, sucrose, raffinose, mannose, and in complex media, such as juice must, at temperatures of 22 °C (Supplementary Figure 18). However, there were conditions where particular species and populations performed better than other strains. In general, sugar concentration affected kinetic parameters. For example, growth rate in both galactose and fructose generally increased as concentrations increased (Supplementary Figure 21a-b). Despite this general trend, maximum growth rate at 2 % galactose was slightly lower than 0.4 % or had no further increase in *S. cerevisiae*, *S. paradoxus*, and *S. arboricola* (Supplementary Figure 20a). In the case of galactose, in all species except *S. mikatae*, the increase of maximum growth rate in response to increased concentrations was faster in strains with a higher total number of putative Gal4-binding sites upstream of *PGM1* and *PGM2*, which likely allows enhanced flux from the *GAL* pathway into glycolysis (Supplementary Figure 20e) [3]. Interestingly, lag time in both galactose and fructose did not have the same trend as growth rate; in fact, lag time in fructose was longer at a higher concentration for *S. arboricola*, *S. uvarum*, and *S. eubayanus* (Supplementary Figure 20d). Surprisingly, at the level of individual strains, for both galactose and fructose, a majority of strains (59 % for galactose and 76 % for fructose) showed a shorter lag time in 0.1 % than in 2 % (dots on the upper left side of the gray dash line) (Supplementary Figure 21a-b). Lag time

variation on disaccharides or trisaccharides at the strain level may be partly explained by the growth variation on their constituent monosaccharides or disaccharides (Supplementary Figure 22).

In the presence of maltose, non-domesticated *S. cerevisiae, S. arboricola, S. kudriavzevii* Asia A, *S. mikatae, S. eubayanus* Holarctic, and *S. uvarum* Australasia grew poorly (Supplementary Figure 29). Some *S. mikatae* and *S. arboricola* Asia A strains were found to produce more biomass in the presence of melibiose than other *Saccharomyces* (Supplementary Figure 18, 26c, 27c). Brewing and some *S. cerevisiae* wine/European strains were able to grow in the presence of maltotriose (Figure 5b, Supplementary Figure 29a), but surprisingly, some *S. mikatae* strains may also have been able to produce limited biomass from this carbon source (Supplementary Figure 18, 26c, 29c). European *S. kudriavzevii,* except two strains from a Europe-Asia A admixed lineage (Supplementary Figure 9d), grew on galactose as expected [4], while non-admixed strains from Asia A and Asia B did not (Figures 5b, 6b, Supplementary Figure 29d). As expected [5], *S. cerevisiae* West Africa did not grow well in the presence of galactose (Figure 5b, Supplementary Figure 29a). Some domesticated *S. cerevisiae*, *S. mikatae* Asia A, *S. kudriavzevii,* non-Holarctic *S. eubayanus,* and *S. uvarum* strains were able to grow at a higher pH (5 g/L histidine, pH 7.5) (Figure 5b, Supplementary Figure 18, 26, 29). *S. eubayanus* Patagonia B and few domesticated *S. cerevisiae* strains were more osmotolerant (1.4 NaCl) than other *Saccharomyces* strains (Supplementary Figure 18, 26c, 29g). With the exception of many *S. eubayanus* and *S. uvarum* strains, few *Saccharomyces* strains grew on glycerol during the timeframe of the experiment (Figure 5b, Supplementary Figure 26c).

Some growth conditions promoted flocculation in specific *Saccharomyces* strains (Supplementary Figure 19). In particular, mannose, 10 °C, 37 °C, histidine, and galactose were the most influential conditions, with more than 5 *Saccharomyces* strains flocculating in each.

Supplementary Figures

Supplementary Figure 1

a

S. cerevisiae populations

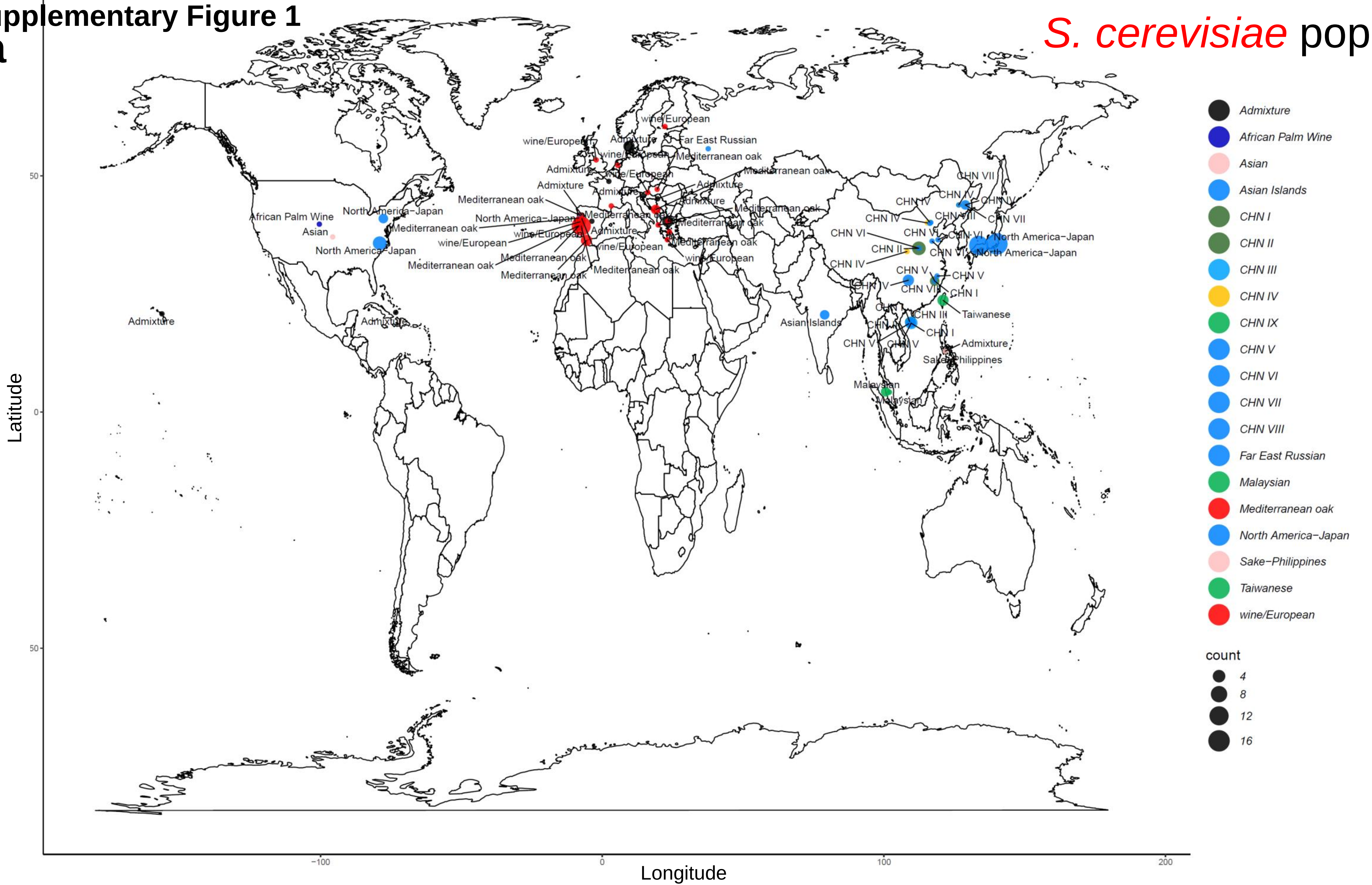

Supplementary Figure 1

b

S. paradoxus populations

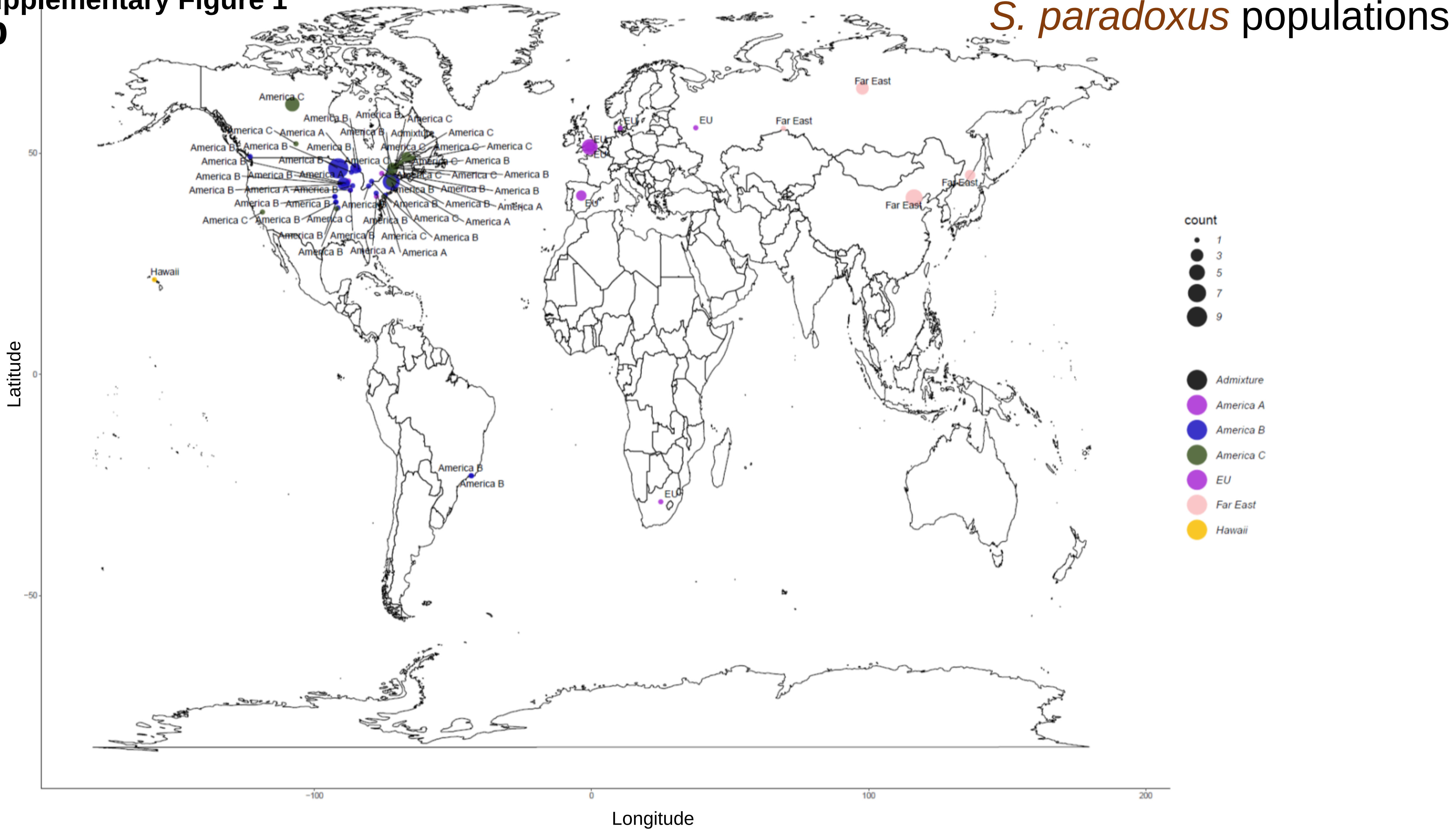

Supplementary Figure 1

c

S. mikatae populations

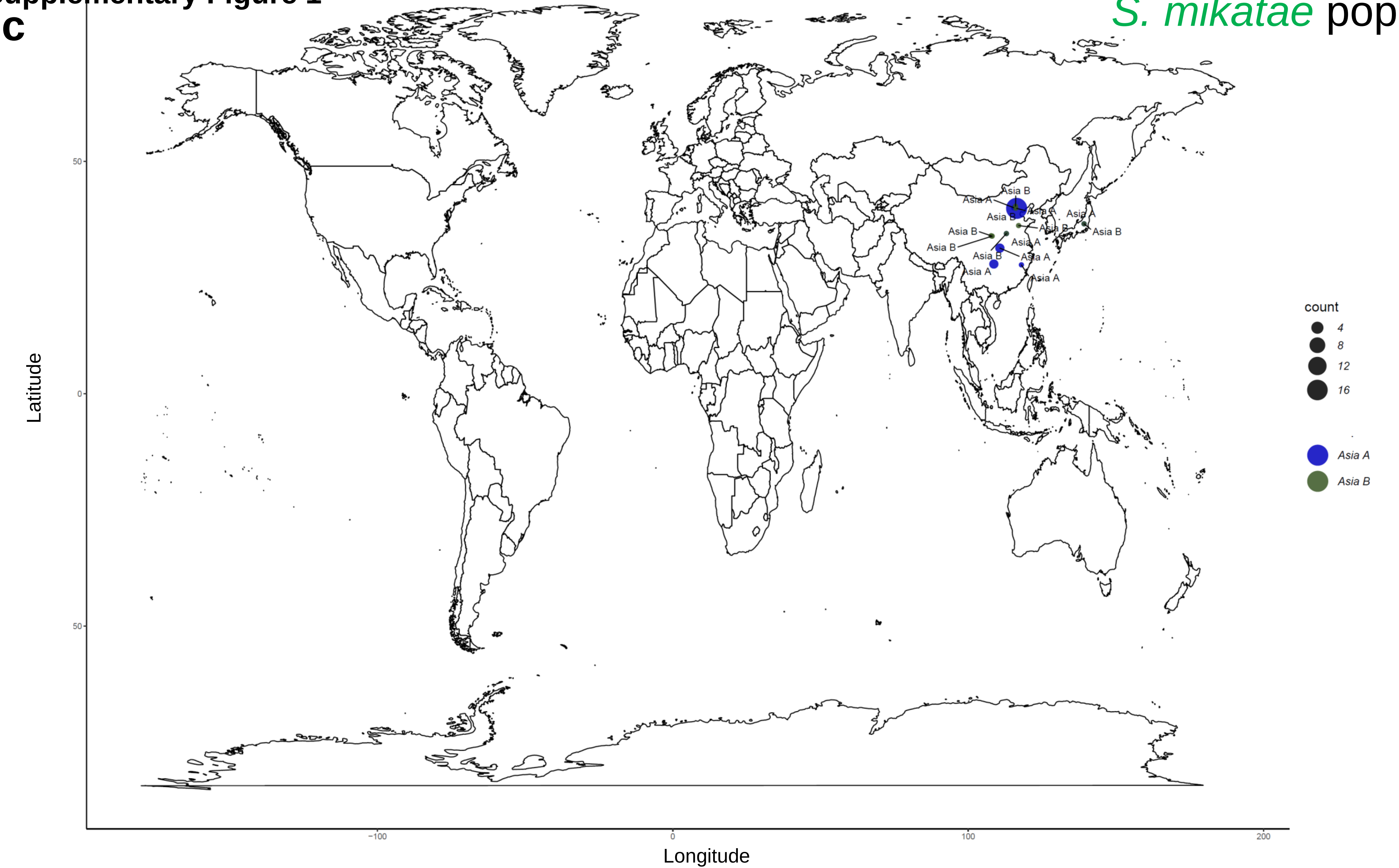

Supplementary Figure 1

d

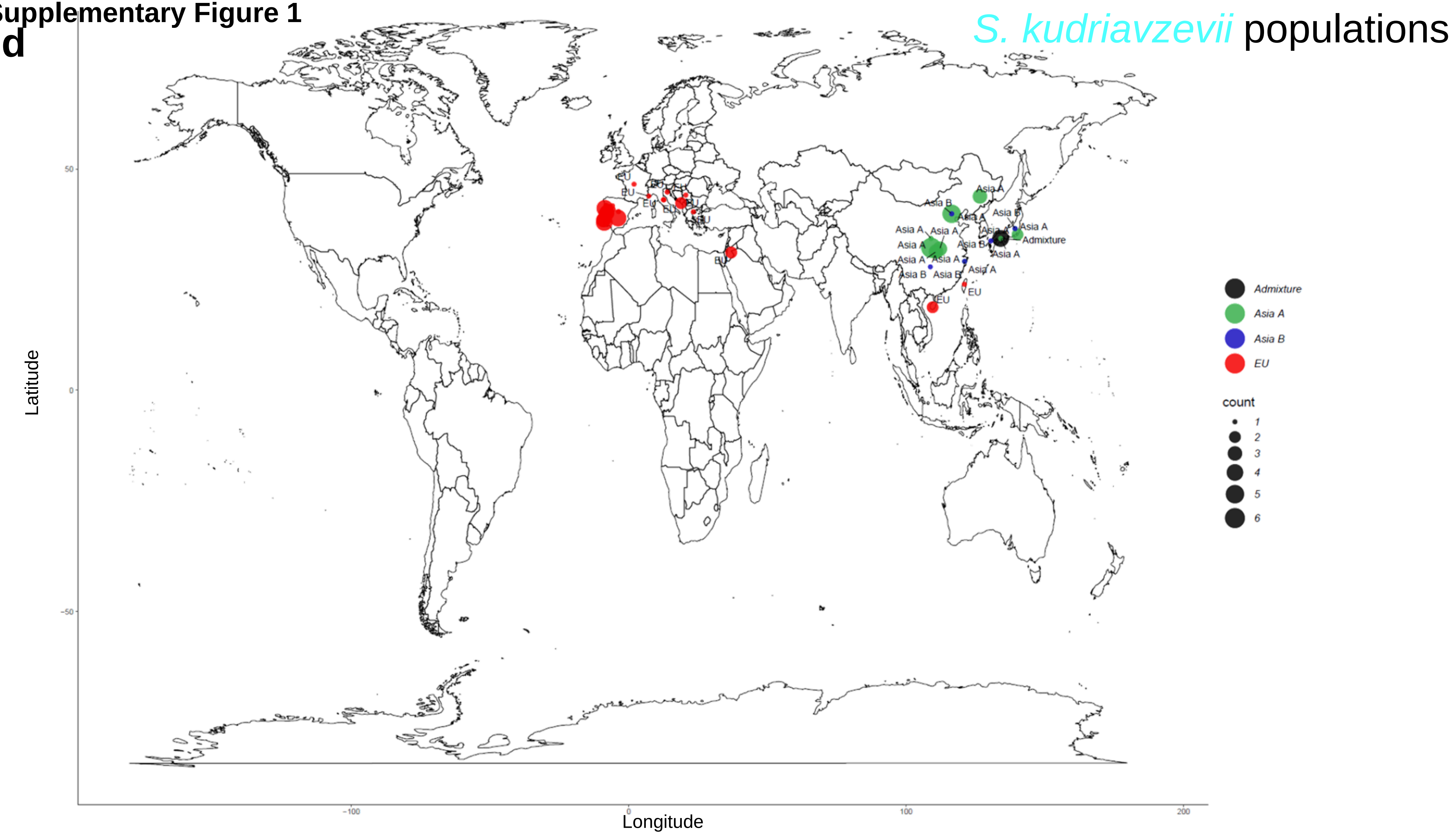

Supplementary Figure 1

e

S. arboricola populations

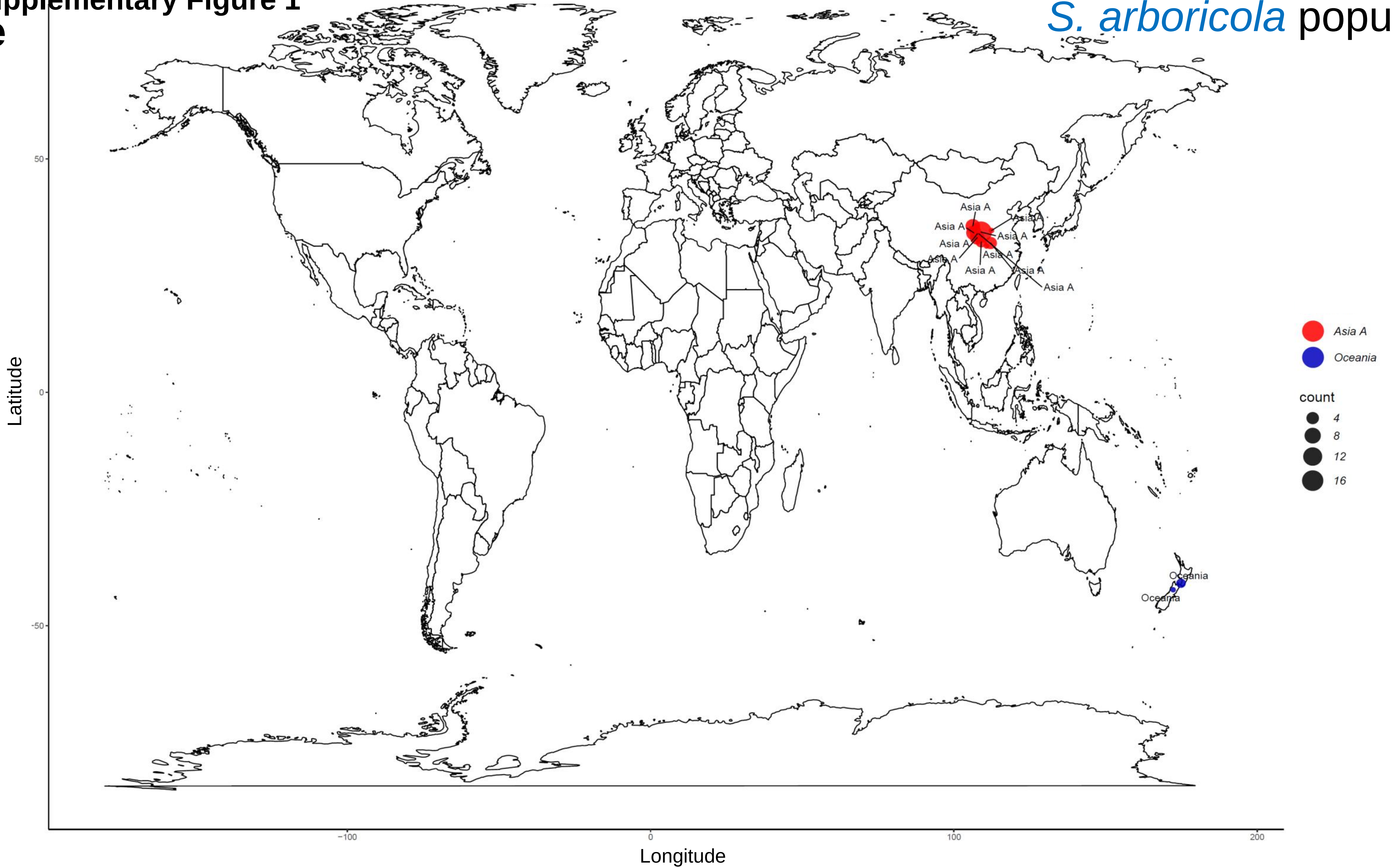

Supplementary Figure 1

f

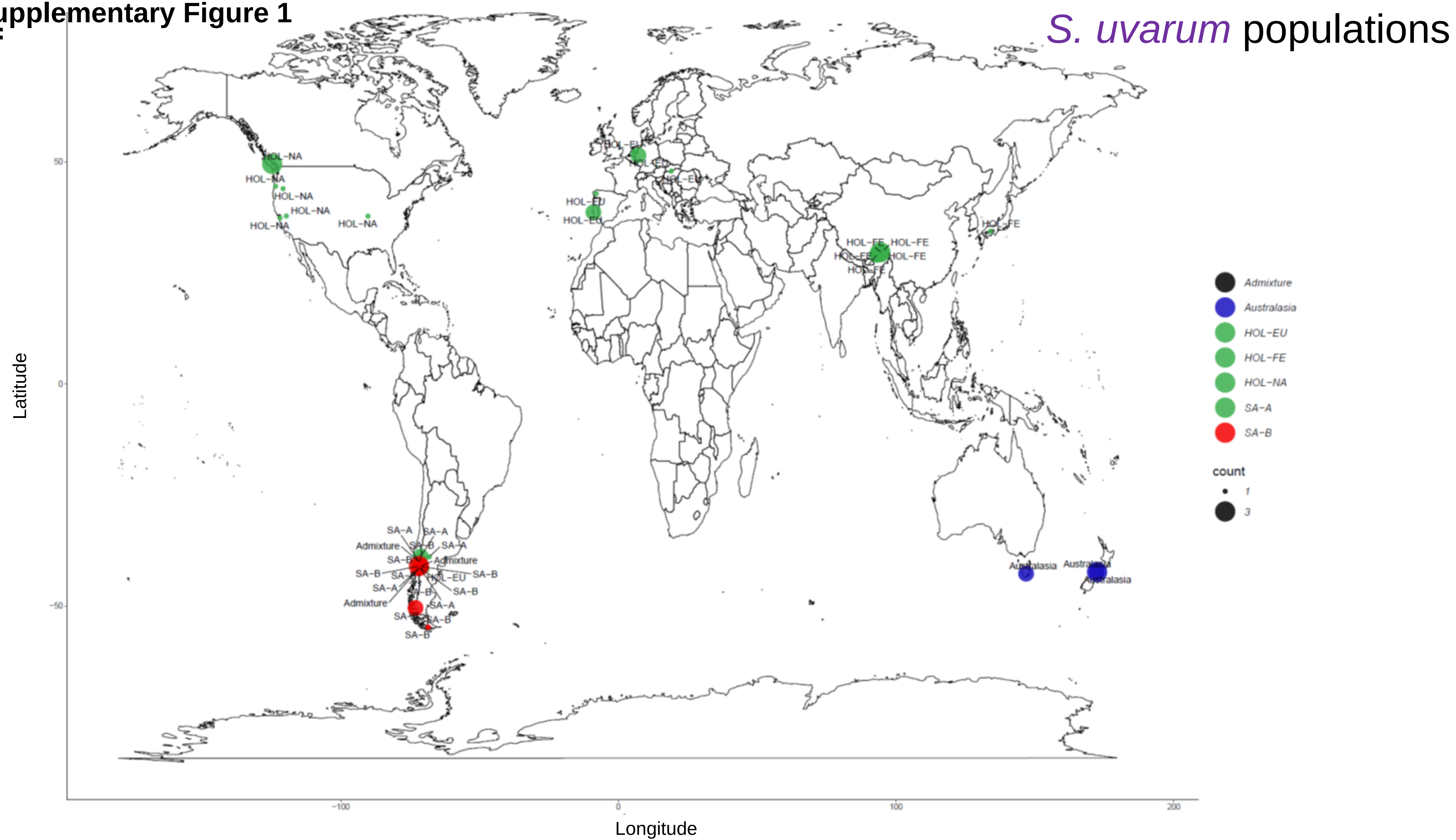

Supplementary Figure 1

g

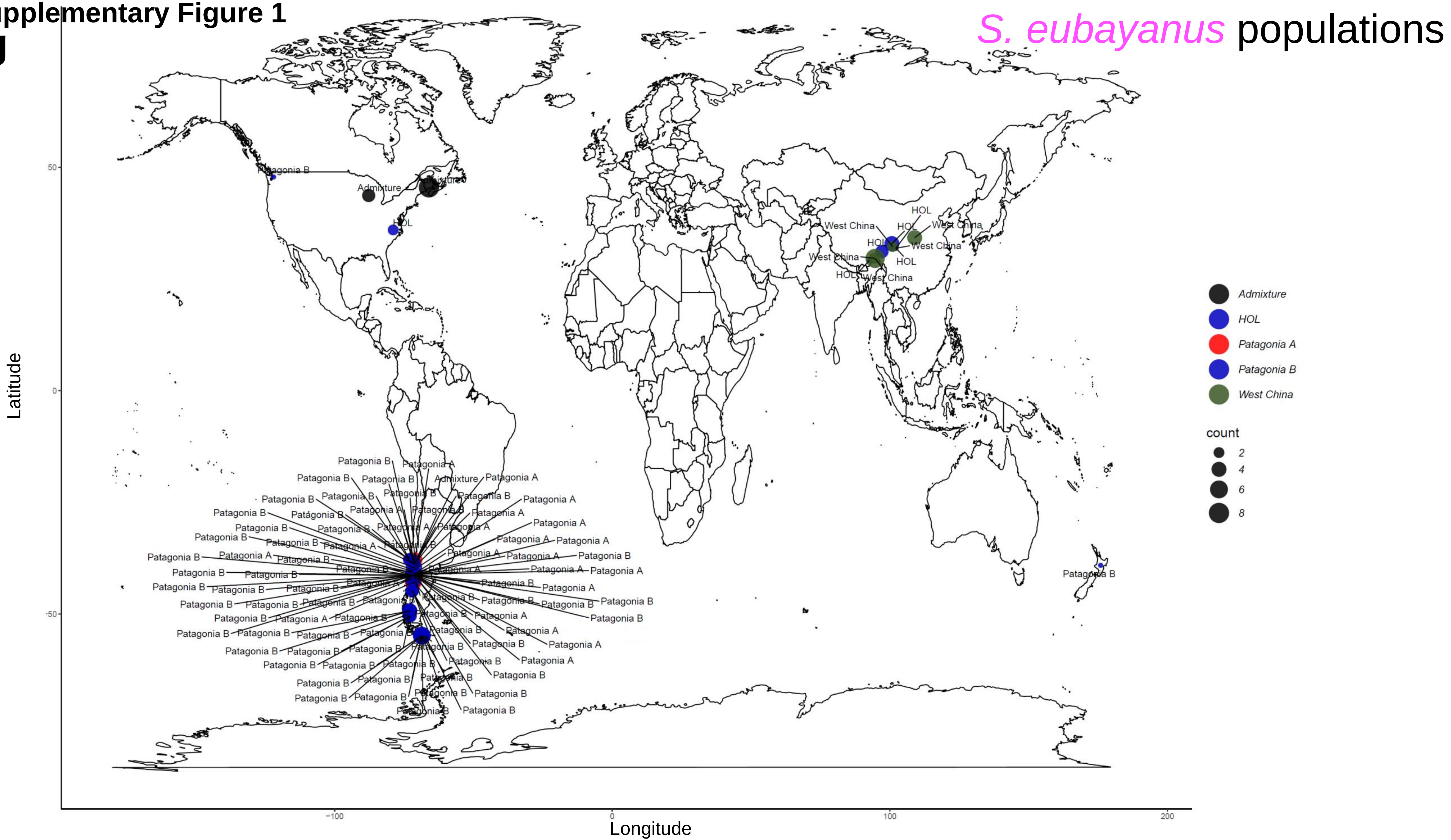

Supplementary Figure 1. Geographic locations of *Saccharomyces* populations.

Panels **a-g**) show the geographic distribution of wild populations for each *Saccharomyces* species (Supplementary Data 1). Note that anthropic strains are not shown for clarity. The size of the symbols represent the number of strains from each locations. For clarity, symbol colors represent phylogenetic relationships (group or population) according to the legend and results in Supplementary Figure 9. CHN: China; EU: Europe; FE: Far East; HOL: Holarctic; NA: North America; SA-A: South America A; SA-B: South America B. The maps were generated using the `map_data` function implemented in `R` package `ggplot2` [6].

Supplementary Figure 2

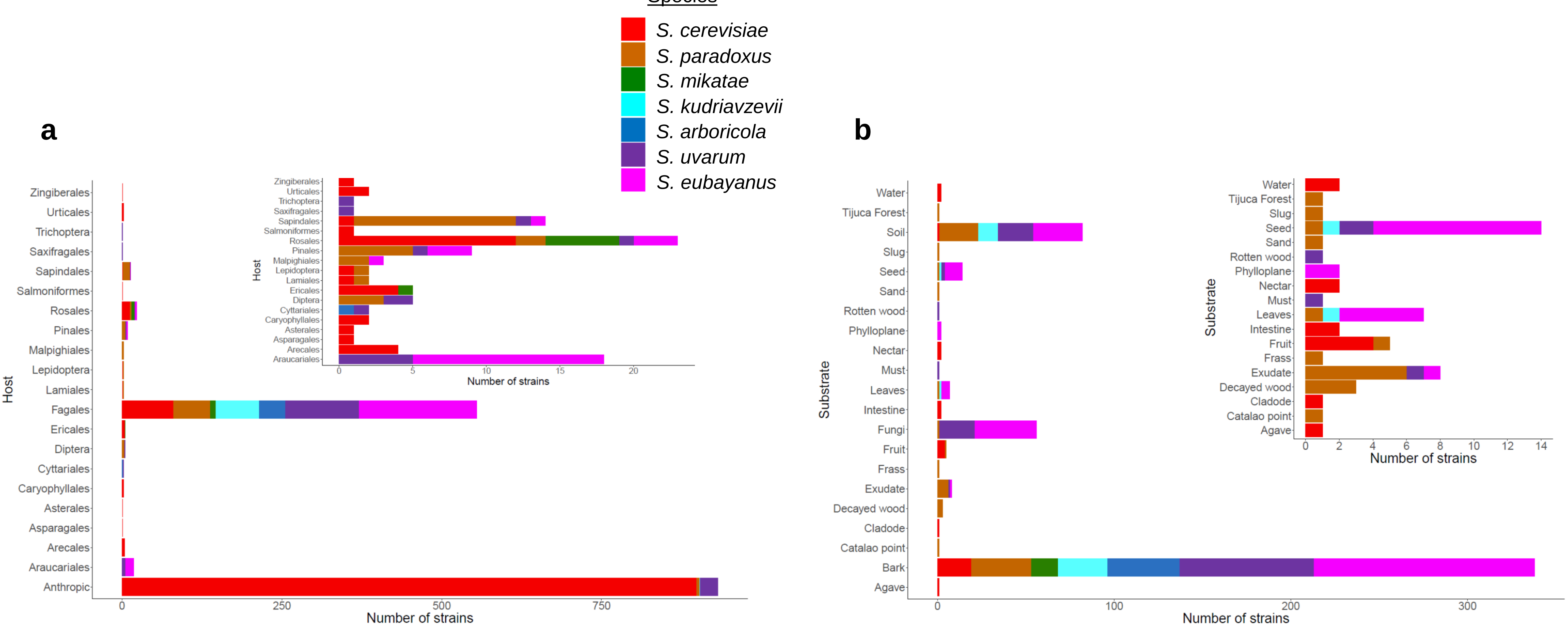

Supplementary Figure 2. Association biases for hosts and substrates of *Saccharomyces* strains.

a) A stacked barplot for *Saccharomyces* strains isolated from different hosts (Supplementary Data 1). The taxonomic rank of order was used to group *Saccharomyces* isolates by their host order. Human-related environments, such as vineyards, were grouped in the "Anthropic" category. **b**) A stacked bar plot for wild *Saccharomyces* strains isolated from different substrates (Supplementary Data 1). Bar plots are colored according to species.

Supplementary Figure 3

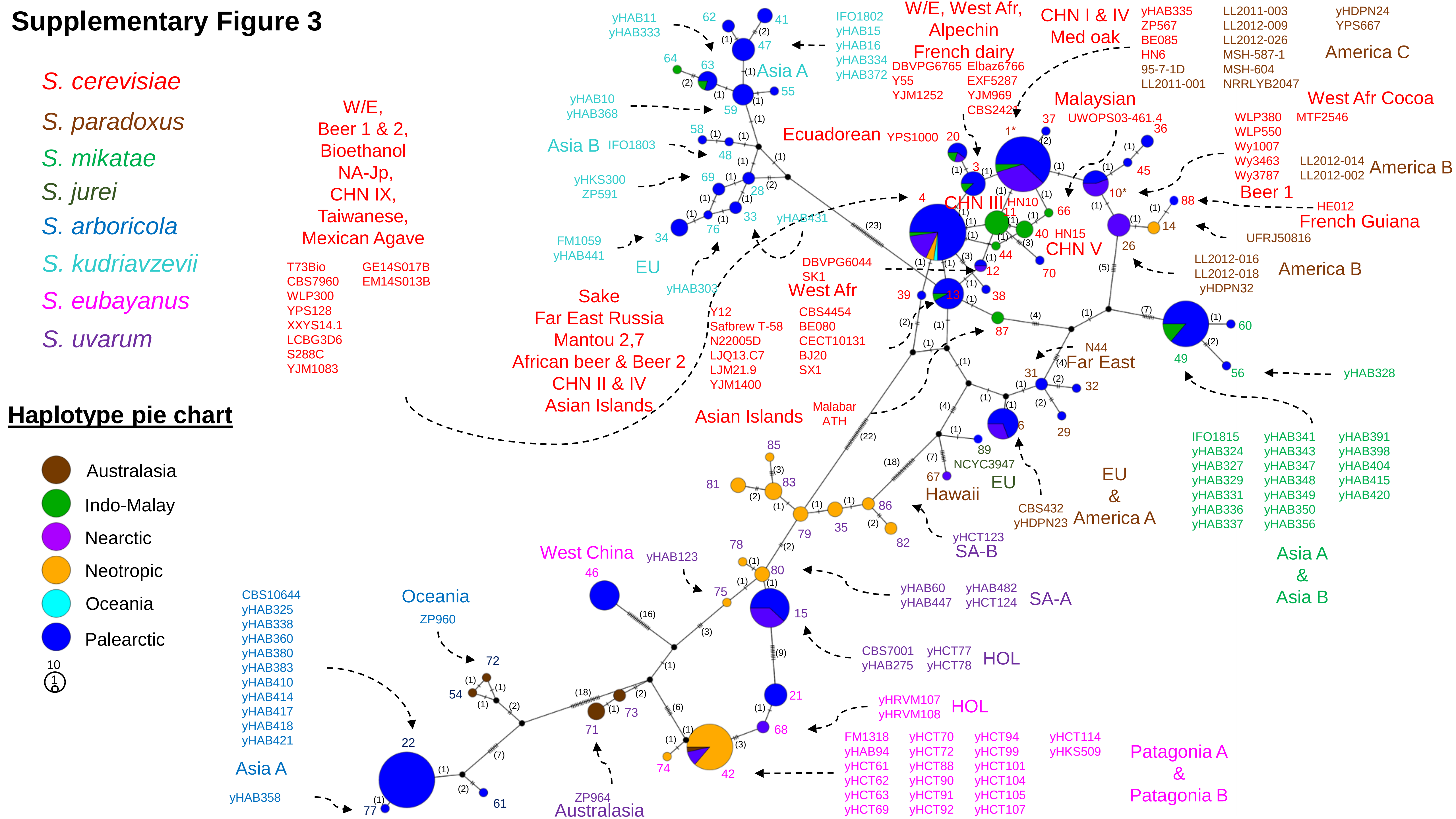

Supplementary Figure 3. *COX3* phylogenetic network of *Saccharomyces* strains.

A Templeton, Crandall, and Sing (TCS) phylogenetic network of 395 trimmed *COX3* sequences from wild *Saccharomyces* strains is shown. The *COX3* haplotype classification for the wild and anthropic *Saccharomyces* strains is shown in Supplementary Data 1 (n = 996 *COX3* sequences). Haplotypes are represented by circles, and haplotype names are colored based on species designations. When two different species shared the same haplotype, the haplotype name has both colors and is highlighted with an asterisk. Circle size is scaled according to the haplotype frequency. Pie charts show the frequency of haplotypes based on the biogeographic realm of origin. The number of mutations separating each haplotype is indicated by lines on the edges connecting different haplotype circles and by numbers between parentheses. Strain names for those yeasts phenotyped or studied in the main text are indicated. Population and strain names are colored according to their species designations. Afr: Africa; CHN: China; EU: European; HOL: Holarctic; Med: Mediterranean; NA-Jp: North America-Japan (=North America); SA-A: South America A; SA-B: South America B; W/EU: Wine/European.

Supplementary Figure 4

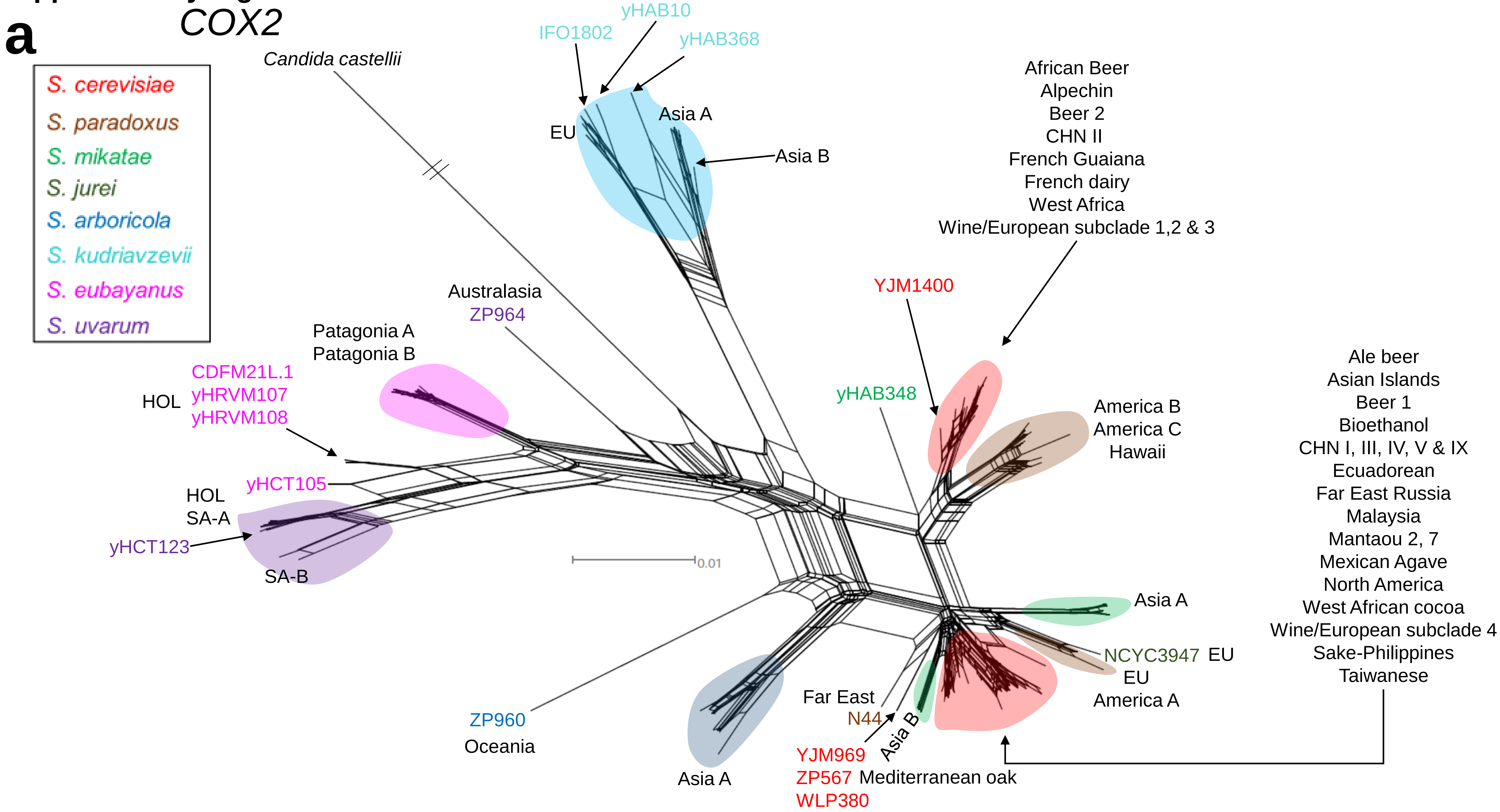

Supplementary Figure 4

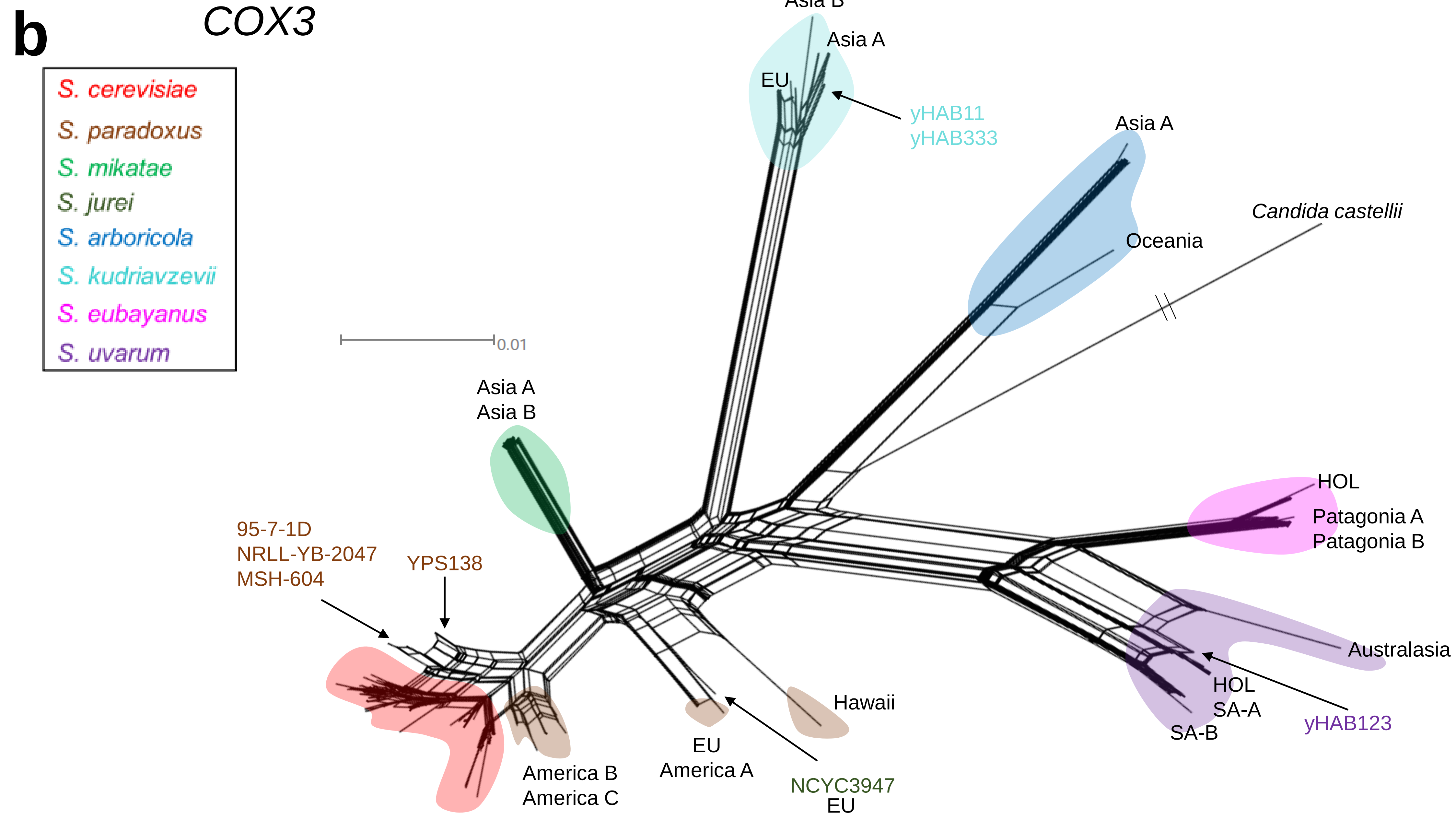

Supplementary Figure 4

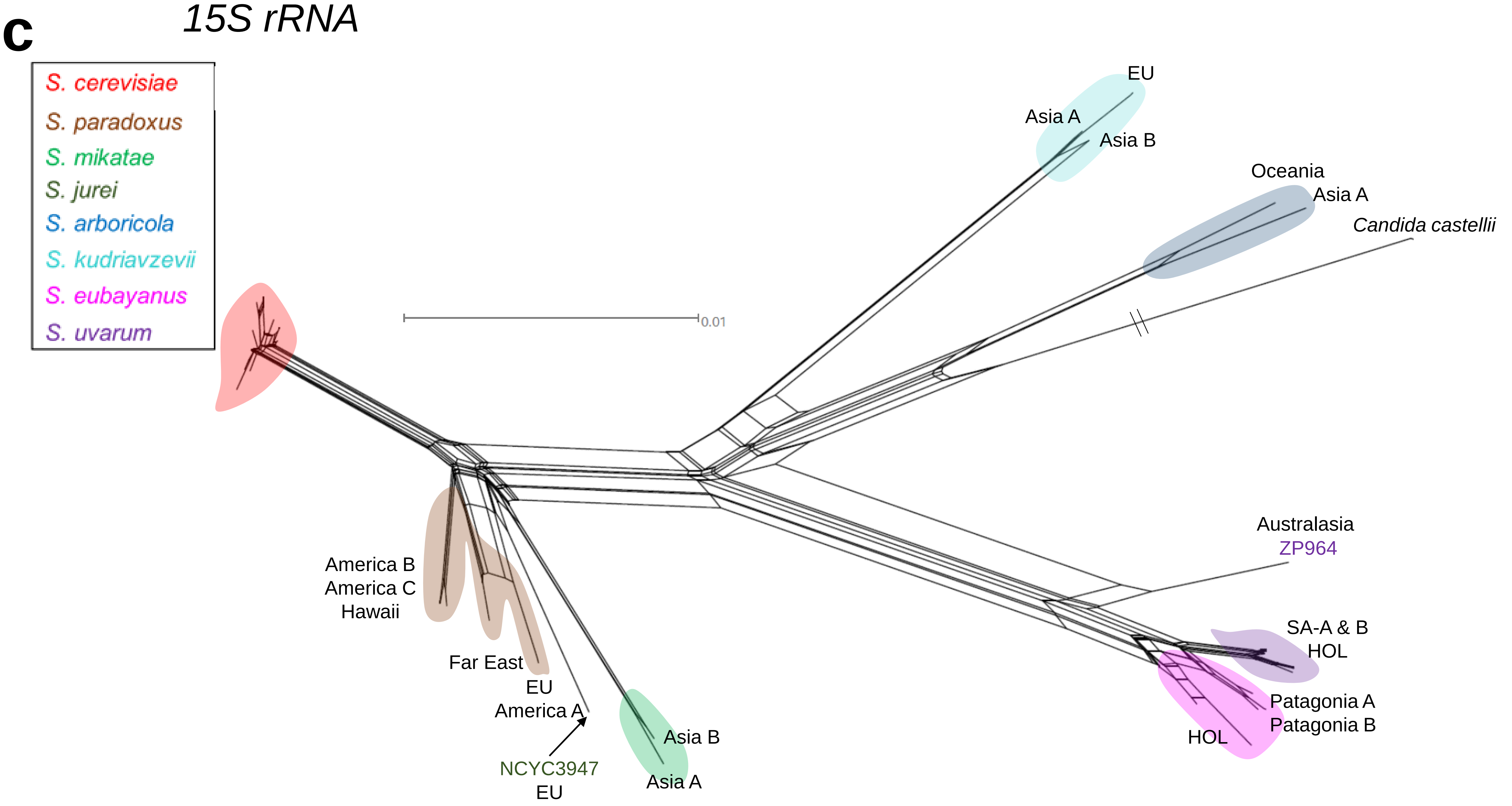

Supplementary Figure 4

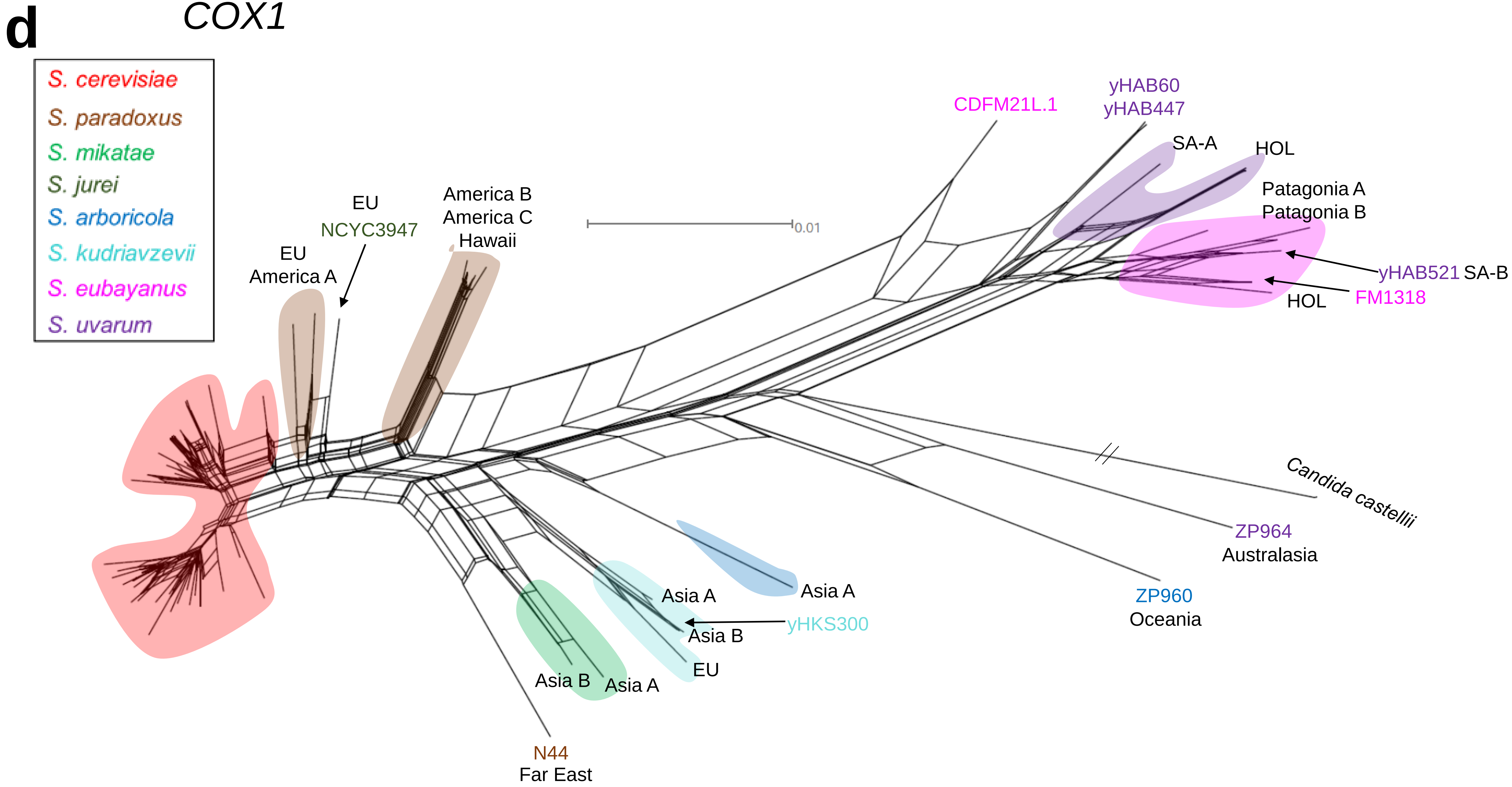

Supplementary Figure 4

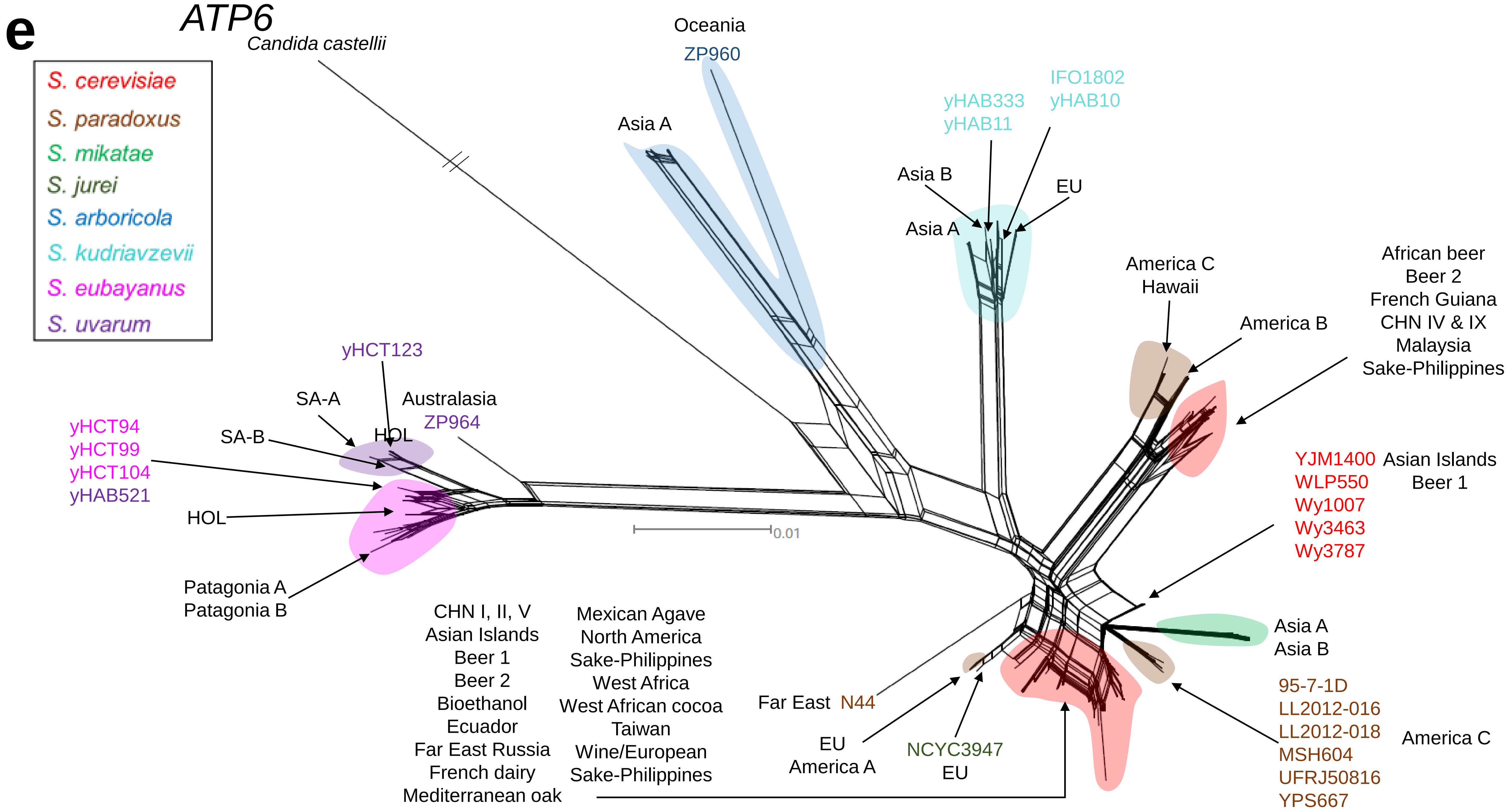

Supplementary Figure 4

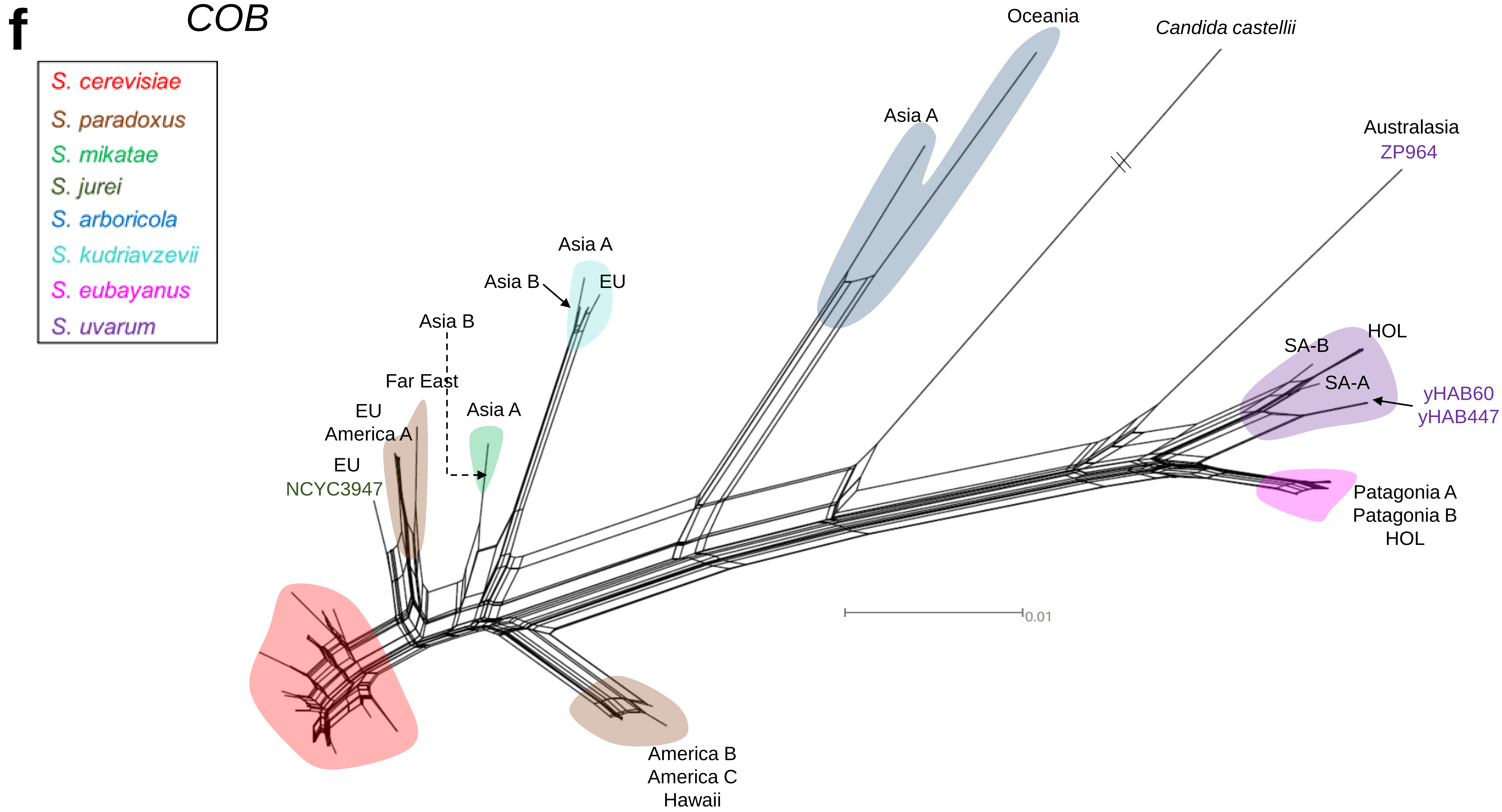

Supplementary Figure 4

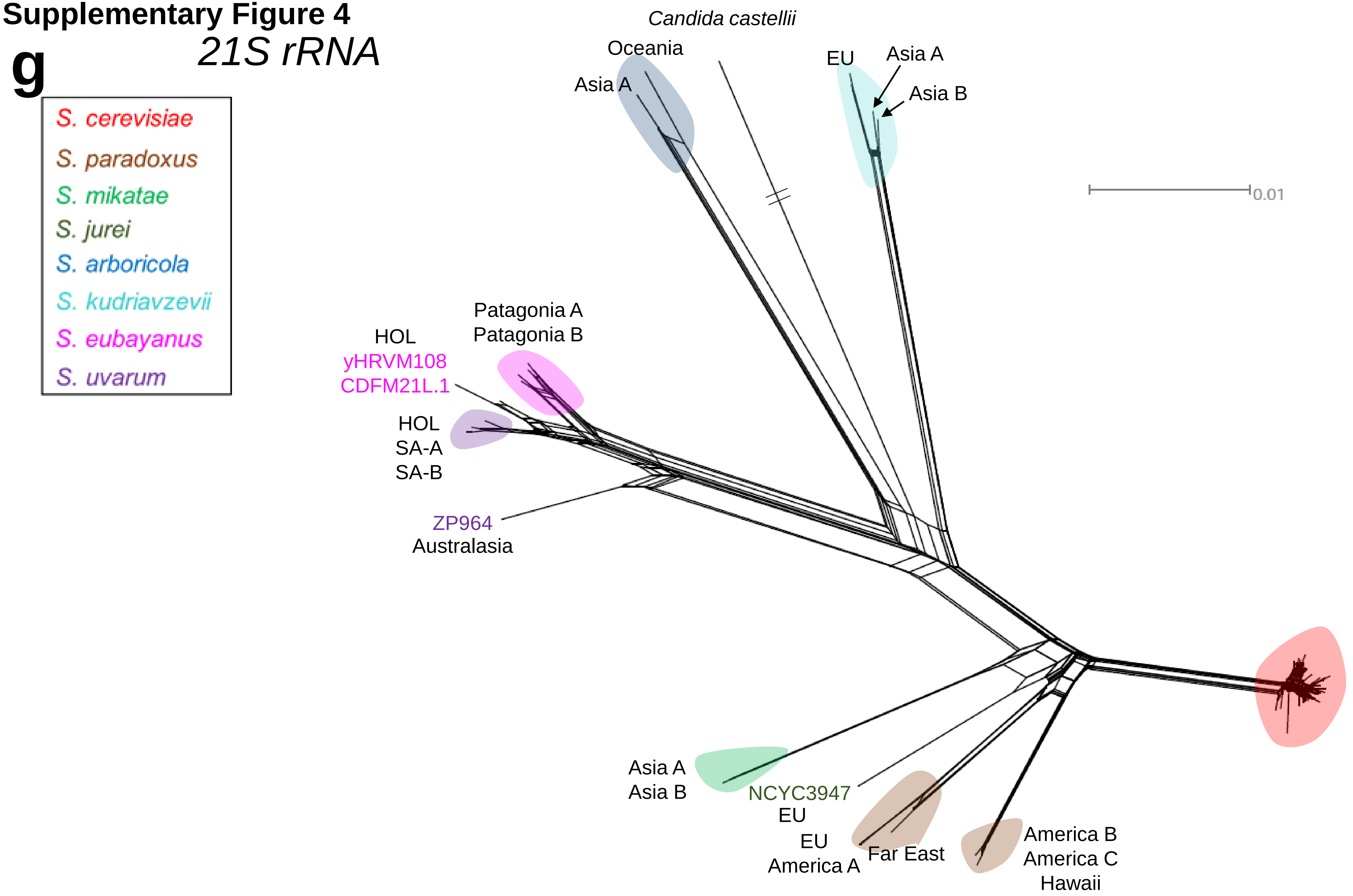

Supplementary Figure 4. Phylogenetic networks of mitochondrial genes.

Neighbor-Net phylogenetic networks for informative mitochondrial genes are shown in panels **a-g**). For the *COB* gene, the *S. mikatae* Asia B position is indicated with a discontinuous arrow due to an incomplete *COB* gene reconstruction. Strains found in an unexpected location are noted and colored according to their species designations. Populations are designated for all species, except for *S. cerevisiae* when all *S. cerevisiae* populations are clustered together. Scale bars represent nucleotide substitutions per site. *Candida castellii* (syn. ‘*Nakaseomyces*' *castellii*) is the outgroup.

Supplementary Figure 5

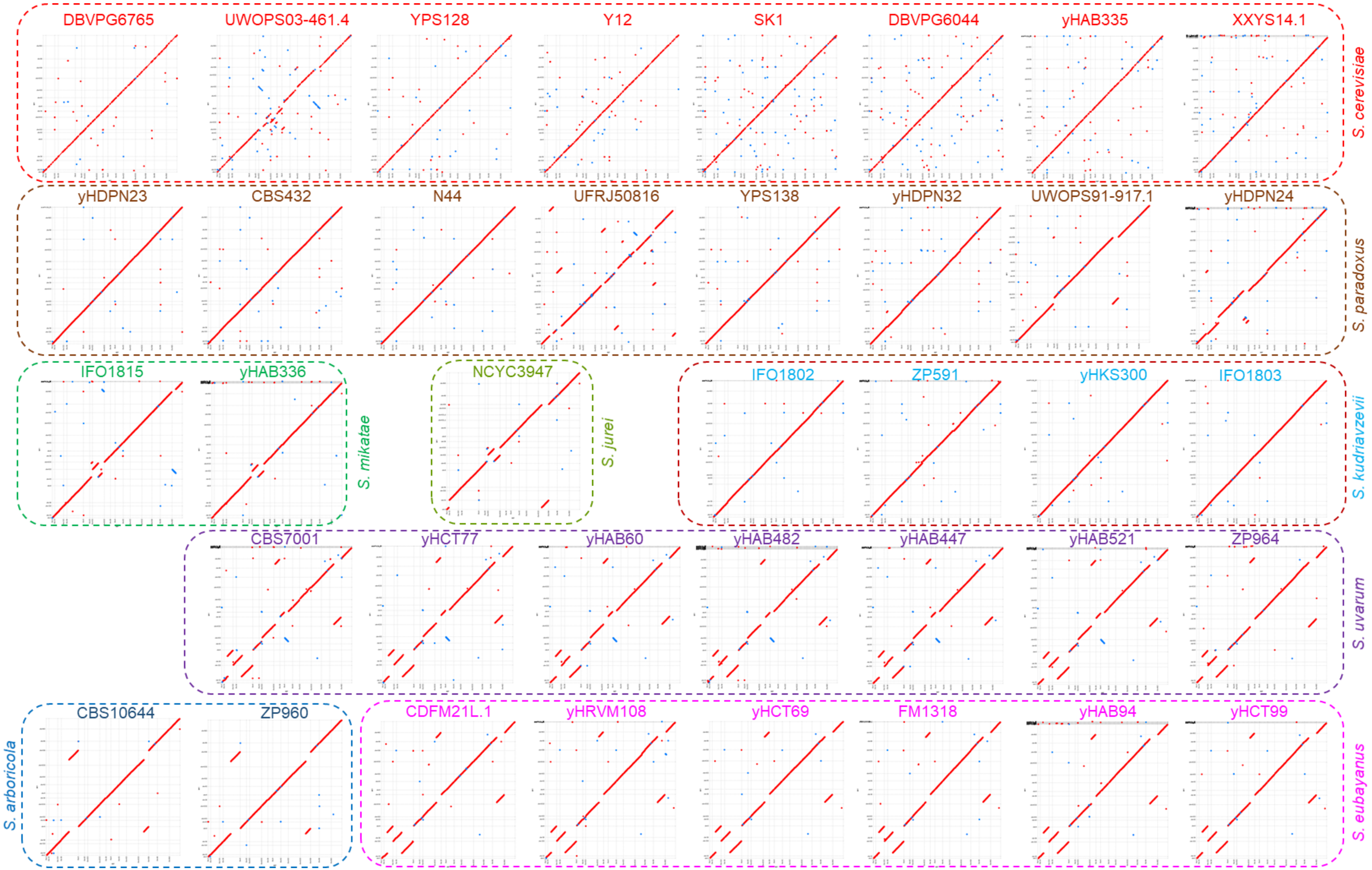

Supplementary Figure 5. Genome dot plots of *Saccharomyces* strains compared to the *S. cerevisiae* S288C laboratory strain.

Syntenic maps between *Saccharomyces* strains and *S. cerevisiae* (S288C). Blue dots indicate inversions. Strain names are colored according to their species designations. We have also provided high-quality figures in the dedicated FigShare repository (https://dx.doi.org/10.6084/m9.figshare.17185874) where chromosome and scaffold boundaries are marked and can be observed on the axes. Chromosomal translocations compared to S288C are highlighted in the coalescent phylogenetic tree (Figure 4a). Roman numerals represent chromosomes.

Supplementary Figure 6

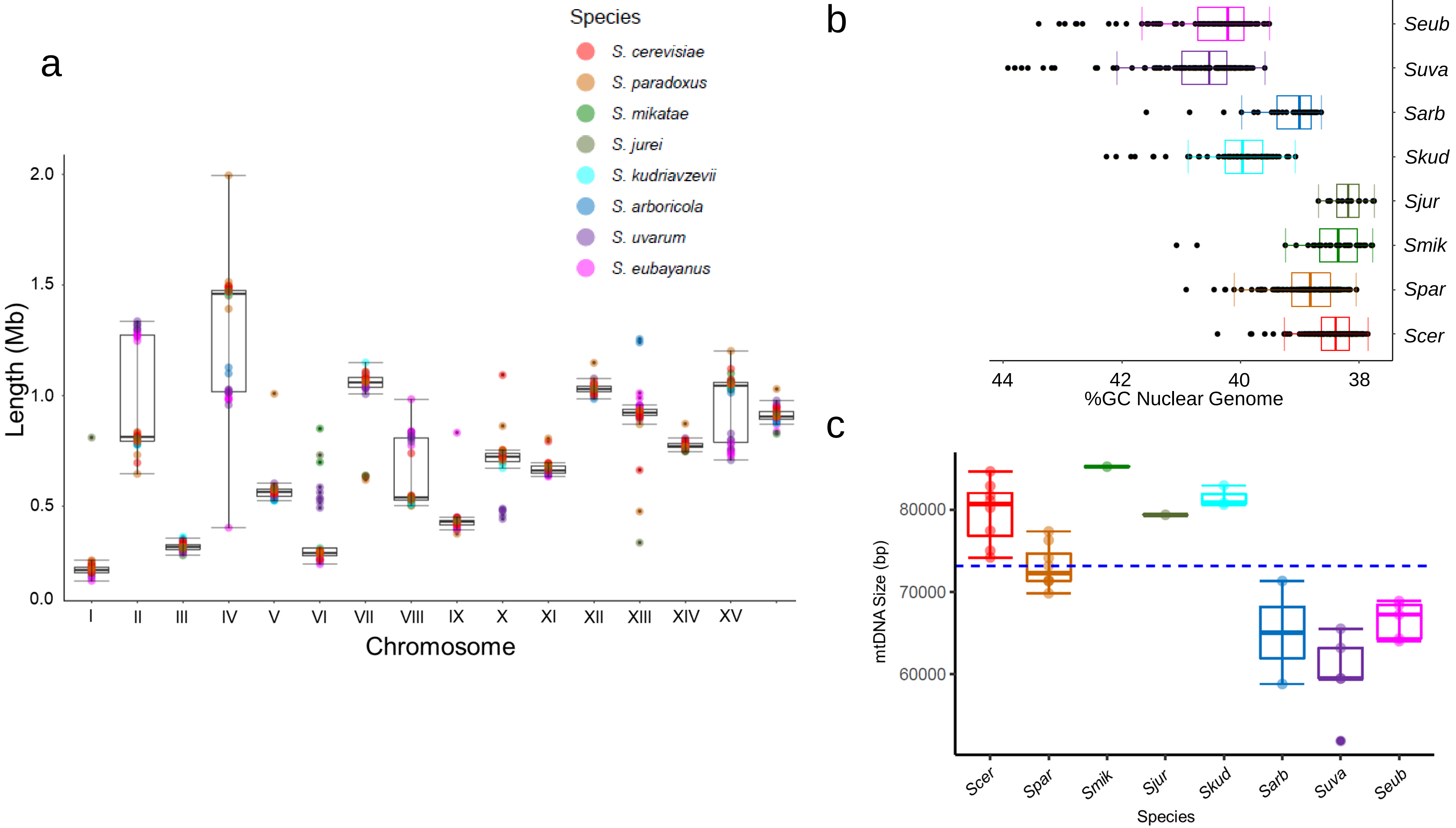

Supplementary Figure 6. Highly diverse genomic architectures among *Saccharomyces* species.

Chromosome length, percent of GC in the nuclear genome, and mitochondrial genome (mtDNA) size for *Saccharomyces* strains (n = 38) are shown in panel **a**), **b**), and **c**), respectively. Dots or boxplots are colored according to the species designations. Median values for the chromosomes are represented by a horizontal line inside the box, and the upper and lower whiskers represent the highest and lowest values of the 1.5 * IQR (inter-quartile range), respectively. In panel **b**), the dots correspond to the GC-content for each chromosome per strain. The dashed blue line in panel **c**) corresponds to the median mitochondrial genome size of all mitochondrial genomes (Supplementary Data 2). In panels **b**) and **c**), median values for the species are represented by a horizontal line inside the box.

Supplementary Figure 7

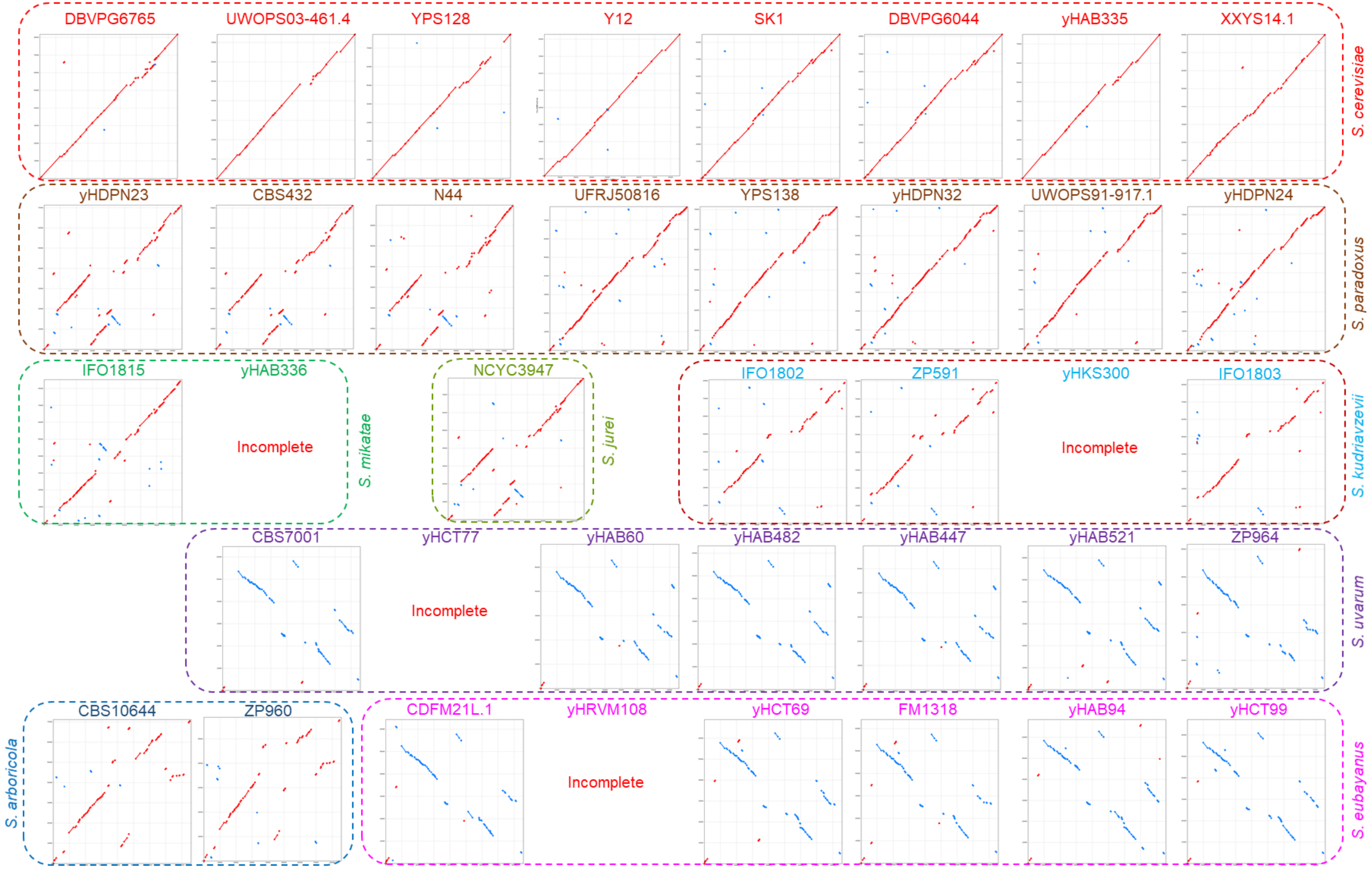

Supplementary Figure 7. Mitochondrial genome dot plots of *Saccharomyces* strains compared to the *S. cerevisiae* S288C laboratory strain.

Syntenic maps between *Saccharomyces* strains and *S. cerevisiae* (S288C). For consistency, all mitochondrial genomes were oriented to set the first nucleotide as the gene encoding tRNA-Serine, which is close to *VAR1*. Blue dots indicate inversions. Strain names are colored according to their species designations. Axes are represented in base pairs, and dashed lines are plotted every 10 kbp. Regions relocated compared to S288C are highlighted in the coalescent phylogenetic tree (Figure 4a).

Supplementary Figure 8

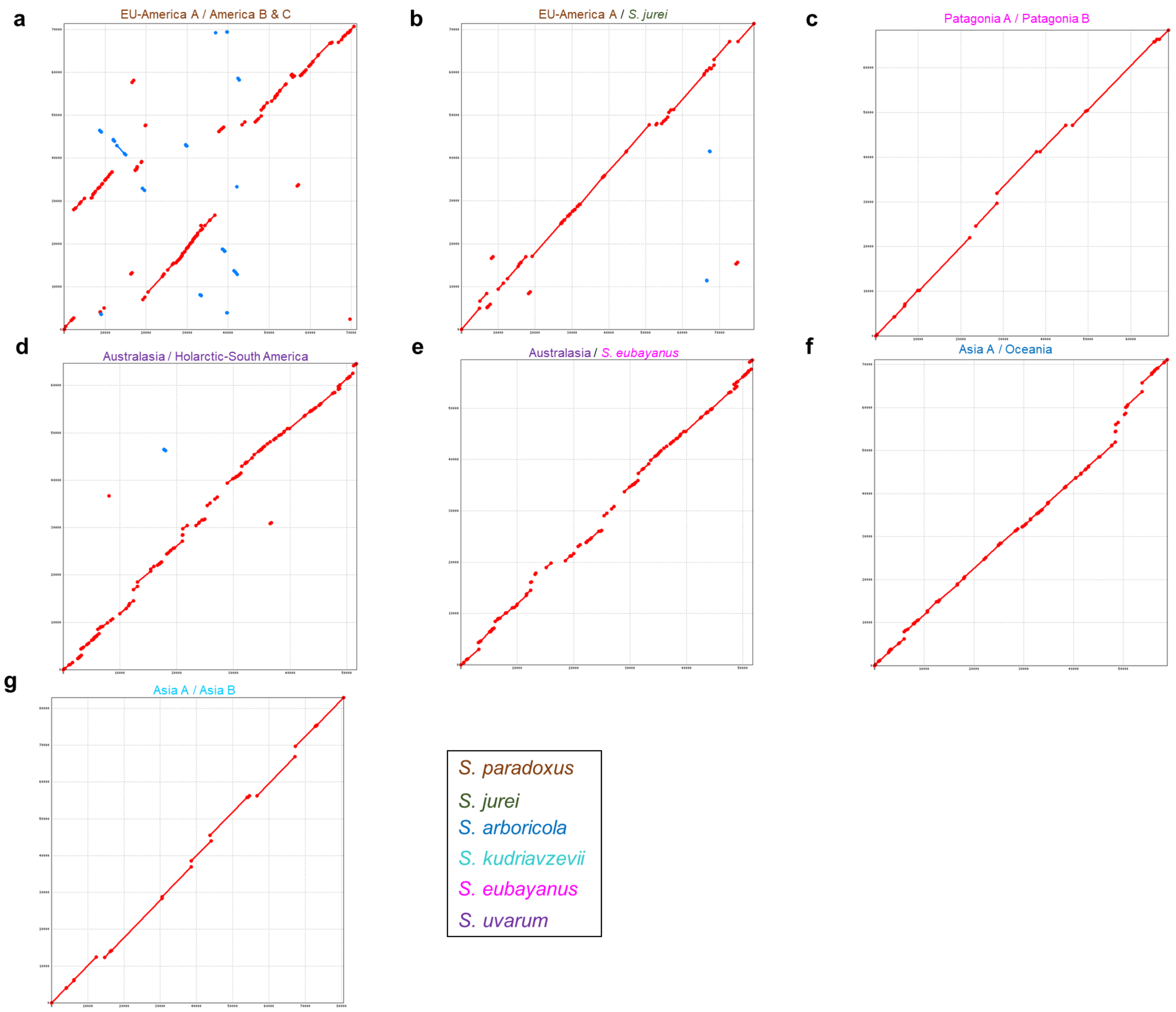

Supplementary Figure 8. Mitochondrial genome dot plots of *Saccharomyces* populations compared to other populations.

Syntenic maps between strains representatives of *Saccharomyces* populations and representative strains of other populations to highlight the diversity in the architectures among them (Supplementary Data 1). For consistency, all mitochondrial genomes were oriented to set the first nucleotide as the gene encoding tRNA-Serine, which is close to *VAR1*. Blue dots indicate inversions. Population names were colored according to their species designations. Axes are represented in base pairs, and dashed lines are plotted every 10 kbp. Mitochondrial genome syntenic map between *S. paradoxus* EU-America A and *S. paradoxus* America B/C is displayed in panel **a)**, between *S. paradoxus* EU-America A and *S. jurei* in panel **b)**, between *S. eubayanus* Patagonia A and *S. eubayanus* Patagonia B in panel **c)**, between *S. uvarum* Australasia and *S. uvarum* Holarctic-South America (A & B) in panel **d)**, *S. uvarum* Australasia and *S. eubayanus* (Patagonia A & Holarctic-B) in panel **e)**, *S. arboricola* Asia A and *S. arboricola* Oceania in panel **f)**, and *S. kudriavzevii* Asia A and Asia B in panel **g)**.

Supplementary Figure 9

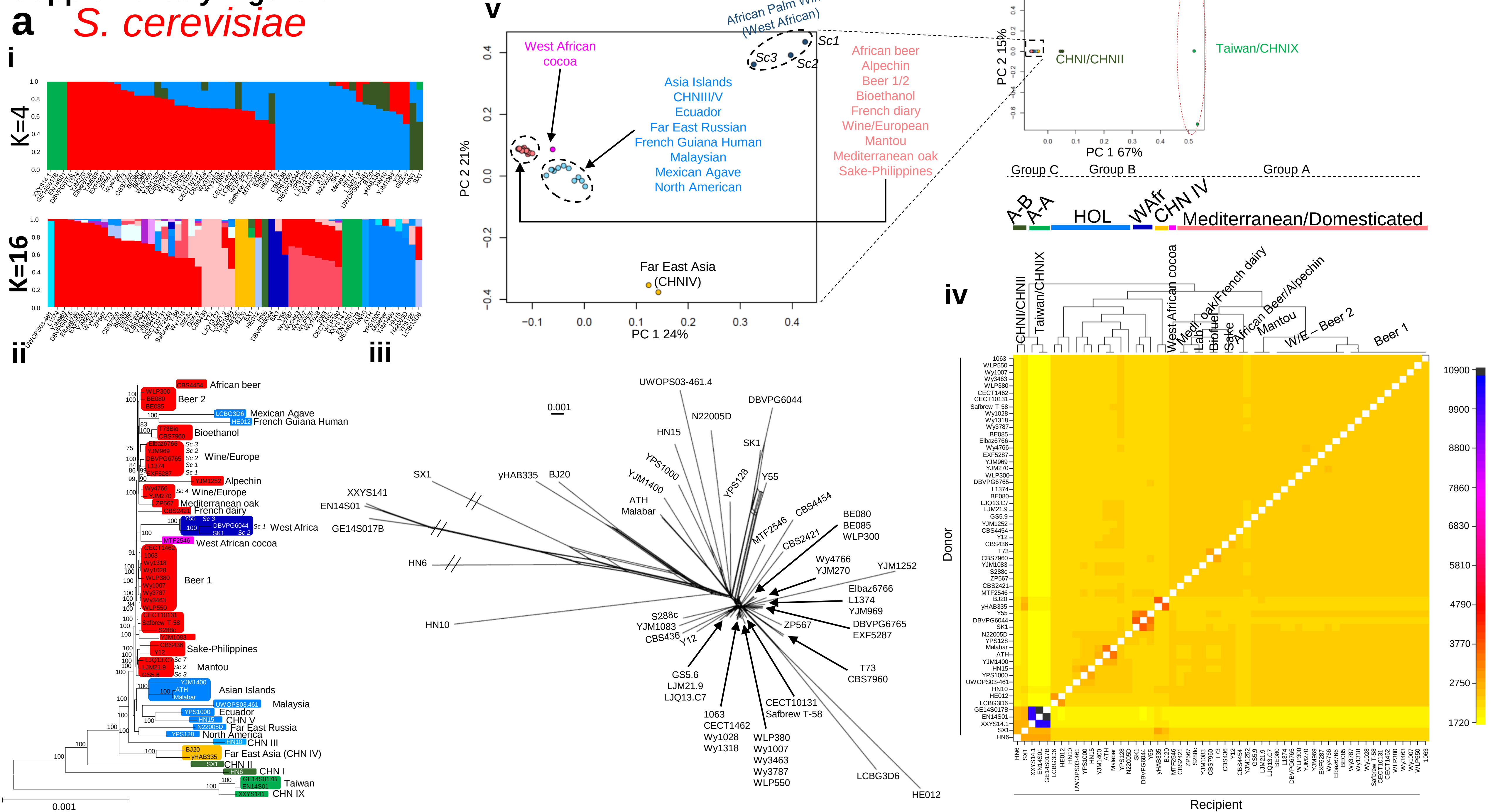

Supplementary Figure 9

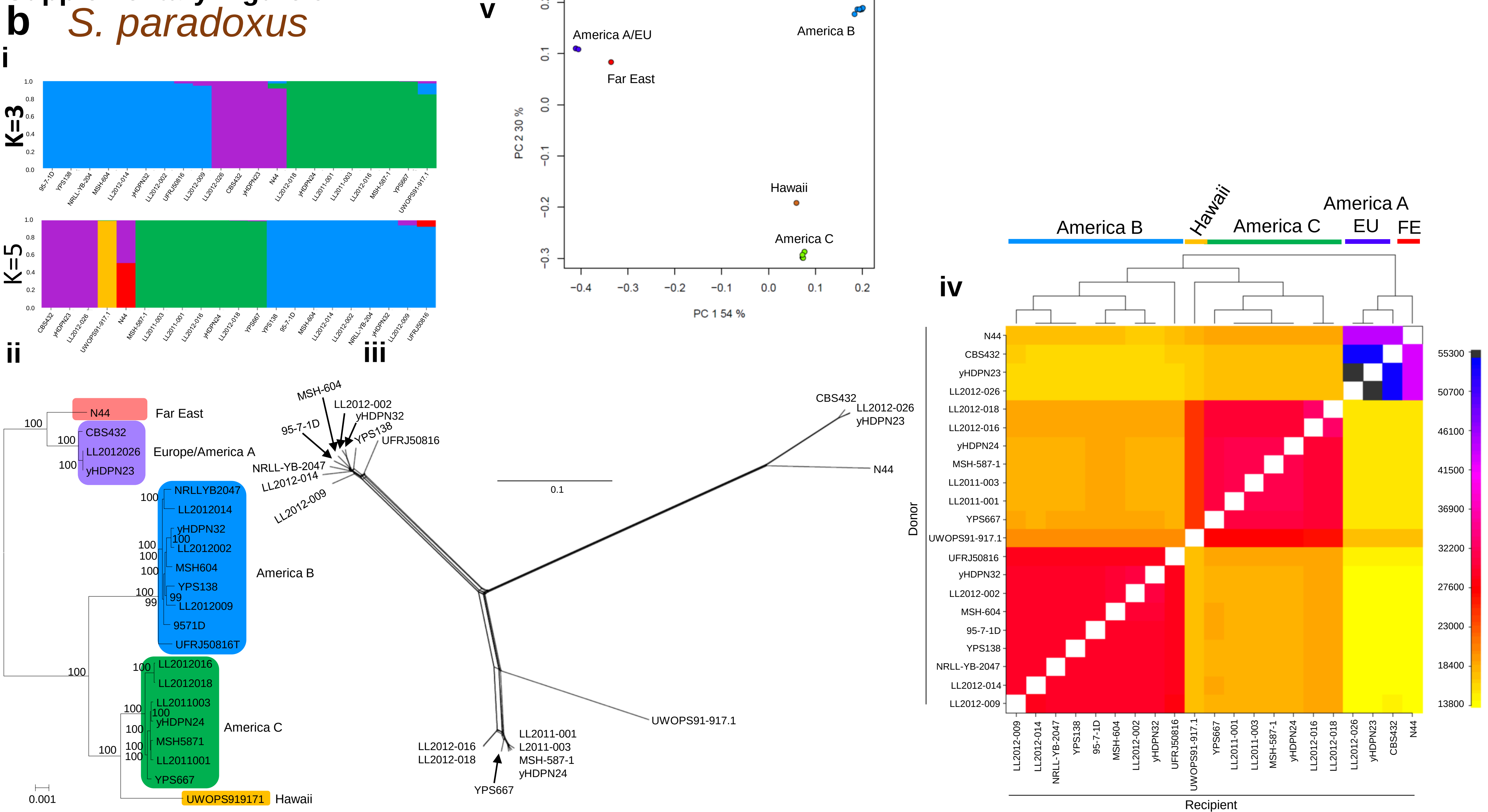

Supplementary Figure 9

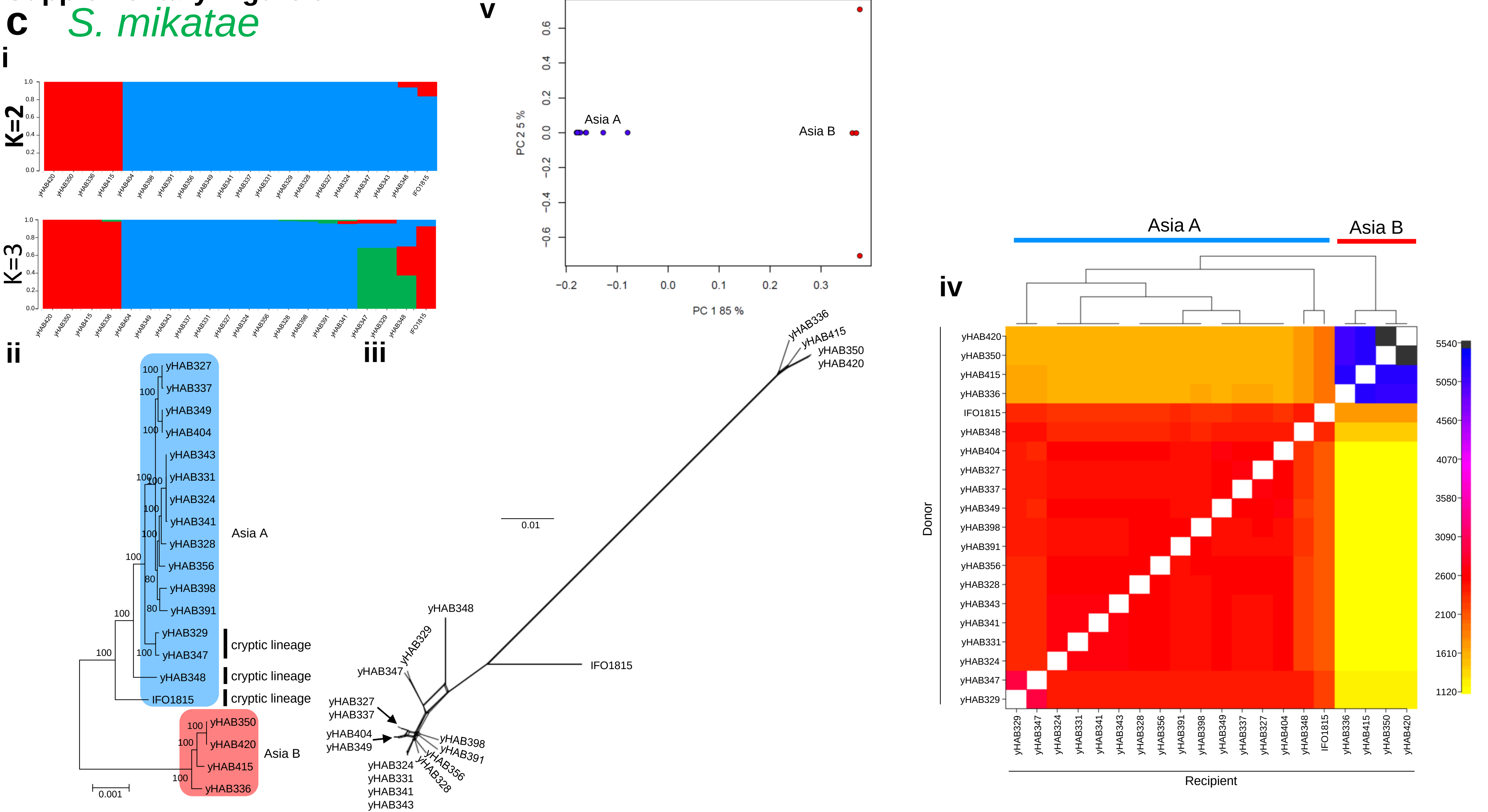

Supplementary Figure 9

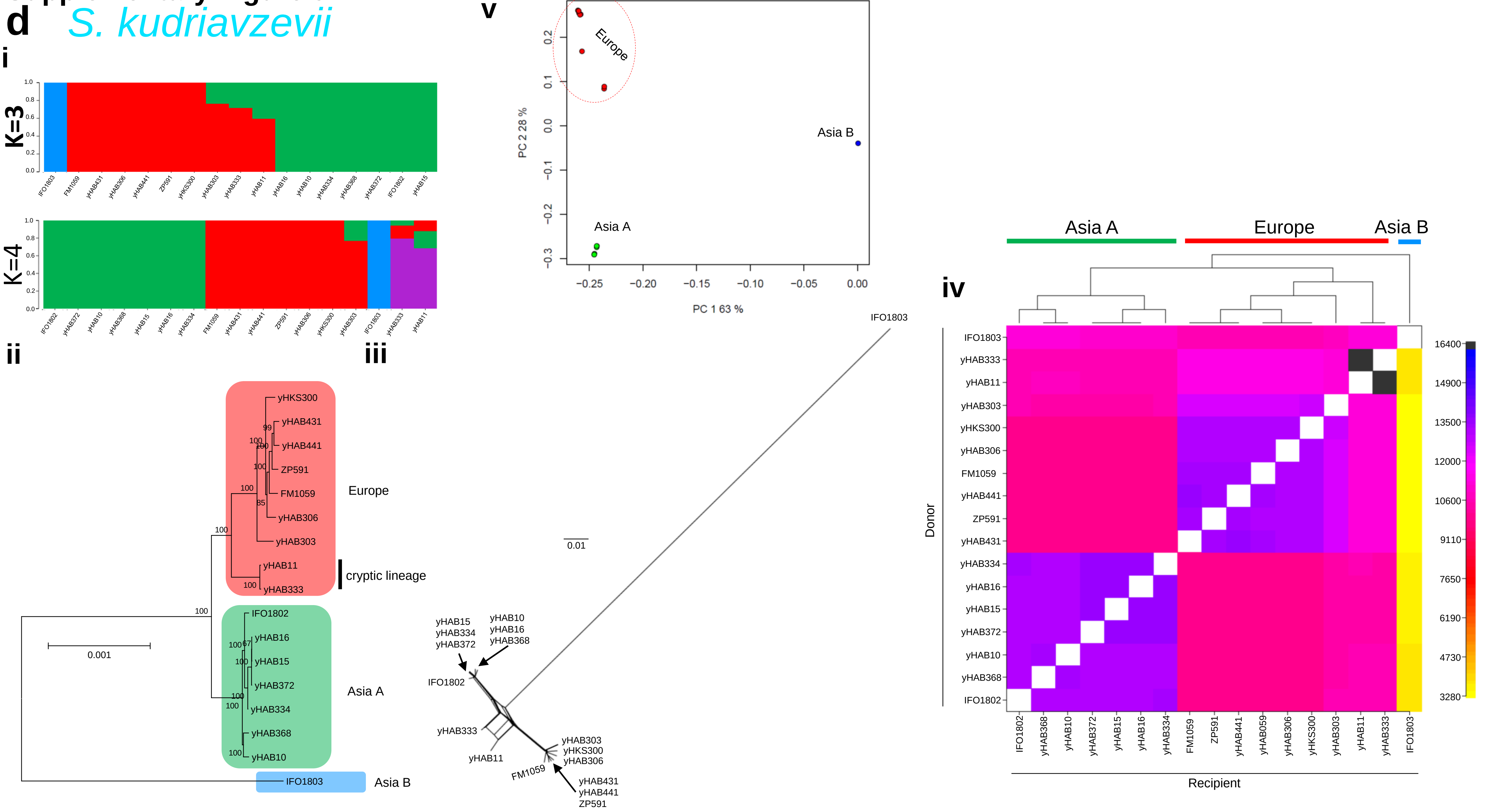

Supplementary Figure 9

e *S. arboricola*

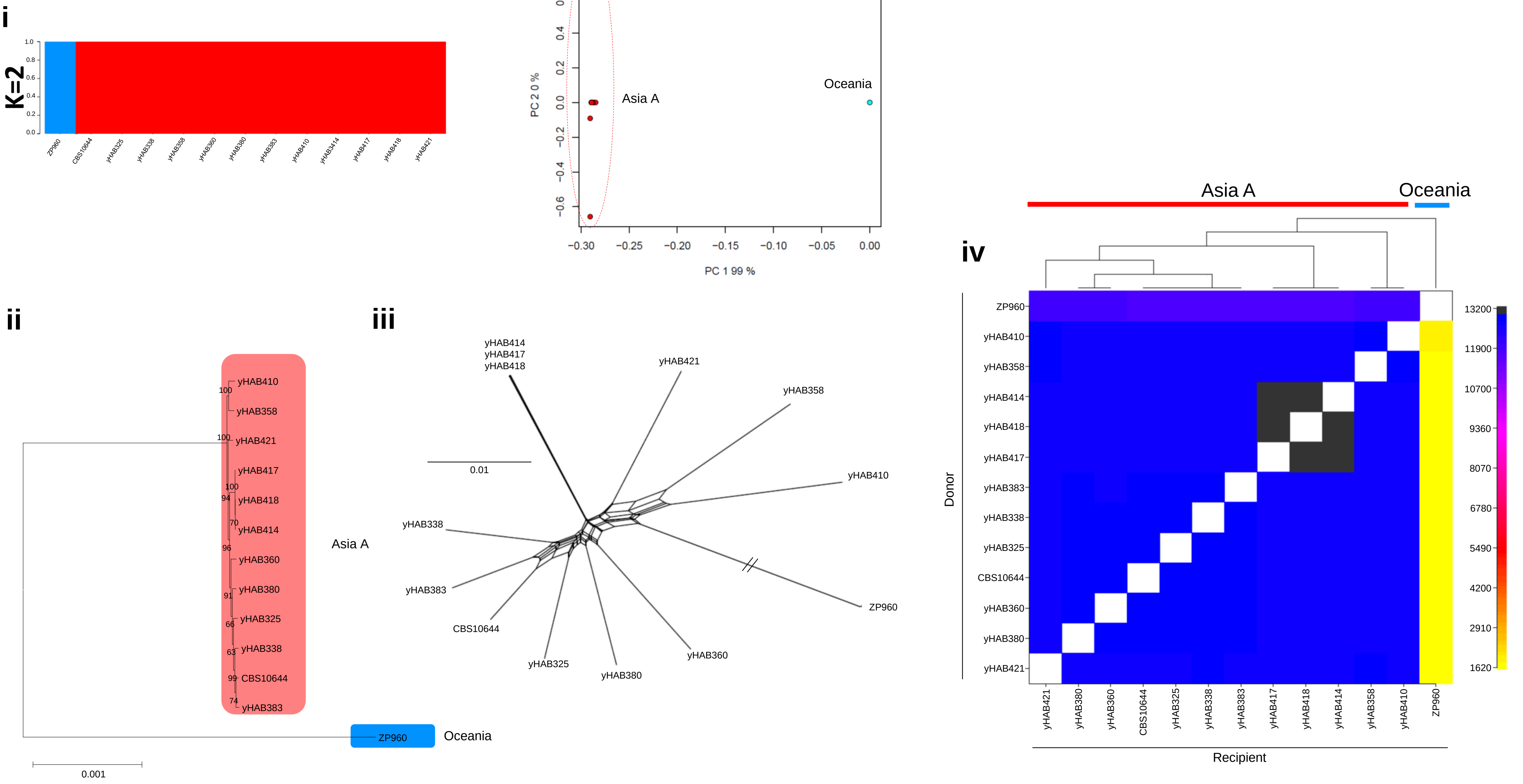

Supplementary Figure 9

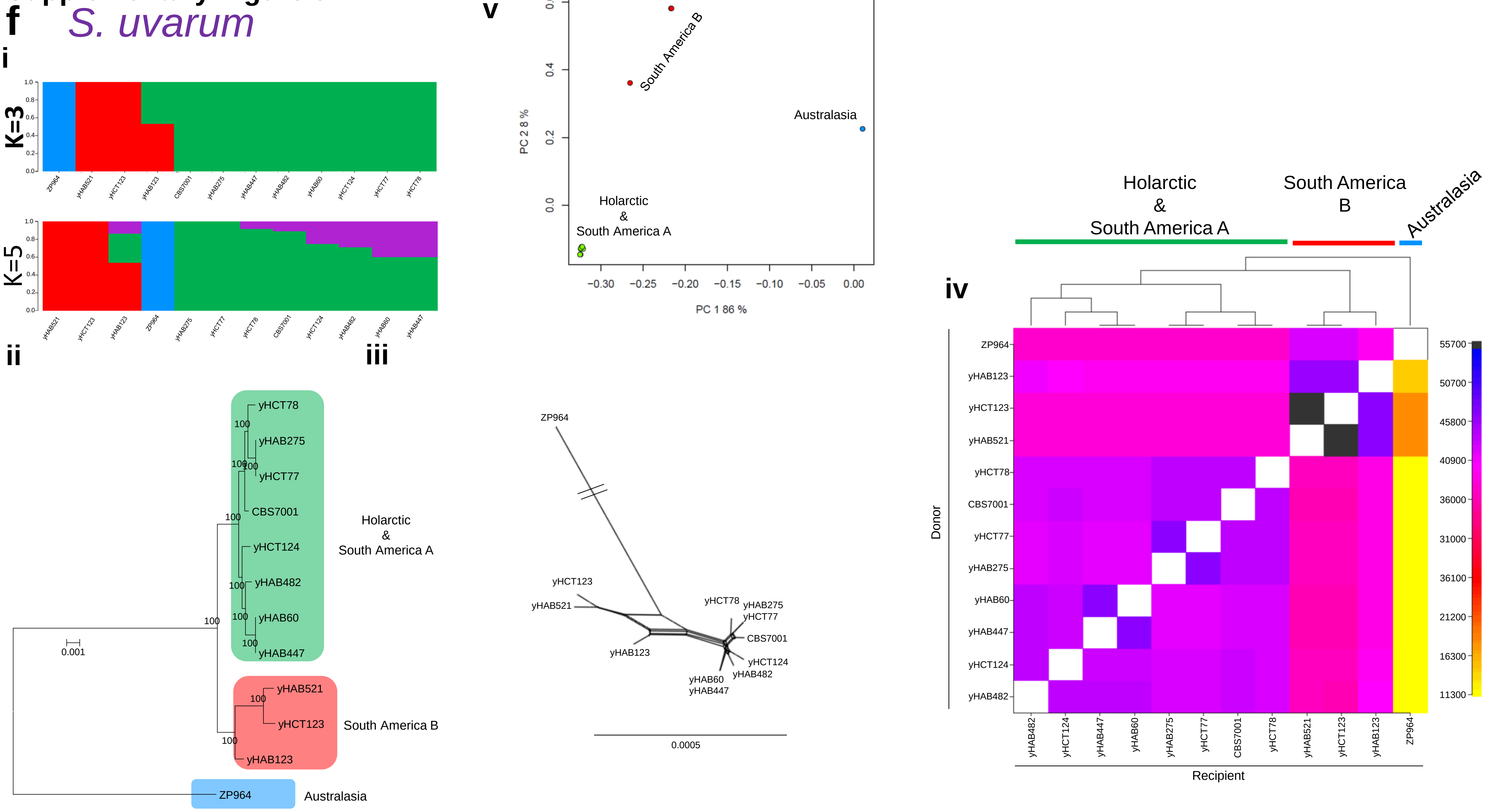

Supplementary Figure 9

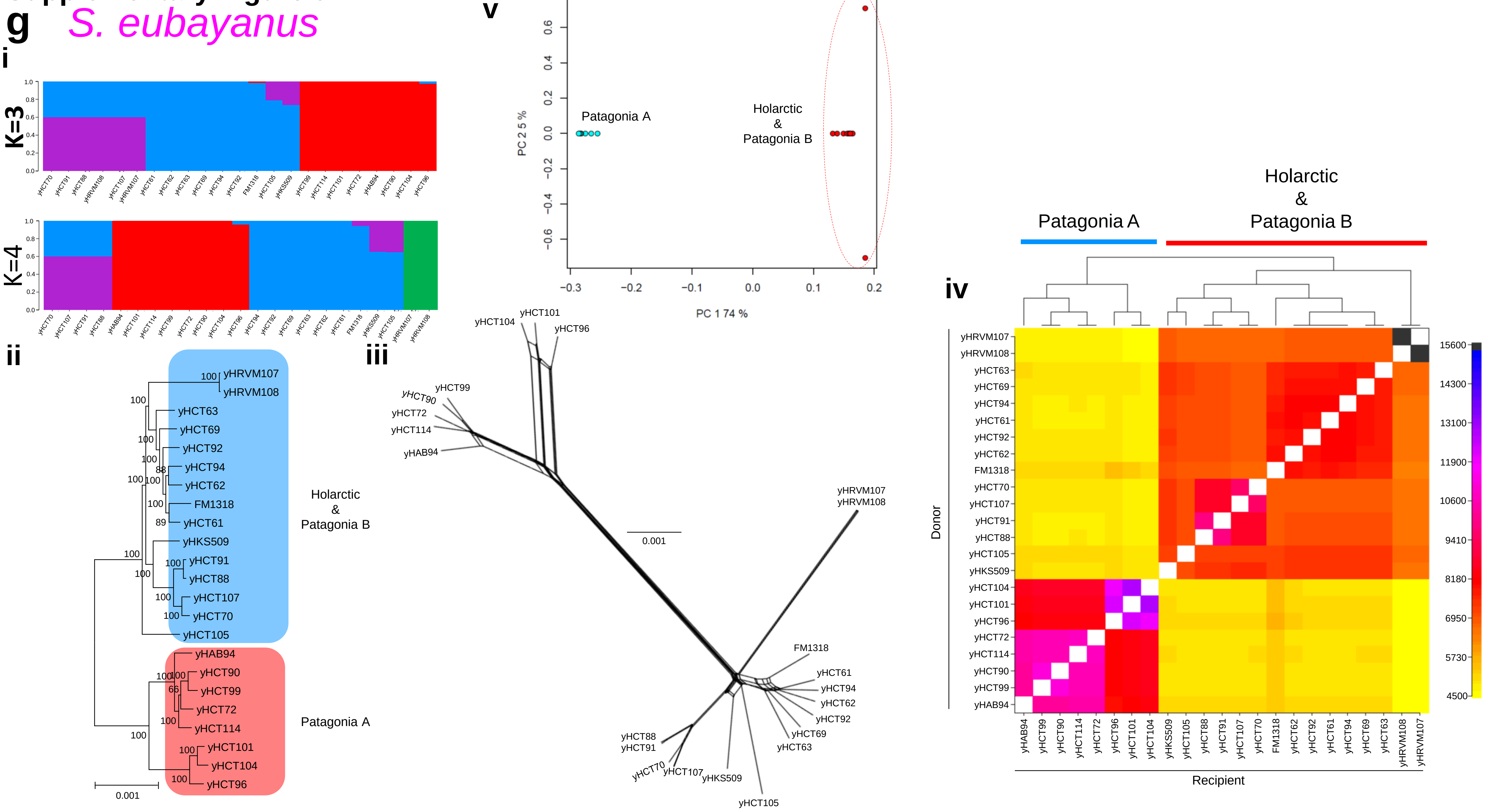

Supplementary Figure 9. Population genomics of seven *Saccharomyces* species.

Population genomic analyses for *S. cerevisiae* (n = 56), *S. paradoxus* (n = 21), *S. mikatae* (n = 20), *S. kudriavzevii* (n = 17), *S. arboricola* (n = 13), *S. uvarum* (n = 12), and *S. eubayanus* (n = 24) are shown in panels **a**), **b**), **c**), **d**), **e**), **f**), and **g**), respectively. **i**) Inference of the genetic clusters (*K*) and composition of individuals utilizing a random subsample of 10,000 SNPs in STRUCTURE. The most consistent number of genetic clusters/populations is highlighted in bold. Two summary plots for the most consistent number of genetic clusters/populations, from five independent runs are shown, except for *S. arboricola* because increasing K gave the same membership plot. Each color in the bar plots represents the cluster membership coefficients. The presence of several colors in the same strain suggests admixture. **ii**) Maximum likelihood phylogenetic tree reconstructed using all SNPs and corrected for invariant sites (see Online Material and Methods). The scale bars show the number of substitutions per site. Bootstrap values above 75 are reported at their corresponding nodes. Colored boxes enclose strains by their lineage designation. **iii**) Neighbor-Net phylogenetic network reconstructed with the SNP dataset. Incongruent data are represented by nodes subtended by multiple edges. Parallel lines in panels **a**, **e**, and **f** denote that the branch length is much longer but was shortened to fit the phylonetwork on the panel. **iv**) Coancestry heatmap. The coancestry units give measures of ancestry sharing. Darker colors indicate higher coancestry between strains. Briefly, each coancestry value measures the number of chromosomal ‘chunks’ given from a donor genome (column, x-axis) to a recipient genome (row, y-axis). Colored bars indicate populations. In *S. cerevisiae*, for simplification, populations

are described on the top tree branches, and larger groups were described in the bars. The *S. cerevisiae* groups are described according to the PCA in panel **v**. **v**) Principal Component Analysis (PCA) plots of PC1 versus PC2. The PCA is built using a normalized version of the coancestry matrix. The percent of the variance accounted for by each component is indicated. For *S. cerevisiae*, due to the low genetic diversity among domesticated strains and their close relatives, we reanalyzed the dataset by removing CHN I, CHN II, CHN IX, and the Taiwanese strains.

Supplementary Figure 10

a

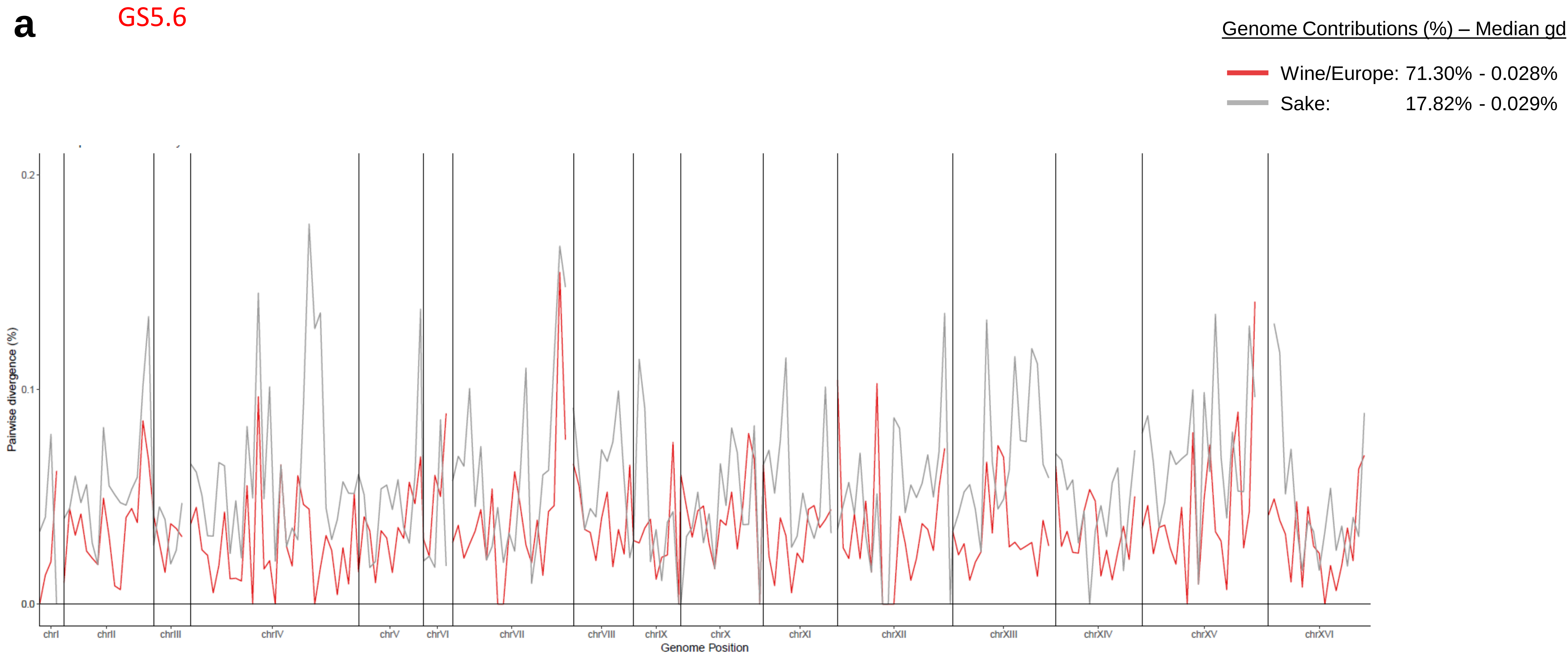

Supplementary Figure 10

b SK1

Genome Contributions (%) – Median gd

West Africa: 74.63% - 0.000%

Sake: 23.12% - 0.081%

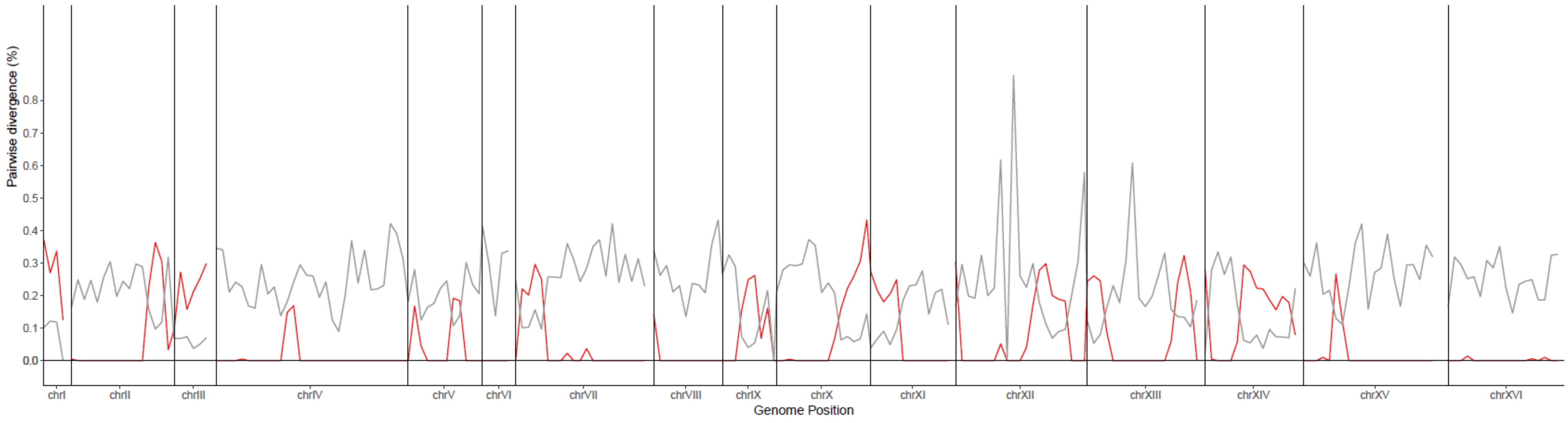

Supplementary Figure 10

c Y55

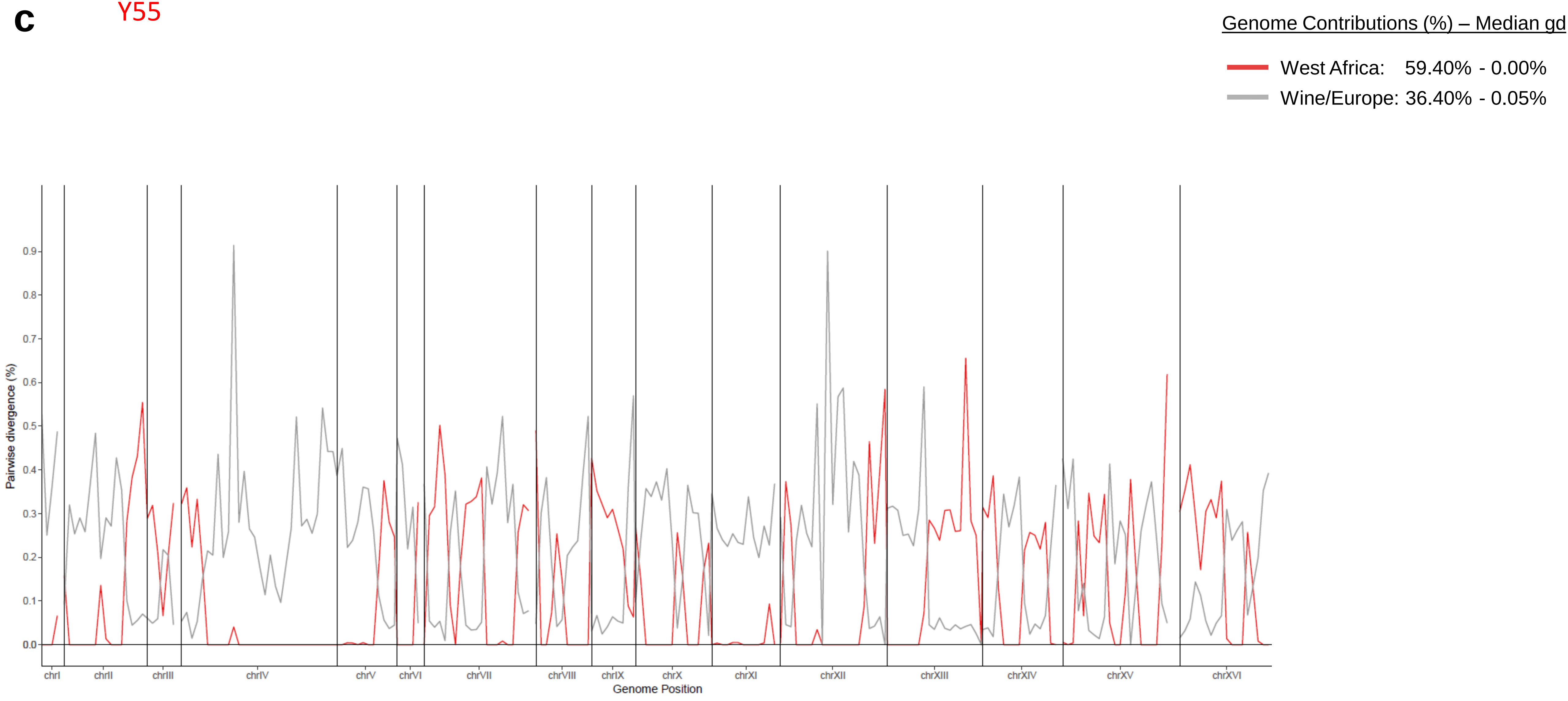

Supplementary Figure 10

d

YJM1083

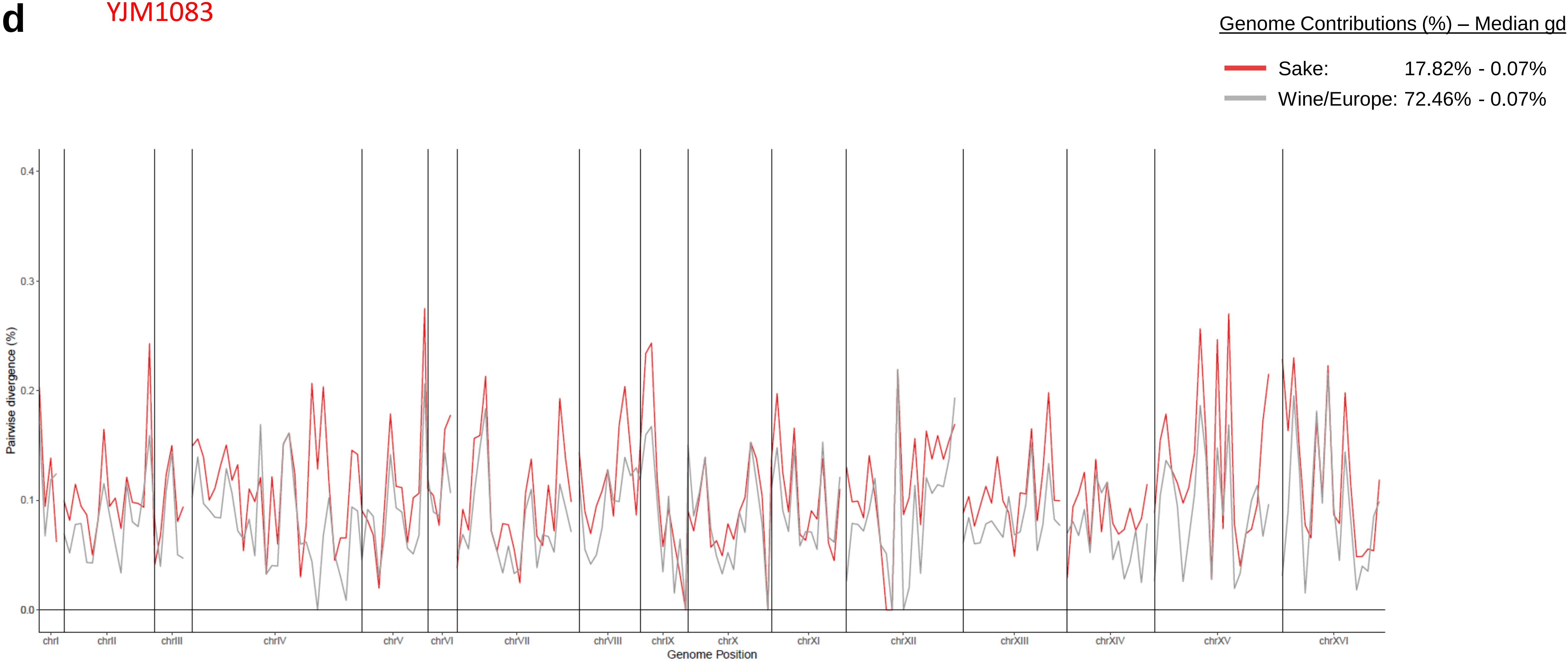

Supplementary Figure 10

e WLP380

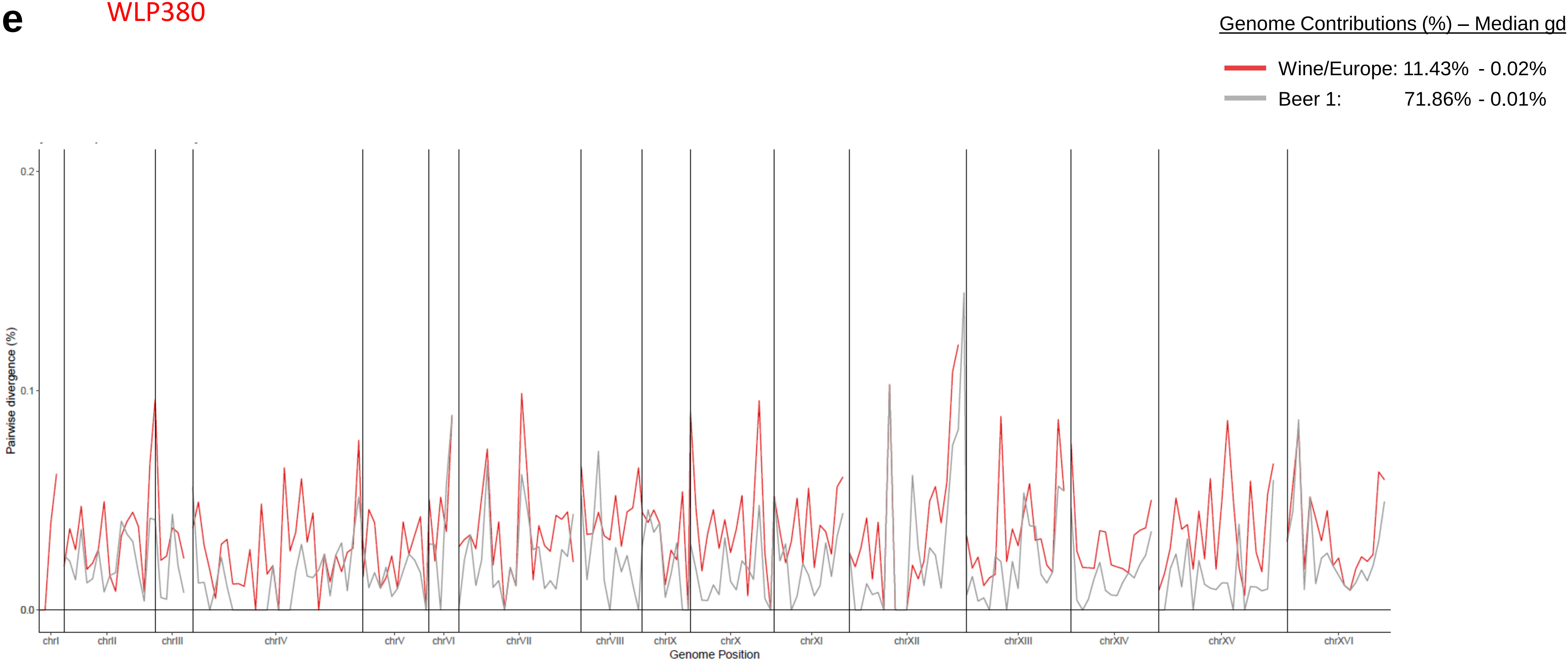

Supplementary Figure 10

f Wy1318

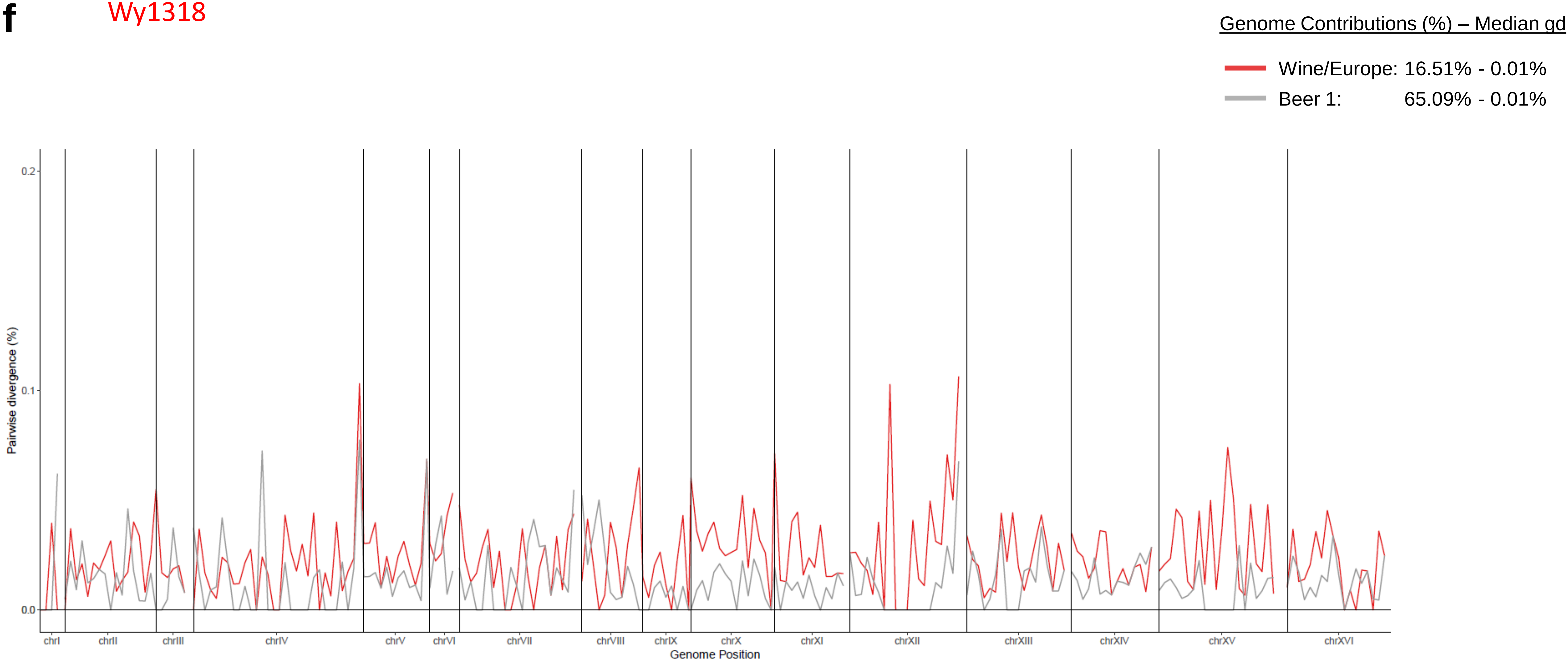

Supplementary Figure 10

g LL2012-016/LL2012-018

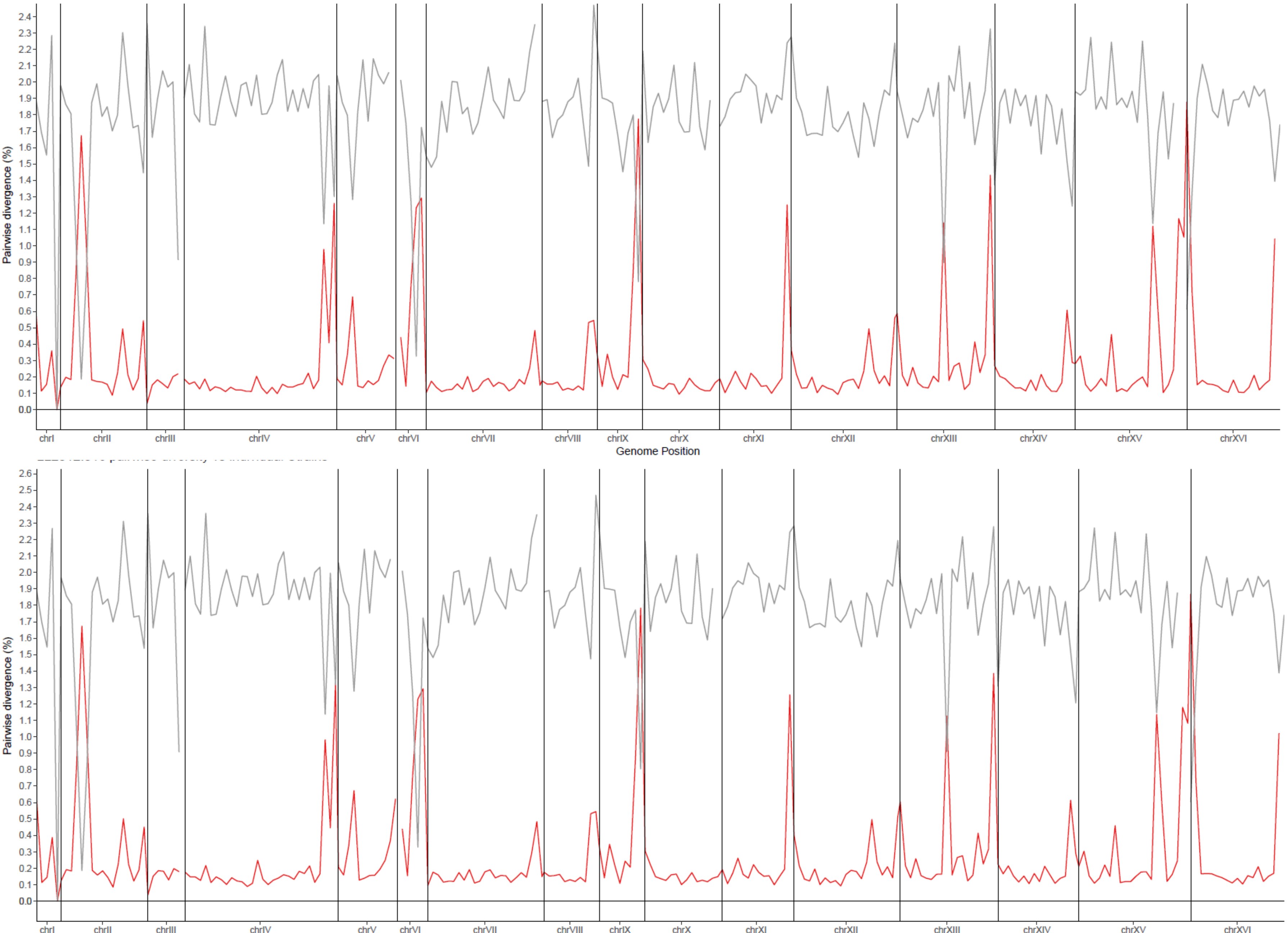

<u>Genome Contributions (%) – Median gd</u>

America C: 97.17% - 0.16%

America B: 2.46% - 0.78%

Supplementary Figure 10

h i) yHAB11/yHAB333

Genome Contributions (%) – Median gd

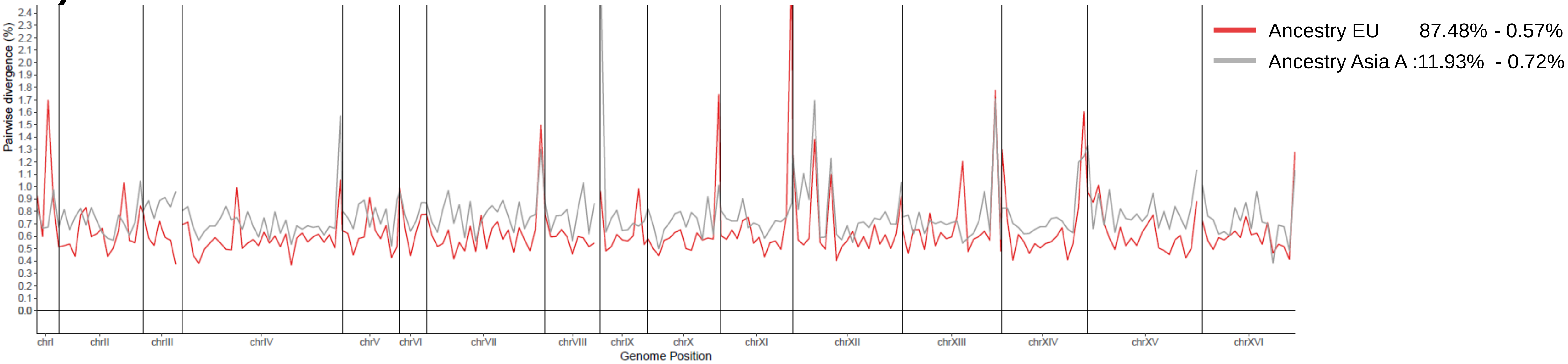

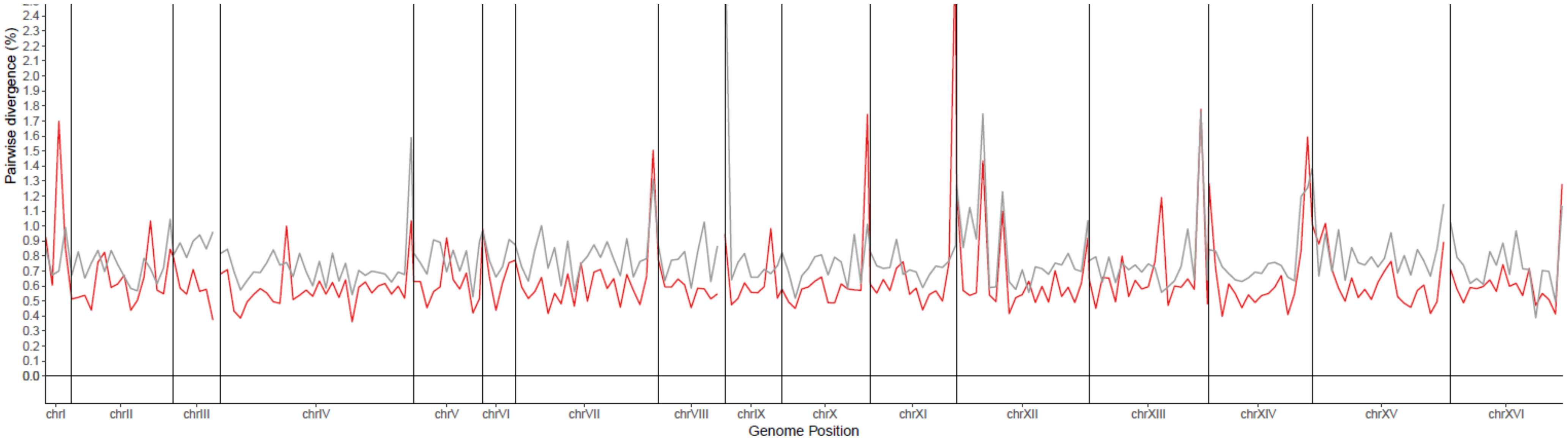

Supplementary Figure 10

5 Kbp windows

h ii) yHAB11/yHAB333

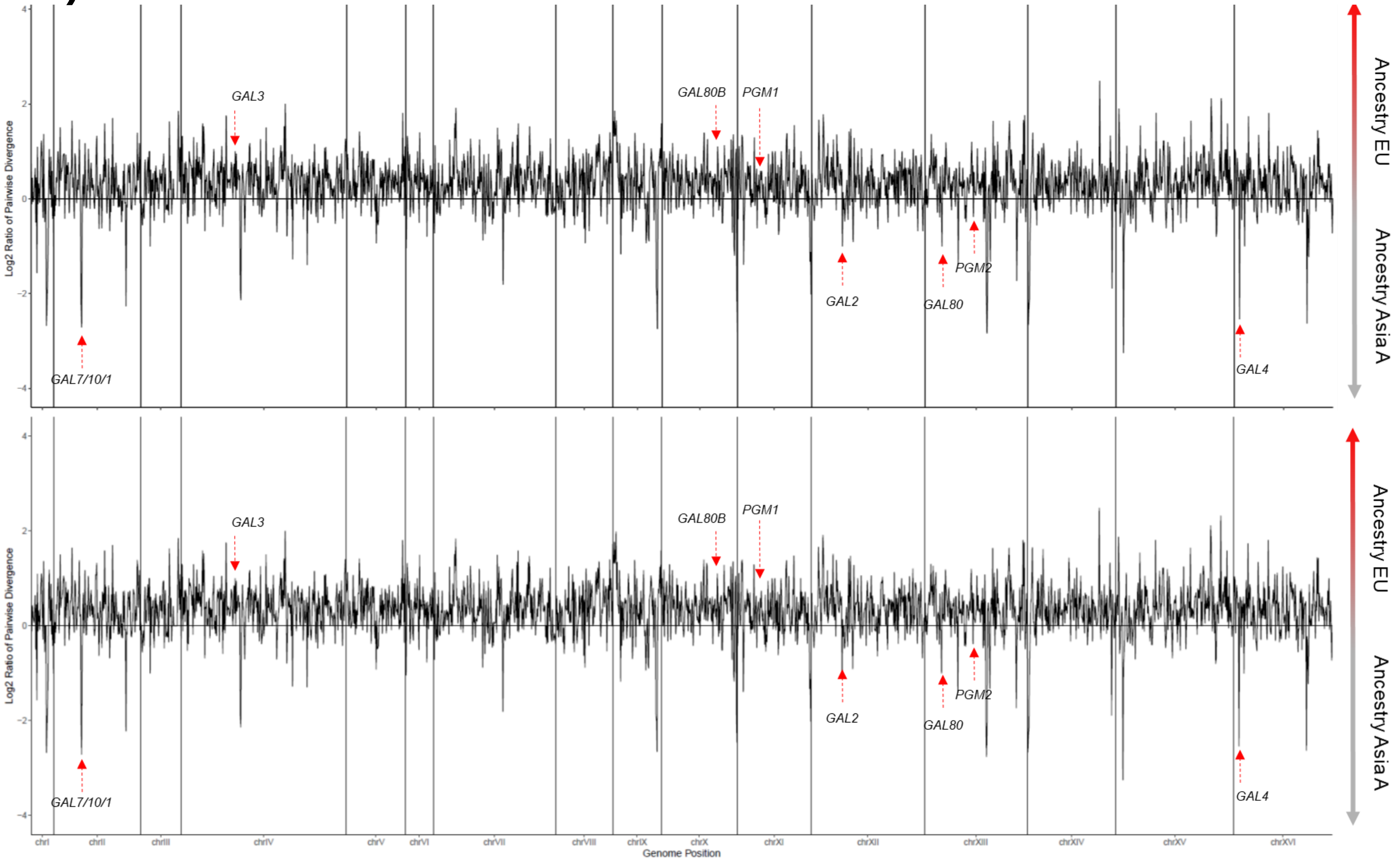

Supplementary Figure 10

i yHAB123

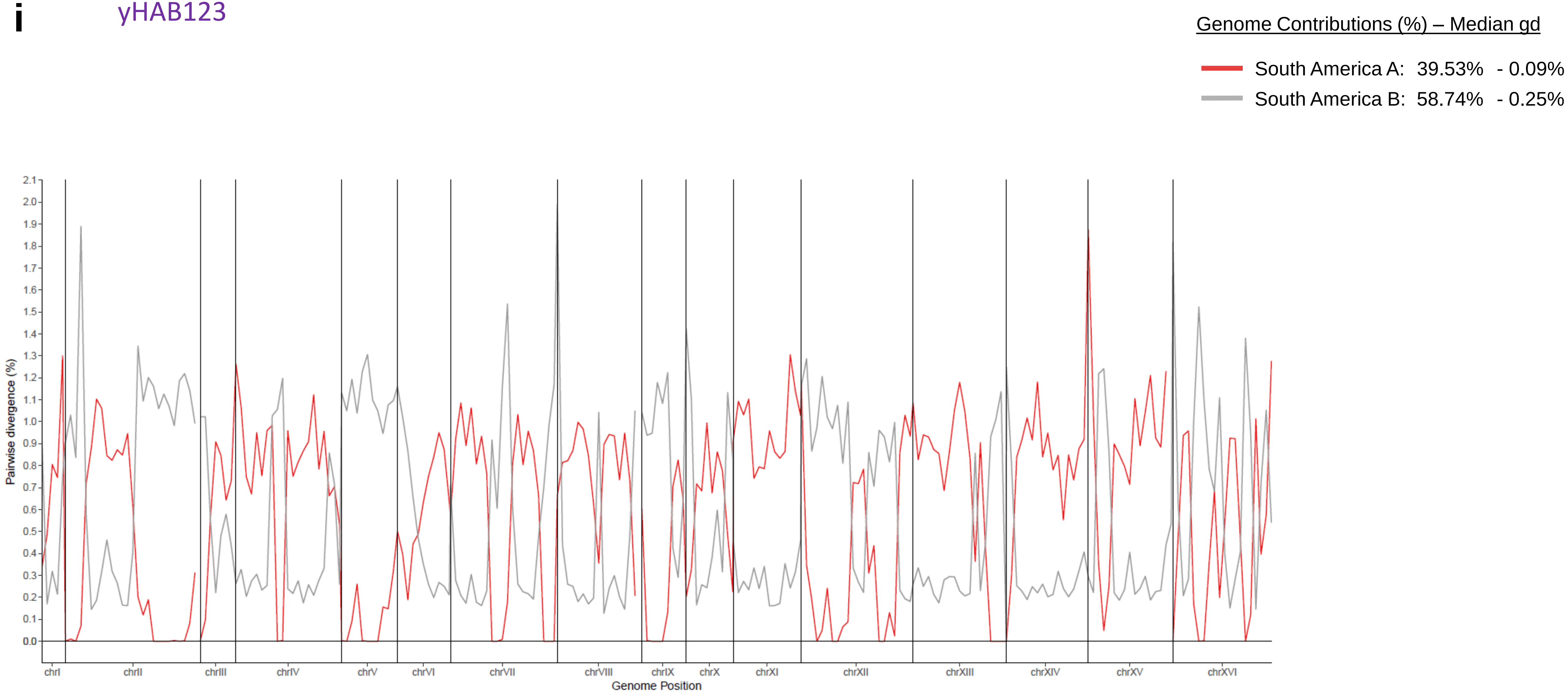

Supplementary Figure 10

j yHAB482

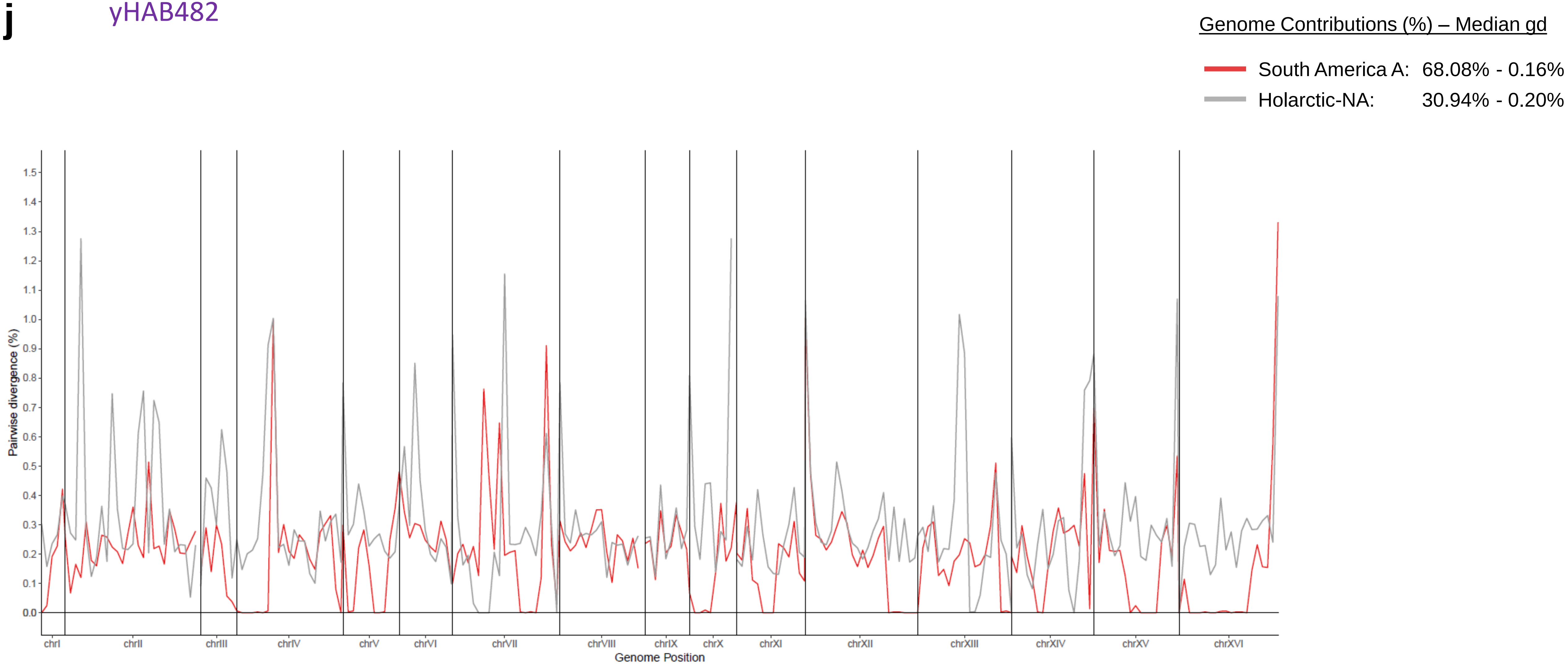

Supplementary Figure 10

k FM1318

Genome Contributions (%) – Median gd

Patagonia B: 89.42% - 0.08%

Patagonia A: 2.46% - 0.12%

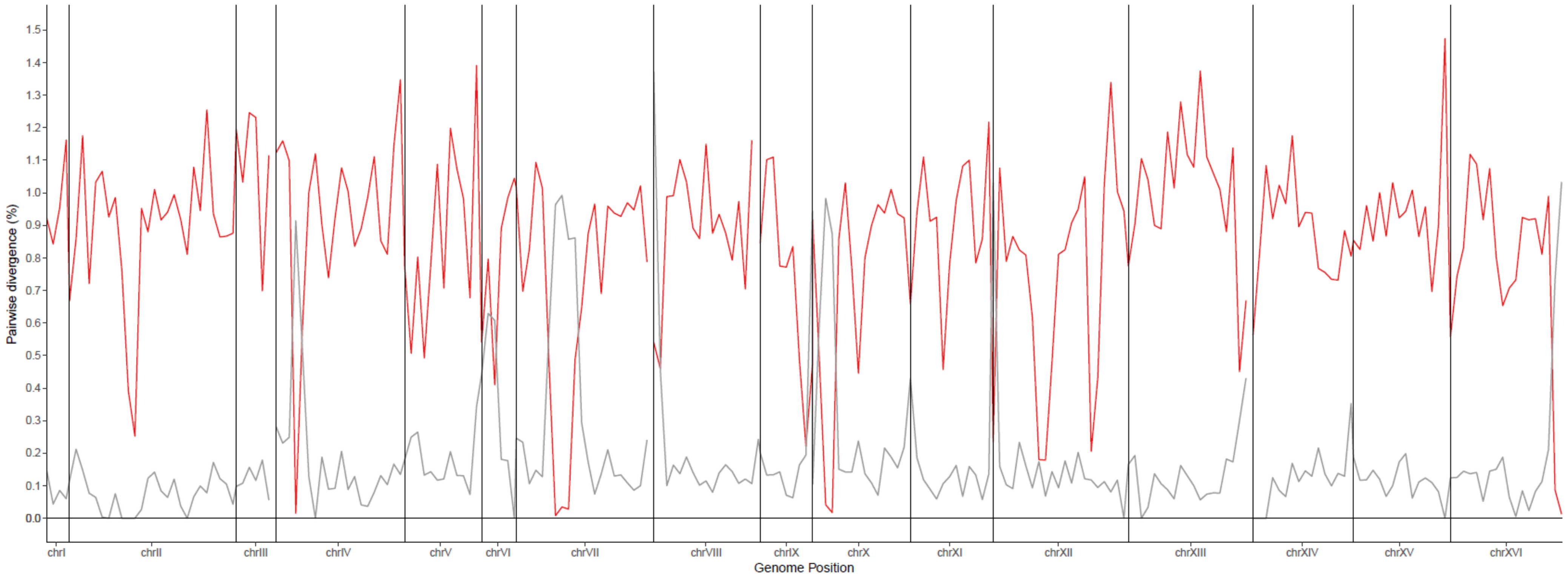

Supplementary Figure 10

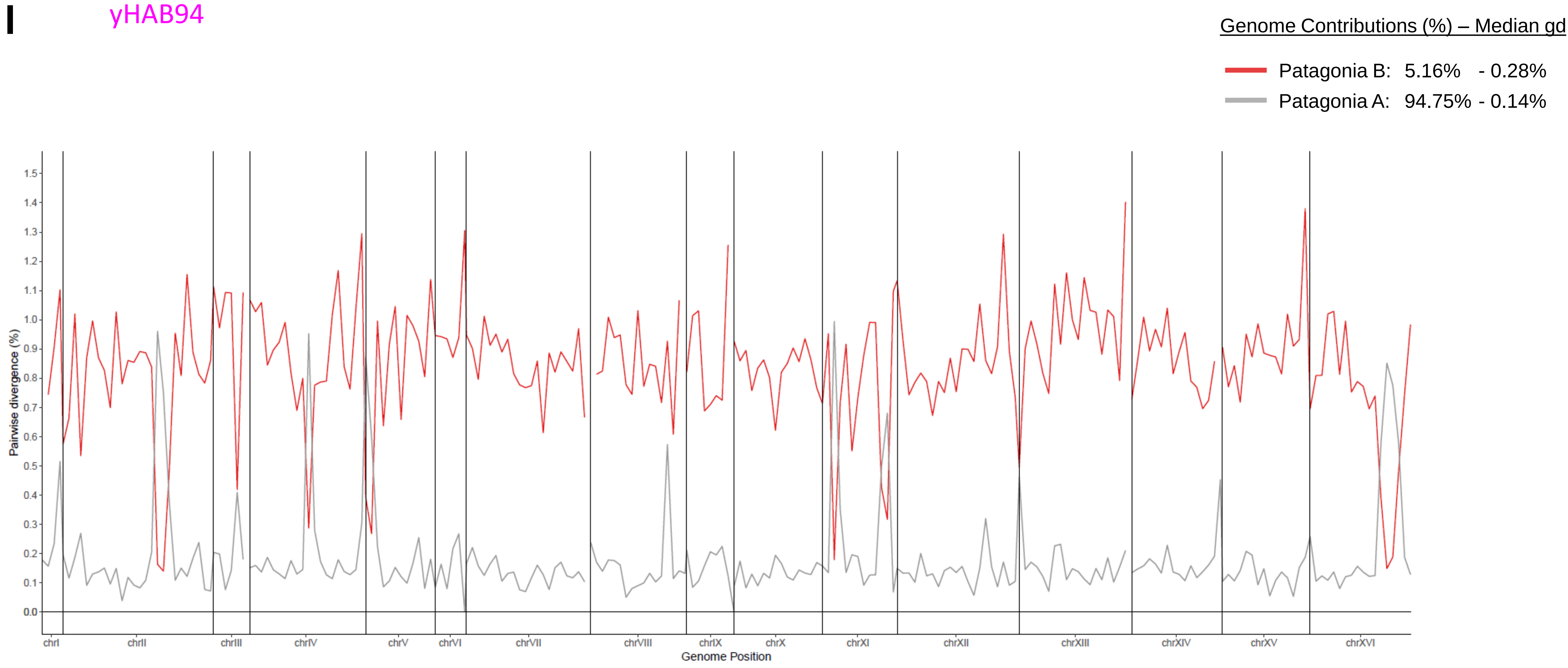

Supplementary Figure 10

m yHCT101

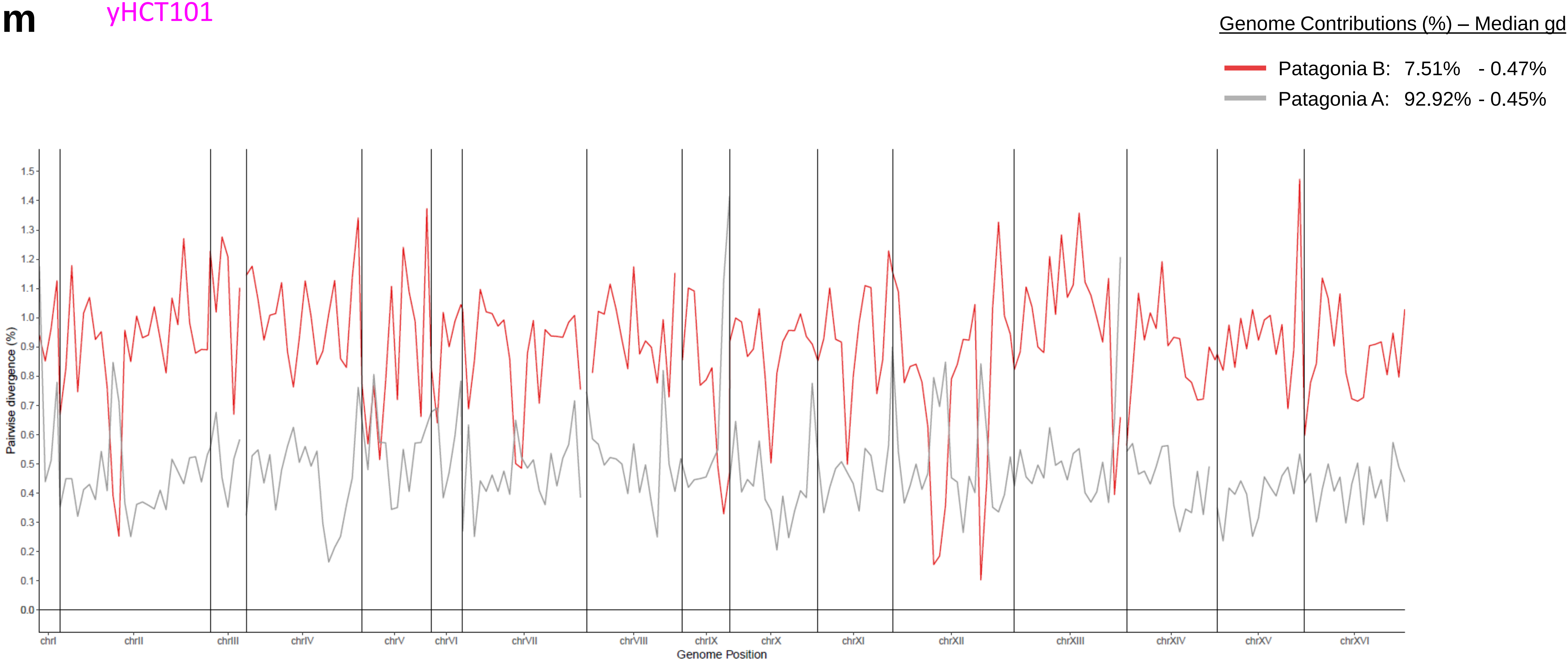

Supplementary Figure 10

n yHCT104

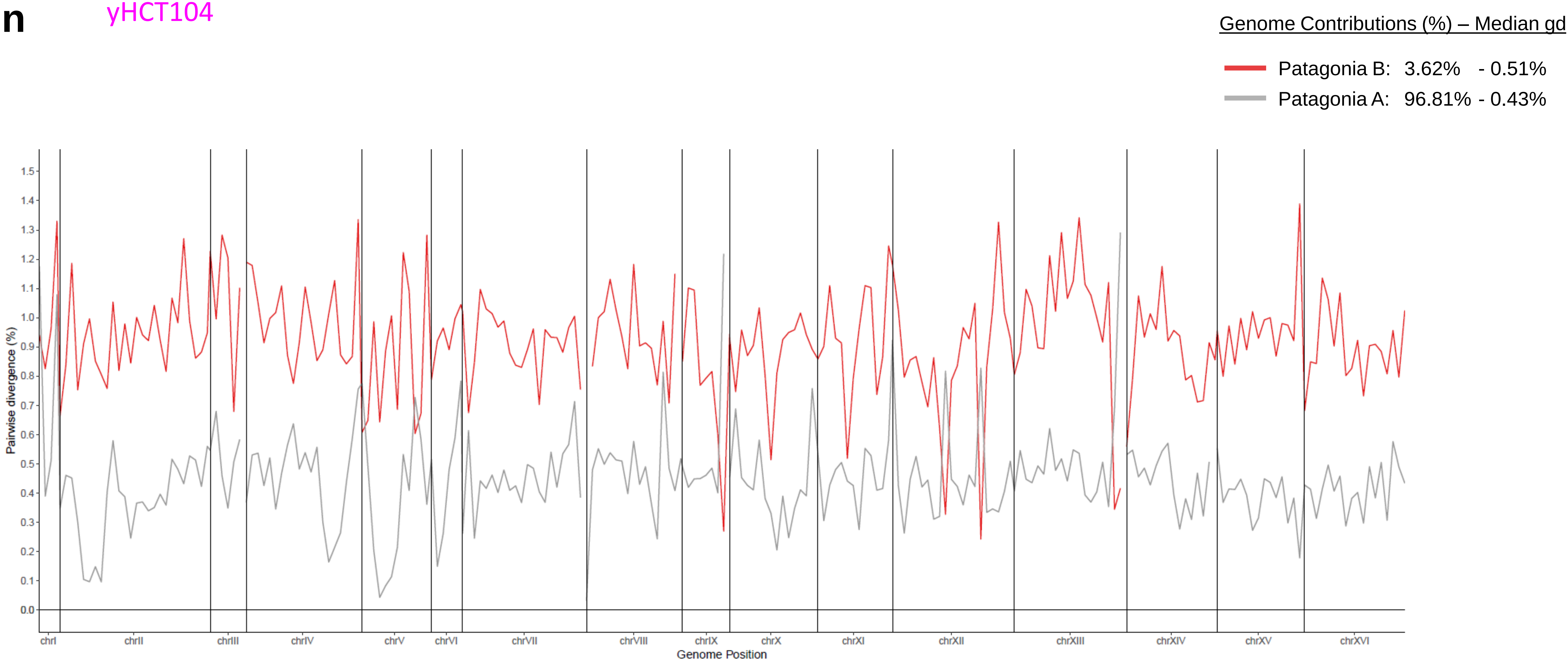

Supplementary Figure 10

O yHCT96

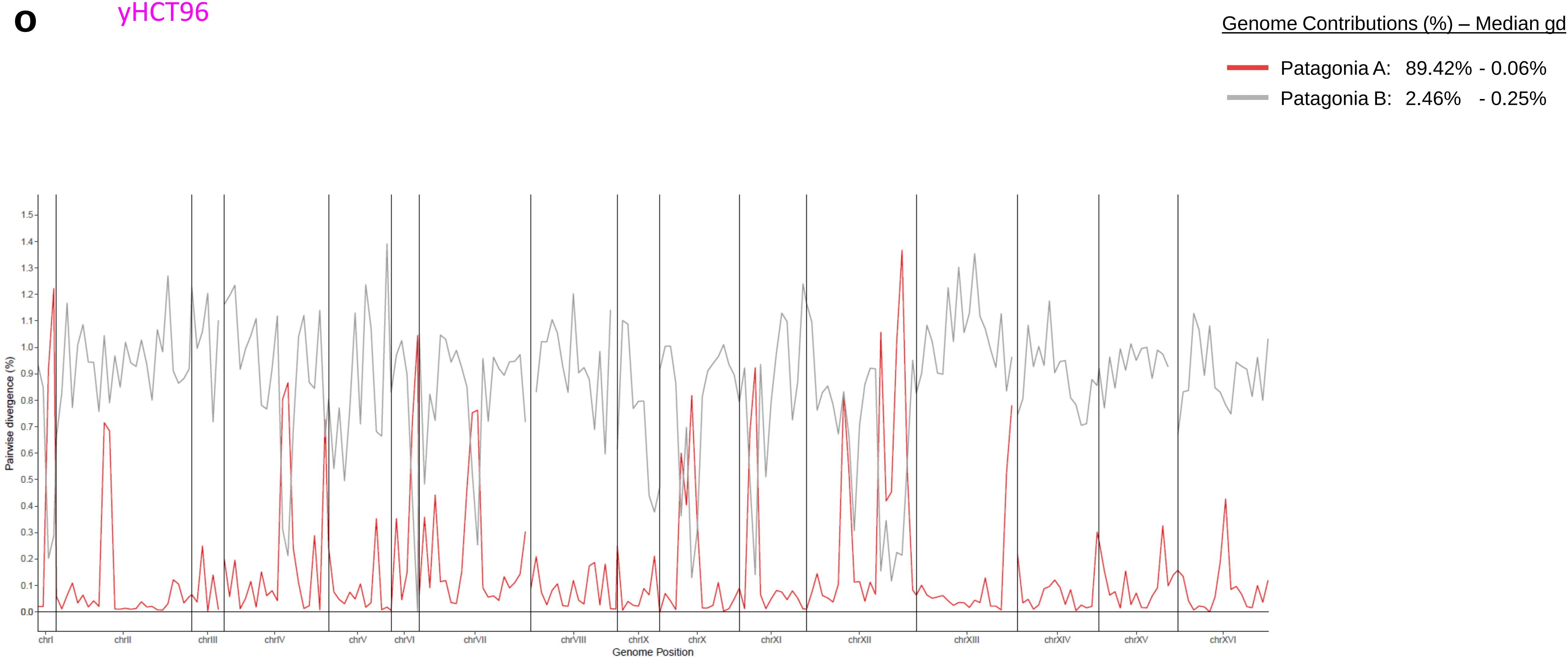

Supplementary Figure 10. Genome-wide pairwise nucleotide sequence divergence plots for admixture *Saccharomyces* strains.

Pairwise nucleotide divergence comparisons for admixture strains of five different *Saccharomyces* species compared to representative strains from the potential donor population are shown in panels **a-h i)**,**i-o**. The percentage of pairwise divergence for 50-kbp windows is shown on the y-axis. Roman numerals represent chromosomes. Strain names are colored according to their species designations. Panel **h ii)** represents the $\log_2$ divergence ratio of the pairwise nucleotide divergence comparisons calculated in panel **h i)**, but for 5-kbp windows. Ancestry is indicated by arrows at the right of the plot. In panel **h ii)**, we include the location of genes involved in galactose utilization (Figure 6a), whose phylogenetic tree reconstruction is displayed in Supplementary Figure 28i-k. Holarctic-NA: Holarctic-North America. gd: average genome-wide divergence of the portion of the genome contributed by that population.

Supplementary Figure 11

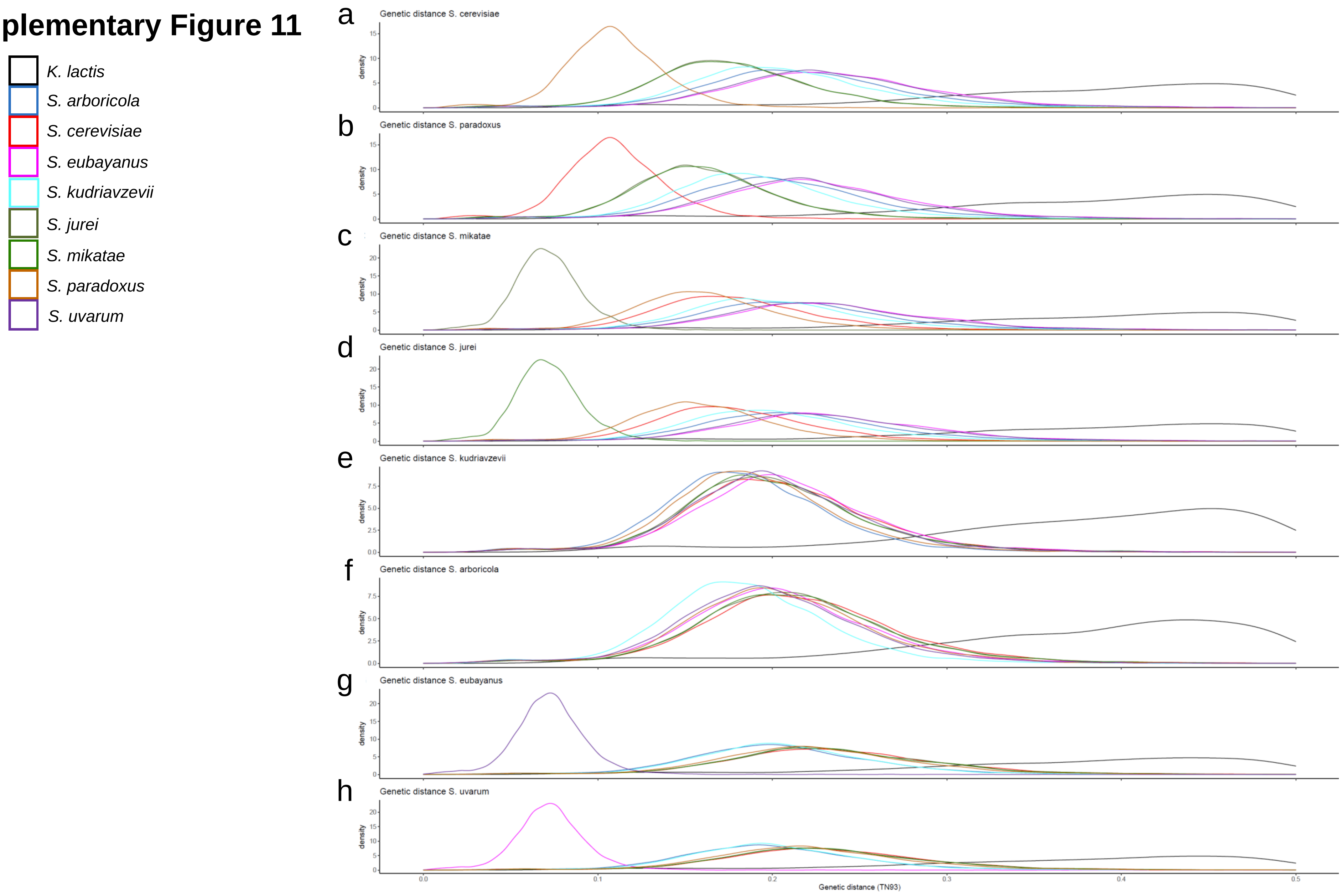

Supplementary Figure 11

i

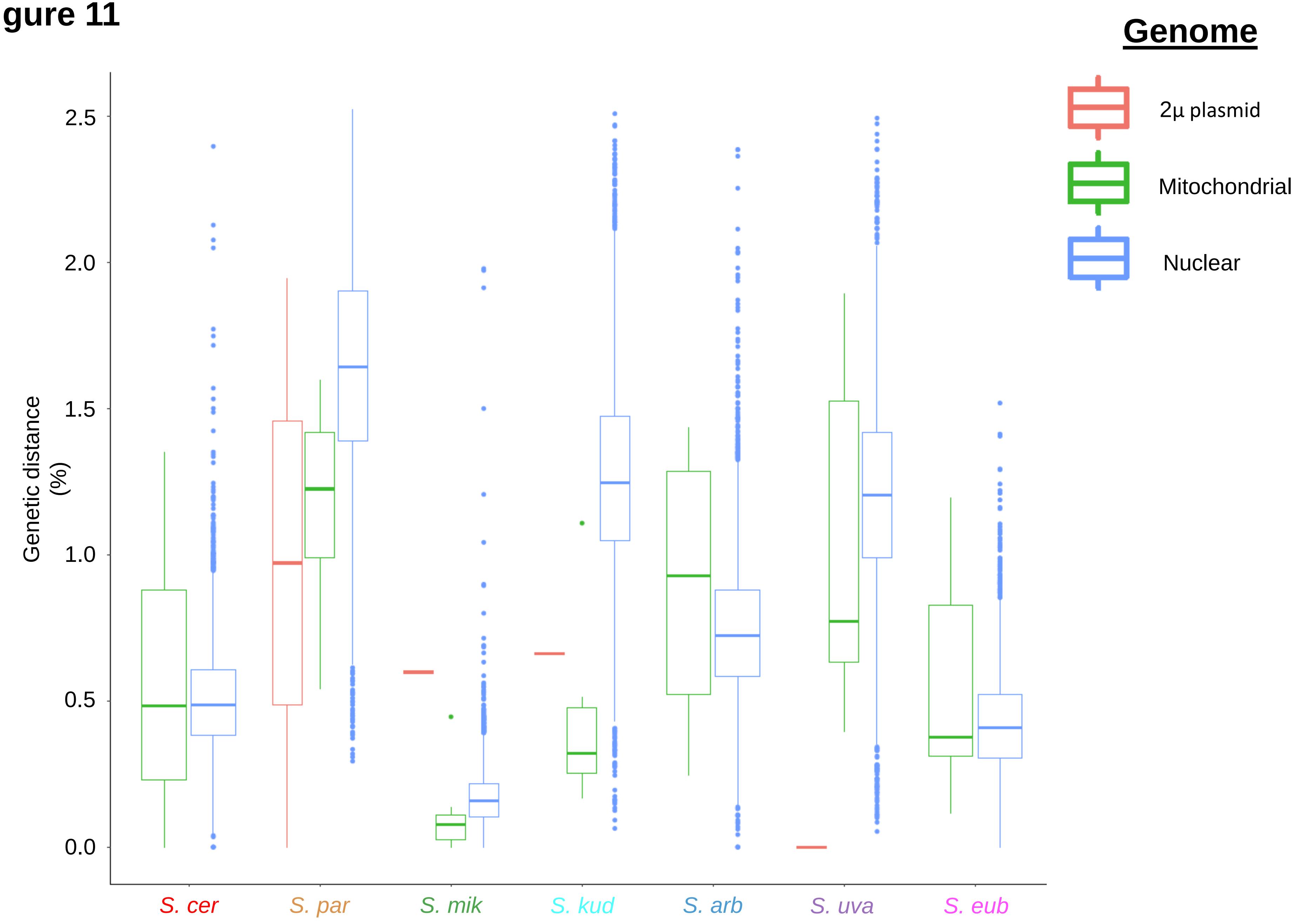

Supplementary Figure 11

j

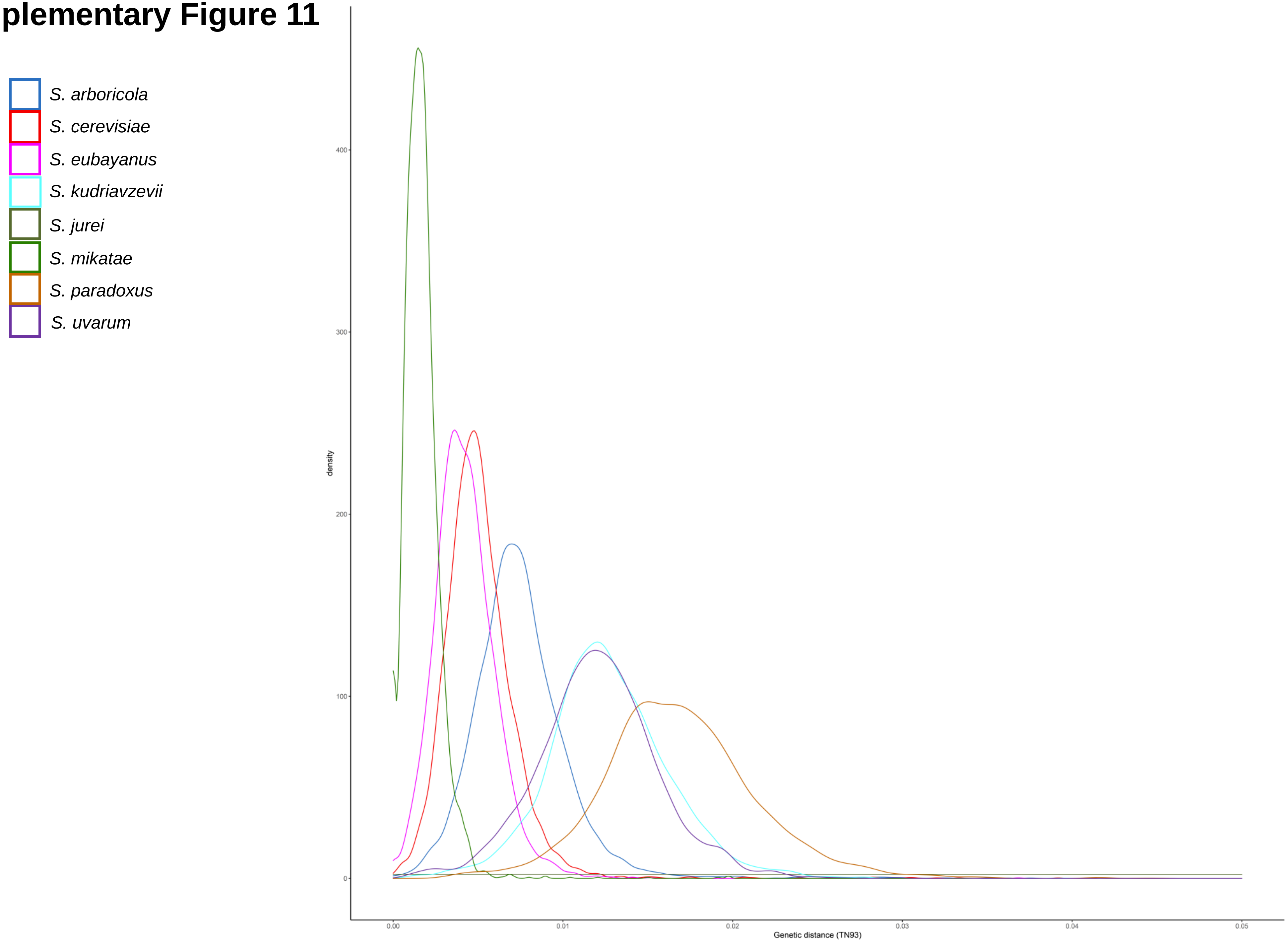

Supplementary Figure 11

k

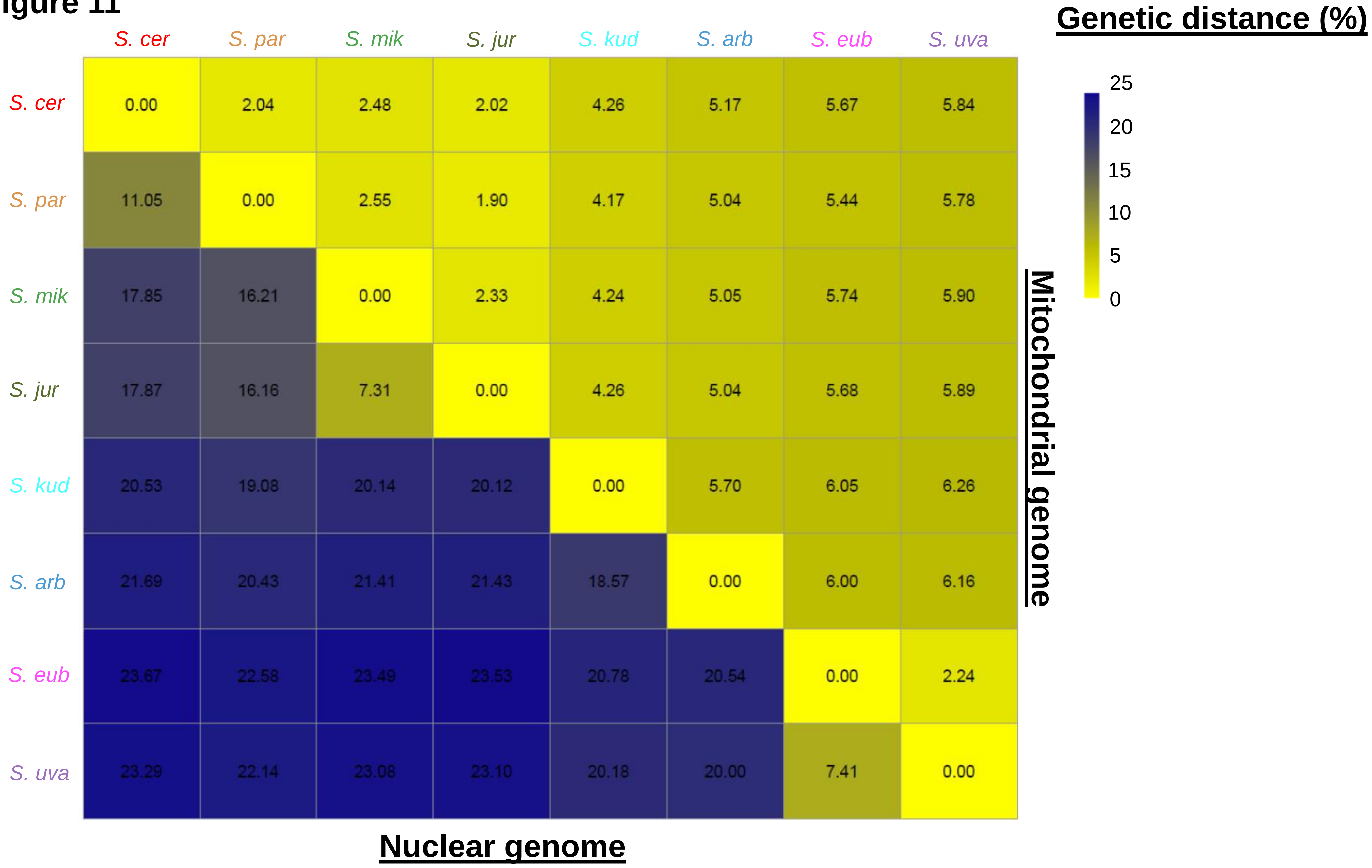

Supplementary Figure 11. Genetic distance distributions.

Tamura-Nei corrected genetic distance distribution for comparisons between species using all annotated `YGAP` orthologous genes (3858 genes). Each panel, from **a)** to **h)**, represents the comparison of a species against all other species with lines colored according to the species compared. Panel **i)** represents boxplots of the genetic distance within species for nuclear `YGAP` orthologous genes, mitochondrial genes used in Supplementary Figure 4, and 2-µm plasmid genes (*REP1* and *REP2*). Median values for mean genetic distances for each species genome are represented by a horizontal line inside the box, and the upper and lower whiskers represent the highest and lowest values of the 1.5 * IQR (inter-quartile range), respectively. Mean genetic distances for each gene (dots) above the 99% percentile are not displayed. Panel **j)** represents the genetic distance within a species with each line colored according to the legend. TN93: Tamura-Nei 1993 model correction. Panel **k)** represents the mean genetic distance between species for the nuclear (lower diagonal) and mitochondrial (upper diagonal) genes. *Saccharomyces* strains (n = 33) are indicated in Supplementary Data 1.

Supplementary Figure 12

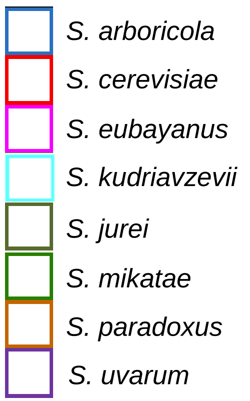

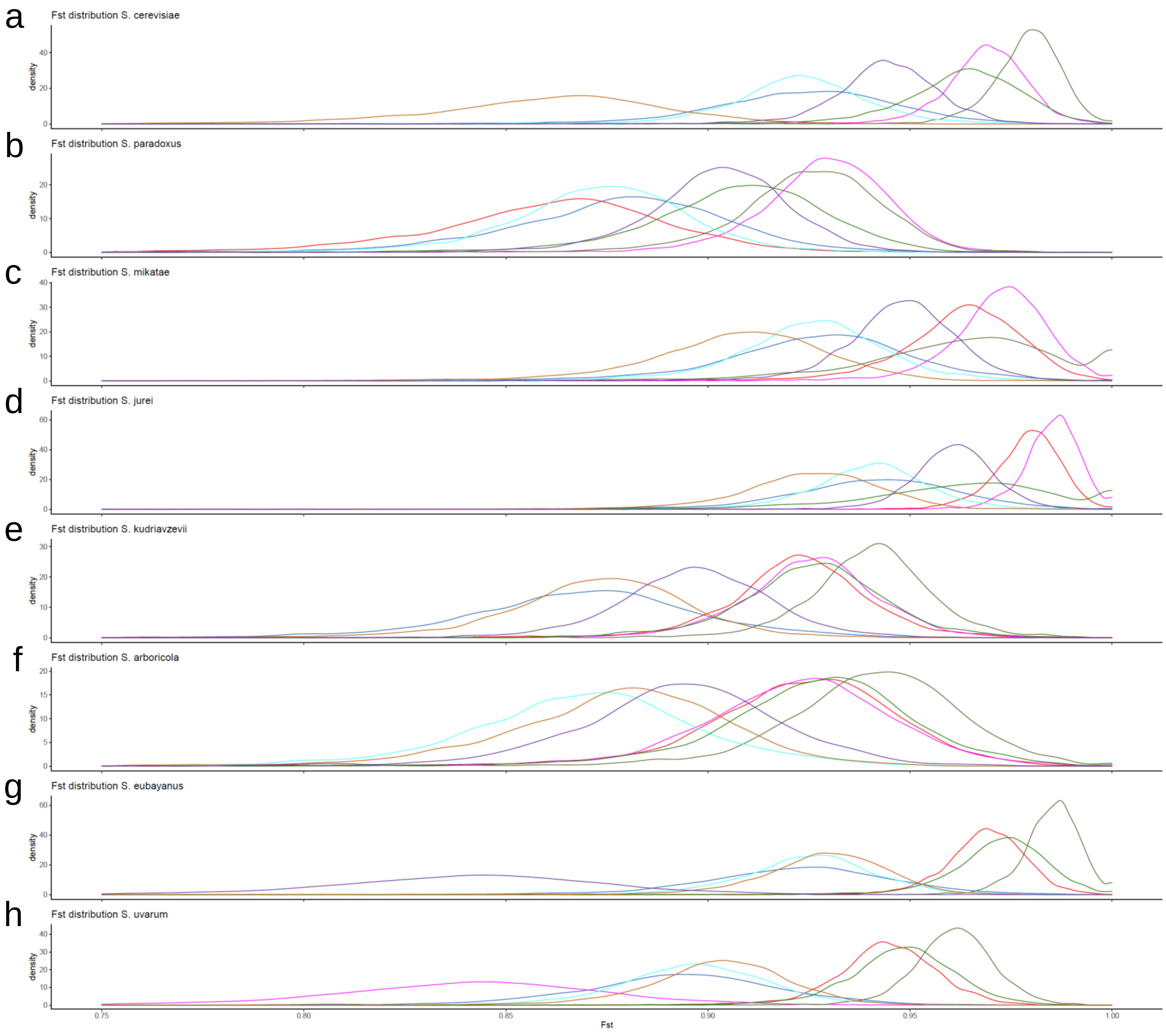

Supplementary Figure 12. Fst distributions.

Relative divergence (Fst) distribution for comparisons between species using all annotated YGAP orthologous genes (3858 genes). Each panel represents the comparison of a species against the other species with lines were colored according to the species compared. *Saccharomyces* strains (n = 33) are indicated in Supplementary Data 1. Fst distribution of *S. cerevisiae* compared to the rest of species is displayed in panel **a)**, *S. paradoxus* against the rest in panel **b)**, *S. mikatae* against the rest in panel **c)**, *S. jurei* against the rest in panel **d)**, *S. kudriavzevii* against the rest in panel **e)**, *S. arboricola* against the rest in panel **f)**, *S. eubayanus* against the rest in panel **g)**, and *S. uvarum* against the rest in panel **h)**.

Supplementary Figure 13

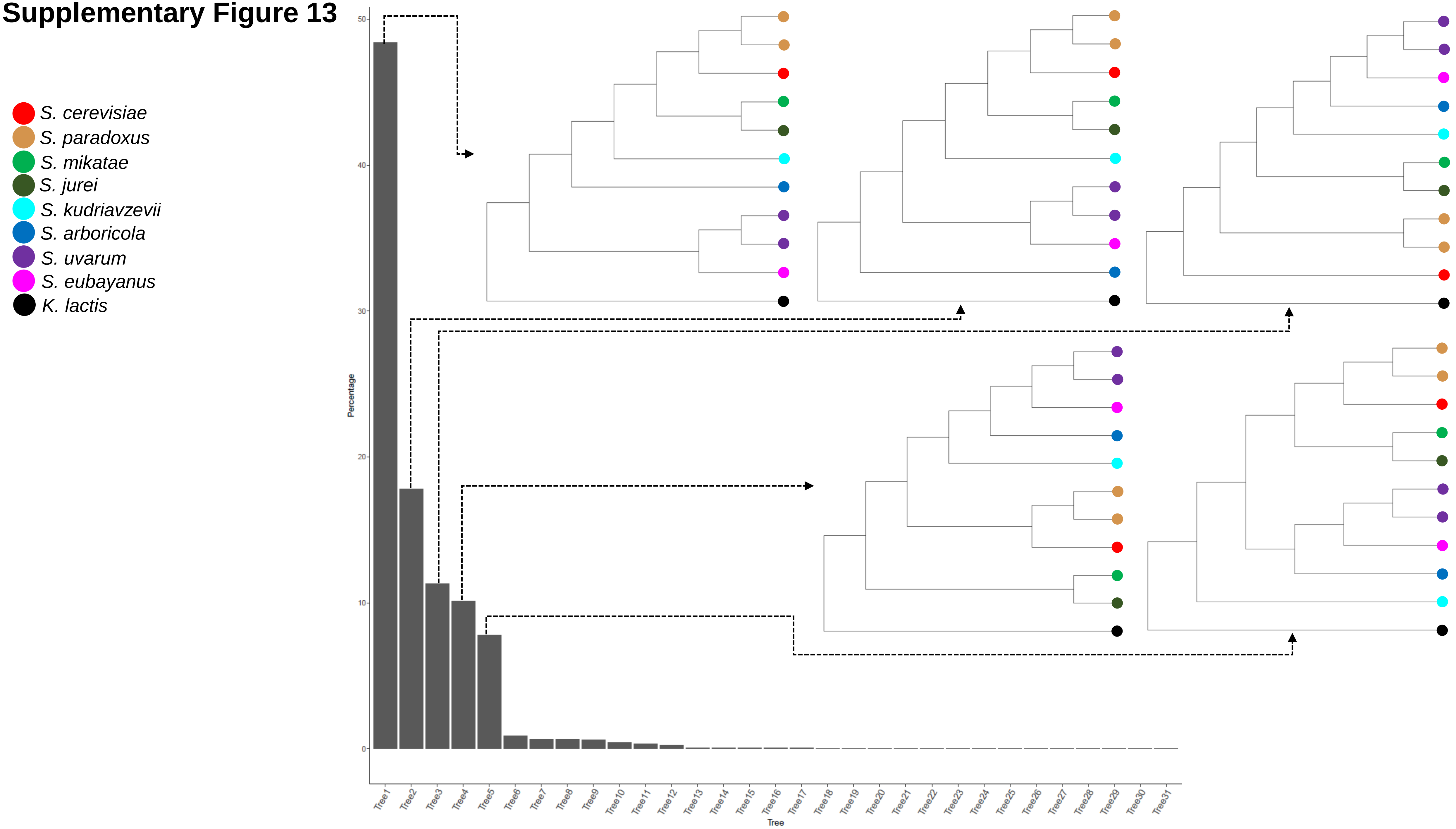

Supplementary Figure 13. BUCKy concordance primary tree and alternative topologies.

Distribution of the most probable Bayesian tree topologies for each `YGAP` orthologous gene (from a set of 3802) for a selection of 10 strains, mostly from Asia, chosen to represent each species or the most divergent population within a species. Tip circles are colored according to the species designation.

Supplementary Figure 14

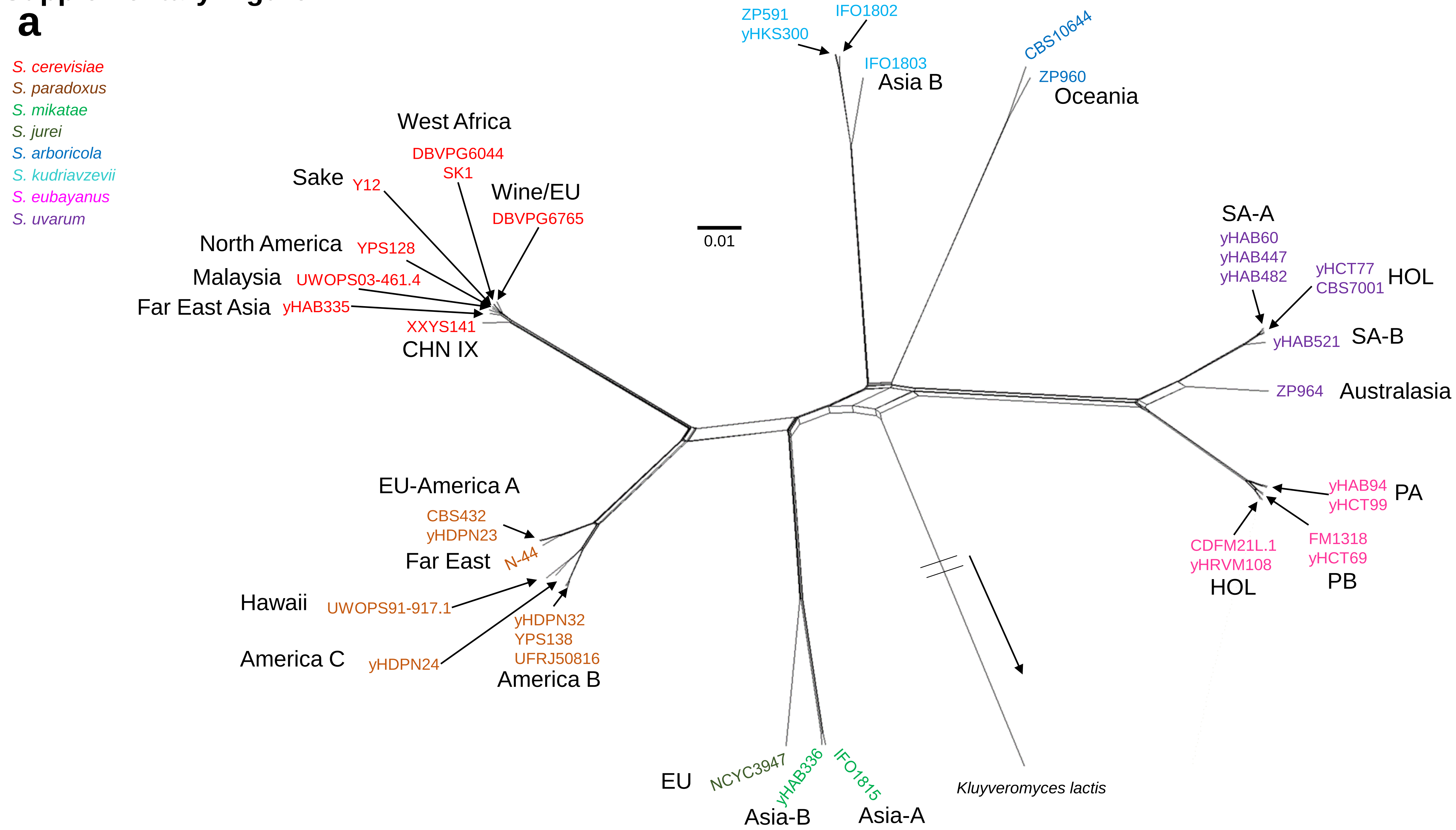

Supplementary Figure 14

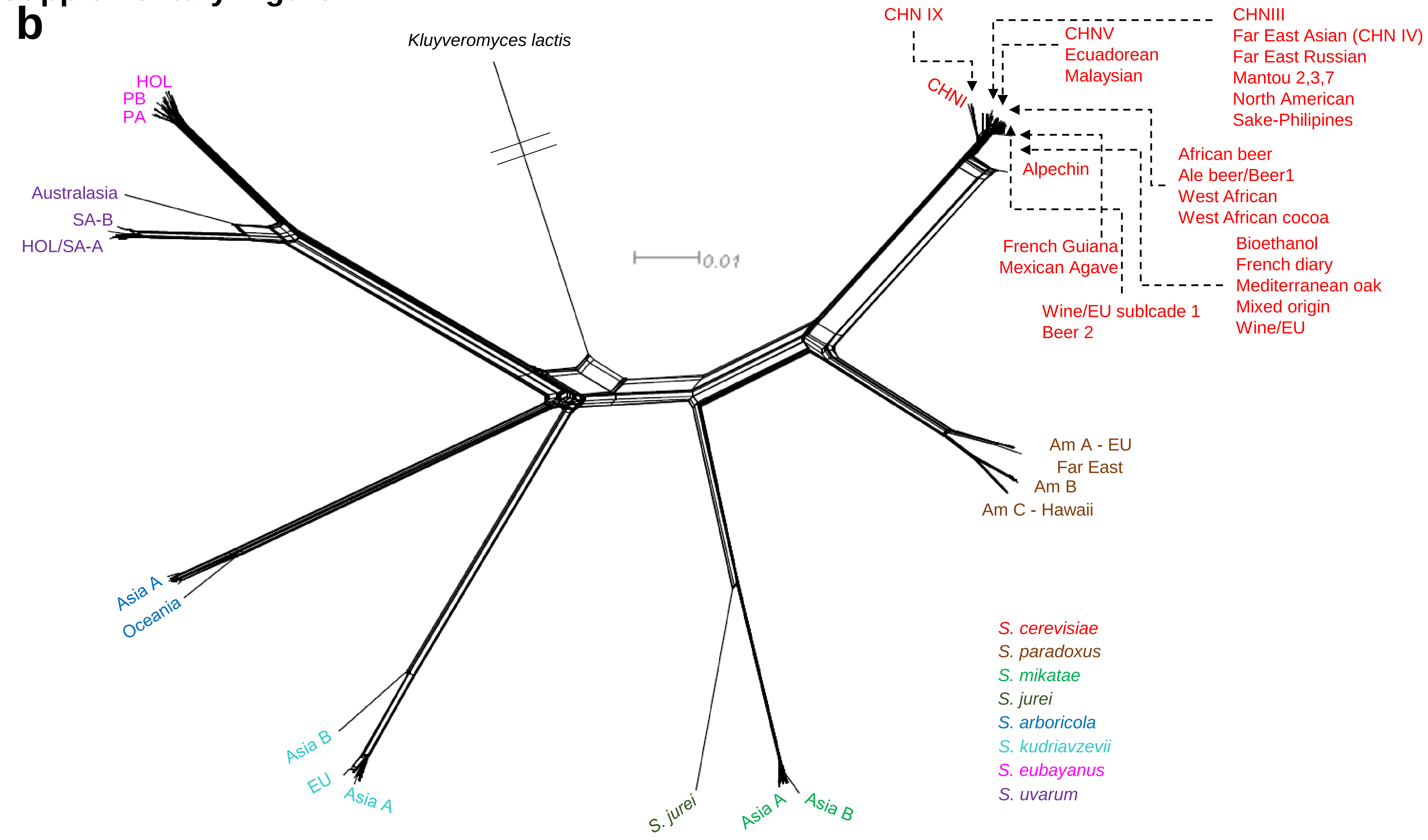

Supplementary Figure 14. Phylogenomic network of *Saccharomyces* single-copy orthologous genes.

Neighbor-Net phylogenetic network reconstructed with a concatenated alignment of 3859 (~5.5 Mbp) `YGAP` genes for 38 strains (panel **a**, Supplementary Data 1), as well as for 14 (11.5 Kbp) single-copy orthologous (`BUSCO`) genes for 163 strains (panel **b**, Supplementary Data 1). Strain names in **a**), and linages in **b)**, are colored according to their species designations. The scale bar represents the number of substitutions per site. *Kluyveromyces lactis* is the outgroup. A ML phylogenetic tree of 14 BUSCO genes is shown in Figure 5b, and the schematic representation of the ML phylogenetic tree is shown in Figure 4bi-ii.

Supplementary Figure 15

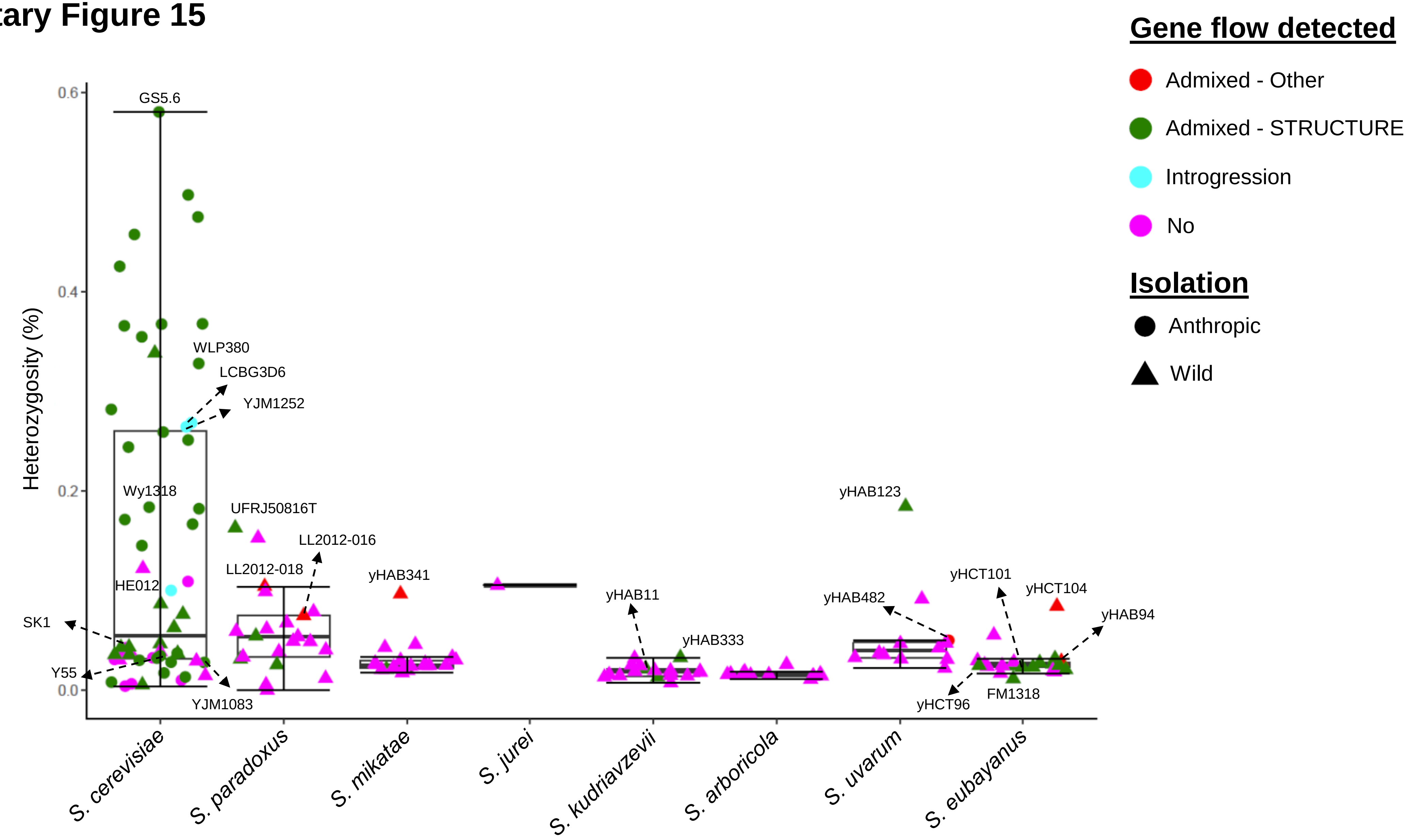

Supplementary Figure 15. Levels of heterozygosity among *Saccharomyces* strains.

Boxplots show the percentage of heterozygous sites across the genomes for each *Saccharomyces* strain (n = 163, Supplementary Data 1), grouped by species. Color dots correspond to the type of gene flow detected. We considered admixed-`STRUCTURE` strains those showing a membership coefficient lower than 0.99. Admixed-Other strains were detected by `PopGenome` plots (Supplementary Figure 10) or `sppIDer` plots. Whether a strain is anthropic or wild is indicated by a circle or triangle, respectively. Strain names with quantified gene flow (Supplementary Data 3, Supplementary Figure 10, 16) are displayed. Median values for the species are represented by a horizontal line inside the box, and the upper and lower whiskers represent the highest and lowest values of the 1.5 * IQR (inter-quartile range), respectively

Supplementary Figure 16

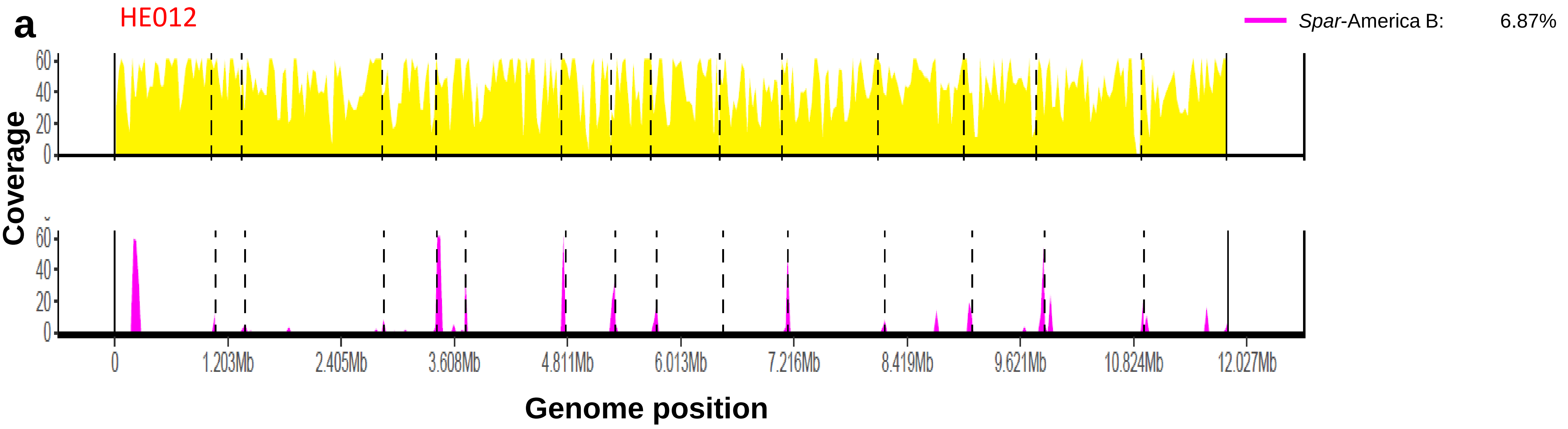

Supplementary Figure 16

b

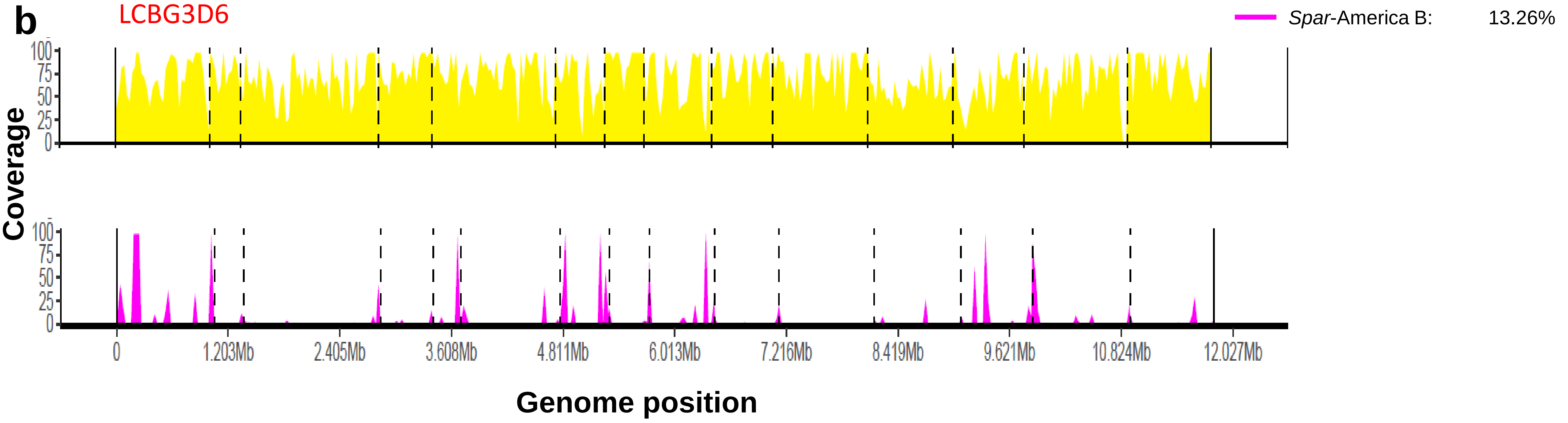

Supplementary Figure 16

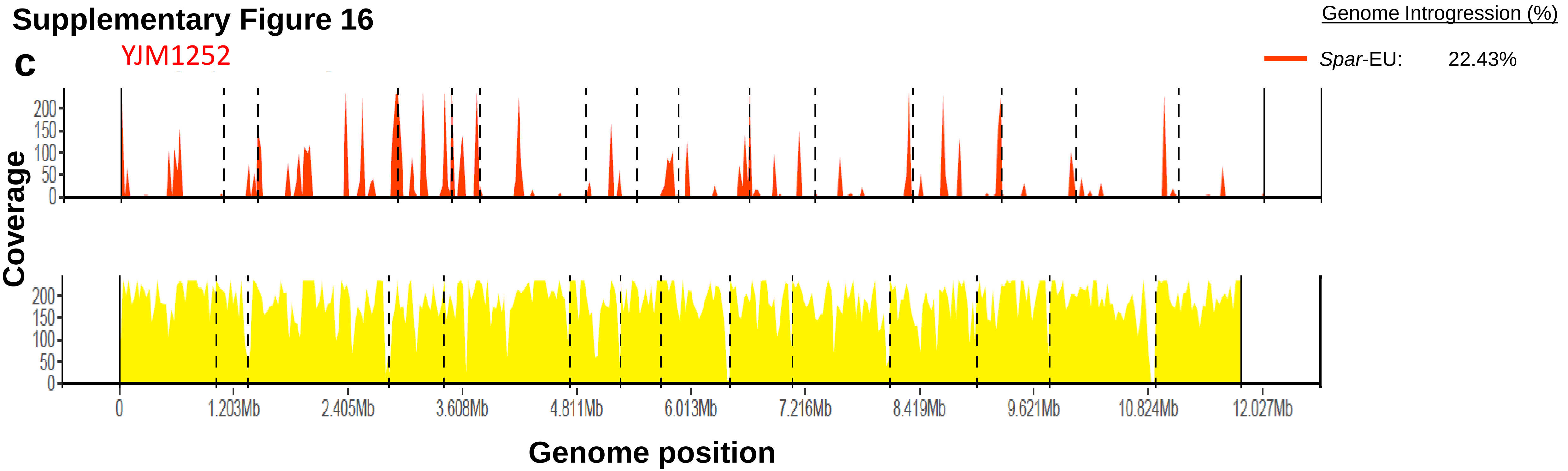

Supplementary Figure 16

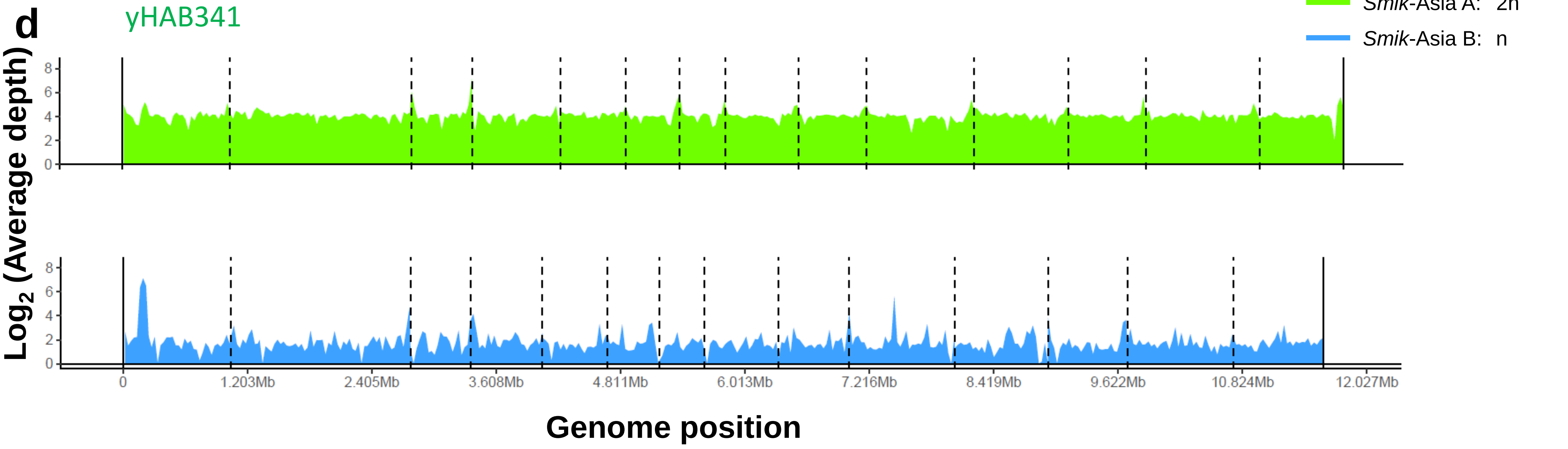

Supplementary Figure 16. Introgressions between *S. cerevisiae* and *S. paradoxus* and recent admixture between *S. mikatae* populations.

`sppIDer` plots of three *S. cerevisiae* strains with evidence of *S. paradoxus* introgressions are shown in panels **a-c**). The recent admixture event between a diploid Asia A and a haploid Asia B strain of *S mikatae*, generating a triploid Asia A (2n) and Asia B (n) strain of *S. mikatae* is shown in panel **d**). The y-axis is the average coverage depth. As reference genomes, we used a representative high-quality genome for each *Saccharomyces* lineage. Strain names are colored according to their species designations.

Supplementary Figure 17

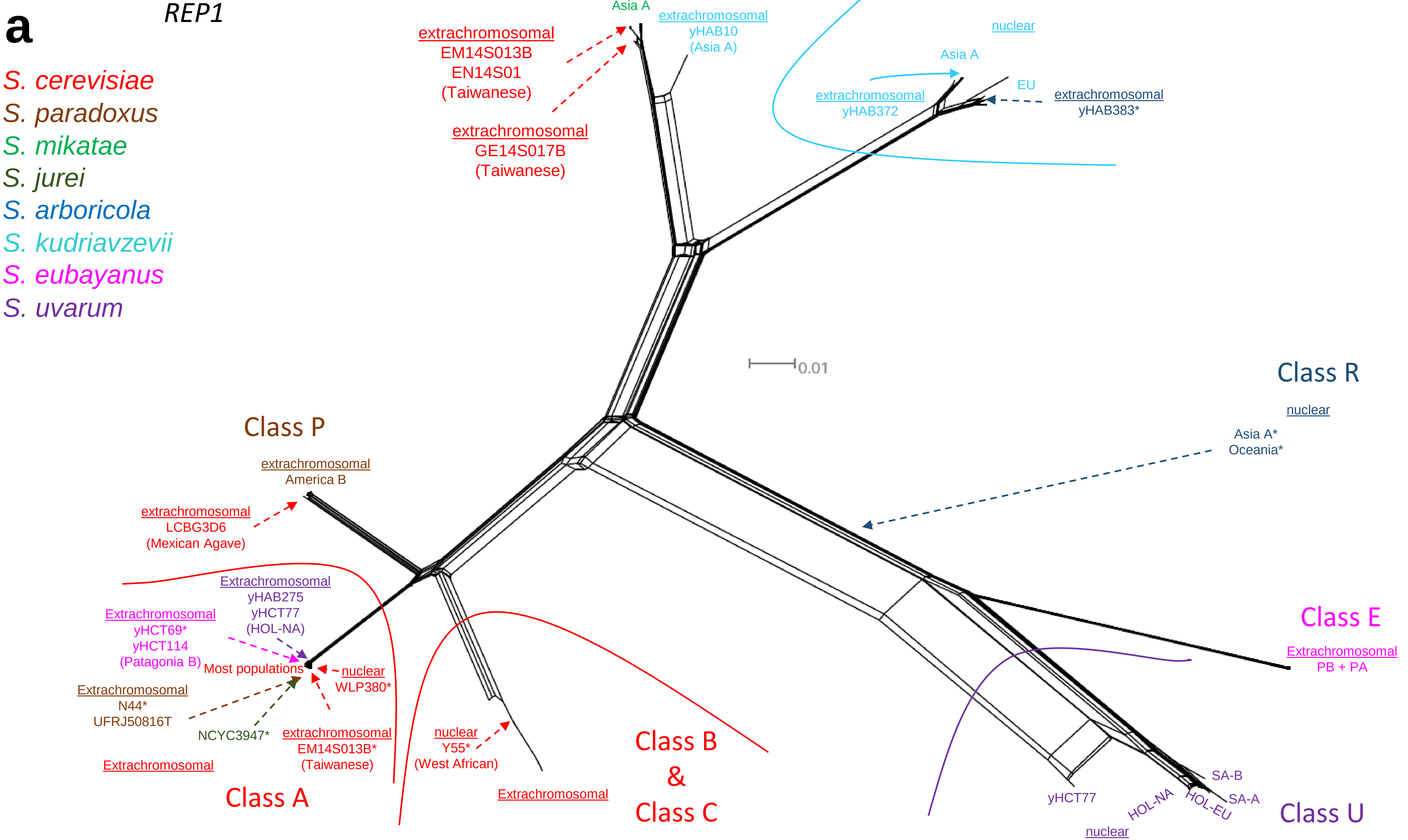

Supplementary Figure 17

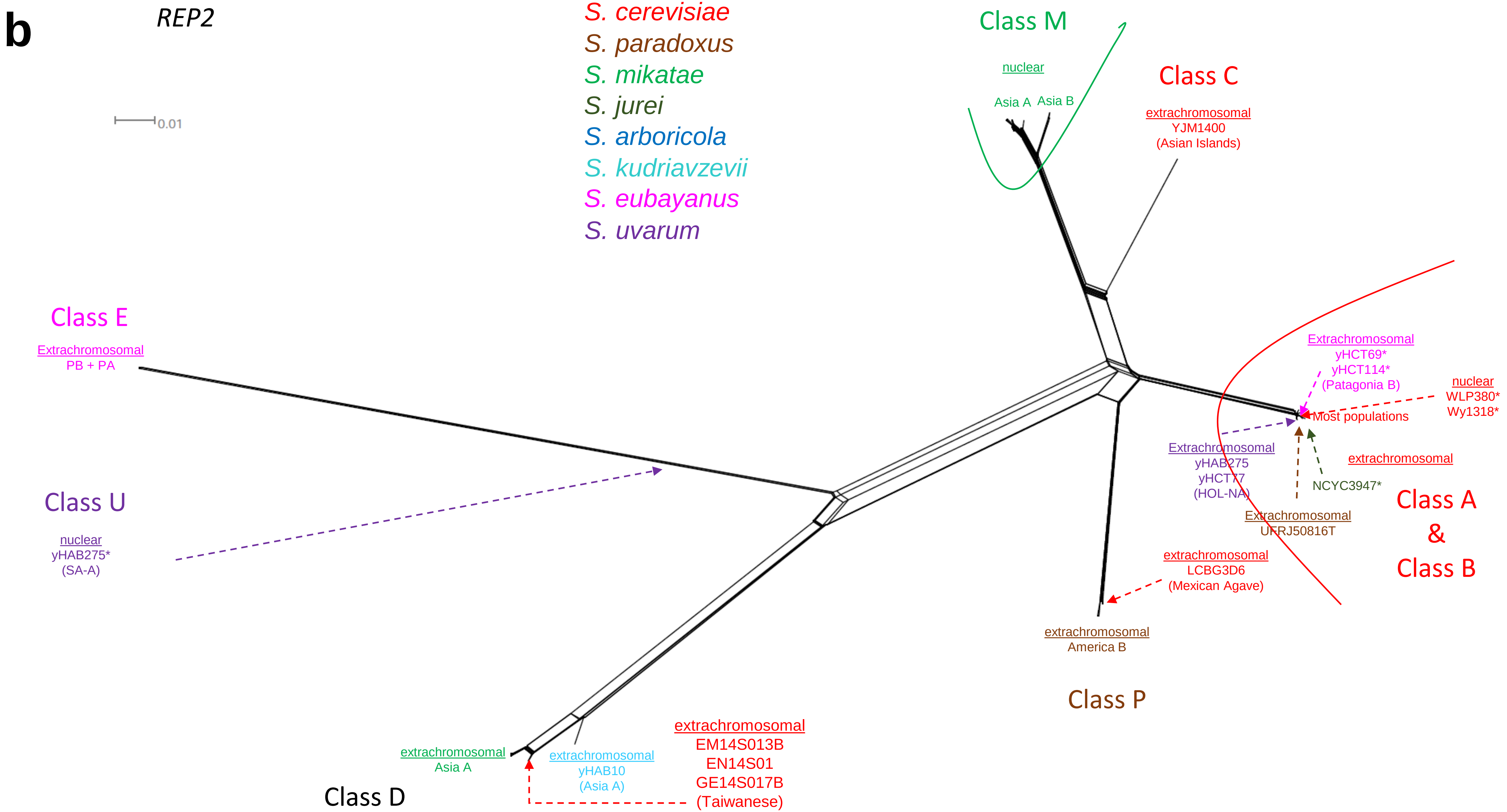

Supplementary Figure 17. *Saccharomyces* 2-µm plasmid inheritance.

Neighbor-Net phylogenetic networks for *REP1* (panel A) and *REP2* (panel B) 2-µm plasmid genes (Supplementary Data 4). *Saccharomyces* population, species 2-µm plasmid class, and strain names of interest are colored according to their species designations. Class D was colored in black due to its uncertain species designation. Asterisks highlight sequences that were too short or incomplete and were individually explored to confirm their relationships. The scale is given in nucleotide substitutions per site.

Supplementary Figure 18

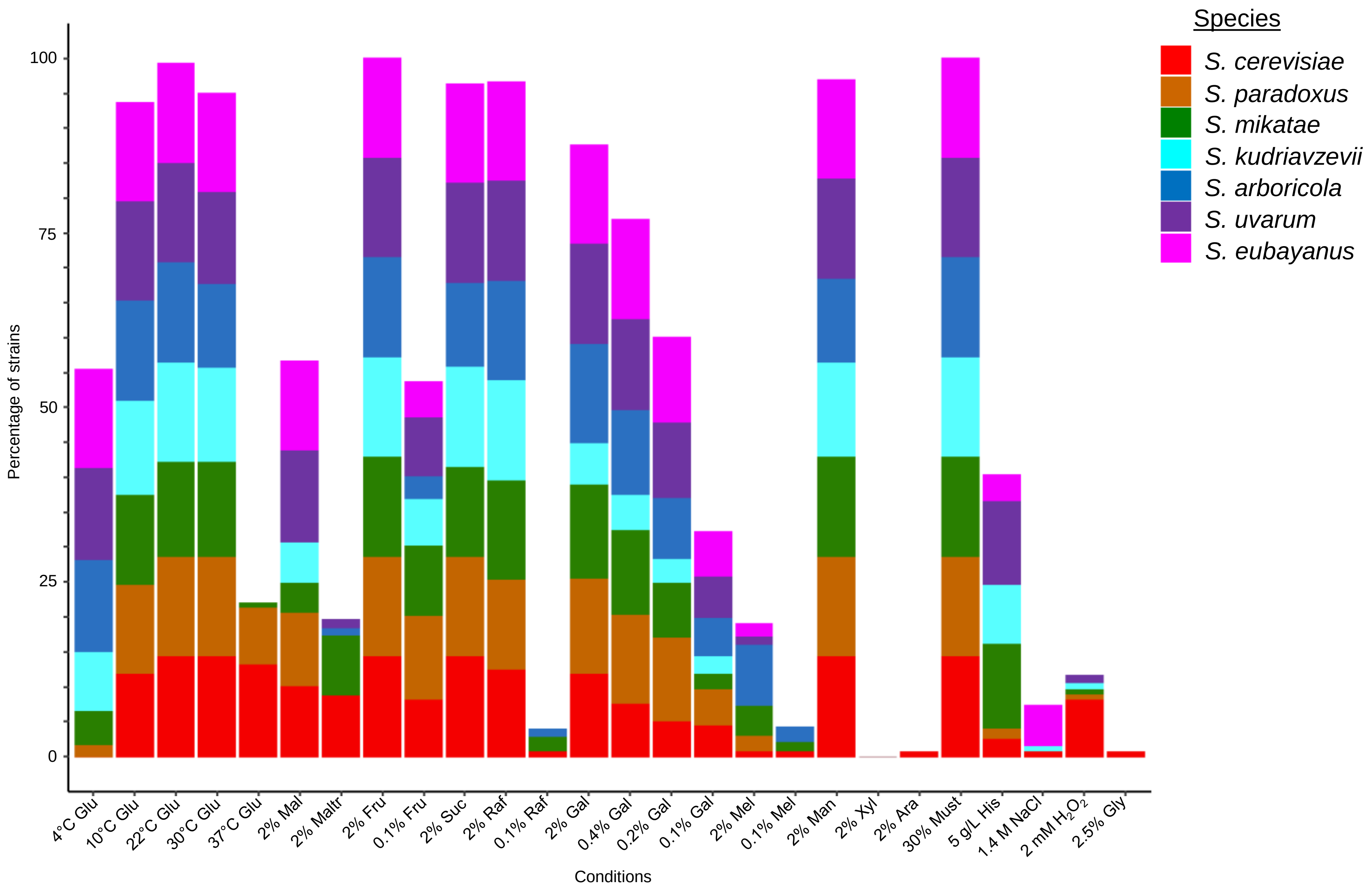

Supplementary Figure 18. Percentage of *Saccharomyces* that grew above OD_{600}=0.5 in various growth conditions.

A stacked bar plot for *Saccharomyces* strains growing above OD_{600}=0.5 is shown (Supplementary Data 6). Values were normalized by species such that each species represents 1/7 of the total, and each species is colored according to its species designation. Bars are colored according to the species designations.

Supplementary Figure 19

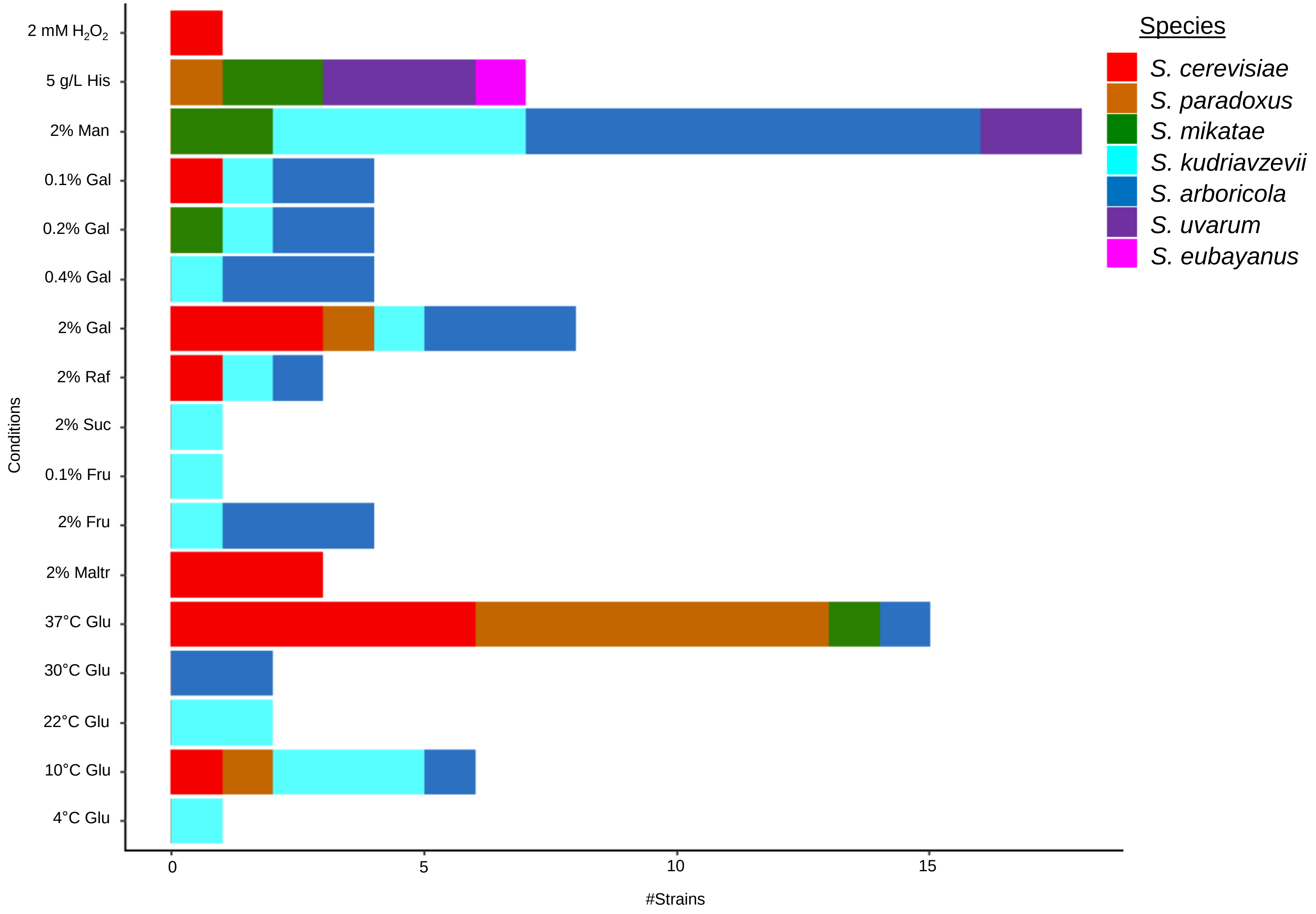

Supplementary Figure 19. Growth conditions promoting flocculation among *Saccharomyces* strains.

A stacked bar plot shows the number of *Saccharomyces* strains flocculating in various growth conditions (Supplementary Data 6). Bars are colored according to the species designations.

Supplementary Figure 20

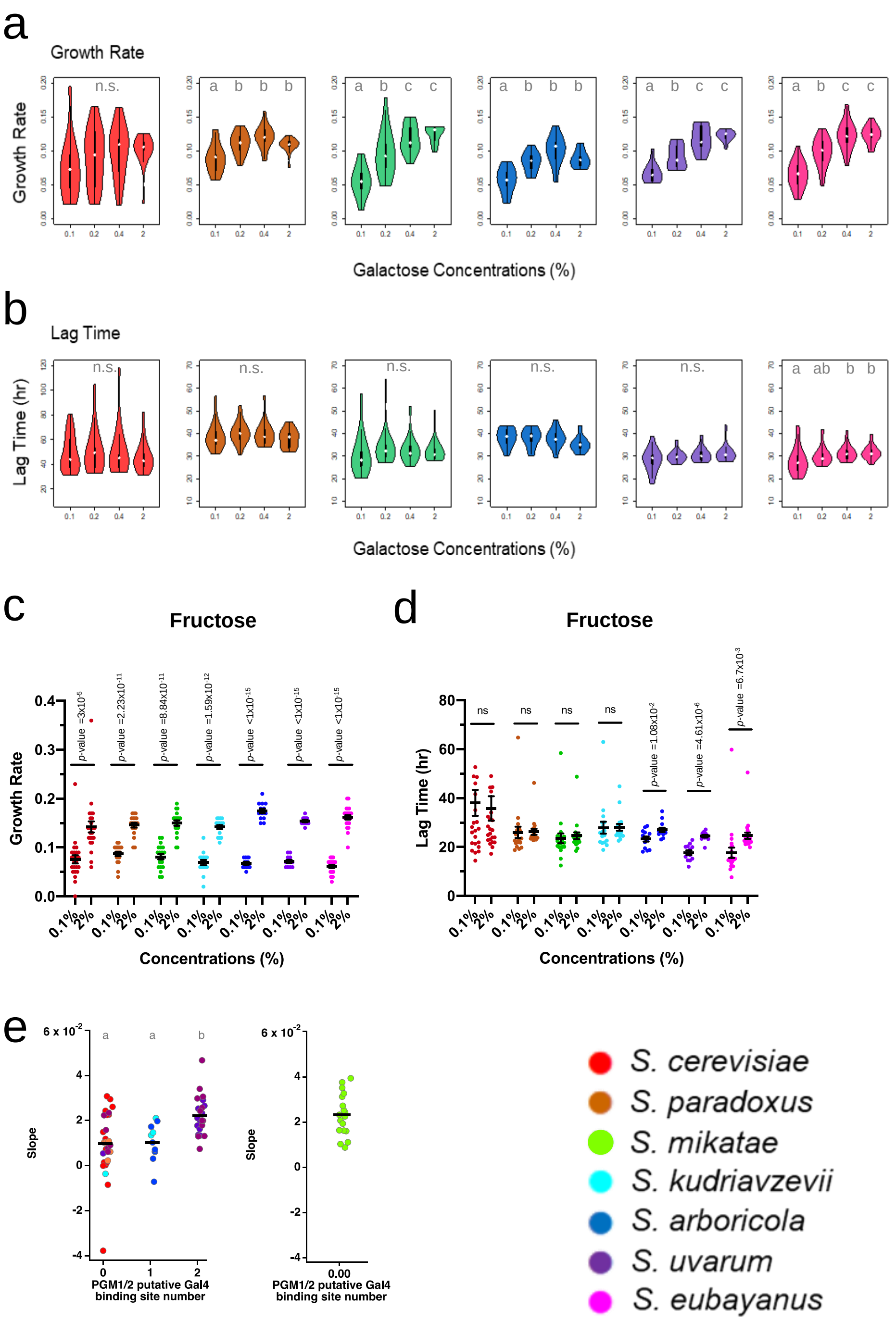

Supplementary Figure 20. Growth variation in simple sugars across concentrations and the impact of Gal4-binding sites.

a) The maximum growth rate distribution of each species across galactose concentrations from 0.1 % to 2 % (Supplementary Data 6). **b)** The lag time distribution of each species across galactose concentrations from 0.1 % to 2 %. Median values for the maximum growth rate (panel **a**) and lag time (panel **b**) are represented by a horizontal line inside the violin, and the upper and lower whiskers represent the highest and lowest values of the 1.5 * IQR (inter-quartile range), respectively. Two bounds of each box correspond to 25th and 75th percentile, respectively. Panels **c)** and **d)** show dot plots for the maximum growth rate and lag time, respectively, for each species at 0.1 % fructose and 2 % fructose. Mean values for the maximum growth rate (panel **c**) and lag time (panel **d**) are represented by a horizontal line inside the box, and the upper and lower whiskers represent the SEM (Standard Error of the Mean), respectively. **e)** Slope of maximum growth rate against galactose concentrations plotted against the number of putative Gal4-binding sites ($CGGN_{11}CCG$) upstream (maximum 1 Kbp distance) of *PGM1/2*. We observed an increase in the slope when the number of putative Gal4-binding sites is 2, except for *S. mikatae* and some *S. cerevisiae* and *S. uvarum* strains that contained 0 putative Gal4-binding sites. One-way ANOVA followed by Tukey's test was performed in **a**, **b**, and **e**. Two-sided with 95% confidence interval and p-values were adjusted by Tukey's test. Nonbold letters inside plots (panels **a**, **b**, **e**) indicate the significant homogeneous groups obtained by one-way ANOVA analysis followed by Tukey's test (p-value <0.05); n.s.: not significant. Unpaired two-tailed t-test with 95%

confidence interval was performed in **c** (100 degrees of freedom) and **d** (126 degrees of freedom): *p*-values are indicated in the plots. Violin plots (panels **a** and **b**) were built with `vioplot 0.3.2 R` package [7], and dotplots (panels **c** to **e**) were drawn with `GraphPad Prism 8` (RRID:SCR_002798). The number of species-specific strains and biological replicates are indicated in Supplementary Data 6.

Supplementary Figure 21

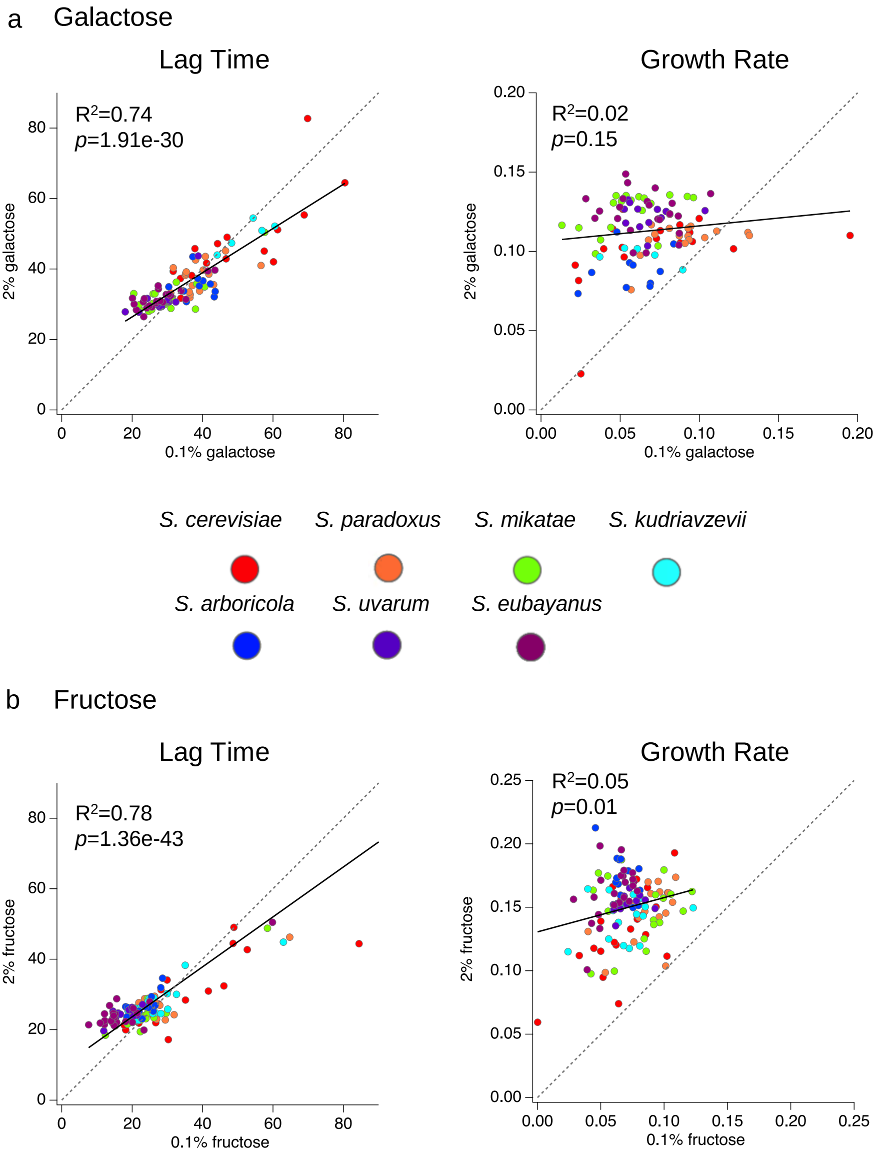

Supplementary Figure 21. Lag time and maximum growth rate correlations between low and high sugar concentrations.

Correlation for lag time and maximum growth rates of strains cultured at low (0.1 %) and high (2 %) concentrations of galactose (**a)** and fructose (**b)** (Supplementary Data 6). Linear regression lines are plotted as black solid lines. The dashed gray line is y=x. Data points are colored according to their species designations. R^2 values from linear regression and *p*-values calculated from F-tests for regression are indicated in the top right corner in each plot. Plots were drawn with `Igor Pro 9` (RRID:SCR_000325). The number of species-specific strains and biological replicates are indicated in Supplementary Data 6.

Supplementary Figure 22

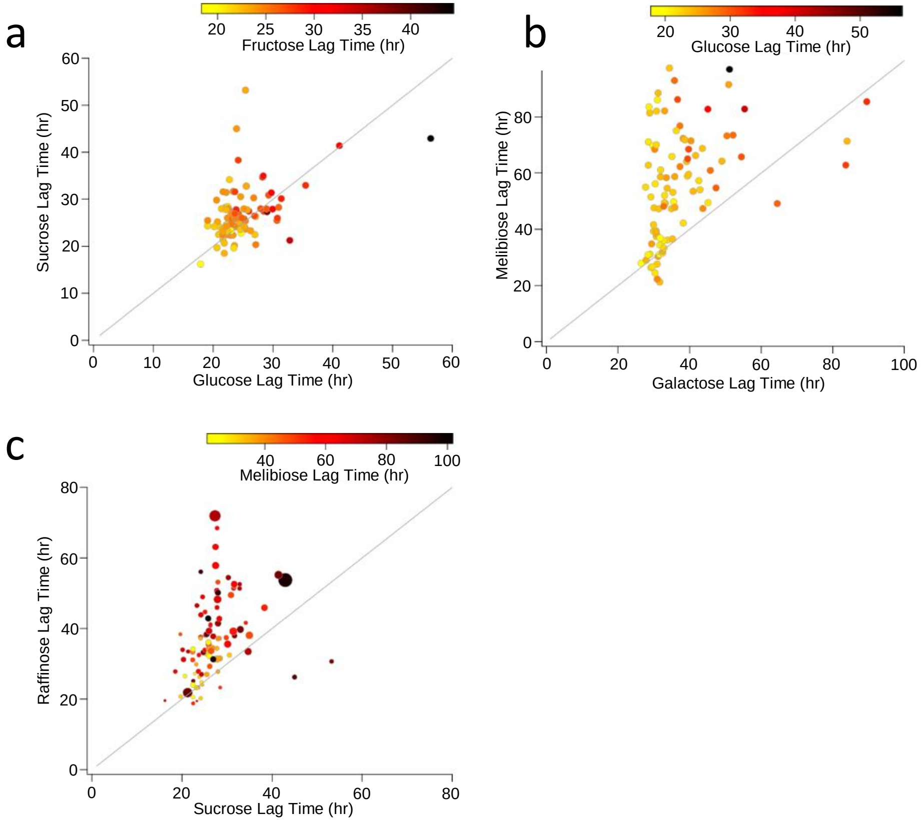

Supplementary Figure 22. Lag time correlations between monosaccharides and their disaccharides or trisaccharides.

a) Lag time on sucrose (y-axis) is plotted by variation in its constituent monosaccharide units, glucose (x-axis) and fructose (color-coded by lag time) (Supplementary Data 6). **b)** Lag time on melibiose (y-axis) is plotted by variation in its constituent monosaccharide units, glucose (color-coded by lag time) and galactose (x-axis) (Supplementary Data 6). **c)** Lag time on raffinose (y-axis) is plotted by variation in the constituent disaccharides sucrose (x-axis) and melibiose (color-coded by lag time) (Supplementary Data 6). Data point sizes are proportional to the lag time on fructose, which is generally the first constituent monosaccharide unit consumed, leaving behind melibiose. Each data point represents an average of three biological replicates from one strain. Data for a total of 96 strains are shown (*S. arboricola*=5, *S. cerevisiae*=15, *S. eubayanus*=22, *S. kudriavzevii*=7, *S. mikatae*=18, *S. paradoxus*=17, *Suva*=12). Plots were drawn with `Igor Pro 7` (RRID:SCR_000325). The number of species-specific strains and biological replicates are indicated in Supplementary Data 6.

Supplementary Figure 23

a

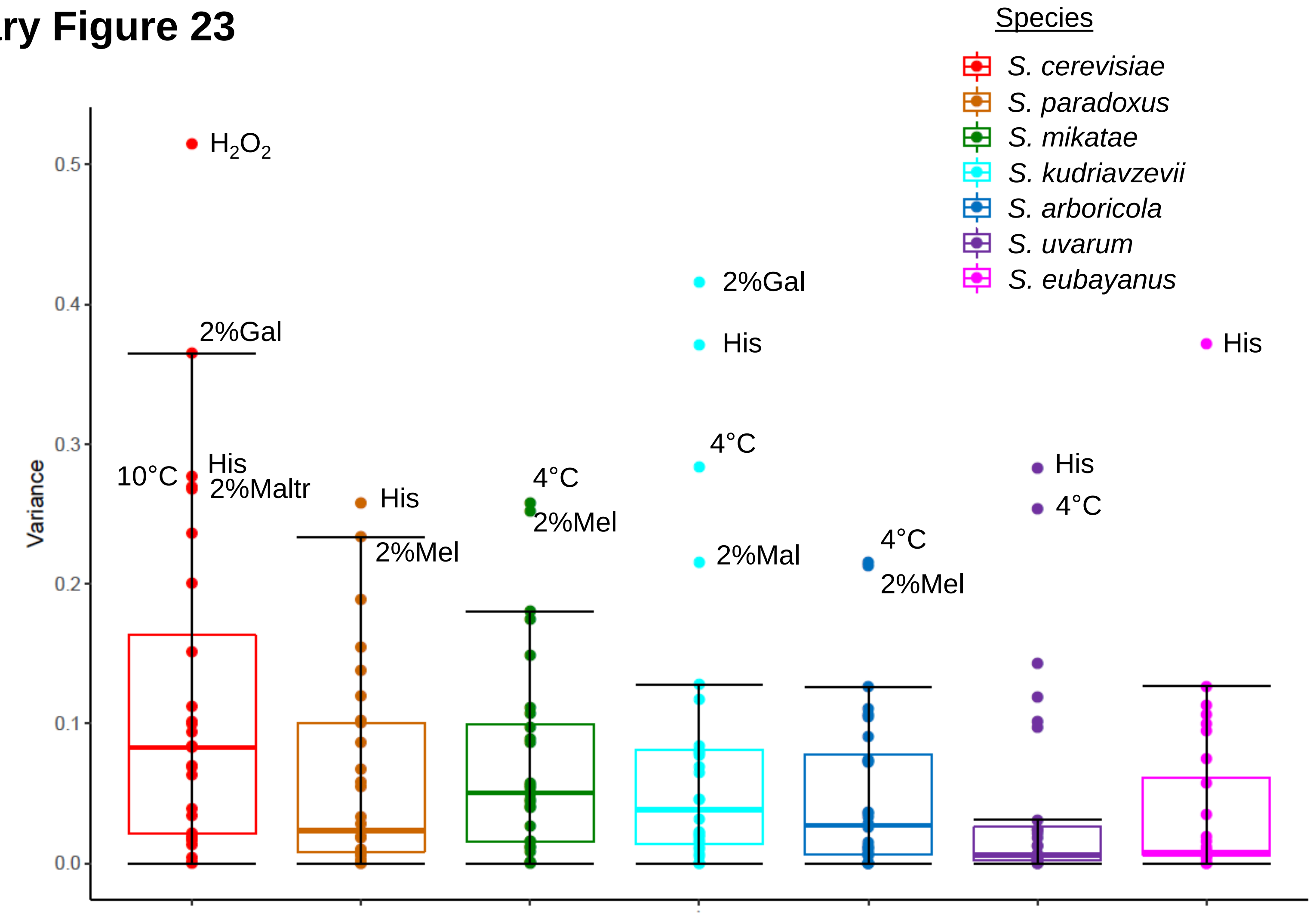

Supplementary Figure 23

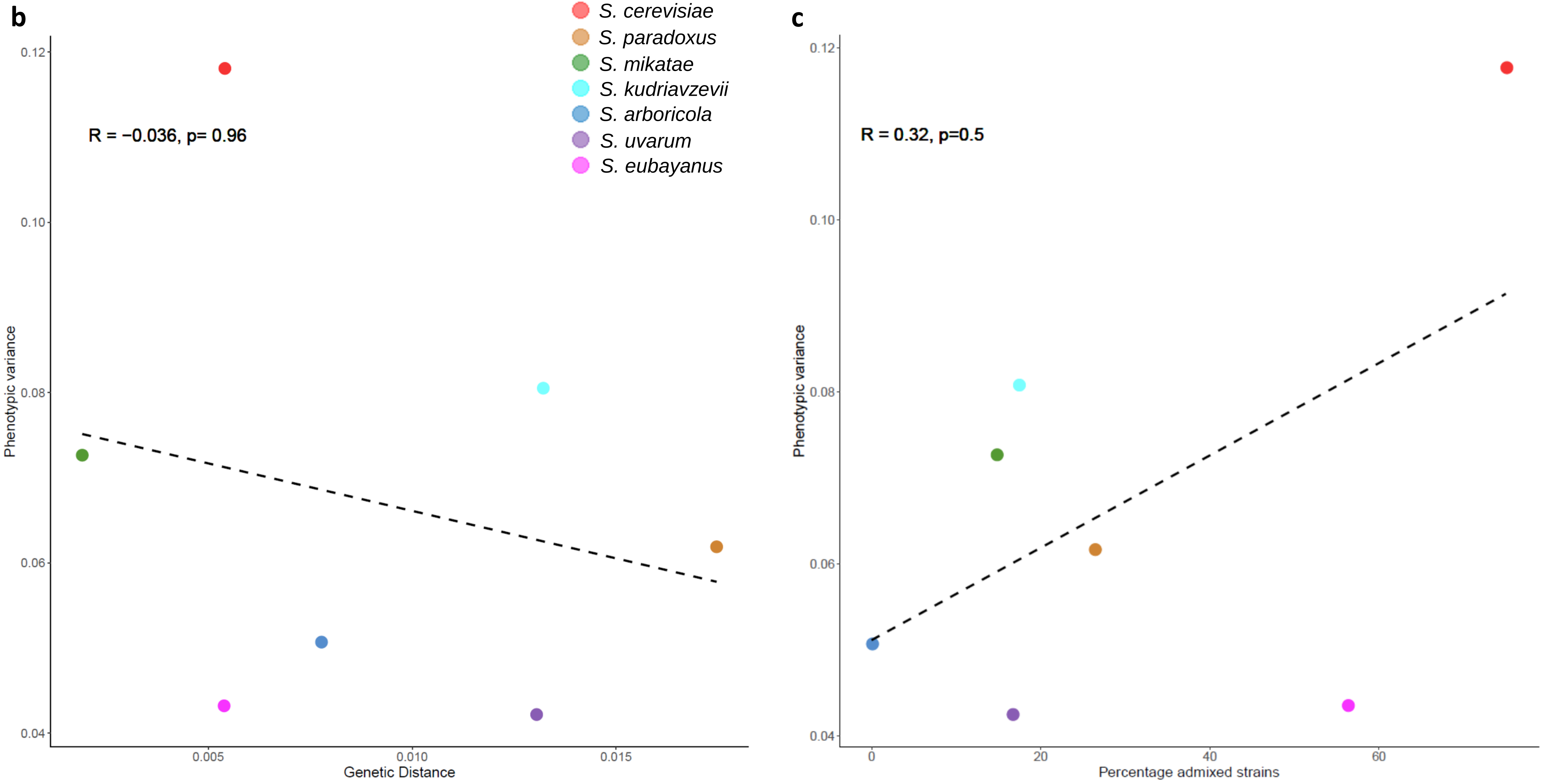

Supplementary Figure 23. Phenotypic variance across *Saccharomyces* species.

a) Phenotypic variance in maximum OD_{600} (Supplementary Data 6) across the genus *Saccharomyces* is shown for several growth conditions. Median variance is represented by a horizontal line inside the box, and the upper and lower whiskers represent the highest and lowest values of the 1.5*IQR (inter-quartile range), respectively. Growth conditions with unusually high variance for a species are noted close to their values. Gal: Galactose; His: Histidine; Mal: Maltose; Maltr: Maltotriose; Mel: Melibiose. **b)** Spearman correlation test of the average Tamura-Nei corrected genetic distance within species and the average of phenotypic variance for the species. **c)** Spearman correlation test of the percentage of admixed strains detected for each species and the average of phenotypic variance for each species. The number of species-specific strains and biological replicates are indicated in Supplementary Data 6.

Supplementary Figure 24

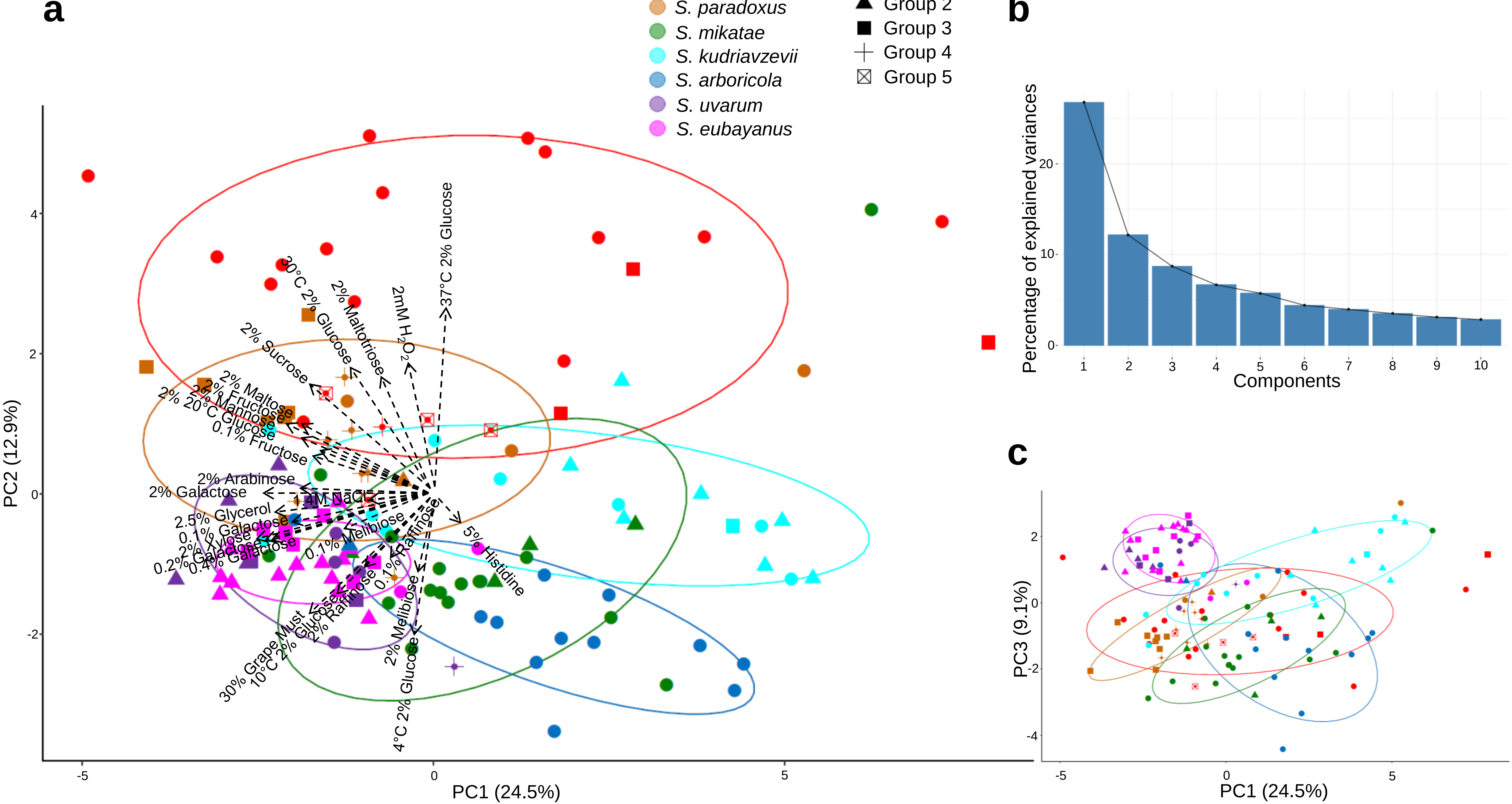

Supplementary Figure 24. Principal component analysis of maximum OD_{600}.

A higher image resolution PCA (Figure 5A) of PC1 and PC2 of the maximum OD_{600} as calculated from growth curves (n = 3) (Supplementary Data 6) is shown in panel **a)**. PC1 and PC2 accounted for 37.4 % of the total variation. Growth condition weights (Supplementary Figure 25) are represented by black arrows. Strains are colored according to their species designations, and different shapes represent their lineage/group designations. The percentage of variance explained by each component is shown in panel **b)**. A PCA of PC1 and PC3 accounting for 33.6 % of the total variation is shown in panel **c)**. The groups in panel **a)** and **c)** are defined as follows:

i) *S. cerevisiae*: Group1 (Domesticated strains: Bioethanol, Beer 1 & 2, Wine/European and Sake populations), Group 3 (West African population), Group 4 (CHN IV population), Group 5 (Asian Islands, Malaysian and North American populations).

ii) *S. paradoxus*: Group 1 (European population), Group 2 (Far East population), Group 3 (America B population), Group 4 (America C population).

iii) *S. mikatae*: Group 1 (Asia A population), Group 2 (Asia B population).

iv) *S. kudriavzevii*: Group 1 (EU population), Group 2 (Asia A population), Group 3 (Asia B population).

v) *S. arboricola*: Group 1 (Asia A population), Group 2 (Oceania population).

vi) *S*. *uvarum*: Group 1 (Holarctic lineage), Group 2 (South America A lineage), Group 3 (South America B population), Group 4 (Australasia population). Note that Holarctic and South America A are considered a single population in our `STRUCTURE`/`fineSTRUCTURE` analyses.

vii) *S*. *eubayanus*: Group 1 (Holarctic lineage), Group 2 (Patagonia B lineage), Group 3 (Patagonia A population). Note that Holarctic and Patagonia B are considered a single population in our `STRUCTURE`/`fineSTRUCTURE` analyses.

Supplementary Figure 25

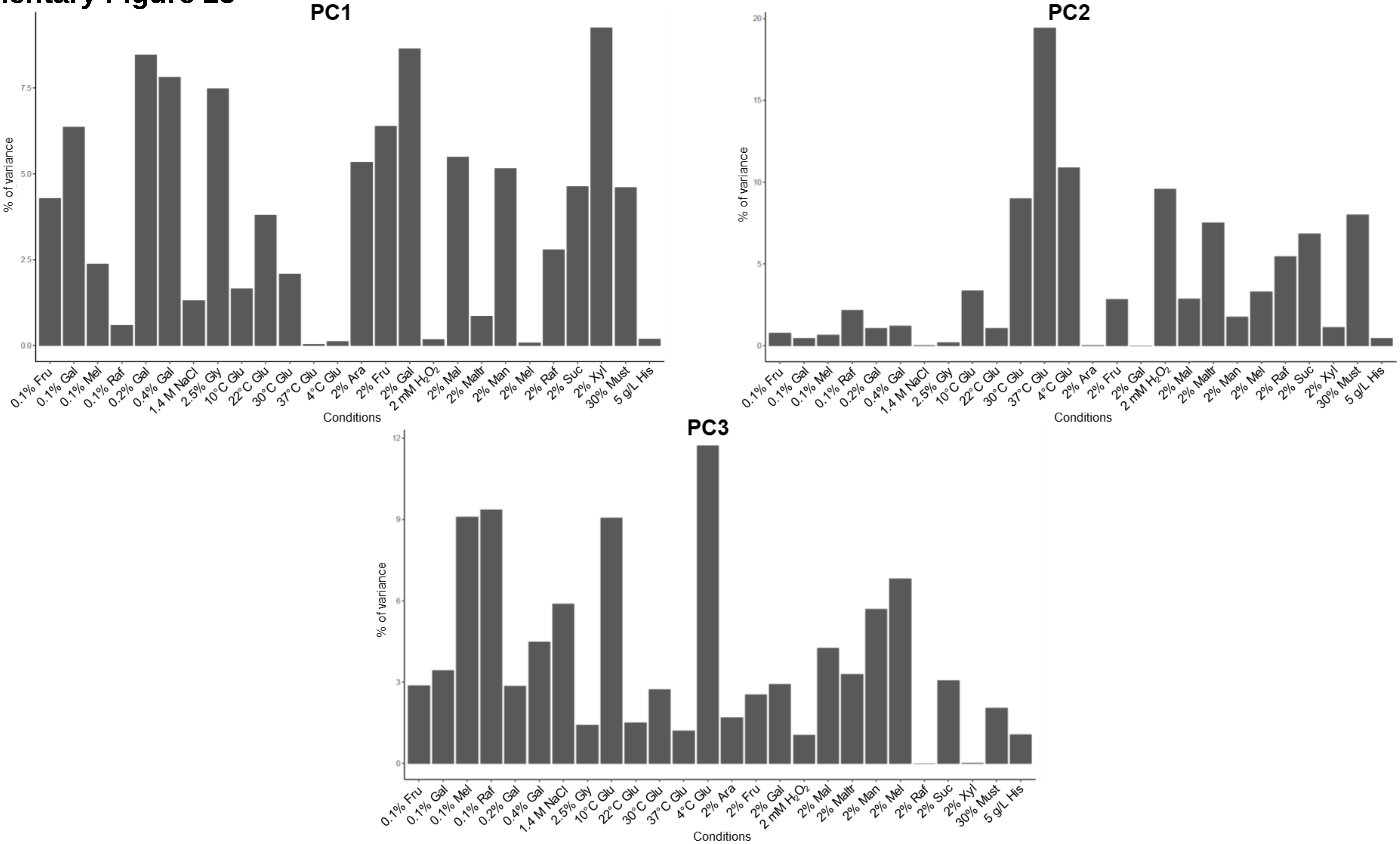

Supplementary Figure 25. Variance contributed to each component by growth condition.

The variances contributed to PC1, PC2, and PC3 by each growth condition is shown in bar plots. Ara: Arabinose; Fru: Fructose; Gal: Galactose; Gly: Glycerol; His: Histidine; Mal: Maltose; Maltr: Maltrotriose; Man: Mannose; Mel: Melibiose; Raf: Raffinose; Suc: Sucrose; Xyl: Xylose.

Supplementary Figure 26

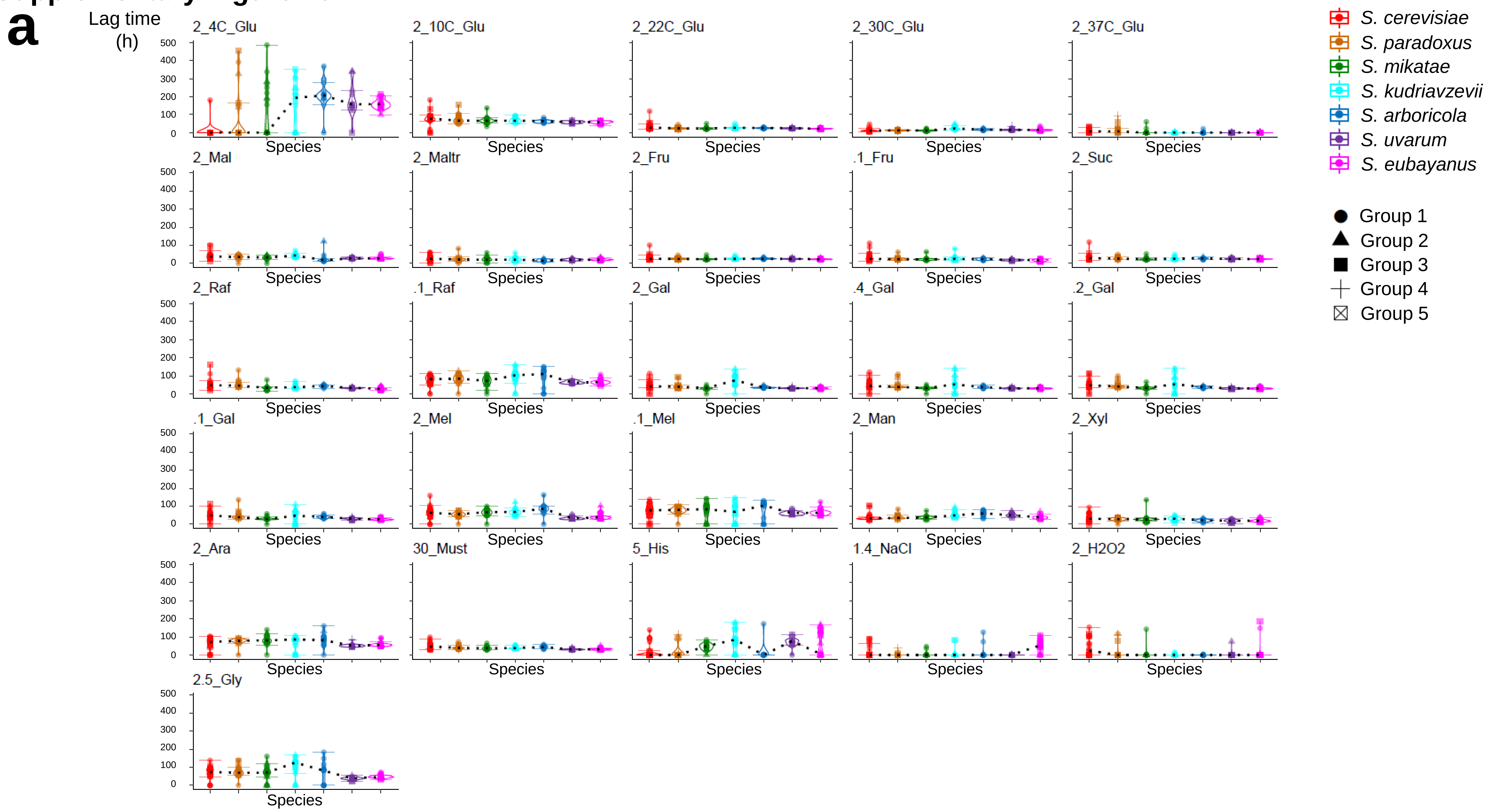

Supplementary Figure 26

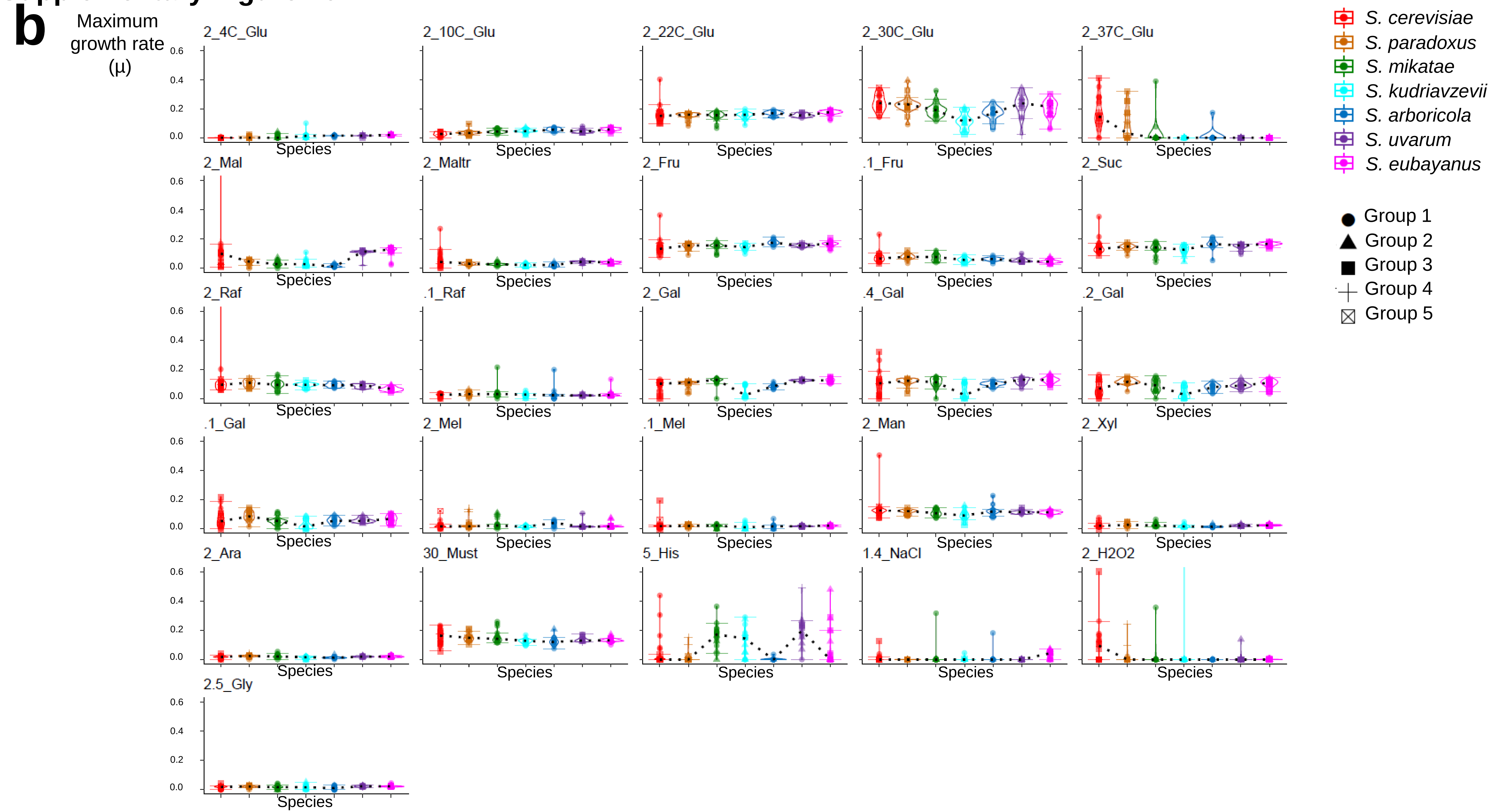

Supplementary Figure 26

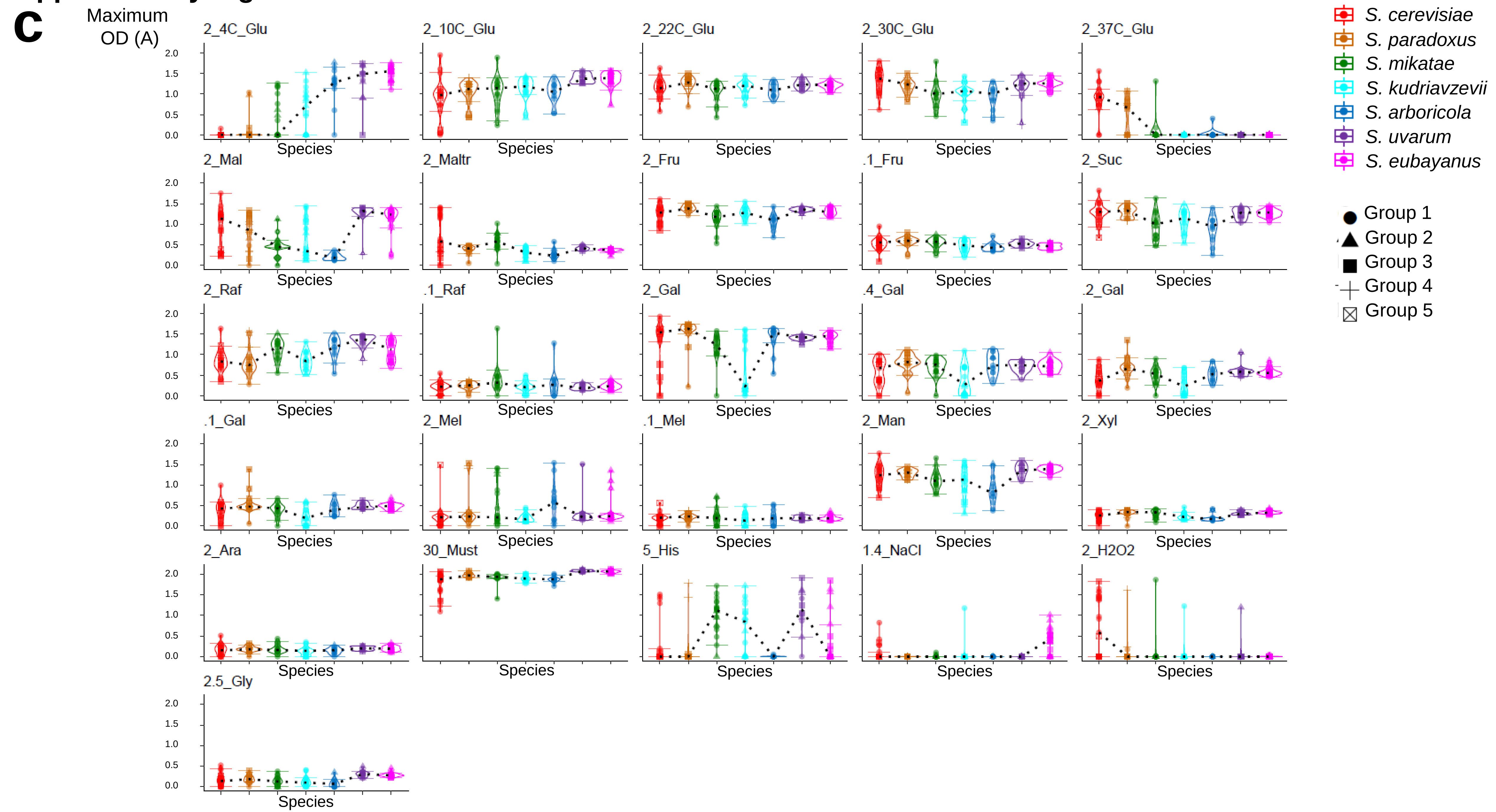

Supplementary Figure 26

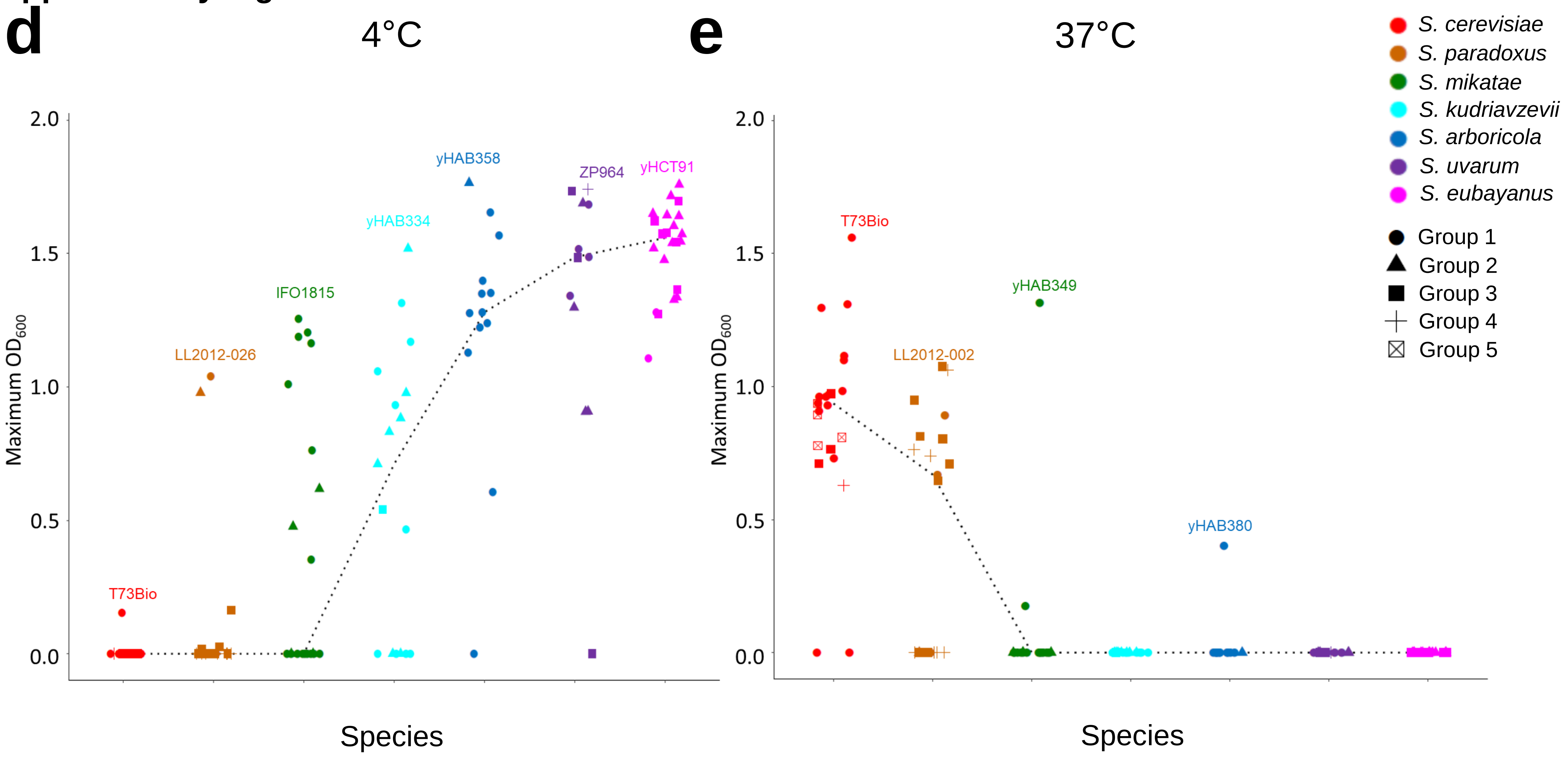

Supplementary Figure 26

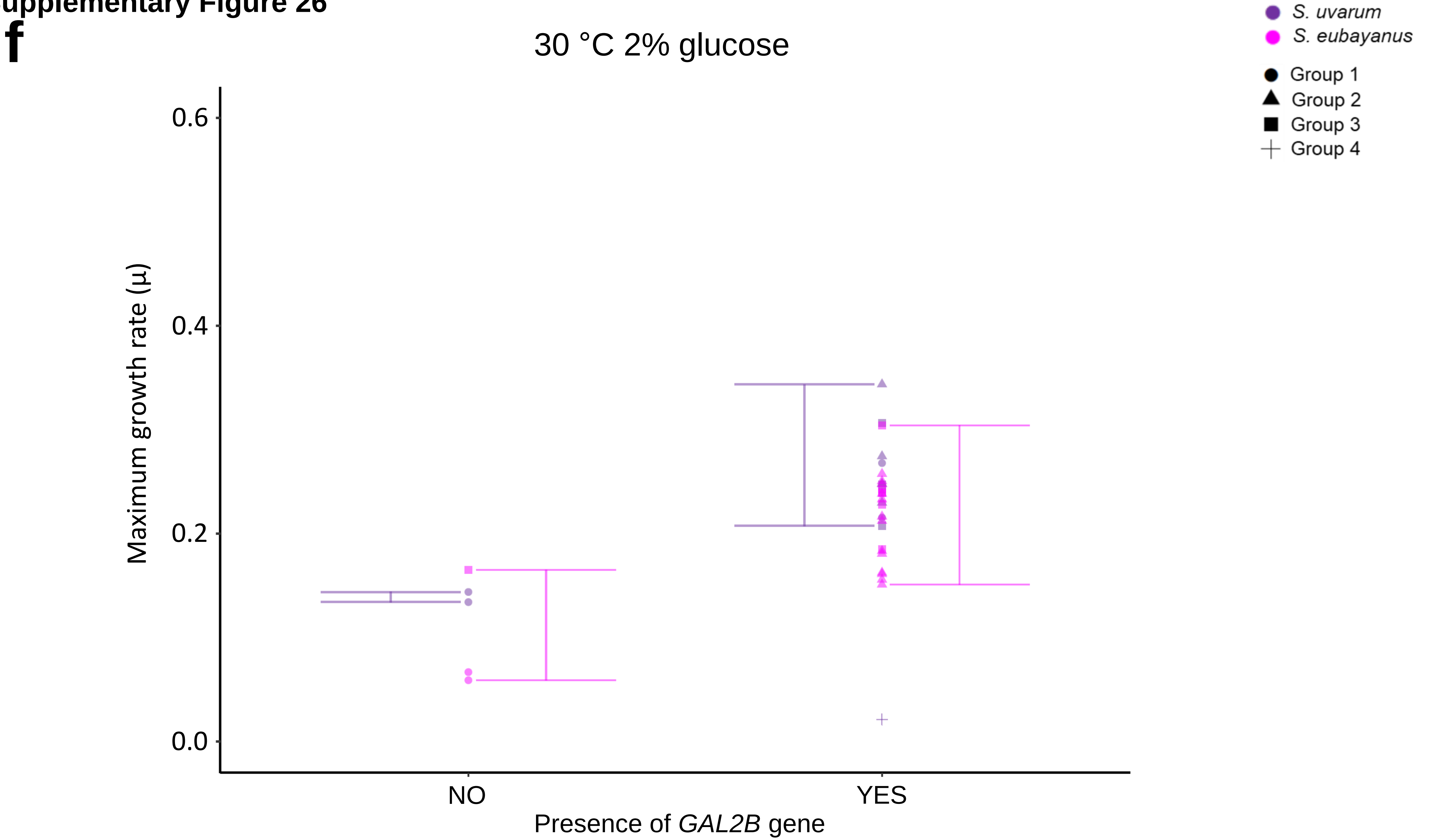

Supplementary Figure 26. Kinetic parameters of *Saccharomyces* strains in different growth conditions.

Saccharomyces species violin boxplots of adaptation time (lag time, hours), maximum specific growth rate (µ, defined as $(\ln(OD_2)-\ln(OD_1))/(T_2-T_1)$), and biomass production (maximum OD_{600}) are represented in panels **a)**, **b)**, and **c)**, respectively (Supplementary Data 6). Boxplots are colored according to their species designations. Panels **d)** and **e)** show the maximum biomass production (OD_{600}) at 4°C and 37°C temperatures in minimal media with 2 % glucose growth conditions, respectively (Supplementary Data 6). Names of the top-performing strains of each species at 4 °C and 37 °C are shown. In panels **a)-e)**, data points are split based on the species designations, and dashed lines connect the median values for each species. **f)** Median maximum growth rates for *S. eubayanus* and *S. uvarum* strains with and without *GAL2B* genes. Shapes highlight different *Saccharomyces* populations/groups. Upper and lower whiskers in panels **a)-c)** and **f)** represent the highest and lowest values of the 1.5 * IQR (inter-quartile range), respectively. The number of species-specific strains and biological replicates are indicated in Supplementary Data 6. 2_4C_Glu: 2 % glucose at 4°C; 2_10C_Glu: 2 % glucose at 10°C; 2_22C_Glu: 2 % glucose at 22°C; 2_30C_Glu: 2 % glucose at 30°C; 2_37C_Glu: 2 % glucose at 37°C; 2_Mal: 2 % maltose; 2_Maltr: 2 % maltotriose; 2_Fru: 2 % fructose; .1_Fru: 0.1 % fructose; 2_Suc: 2 % sucrose; 2_Raf: 2 % raffinose; .1_Raf: 0.1 % raffinose; 2_Gal: 2 % galactose; .4_Gal: 0.4 % galactose; .2_Gal: 0.2 % galactose; .1_Gal: 0.1 % galactose; 2_Mel: 2 % melibiose; .1_Mel: 0.1 % melibiose; 2_Man: 2 % mannose; 2_Xyl: 2 % xylose; 2_Ara: 2 % arabinose; 30_Must: 30 %

must; 5_His: 5 g/L histidine; 1.4_NaCl: 1.4 mM NaCl; 2_H2O2: 2mM H_2O_2; 2.5_Gly: 2.5 % glycerol. The groups are defined as follows:

i) *S. cerevisiae*: Group1 (Domesticated strains: Bioethanol, Beer 1 & 2, Wine/European and Sake populations), Group 3 (West African population), Group 4 (CHN IV population), Group 5 (Asian Islands, Malaysian and North American populations).

ii) *S. paradoxus*: Group 1 (European population), Group 2 (Far East population), Group 3 (America B population), Group 4 (America C population).

iii) *S*. *mikatae*: Group 1 (Asia A population), Group 2 (Asia B population).

iv) *S*. *kudriavzevii*: Group 1 (EU population), Group 2 (Asia A population), Group 3 (Asia B population).

v) *S*. *arboricola*: Group 1 (Asia A population), Group 2 (Oceania population).

vi) *S*. *uvarum*: Group 1 (Holarctic lineage), Group 2 (South America A lineage), Group 3 (South America B population), Group 4 (Australasia population). Note that Holarctic and South America A are considered a single population in our `STRUCTURE`/`fineSTRUCTURE` analyses.

vii) *S*. *eubayanus*: Group 1 (Holarctic lineage), Group 2 (Patagonia B lineage), Group 3 (Patagonia A population). Note that Holarctic and Patagonia B are considered a single population in our `STRUCTURE`/`fineSTRUCTURE` analyses.

Supplementary Figure 27

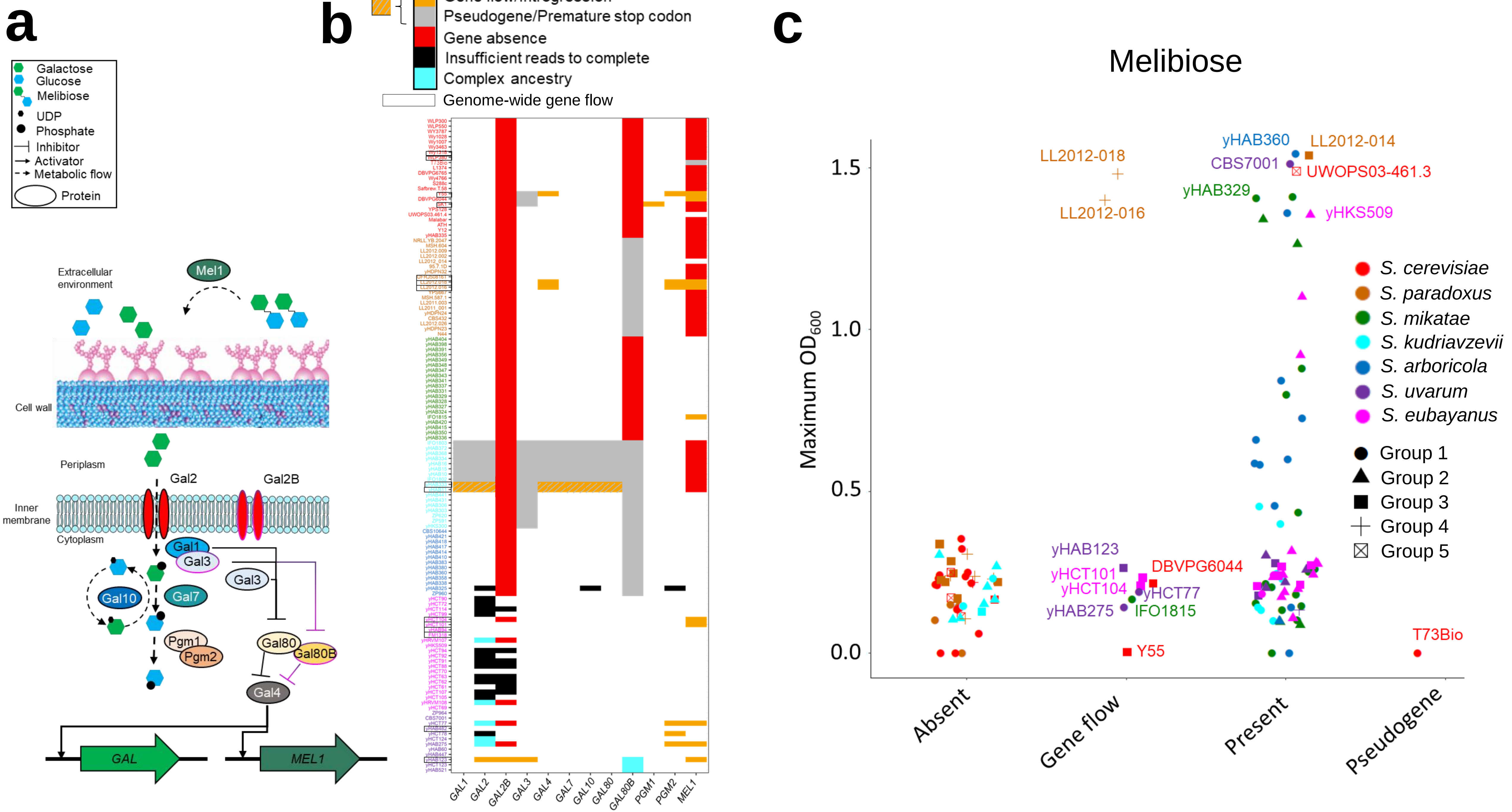

Supplementary Figure 27. Melibiose phenotypic diversity generated through complex genomic ancestries.

The *GAL*/*MEL* pathway is represented in panel **a)**. Outlines of circles representing common *Saccharomyces* proteins are colored in black. Outlines of circles representing proteins specific to *S. eubayanus* and *S. uvarum* are colored mixing purple and pink according to the species' colors [3,8]. Lines of specific *S. eubayanus* and *S. uvarum* activities are colored mixing purple and pink colors. *Saccharomyces* strains with complex ancestries for the *GAL/MEL* pathway genes are shown in panel **b)**. Names of strains with genome-wide admixture (Supplementary Data 3) are boxed. Incomplete gene sequences due to low coverage are labelled as black. Complete genes with a phylogenetic position (Supplementary Figure 28) as expected based on population genomic analysis (Supplementary Figure 9) are labeled as white. Genes acquired from another lineage by gene flow (within species) or introgression (between species) are labelled orange. Genes with premature stop codons or in a more advanced state of pseudogenization are labelled gray. Genes with complex ancestries are labelled cyan. Genes not detected by any of the methods employed in this study (see Online Material and Methods) were considered to have been evolutionarily lost and are labelled red. Maximum biomass production (OD_{600}) in 2 % melibiose is shown in panel **c)** (Supplementary Data 6). Each point is a strain colored by its species designation. Data were split based on the absence, gene flow/introgression, presence, or pseudogene state of *MEL1*. The groups in panel **c)** are defined as follows:

i) *S. cerevisiae*: Group1 (Domesticated strains: Bioethanol, Beer 1 & 2, Wine/European and Sake populations), Group 3 (West African population), Group 4 (CHN IV population), Group 5 (Asian Islands, Malaysian and North American populations).

ii) *S. paradoxus*: Group 1 (European population), Group 2 (Far East population), Group 3 (America B population), Group 4 (America C population).

iii) *S*. *mikatae*: Group 1 (Asia A population), Group 2 (Asia B population).

iv) *S*. *kudriavzevii*: Group 1 (EU population), Group 2 (Asia A population), Group 3 (Asia B population).

v) *S*. *arboricola*: Group 1 (Asia A population), Group 2 (Oceania population).

vi) *S*. *uvarum*: Group 1 (Holarctic lineage), Group 2 (South America A lineage), Group 3 (South America B population), Group 4 (Australasia population). Note that Holarctic and South America A are considered a single population in our `STRUCTURE`/`fineSTRUCTURE` analyses.

vii) *S*. *eubayanus*: Group 1 (Holarctic lineage), Group 2 (Patagonia B lineage), Group 3 (Patagonia A population). Note that Holarctic and Patagonia B are considered a single population in our `STRUCTURE`/`fineSTRUCTURE` analyses.

Supplementary Figure 28

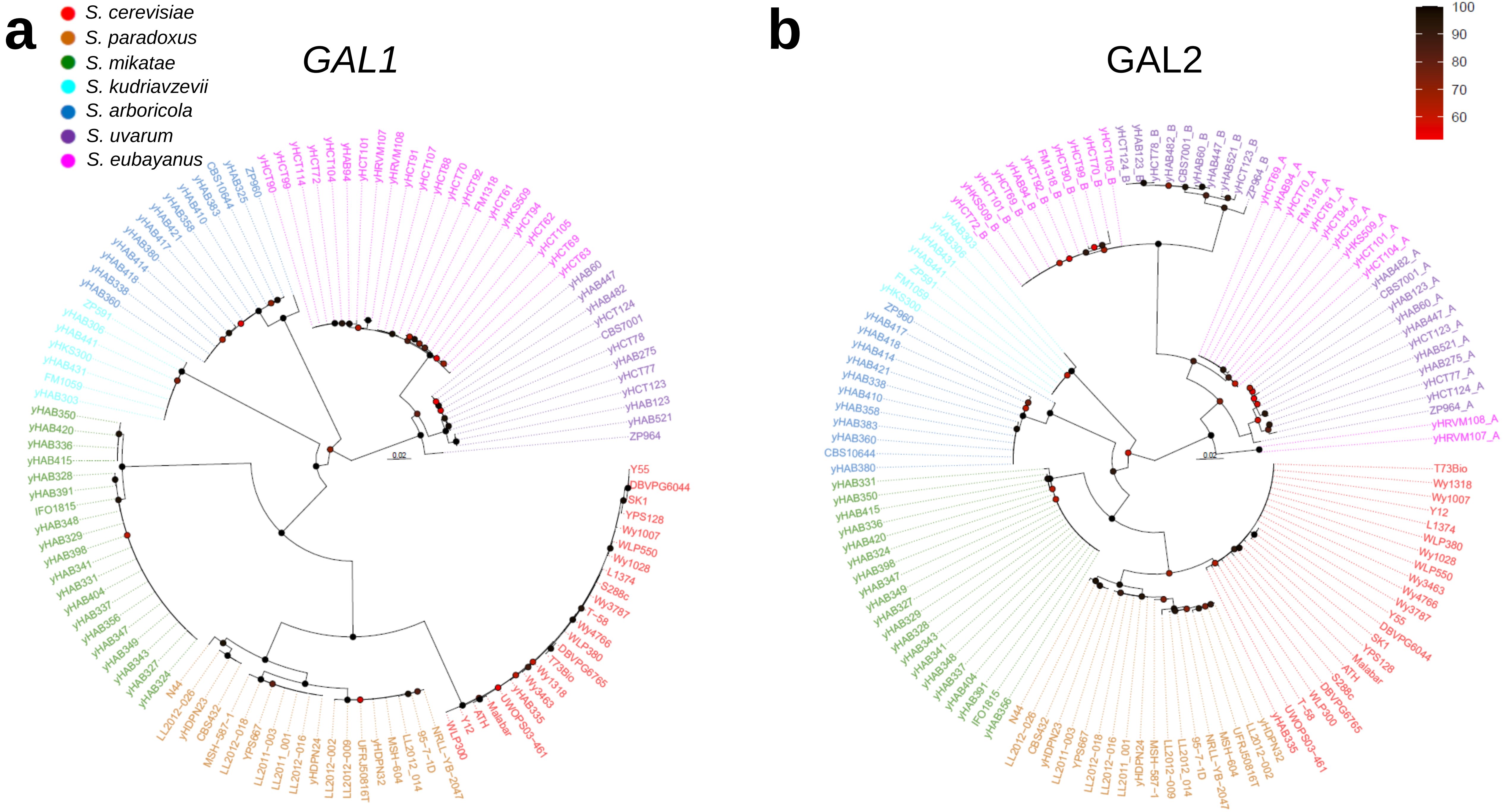

Supplementary Figure 28

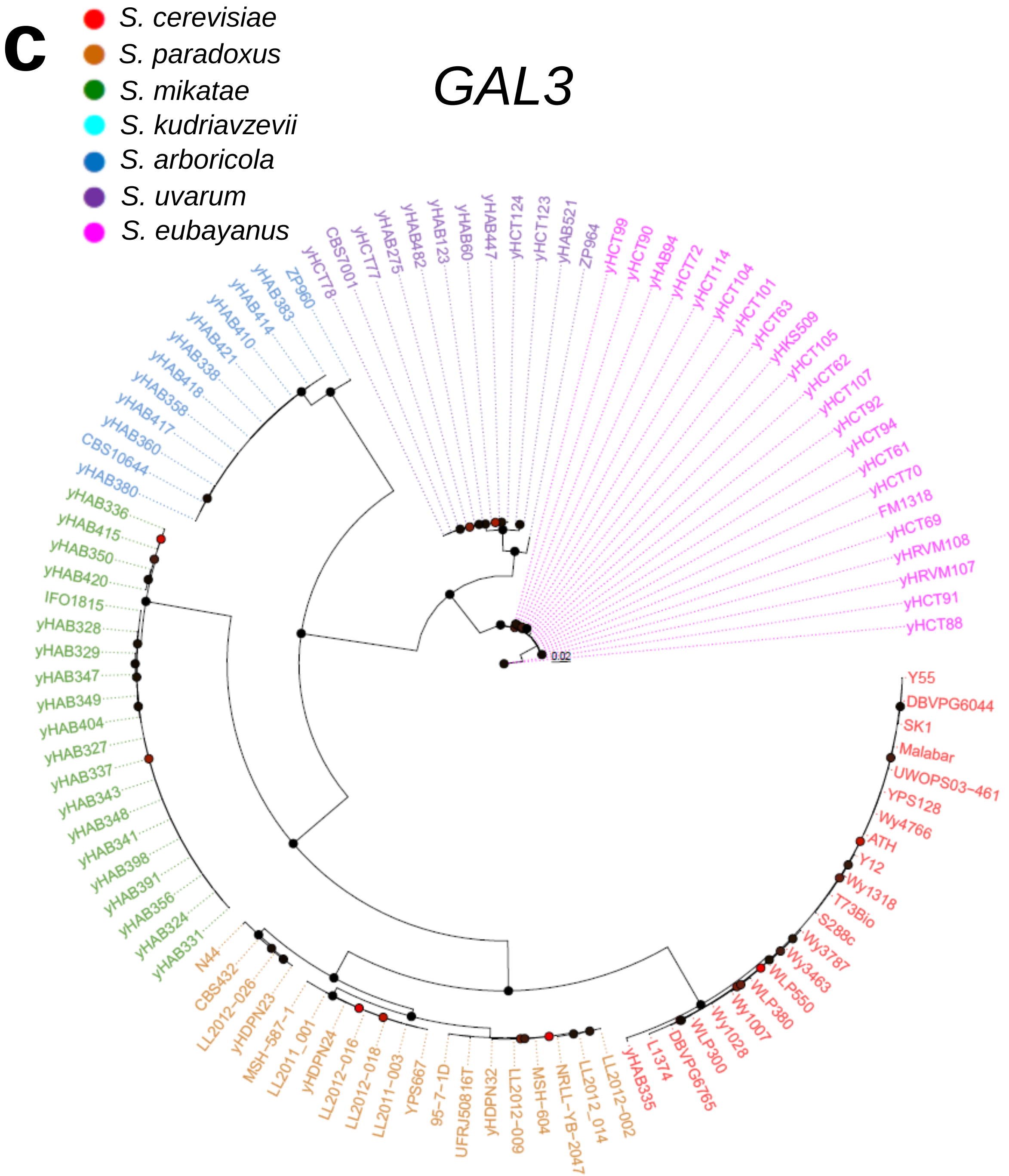

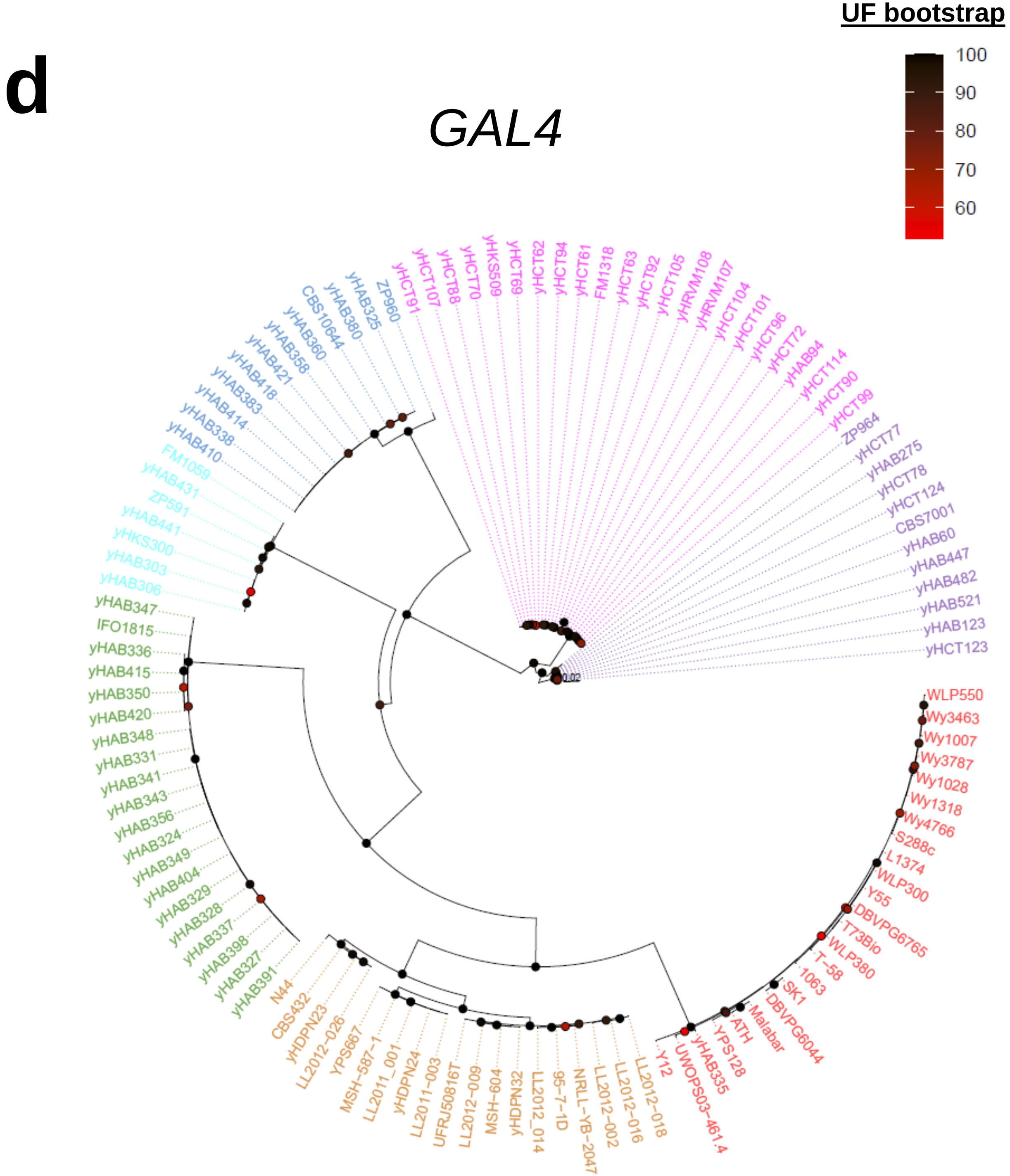

Supplementary Figure 28

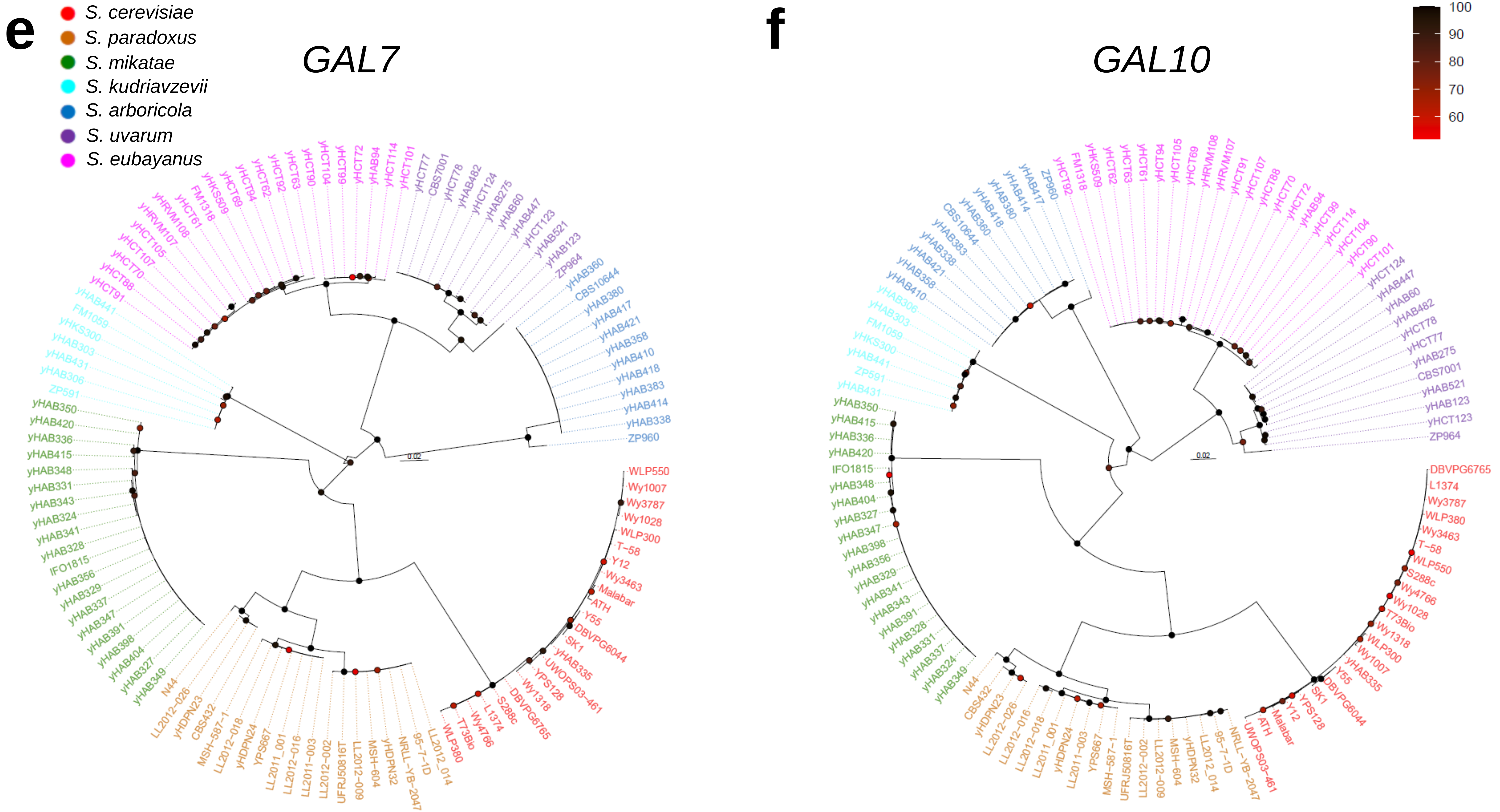

Supplementary Figure 28

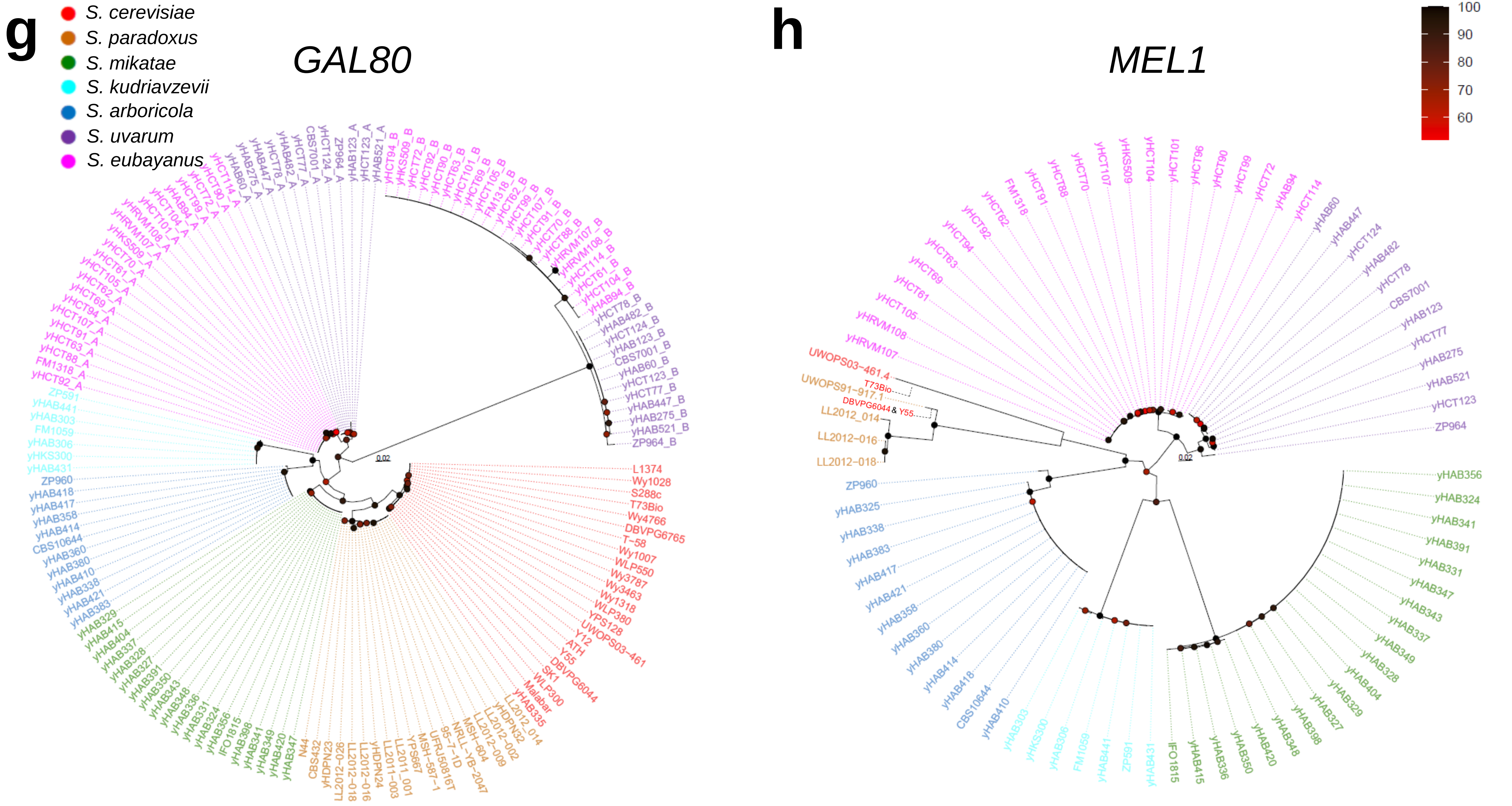

Supplementary Figure 28

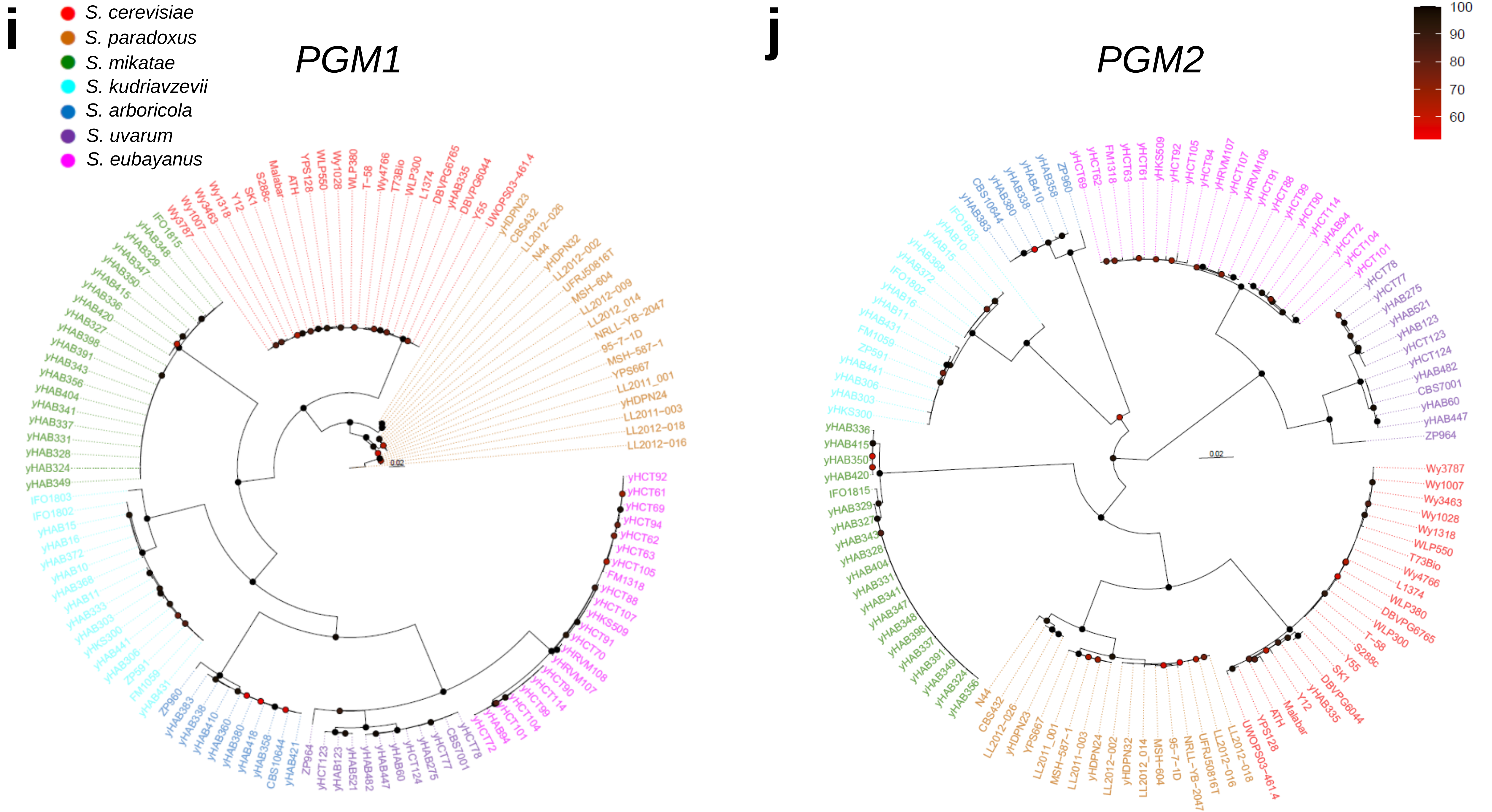

Supplementary Figure 28

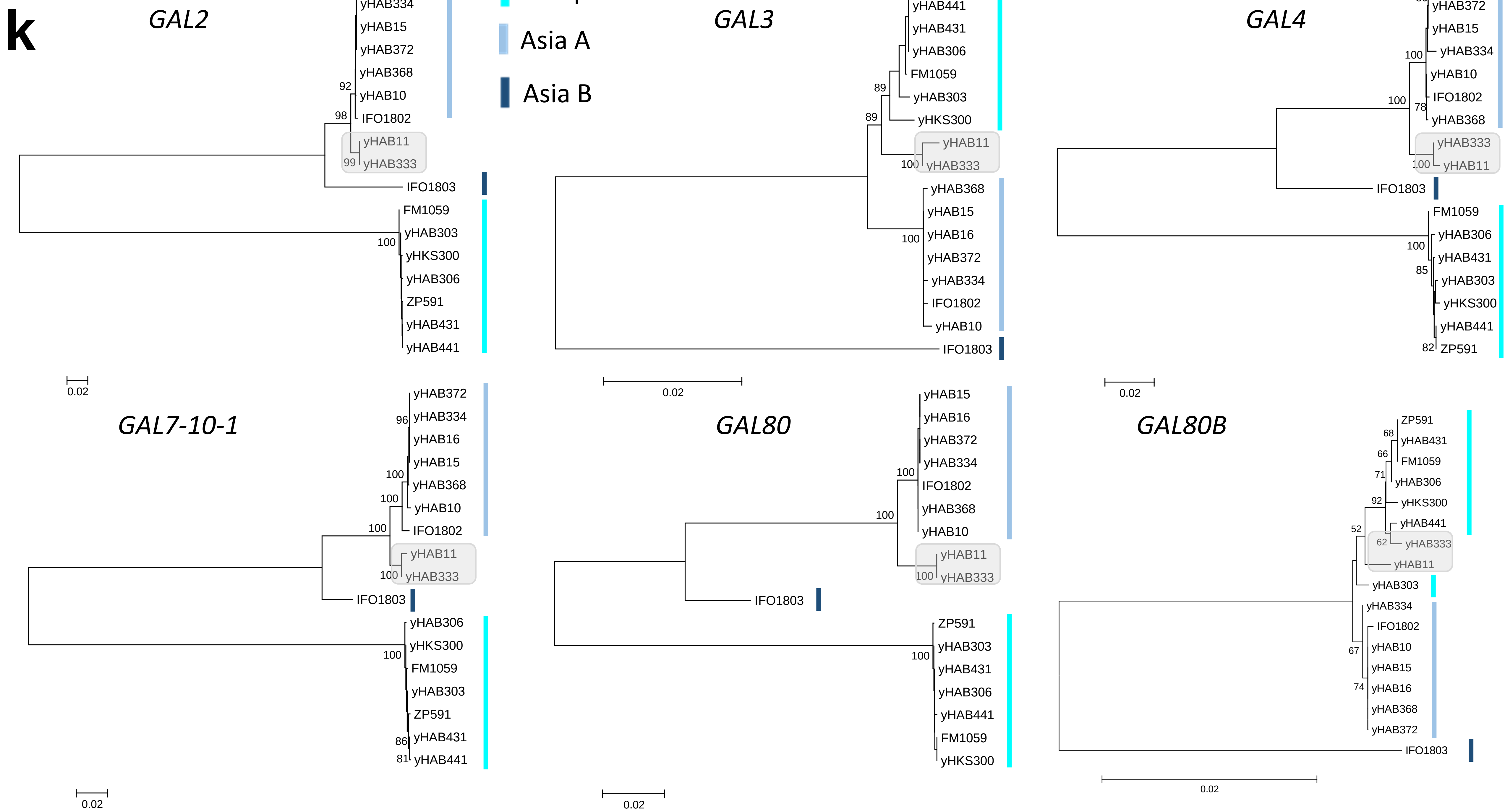

Supplementary Figure 28. Individual phylogenetics trees of the *GAL/MEL* pathway.

Maximum Likelihood (ML) phylogenetic trees of individual genes from the *GAL/MEL* pathway are represented in panels **a-j**. Strain names are colored according to their species designations. Branch support was assessed by using the UltraFast (UF) bootstrap method implemented in `IQTree`. Nodes with UF bootstrap higher than 50 % are reported and colored according to the legend. Panel **k)** displays the Neighbor-Joining (NJ) phylogenetic tree for the *S. kudriavzevii* genes, pseudogenes, and intergenic sequence (covering from one neighboring gene to the other, in the case of *GAL7/GAL10/GAL1*) alignments. Branch support was assessed by using 1,000 non-parametric bootstrap resampling and the Maximum Composite Likelihood model in `MEGA v5`. Panel **b)** and **g)** display the ML phylogenetic tree for the Gal2 and Gal80 protein sequences of *Saccharomyces*, respectively. The scale bars for all panels represented the number of nucleotide substitutions per site, except panel **b)** and **g)** where scale bars represented the number of amino acid substitutions per site. In panel **b)** Gal2 and Gal2b proteins are indicated by A or B, respectively. In panel **g)** Gal80 and Gal80b proteins are indicated by A and B, respectively. Phylogenetic location of T73Bio, DBVPG6044, and Y55 were manually added due to the high degree of gene degeneration, but our findings are supported by recent analyses with additional *S. cerevisiae* and *S. paradoxus* strains [9].

Supplementary Figure 29

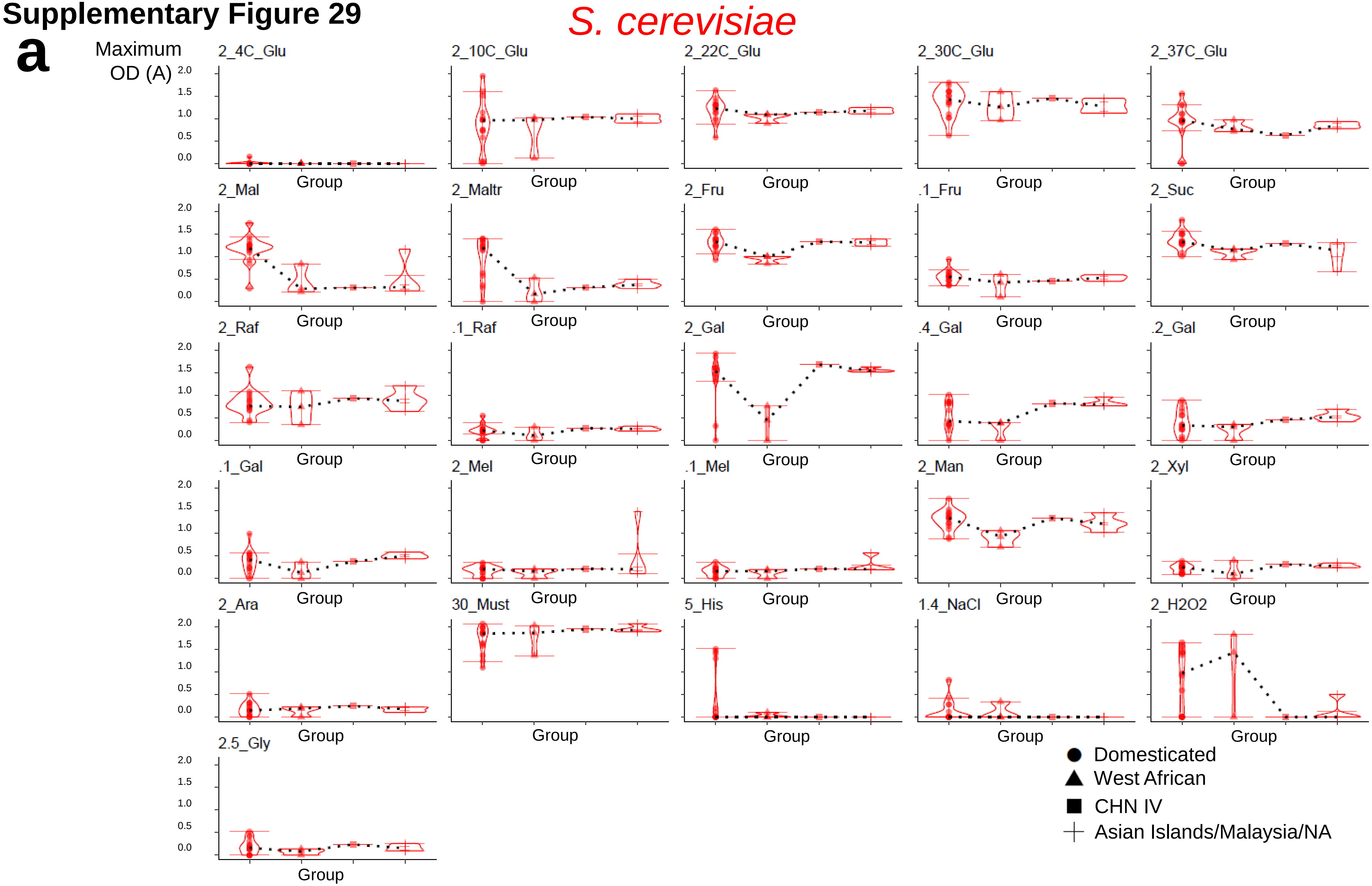

Supplementary Figure 29

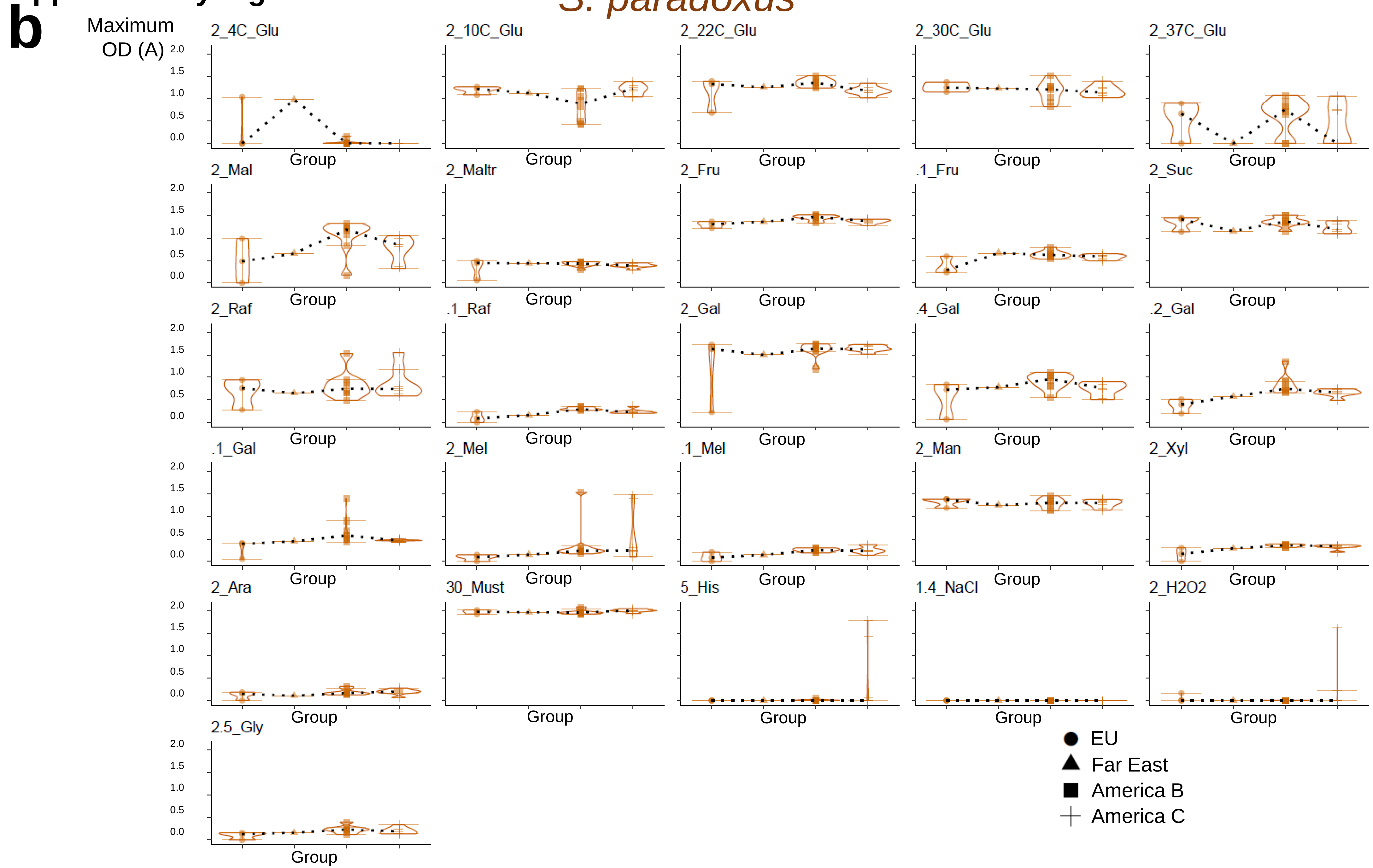

Supplementary Figure 29

S. mikatae

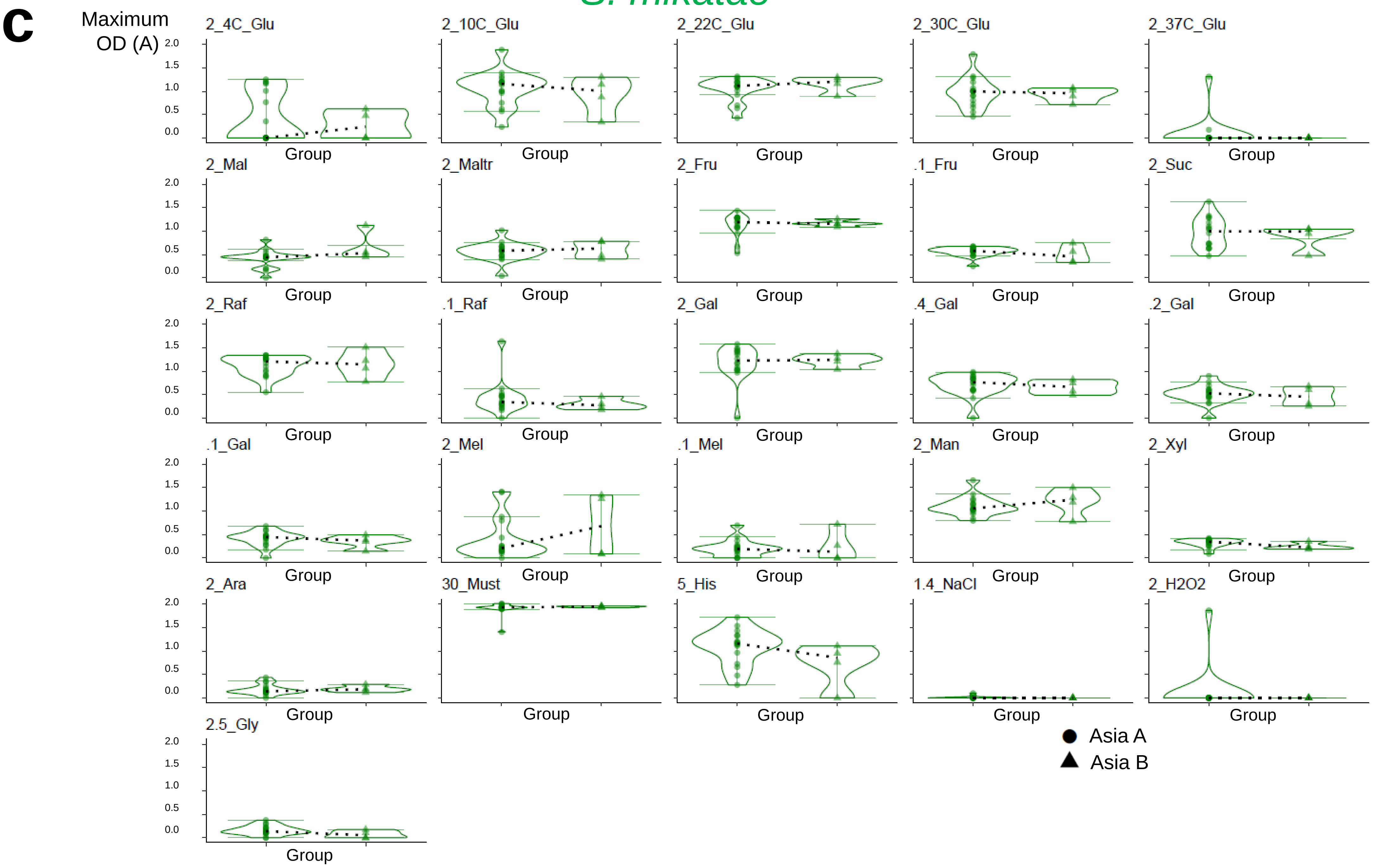

Supplementary Figure 29

S. kudriavzevii

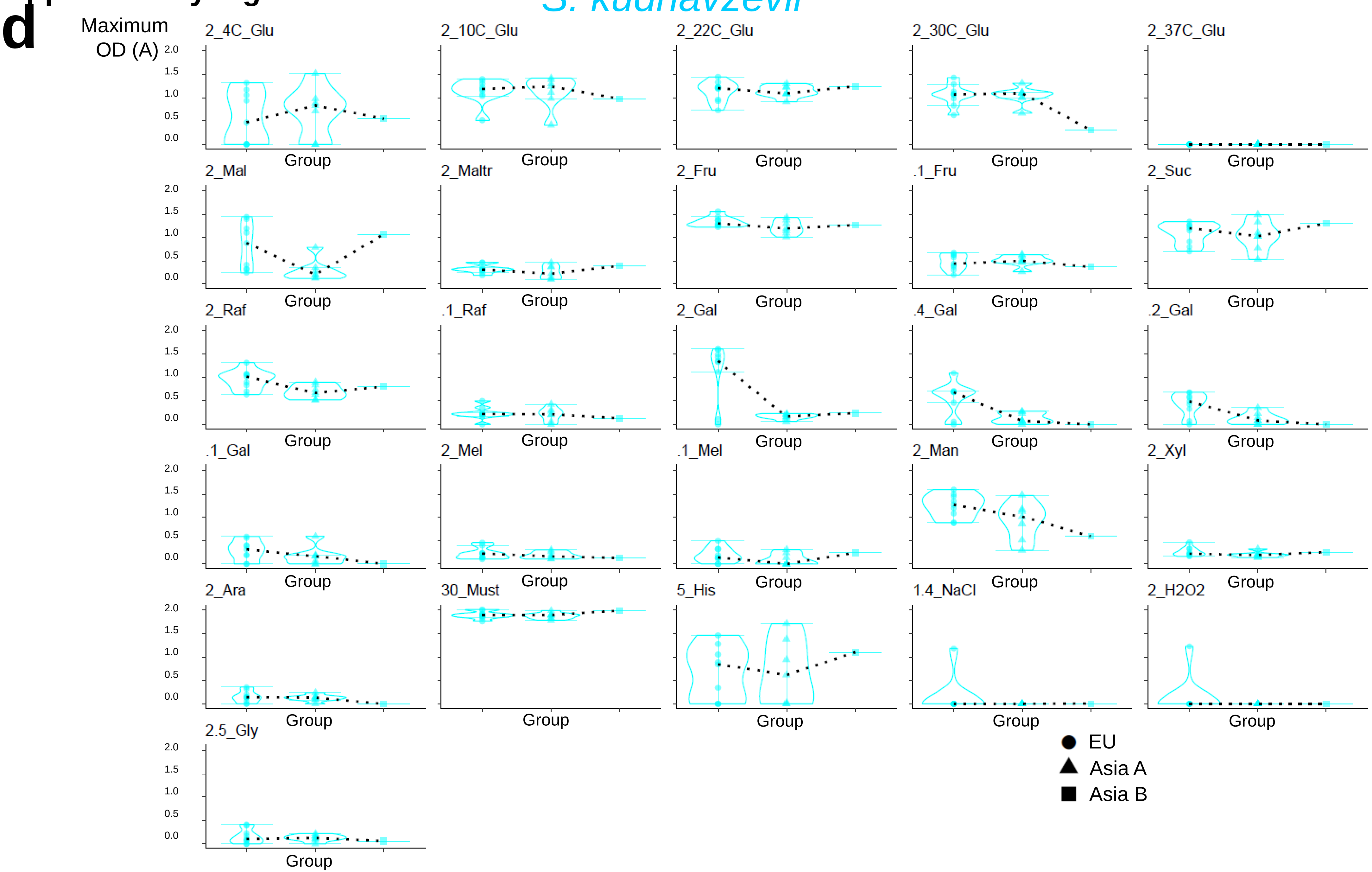

Supplementary Figure 29

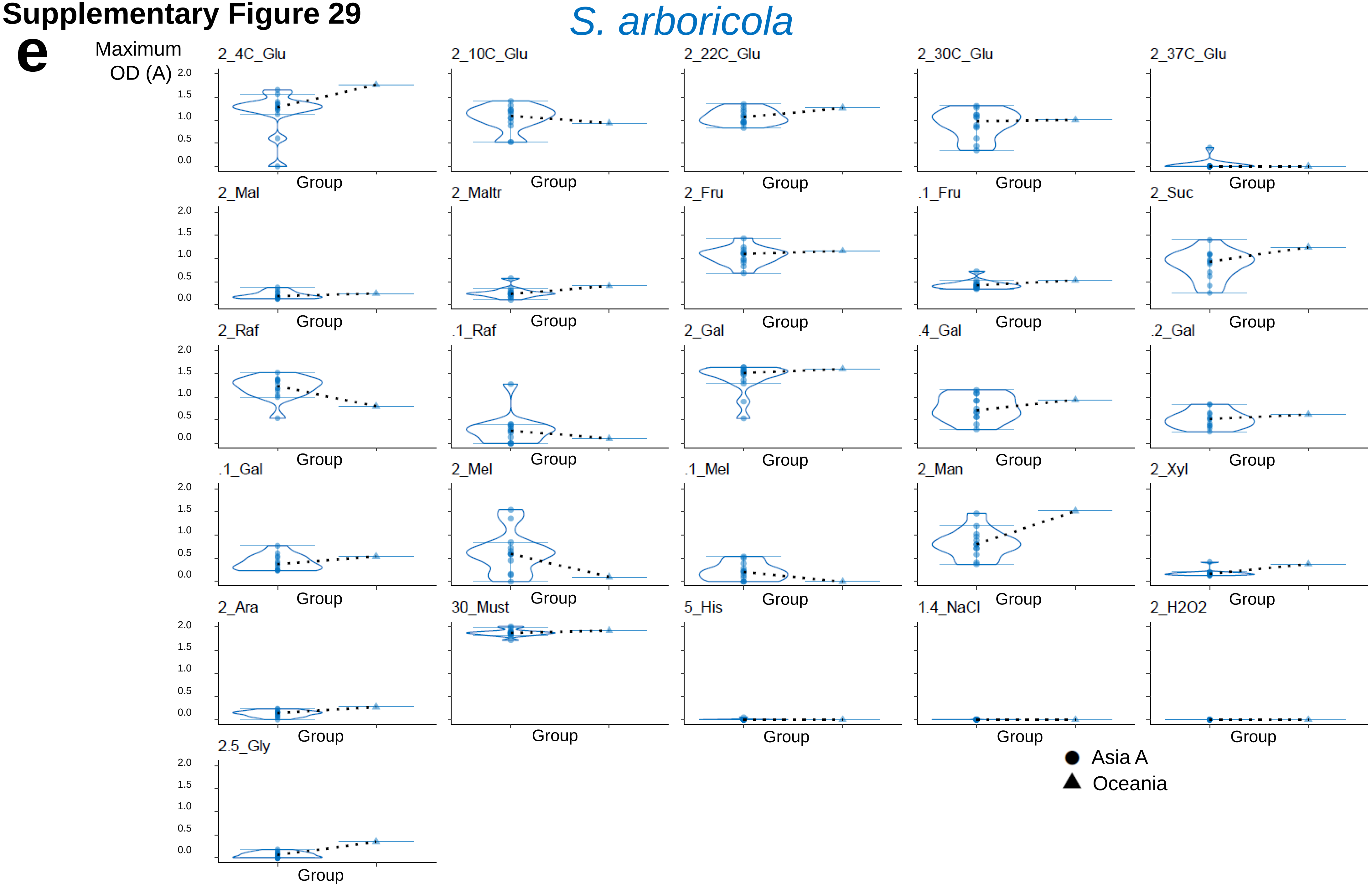

Supplementary Figure 29

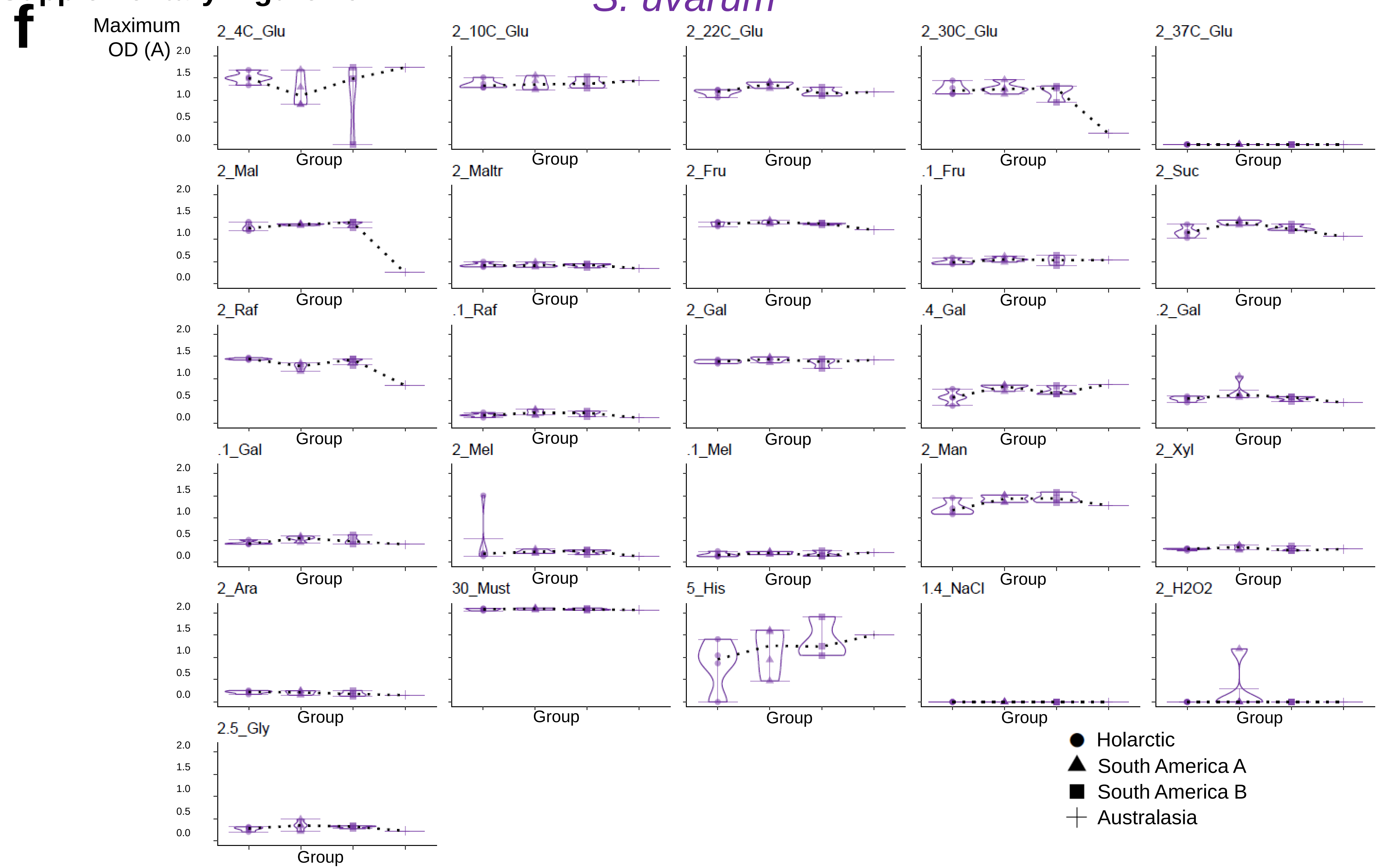

Supplementary Figure 29

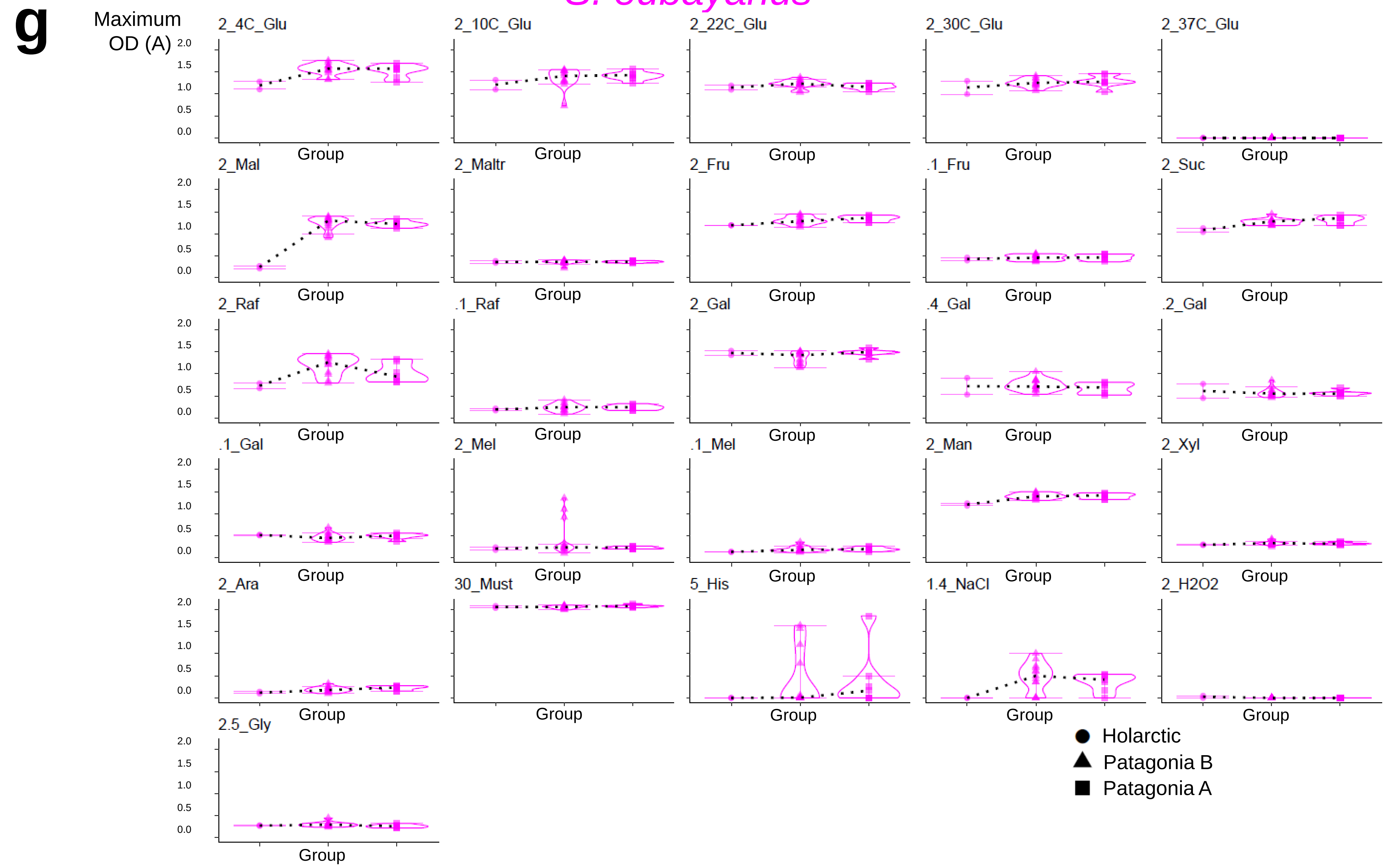

Supplementary Figure 29. Maximum OD_{600} violin boxplots of *Saccharomyces* populations/groups.

Populations/groups of *Saccharomyces* violin boxplots for biomass production (maximum OD_{600}) for *S.* cerevisiae, *S. paradoxus*, *S. mikatae*, *S. kudriavzevii*, *S. arboricola*, *S. uvarum*, *S. eubayanus* are shown in panels **a)**, **b)**, **c)**, **d)**, **e)**, **f)**, and **g)**, respectively, and violin boxplots were colored according to their species designations (Supplementary Data 6). Upper and lower whiskers represent the highest and lowest values of the 1.5 * IQR (inter-quartile range), respectively. Dotted lines connect the median values of each *Saccharomyces* population/group. Shapes highlight different *Saccharomyces* populations or combined groups. 2_4C_Glu: 2 % glucose at 4°C; 2_10C_Glu: 2 % glucose at 10°C; 2_22C_Glu: 2 % glucose at 22°C; 2_30C_Glu: 2 % glucose at 30°C; 2_37C_Glu: 2 % glucose at 37°C; 2_Mal: 2 % maltose; 2_Maltr: 2 % maltotriose; 2_Fru: 2 % fructose; .1_Fru: 0.1 % fructose; 2_Suc: 2 % sucrose; 2_Raf: 2 % raffinose; .1_Raf: 0.1 % raffinose; 2_Gal: 2 % galactose; .4_Gal: 0.4 % galactose; .2_Gal: 0.2 % galactose; .1_Gal: 0.1 % galactose; 2_Mel: 2 % melibiose; .1_Mel: 0.1 % melibiose; 2_Man: 2 % mannose; 2_Xyl: 2 % xylose; 2_Ara: 2 % arabinose; 30_Must: 30 % must; 5_His: 5 g/L histidine; 1.4_NaCl: 1.4 mM NaCl; 2_H2O2: 2mM H_2O_2; 2.5_Gly: 2.5 % glycerol.

Supplementary Figure 30

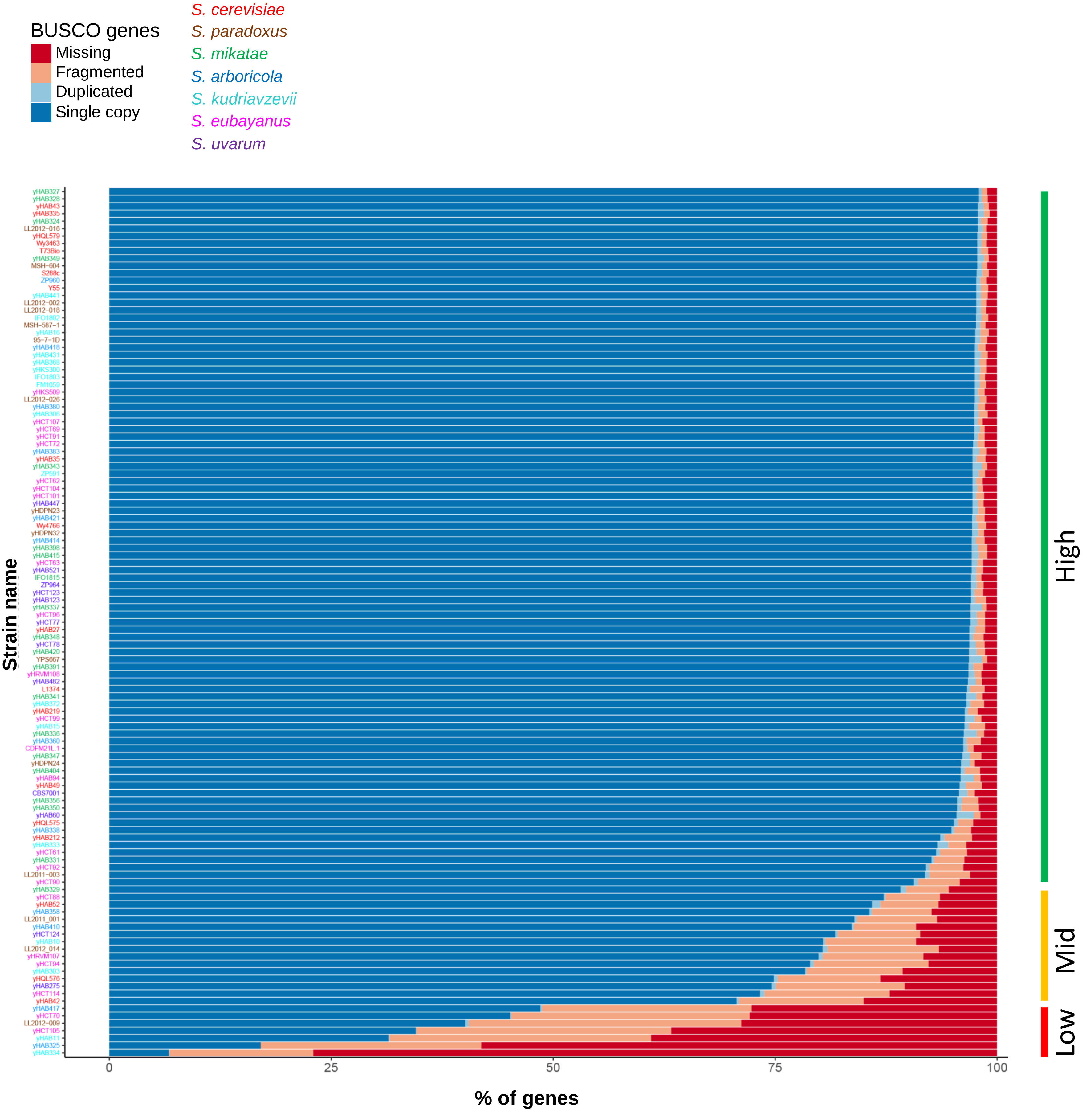

Supplementary Figure 30. Summary statistics of *Saccharomyces* genome assemblies.

Stacked bar plots for the four categories (single-copy, duplicated, fragmented, and missing) of genes annotated by `BUSCO` [10] for each *Saccharomyces* strain assembled (n = 117, Supplementary Data 1). Strain names were colored according to their species designations. Genomes were classified as high, mid, and low quality according to the percentage of single-copy `BUSCO` genes (> 90 % High, between 70 % and 90 % Mid, and < 70 % Low).

References in supplementary figures and notes

1 Naseeb, S.*, et al.*, "Whole genome sequencing, *de novo* assembly and phenotypic profiling for the new budding yeast species *Saccharomyces jurei*," *G3* **8**, 2967-2977 (2018).

2 Musto, H.*, et al.*, "Correlations between genomic GC levels and optimal growth temperatures in prokaryotes," *FEBS Letters* **573**, 73-77 (2004).

3 Kuang, M. C.*, et al.*, "Repeated cis-regulatory tuning of a metabolic bottleneck gene during evolution," *Mol. Biol. Evol.* **35**, 1968-1981 (2018).

4 Hittinger, C. T.*, et al.*, "Remarkably ancient balanced polymorphisms in a multi-locus gene network," *Nature* **464**, 54-58 (2010).

5 Warringer, J.*, et al.*, "Trait variation in yeast is defined by population history," **7**, e1002111 (2011).

6 H Wickham, *ggplot2: elegant graphics for data analysis* (Springer, NY, 2009).

7 Adler, D. and Kelly, S. T., "Vioplot: Violin Plot.," in https://github.com/TomKellyGenetics/vioplot (2020).

8 Kuang, M. C.*, et al.*, "Ongoing resolution of duplicate gene functions shapes the diversification of a metabolic network," *ELife Sciences* **5**, e19027 (2016).

9 Pontes, A.*, et al.*, "Revisiting the taxonomic synonyms and populations of *Saccharomyces cerevisiae* - phylogeny, phenotypes, ecology and domestication," **8** (2020).

10 Waterhouse, R. M.*, et al.*, "BUSCO applications from quality assessments to gene prediction and phylogenomics," *Mol. Biol. Evol.* **35**, 543-548 (2018).
