# Supplementary material for: Macroevolutionary diversity of traits and genomes in the model yeast genus Saccharomyces
Source: Nat Commun. 2023 Feb 8;14:690. doi: 10.1038/s41467-023-36139-2 (PMC9908912; doi:10.1038/s41467-023-36139-2)
Supplement: Supplementary file 2 — Description of Additional Supplementary Files [file 41467_2023_36139_MOESM2_ESM.pdf]

Description of Additional Supplementary Files

File Name: Supplementary Data 1
Description: List of strains used in this study.

File Name: Supplementary Data 2
Description: Sequencing and genome assembly statistics.

File Name: Supplementary Data 3
Description: Genome contributions in admixed and introgressed strains detected in our study.

File Name: Supplementary Data 4
Description: Saccharomyces 2-µm plasmid information.

File Name: Supplementary Data 5
Description: Reciprocal monophyly tests.

File Name: Supplementary Data 6
Description: Kinetic growth parameter information for Saccharomyces strains.

File Name: Supplementary Data 7
Description: PCR primers and conditions.
