# Supplementary material for: Macroevolutionary diversity of traits and genomes in the model yeast genus Saccharomyces
Source: Nat Commun. 2023 Feb 8;14:690. doi: 10.1038/s41467-023-36139-2 (PMC9908912; doi:10.1038/s41467-023-36139-2)
Supplement: Supplementary file 10 — Reporting Summary [file 41467_2023_36139_MOESM10_ESM.pdf]

nature portfolio

Corresponding author(s): Peris D and Hittinger CT

Last updated by author(s): Jan 18, 2023

Reporting Summary

Nature Portfolio wishes to improve the reproducibility of the work that we publish. This form provides structure for consistency and transparency in reporting. For further information on Nature Portfolio policies, see our Editorial Policies and the Editorial Policy Checklist.

Statistics

For all statistical analyses, confirm that the following items are present in the figure legend, table legend, main text, or Methods section.

n/a	Confirmed	
☐	☒	The exact sample size (*n*) for each experimental group/condition, given as a discrete number and unit of measurement
☐	☒	A statement on whether measurements were taken from distinct samples or whether the same sample was measured repeatedly
☐	☒	The statistical test(s) used AND whether they are one- or two-sided<br>*Only common tests should be described solely by name; describe more complex techniques in the Methods section.*
☒	☐	A description of all covariates tested
☐	☒	A description of any assumptions or corrections, such as tests of normality and adjustment for multiple comparisons
☐	☒	A full description of the statistical parameters including central tendency (e.g. means) or other basic estimates (e.g. regression coefficient) AND variation (e.g. standard deviation) or associated estimates of uncertainty (e.g. confidence intervals)
☐	☒	For null hypothesis testing, the test statistic (e.g. *F*, *t*, *r*) with confidence intervals, effect sizes, degrees of freedom and *P* value noted<br>*Give P values as exact values whenever suitable.*
☐	☒	For Bayesian analysis, information on the choice of priors and Markov chain Monte Carlo settings
☒	☐	For hierarchical and complex designs, identification of the appropriate level for tests and full reporting of outcomes
☒	☐	Estimates of effect sizes (e.g. Cohen's *d*, Pearson's *r*), indicating how they were calculated

Our web collection on statistics for biologists contains articles on many of the points above.

Software and code

Policy information about availability of computer code

Data collection	BMG FLUOstar Omega<br>CASAVA v1.8.2<br>iWGS v1.1<br>Github: http://bit.ly/2orfKyT
Data analysis	R v4.0.2 (prcomp) and Rstudio packages: ape v5.4, factoextra v1.0.7, ggbiplot v0.55, ggplot2 v3.3.3, ggpubr v0.4 (ggscatter), ggtree v2.2.4, gridExtra v2.3, pheatmap v4.0.5, phytools v0.7, PopGenome v2.2.4 & v2.7.5, taxize v0.9.99, treeio v1.12, seqinr v4.2, spider v1.5, Staden Package version 1.7<br>AAF v20150930<br>ASTRAL v5.7.7<br>BLAST v2.6<br>BUCKy v1.4.4<br>BUSCO v2.0.1 & v5.1.3<br>bwa v0.7.12<br>CLUMPP v1.1.2<br>DnaSP v5<br>EMBOS package v6.5.7: infoseqout<br>FASconCAT v1.0<br>fineSTRUCTURE v2.0.7<br>GATK v3.1<br>GCAT v6.3<br>Geneious vR6

HARVESTER web v0.6.94
HybPiper v1.2
IQTree v1.6.12 & v2.0.3
iTOL v4.2.3
iWGS v1.1
MAFFT v7.21
MEGA v7
MFannot v1
MrBayes v3.2.3
MuMmer v3.23
NextClip v1.3.1
pal2nal v14
picard v1.98
Pilon v1.22
PopART v1.7
QUAST v3.2
Qualimap v2.2.1
RAxML v8.1
samtools v1.4
SplitsTree 4
spplDer v1
STADEN Package v1.7
STRUCTURE v2.3.4
STRUCTURE PLOT v2
trimal v1.4
Trimmomatic v0.33
YGAP v7

For manuscripts utilizing custom algorithms or software that are central to the research but not yet described in published literature, software must be made available to editors and reviewers. We strongly encourage code deposition in a community repository (e.g. GitHub). See the Nature Portfolio guidelines for submitting code & software for further information.

Data

Policy information about availability of data

All manuscripts must include a data availability statement. This statement should provide the following information, where applicable:

- Accession codes, unique identifiers, or web links for publicly available datasets
- A description of any restrictions on data availability
- For clinical datasets or third party data, please ensure that the statement adheres to our policy

Strains with codes FM[Number] (i.e. FM1198) or yHXX[Number] (i.e. yHAB33) are physically present and may be requested from cthittinger@wisc.edu (Supplementary Data 1). Strains that are also available from the Portuguese Yeast Culture Collection (PYCC) are indicated with PYCC accession numbers in Supplementary Data 1; most were deposited as part of a previous study by Peris et al. 32. For the rest of the strains, references are provided in Supplementary Data 1 to request them from the corresponding lab. The COX2 and COX3 sequences generated in this study were deposited in GenBank under accession nos. MH813536-MH813939. The GAL genes that were Sanger-sequenced in this study were deposited in GenBank under accession nos. OL660614-OL660618. Illumina sequencing data generated in this study have been deposited in NCBI's SRA database under accession Bioproject code PRJNA475869 [https://www.ncbi.nlm.nih.gov/bioproject/?term=PRJNA475869]. Genome assemblies and annotations generated in this study are available on the European Nucleotide Archive (ENA) under project accession code PRJEB48264 [https://www.ebi.ac.uk/ena/browser/view/PRJEB48264]. Accession numbers of downloaded Illumina sequences or genome assemblies are provided in the Supplementary Data 2. Details regarding the location of source data for Figures 2-6, as well as Supplementary Figures 3-15, and 17-29 can be found under the 'Source Data' heading of the Github repository, https://perisd.github.io/Sac2.0/. Raw data generated in this study is deposited in FigShare (https://dx.doi.org/10.6084/m9.figshare.17185874).

Field-specific reporting

Please select the one below that is the best fit for your research. If you are not sure, read the appropriate sections before making your selection.

☒ Life sciences ☐ Behavioural & social sciences ☐ Ecological, evolutionary & environmental sciences

For a reference copy of the document with all sections, see nature.com/documents/nr-reporting-summary-flat.pdf

Life sciences study design

All studies must disclose on these points even when the disclosure is negative.

Sample size	Sample size was not determined using statistical methods. The selected number of strains covers the geographic location of known species.
Data exclusions	In cases where growth was not detected but exaggerated OD values were observed and the plate pictures showed evidence for flocculation, we removed the exaggerated values.

Replication	Average, median, and standard deviations of kinetic parameters from three independent biological replicates. Successful replicates are indicated in Supplementary Data 6.
Randomization	Strain location in the 96-well plates was randomized for each replicate.
Blinding	It was not necessary because strains were not allocated to particular groups.

Reporting for specific materials, systems and methods

We require information from authors about some types of materials, experimental systems and methods used in many studies. Here, indicate whether each material, system or method listed is relevant to your study. If you are not sure if a list item applies to your research, read the appropriate section before selecting a response.

Materials & experimental systems

n/a	Involved in the study
☒	☐ Antibodies
☒	☐ Eukaryotic cell lines
☒	☐ Palaeontology and archaeology
☒	☐ Animals and other organisms
☒	☐ Human research participants
☒	☐ Clinical data
☒	☐ Dual use research of concern

Methods

n/a	Involved in the study
☒	☐ ChIP-seq
☒	☐ Flow cytometry
☒	☐ MRI-based neuroimaging
